# Transcriptomic changes in the pre-implantation uterus highlight histotrophic nutrition of the developing marsupial embryo

**DOI:** 10.1038/s41598-018-20744-z

**Published:** 2018-02-05

**Authors:** Camilla M. Whittington, Denis O’Meally, Melanie K. Laird, Katherine Belov, Michael B. Thompson, Bronwyn M. McAllan

**Affiliations:** 1 The University of Sydney, Sydney School of Veterinary Science, Camperdown, NSW 2006 Australia; 2 The University of Sydney, School of Life and Environmental Sciences, Camperdown, NSW 2006 Australia; 3 The University of Sydney, School of Medical Sciences, Camperdown, NSW 2006 Australia; 4 Present Address: Center for Gene Therapy, Beckman Research Institute of City of Hope, Duarte, CA 91024 USA

**Keywords:** Evolutionary biology, Animal physiology

## Abstract

Early pregnancy is a critical time for successful reproduction; up to half of human pregnancies fail before the development of the definitive chorioallantoic placenta. Unlike the situation in eutherian mammals, marsupial pregnancy is characterised by a long pre-implantation period prior to the development of the short-lived placenta, making them ideal models for study of the uterine environment promoting embryonic survival pre-implantation. Here we present a transcriptomic study of pre-implantation marsupial pregnancy, and identify differentially expressed genes in the *Sminthopsis crassicaudata* uterus involved in metabolism and biosynthesis, transport, immunity, tissue remodelling, and uterine receptivity. Interestingly, almost one quarter of the top 50 genes that are differentially upregulated in early pregnancy are putatively involved in histotrophy, highlighting the importance of nutrient transport to the conceptus prior to the development of the placenta. This work furthers our understanding of the mechanisms underlying survival of pre-implantation embryos in the earliest live bearing ancestors of mammals.

## Introduction

While eutherian mammals primarily nourish their embryos via a placenta, a key feature of marsupial reproduction is a very short period of placentation during a short gestation, followed by an extended investment in lactation^1^. In eutherians, the embryo becomes closely apposed to the uterine epithelium, before implanting into the uterine tissue very early in pregnancy to form the placenta e.g.^2–5^. In contrast, marsupial implantation and placentation do not occur until at least two thirds of the way through pregnancy, making marsupials ideal models for studying the uterine environment required for survival of the mammalian early embryo. In marsupials, the embryo remains unattached within the uterine lumen for most of pregnancy, and is reliant on uterine secretions for nutrient supply^4,6^. The conceptus is coated in several layers, including a tough outer shell coat secreted by the epithelial cells and endometrial glands of the utero-tubal junction and cranial part of the uterus^7,8^. The shell coat persists until implantation, and is permeable to gases and other small molecules of up to 40 kDa in size, permitting histotrophic nutrition^9^. The shell coat may also prevent maternal immune attack of the embryo^8^.

At implantation, the embryo hatches from the shell coat, enabling placentation through direct contact between the trophoblast and the receptive maternal uterine epithelium^3,10^. Placentation in marsupials has been well-studied from morphological e.g.^5,11,12^, physiological e.g.^13,14^ and genetic e.g.^15–17^ perspectives. In contrast, pre-implantation marsupial pregnancy has received much less attention, particularly from genetic studies, which have focused on the immunological changes in the uterus^15,18^. Understanding the complete physiology of pre-implantation marsupial pregnancy is important, because this period represents the majority of gestation, when the embryo is growing and undergoing early organogenesis^19^. The physiology of this period of mammalian pregnancy is an important area of medical research e.g.^20^, due to the high rate of human pregnancy failure [~40–50% of human pregnancies are lost before 20 weeks, 75% of which have been attributed to implantation failure^21^]. Failure to implant is also a major impediment to assisted reproductive technologies such as IVF^21^. As successful establishment of pregnancy requires both a healthy conceptus and a receptive uterus, information about both the maternal and the embryonic components during mammalian pregnancy is required to fully understand implantation^22^.

In this study, we describe the uterine transcriptome of the model marsupial *Sminthopsis crassicaudata* (fat-tailed dunnart) in the period of pre-implantation uterine receptivity. The fat-tailed dunnart has a very brief (13.5 day) pregnancy^23^. Prior to implantation, which occurs around day 10 of pregnancy, the conceptus lies closely apposed to maternal tissues within folds of the uterine epithelium^8,24,25^. Subsequently, a yolk sac placenta forms, which erodes part of the maternal epithelium but does not breach maternal capillaries i.e. endotheliochorial placentation^3^. As the pre-implantation shelled embryo spends twice as long in the uterus as the period of placental attachment, modifications of the uterine environment for efficient gas, nutrient and waste transport must occur during the pre-implantation phase early in pregnancy. The ultrastructural modifications to cell-cell adhesion in the early pregnant *S. crassicaudata* uterus are possibly related to these functional requirements^12,26,27^. Here, we describe the uterine pre-implantation transcriptome in *S. crassicaudata* and identify the broad genetic underpinnings of maternal maintenance of the early marsupial conceptus during pregnancy. We focus on identifying the genes underpinning nutrient transport, which we hypothesise are critical in nourishing the developing embryo prior to the formation of the placenta.

## Results

### Transcriptome sequencing and annotation

Our transcriptome sequencing recovered ~29–35 million paired reads from each of 3 pregnant (days 6–8 of pregnancy) and 3 non-pregnant dunnart uteri. After normalisation, 50.7 million reads were assembled into 234,671 transcripts from 136,066 ‘genes’ using Trinity^28^. The longest was 25,519 bp, the shortest 201 bp and the mean length 1,371.3 bp. We assessed the assembly completeness using BUSCO^29^ and recovered 90% complete or partial alignments of 3950 mammalian orthologs. All sequence data have been uploaded to GenBank (BioProject ID PRJNA399240). We used Kallisto^30^ to estimate abundance and DESeq2^31^ to call differential expression. In total, 1,871 transcripts were differentially expressed between pregnant and non-pregnant animals (FDR-adjusted *P* < 0.001). Approximately 43% of these differentially regulated transcripts were annotated by Trinotate v3.0.2^28^; on the basis of similarity to known genes in the PFam (v31.0) and SwissProt (release 2017_2) databases. Pearson correlation and Principal Component analyses of gene expression data across all samples show that gene expression is more highly correlated within sample groups than between them (Supplementary Figure 1). The 50 most significantly up- and down-regulated genes were identified for further analysis (Tables 1 and 2).

**Table 1:** The top 50 significantly up-regulated annotated genes during pregnancy, ranked by adjusted P-value, displaying best BLAST hit HUGO Gene Symbol, log2 ratios, and FDR‐adjusted p‐values, along with mean expression values per stage.

Gene symbol	Gene name	Mean pregnant expression	Mean non-pregnant expression	log2 Fold Change	Adjusted P-value	Putative Function
*GUCY2C*	Guanylate Cyclase 2C	110.2	0.1	9.7	1.12E-38	Transmembrane receptor
*SDR42E2*	Short Chain Dehydrogenase/Reductase Family 42E, Member 2	437.1	0.3	9.0	2.40E-25	Oxidoreductase activity
*PLA2G10*	Phospholipase A2 Group X	133.1	3.1	5.5	2.32E-20	Lipid hydrolysis
*MOCS2*	Molybdenum Cofactor Synthesis 2	2174.4	22.0	7.3	8.60E-19	Biosynthesis
*MIR639*	MicroRNA 639	22.9	2.8	3.7	9.73E-18	microRNA, regulatory
*TECR*	Trans-2,3-Enoyl-CoA Reductase	22.9	2.8	3.7	9.73E-18	Fatty acid synthesis
*PLA2G3*	Phospholipase A2 Group III	1.6	0.1	4.7	4.87E-16	Lipid hydrolysis
*APOL6*	apolipoprotein L6	151.1	16.1	3.2	1.36E-14	Lipid movement
*S100P*	S100 Calcium Binding Protein P	268.3	0.2	7.9	5.86E-14	Regulation of cellular processes
*STC1*	stanniocalcin 1	3962.9	39.4	6.2	7.96E-14	Calcium and phosphate transport
*GGT1*	Gamma-Glutamyltransferase 1	73.4	2.3	4.9	1.88E-12	Metabolism
*RDH16*	Retinol Dehydrogenase 16 (All-Trans)	42.0	0.7	5.6	8.51E-12	Metabolism
*LRRC31*	Leucine Rich Repeat Containing 31	35.1	1.2	5.2	8.51E-12	Unknown
*SLC2A12*	Solute Carrier Family 2 Member 12	82.8	3.4	4.2	9.84E-12	Glucose transport
*AKR1D1*	Aldo-Keto Reductase Family 1 Member D1	190.3	0.3	7.1	1.45E-11	Steroid hormone reduction
*EHF*	ETS Homologous Factor	179.1	14.0	3.9	1.83E-11	Epithelial cell differentiation
*FZD5*	Frizzled Class Receptor 5	5.9	0.5	3.6	4.97E-11	Wnt signalling
*FGFR1*	fibroblast growth factor receptor 1	141.4	28.4	2.6	1.03E-10	Cell differentiation
*IDO1*	Indoleamine 2,3-Dioxygenase 1	158.9	3.5	5.2	1.14E-10	Protection of the fetus from maternal immune rejection
*CCDC129*	Coiled-Coil Domain Containing 129	1.9	0.0	6.1	2.06E-10	Receptor binding
*BCO1*	Beta-Carotene Oxygenase 1	4.0	0.1	5.8	4.18E-10	Metabolism of beta-carotene to vitamin A
*FOXN4*	Forkhead Box N4	370.7	23.2	4.1	5.76E-10	Transcriptional regulation
*LRRC26*	Leucine Rich Repeat Containing 26	370.7	23.2	4.1	5.76E-10	Regulation of potassium channels
*GRIN1*	glutamate ionotropic receptor NMDA type subunit 1	370.7	23.2	4.1	5.76E-10	Ion channel
*HSD3B7*	Hydroxy-Delta-5-Steroid Dehydrogenase, 3 Beta- And Steroid Delta-Isomerase 7	2298.1	39.5	4.9	7.01E-10	Bile synthesis from cholesterol; Part of enzymatic system biosynthesising steroids
*CYP27A1*	Cytochrome P450 Family 27 Subfamily A Member 1	295.7	46.8	2.9	1.51E-09	Metabolism and biosynthesis
*ATP13A3*	ATPase 13A3	125.9	33.4	2.2	2.34E-09	Cation transport across membranes
*MFSD4A*	Major Facilitator Superfamily Domain Containing 4A	12.3	0.2	5.5	2.93E-09	Transmembrane transport
*CARNS1*	Carnosine Synthase 1	15.8	0.7	4.9	7.66E-09	Metabolism
*ZNF750*	Zinc Finger Protein 750	2.6	0.0	6.0	9.63E-09	Transcription factor mediating cell differentiation
*CCDC28A*	Coiled-Coil Domain Containing 28A	49.3	10.9	2.5	1.07E-08	Protein binding
*IL22RA1*	interleukin 22 receptor subunit alpha 1	127.5	14.6	3.3	1.38E-08	Class II cytokine receptor in innate immune response
*TRAT1*	T Cell Receptor Associated Transmembrane Adaptor 1	3.3	0.5	3.0	1.94E-08	T-cell receptor stabilisation
*LY9*	Lymphocyte Antigen 9	2.6	0.1	4.5	2.89E-08	Modulation of immune cell activity (innate and adaptive)
*SEC62*	SEC62 homolog, preprotein translocation factor	223.0	39.3	2.8	3.18E-08	Protein transport through ER
*ADPGK*	ADP Dependent Glucokinase	50.7	20.3	1.6	4.01E-08	Glycolysis
*BPI*	Bactericidal/Permeability-Increasing Protein	7973.0	8.2	6.3	9.98E-08	Antimicrobial (gram-negative organisms)
*DIP2B*	Disco Interacting Protein 2 Homolog B	12.4	5.5	1.6	1.02E-07	Transcriptional regulation
*LETM2*	Leucine Zipper And EF-Hand Containing Transmembrane Protein 2	12.4	5.5	1.6	1.02E-07	Ribosome binding
*SLC27A2*	solute carrier family 27 member 2	180.1	1.6	5.5	1.40E-07	Fatty acid transport
*SC5D*	Sterol-C5-Desaturase	294.8	13.7	4.3	1.91E-07	Cholesterol biosynthesis
*SLC35D2*	solute carrier family 35 (UDP-GlcNAc/UDP-glucose transporter), member D2	155.8	8.6	4.1	2.09E-07	Nucleoside sugar transport
*TMEM213*	Transmembrane Protein 213	301.1	3.2	5.5	2.32E-07	Membrane component
*SLC35C1*	Solute carrier family 35 member C1	63.8	7.5	3.3	2.52E-07	Nucleoside sugar transport
*SLC16A6*	Solute carrier family 16 member 6	92.4	2.5	5.3	2.52E-07	Lactic acid/ketone
*MICALCL*	MICAL C-Terminal Like	5.3	0.6	3.4	2.81E-07	Signal transduction
*ALG12*	ALG12, Alpha-1,6-Mannosyltransferase	52.8	20.1	1.9	2.81E-07	Protein glycosylation
*SLCO4A1*	solute carrier organic anion transporter family member 4A1	37.6	3.7	3.4	3.11E-07	Bicarbonate transport
*HDC*	Histidine Decarboxylase	102.7	0.4	5.9	4.33E-07	Histamine production
*SH2D1B*	SH2 Domain Containing 1B	1.9	0.2	3.3	4.35E-07	Signal transduction in immune cells

Mean expression values are normalized transcripts per million (TPM).

**Table 2:** The top 50 significantly down-regulated annotated genes during pregnancy, ranked by adjusted P-value, displaying best BLAST hit HUGO Gene Symbol, log2 ratios, and FDR‐adjusted p‐values, along with mean expression values per stage.

Gene Symbol	Gene name	Mean pregnant expression	Mean non-pregnant expression	log2 Fold Change	Adjusted P-value	Putative Function
*MUC5AC*	Mucin 5AC, Oligomeric Mucus/Gel-Forming	0.1	58.6	−8.3	4.57E-38	Extracellular matrix
*COL7A1*	collagen type VII alpha 1 chain	0.1	2.6	−4.8	2.66E-18	Anchoring of basement membrane
*CBX2*	Chromobox 2	1.5	13.6	−2.8	1.35E-15	Transcriptional repression
*PGBD1*	PiggyBac Transposable Element Derived 1	2.9	25.8	−2.7	1.93E-15	Unknown
*IGHV4-28*	Immunoglobulin Heavy Variable 4-28	0.7	99.0	−6.2	3.13E-15	Antigen recognition
*CNTN2*	contactin 2	0.0	3.0	−5.7	2.23E-13	Cell adhesion
*SLCO2A1*	solute carrier organic anion transporter family member 2A1	2.2	32.2	−3.4	4.70E-13	Prostaglandin release
*SHF*	Src Homology 2 Domain Containing F	0.9	9.4	−2.9	1.23E-12	Regulation of apoptosis
*PTGFR*	Prostaglandin F Receptor	0.1	7.5	−5.2	1.63E-12	Receptor for prostaglandin F2-alpha; uterine contraction
*ADGRB2*	adhesion G protein-coupled receptor B2	0.1	5.2	−4.3	3.23E-12	Inhibition of angiogenesis
*CD200*	CD200 Molecule	10.8	152.6	−3.3	7.12E-12	Immunosuppression, T-cell proliferation
*GPR153*	G protein-coupled receptor 153	0.8	7.8	−2.9	1.82E-11	Signalling
*ZNF497*	Zinc Finger Protein 497	0.7	8.4	−3.1	5.25E-11	Transcriptional regulation
*KRT77*	Keratin 77	0.1	9.6	−5.3	7.34E-11	Epithelial cell structure
*CENPF*	Centromere Protein F	4.1	20.0	−2.1	9.29E-11	Mitosis
*ZC2HC1A*	Zinc Finger C2HC-Type Containing 1A	2.1	10.9	−2.2	9.29E-11	Unknown
*IGKV1D-43*	Immunoglobulin Kappa Variable 1D-43	0.7	181.3	−6.3	2.07E-10	Antigen recognition
*ROBO1*	Roundabout Guidance Receptor 1	1.8	19.6	−2.7	2.13E-10	Mediation of cellular migration
*CRISPLD1*	Cysteine Rich Secretory Protein LCCL Domain Containing 1	0.2	3.0	−3.7	2.32E-10	Component of extracellular region
*LEPR*	leptin receptor	4.0	166.7	−4.4	2.32E-10	Regulation of fat metabolism
*GREB1*	growth regulation by estrogen in breast cancer 1	0.0	1.1	−5.7	2.40E-10	Estrogen-simulated cell proliferation
*CNTFR*	ciliary neurotrophic factor receptor	1.4	26.2	−3.4	2.94E-10	Interleukin signalling
*MIR5001*	MicroRNA 5001	1.6	13.1	−2.6	2.97E-10	Post-transcriptional regulation
*C14orf180*	Chromosome 14 Open Reading Frame 180	3.2	17.9	−2.2	3.06E-10	Plasma membrane component
*TGIF2*	TGFB Induced Factor Homeobox 2	1.1	13.3	−3.2	4.25E-10	Transcriptional repression
*KIF26B*	kinesin family member 26B	0.5	10.0	−3.8	4.42E-10	Cytoskeleton
*COL7A1*	collagen type VII alpha 1 chain	0.1	5.7	−5.1	4.44E-10	Anchoring of basement membrane
*PTGER3*	Prostaglandin E Receptor 3	1.6	11.3	−2.6	6.98E-10	Receptor for prostaglandin E2; uterine contraction
*EDN3*	endothelin 3	0.0	11.4	−6.4	7.19E-10	Vasoconstriction
*CDC42EP3*	CDC42 Effector Protein 3	2.6	21.7	−2.6	8.30E-10	Actin cytoskeleton reorganisation
*KIF7*	Kinesin Family Member 7	0.4	3.8	−2.7	1.45E-09	Signalling; cilia-associated
*NCKAP5*	NCK Associated Protein 5	0.3	1.8	−2.3	1.51E-09	Unknown
*SALL4*	Spalt Like Transcription Factor 4	0.6	4.0	−2.3	2.21E-09	Transcription factor
*NYNRIN*	NYN Domain And Retroviral Integrase Containing	0.3	3.1	−2.7	2.62E-09	RNA binding
*IGKV3D-11*	Immunoglobulin Kappa Variable 3D-11	0.0	38.0	−6.5	2.79E-09	Antigen recognition
*FREM2*	FRAS1 related extracellular matrix protein 2	0.2	1.9	−3.0	2.85E-09	Basement membrane component; epidermal adhesion
*MEX3A*	Mex-3 RNA Binding Family Member A	0.7	7.6	−2.9	2.93E-09	RNA binding
*JCHAIN*	Joining Chain Of Multimeric IgA And IgM	4.6	456.8	−5.3	5.05E-09	Antigen recognition
*AKR1B1*	Aldo-keto reductase family 1, member B1 (aldose reductase)	11.8	66.3	−2.0	6.85E-09	Sugar metabolism
*SMOC2*	SPARC related modular calcium binding 2	43.5	491.6	−3.0	6.85E-09	Cell matrix; cell proliferation; angiogenesis
*IGHV3-23*	Immunoglobulin Heavy Variable 3-23	0.9	54.0	−4.9	8.50E-09	Antigen recognition
*CASR*	Calcium Sensing Receptor	0.3	6.7	−4.4	8.64E-09	Intracellular signalling
*NINL*	Ninein Like	0.5	10.3	−3.7	8.87E-09	Mitosis
*NRG1*	Neuregulin 1	0.3	4.9	−3.9	9.31E-09	Cell signalling
*IGLV1-51*	Immunoglobulin Lambda Variable 1-51	0.0	82.6	−6.4	1.08E-08	Antigen recognition
*DACT1*	Dishevelled Binding Antagonist Of Beta Catenin 1	1.3	14.6	−3.0	1.16E-08	Intracellular signalling
*TCTN3*	Tectonic Family Member 3	3.0	16.6	−2.0	1.26E-08	Ciliogenesis
*IFIT5*	Interferon Induced Protein With Tetratricopeptide Repeats 5	1.9	16.1	−2.6	1.27E-08	RNA binding to viral RNAs
*LRRN3*	Leucine Rich Repeat Neuronal 3	0.3	5.1	−3.3	1.80E-08	Protein binding
*IGHA1*	Immunoglobulin Heavy Constant Alpha 1	17.0	1722.2	−5.3	2.01E-08	Antigen recognition

Mean expression values are normalized transcripts per million (TPM).

### Gene ontology analysis

We conducted analyses of gene ontology for differentially expressed *S. crassicaudata* genes and identified broad functional categories on which to focus our analysis. These analyses are ideal for examining system-level gene expression changes in non-model species^32^. GO functional annotation of transcripts upregulated in pregnant compared with non-pregnant uteri identified 102 GO terms (Supplementary Table 1). In particular, there was significant enrichment for genes involved in metabolism, biosynthesis, lipid metabolism, transport and cellular structures (Supplementary Figure 2). There were 269 significantly enriched Gene Ontology categories for genes that are downregulated during pregnancy (Supplementary Table 2). There was enrichment for genes involved in development, transport, cell signalling, morphogenesis, metabolism and cellular structures membrane (Supplementary Figure 3). KEGG pathway analysis of pregnancy-upregulated genes showed significant enrichment of 13 pathways involved in metabolism, biosynthesis, lysosome, peroxisome, protein processing and export, signalling, one of which (metabolic pathways) survived Benjamini-Hochberg correction (Table 3). In contrast, KEGG pathway analysis of downregulated genes during pregnancy showed significant enrichment of 11 pathways involved in axon function, cell cycle, signalling, cancer, cell adhesion, metabolism, and receptor interaction, none of which survived Benjamini-Hochberg correction (Table 4).

**Table 3:** KEGG pathways analysis using DAVID of genes upregulated during pregnancy.

Pathway accession	Pathway Term	Count	%	P-Value	Genes	Fold Enrichment	Benjamini-adjusted P-value	FDR
mdo01100	Metabolic pathways	40	15.1	6.8E-07	*GALNT3, ALAD, SC5D, TALDO1, NAGS, ADPGK, HSD3B7, PAFAH2, EHHADH, ALG2, HMGCS1, GMPPB, ATP6V0C, CEPT1, PGP, ACSL1, DHCR7, HDC, ACAD8, IPMK, GALNT12, HSD17B7, MOCS2, PLA2G10, SLC33A1, PDXP, DPAGT1, IDO1, MGAT2, CYP27A1, MLYCD, SQLE, BCO1, AGXT2, PLA2G3, RDH16, AKR1D1, ALG12, PC, MDH1*	2.2	1.03E-04	0.0
mdo00100	Steroid biosynthesis	4	1.5	2.9E-03	*SC5D, SQLE, DHCR7, HSD17B7*	13.6	1.94E-01	3.4
mdo01130	Biosynthesis of antibiotics	10	3.8	4.0E-03	*SC5D, PGP, TALDO1, ADPGK, PAFAH2, SQLE, EHHADH, HMGCS1, HSD17B7, MDH1*	3.2	1.82E-01	4.7
mdo00120	Primary bile acid biosynthesis	3	1.1	1.9E-02	*CYP27A1, HSD3B7, AKR1D1*	13.8	5.13E-01	20.4
mdo00565	Ether lipid metabolism	4	1.5	2.6E-02	*CEPT1, PLA2G10, PAFAH2, PLA2G3*	6.1	5.52E-01	27.3
mdo01200	Carbon metabolism	6	2.3	2.8E-02	*PGP, TALDO1, ADPGK, EHHADH, PC, MDH1*	3.5	5.07E-01	28.5
mdo04142	Lysosome	6	2.3	3.5E-02	*ATP6V0C, NAGPA, MFSD8, AP3D1, CD164, AP4S1*	3.3	5.34E-01	34.5
mdo04146	Peroxisome	5	1.9	3.8E-02	*ACSL1, MLYCD, EHHADH, GNPAT, SLC27A2*	3.9	5.23E-01	37.4
mdo04141	Protein processing in endoplasmic reticulum	7	2.6	4.0E-02	*HYOU1, SYVN1, PDIA6, HSPA5, DNAJC3, LMAN1, SEC62*	2.7	4.96E-01	38.7
mdo00510	N-Glycan biosynthesis	4	1.5	4.1E-02	*MGAT2, ALG2, DPAGT1, ALG12*	5.2	4.69E-01	39.4
mdo03060	Protein export	3	1.1	5.2E-02	*SRPRA, HSPA5, SEC62*	8.1	5.19E-01	47.1
mdo03320	PPAR signaling pathway	4	1.5	7.8E-02	*ACSL1, CYP27A1, EHHADH, SLC27A2*	4.0	6.39E-01	62.0
mdo00410	beta-Alanine metabolism	3	1.1	8.2E-02	*MLYCD, EHHADH, CARNS1*	6.2	6.28E-01	63.9

P-values are modified Fisher’s Exact P-Values for gene-enrichment analysis (where P = 0 represents perfect enrichment) and threshold 0.1, and only pathways with membership of at least two upregulated genes are shown. FDR = False discovery rate.

**Table 4:** KEGG pathways analysis using DAVID of genes downregulated during pregnancy.

Pathway accession	Pathway Term	Count	%	P-Value	Genes	Fold Enrichment	Benjamini-adjusted P-value	FDR
mdo04360	Axon guidance	8	2.22	4.42E-03	*SEMA5A, EPHA8, ROBO1, NTNG2, ROBO2, NFATC4, EFNA5, EPHB4*	3.8	4.55E-01	5.1
mdo04110	Cell cycle	7	1.94	1.51E-02	*CCNB1, CDC45, MAD2L1, PLK1, TTK, ORC1, MCM5*	3.5	6.47E-01	16.4
mdo04310	Wnt signaling pathway	7	1.94	2.02E-02	*SFRP2, WIF1, NFATC4, FZD2, AXIN2, DAAM2, FZD7*	3.2	6.06E-01	21.3
mdo05200	Pathways in cancer	13	3.6	2.43E-02	*PTGER3, TGFBR1, ARNT2, RUNX1T1, FZD2, CXCL12, FZD7, EDNRA, VEGFD, LAMA3, RARB, PTCH2, AXIN2*	2.0	5.69E-01	25.1
mdo04514	Cell adhesion molecules (CAMs)	7	1.94	2.63E-02	*VTCN1, CNTN2, NTNG2, ITGA4, JAM2, NEGR1, SDC3*	3.0	5.19E-01	26.9
mdo00230	Purine metabolism	8	2.22	2.90E-02	*NME4, PDE7B, POLE, PDE5A, GUCY1A3, NPR2, PDE4D, AMPD3*	2.7	4.89E-01	29.2
mdo04022	cGMP-PKG signaling pathway	7	1.94	3.88E-02	*EDNRA, GTF2IRD1, PDE5A, GUCY1A3, NPR2, NFATC4, CACNA1D*	2.8	5.39E-01	37.2
mdo04060	Cytokine-cytokine receptor interaction	8	2.22	4.13E-02	*VEGFD, TGFBR1, LEPR, TNFSF15, TNFSF13, CNTFR, TNFSF12, CXCL12*	2.5	5.15E-01	39.1
mdo04330	Notch signaling pathway	4	1.11	4.69E-02	*NOTCH3, DTX3L, MAML2, JAG1*	4.9	5.19E-01	43.1
mdo05217	Basal cell carcinoma	4	1.11	4.94E-02	*PTCH2, FZD2, AXIN2, FZD7*	4.8	5.00E-01	44.9
mdo04724	Glutamatergic synapse	5	1.39	9.61E-02	*SLC1A3, GNAO1, GLS, GRIA4, CACNA1D*	2.8	7.16E-01	69.5

P-values are modified Fisher’s Exact P-Values for gene-enrichment analysis (where P = 0 represents perfect enrichment) and threshold 0.1, and only pathways with membership of at least two upregulated genes are shown. FDR = False discovery rate.

### Comparison between *Monodelphis domestica* and *Sminthopsis crassicaudata*

**Figure 1:**
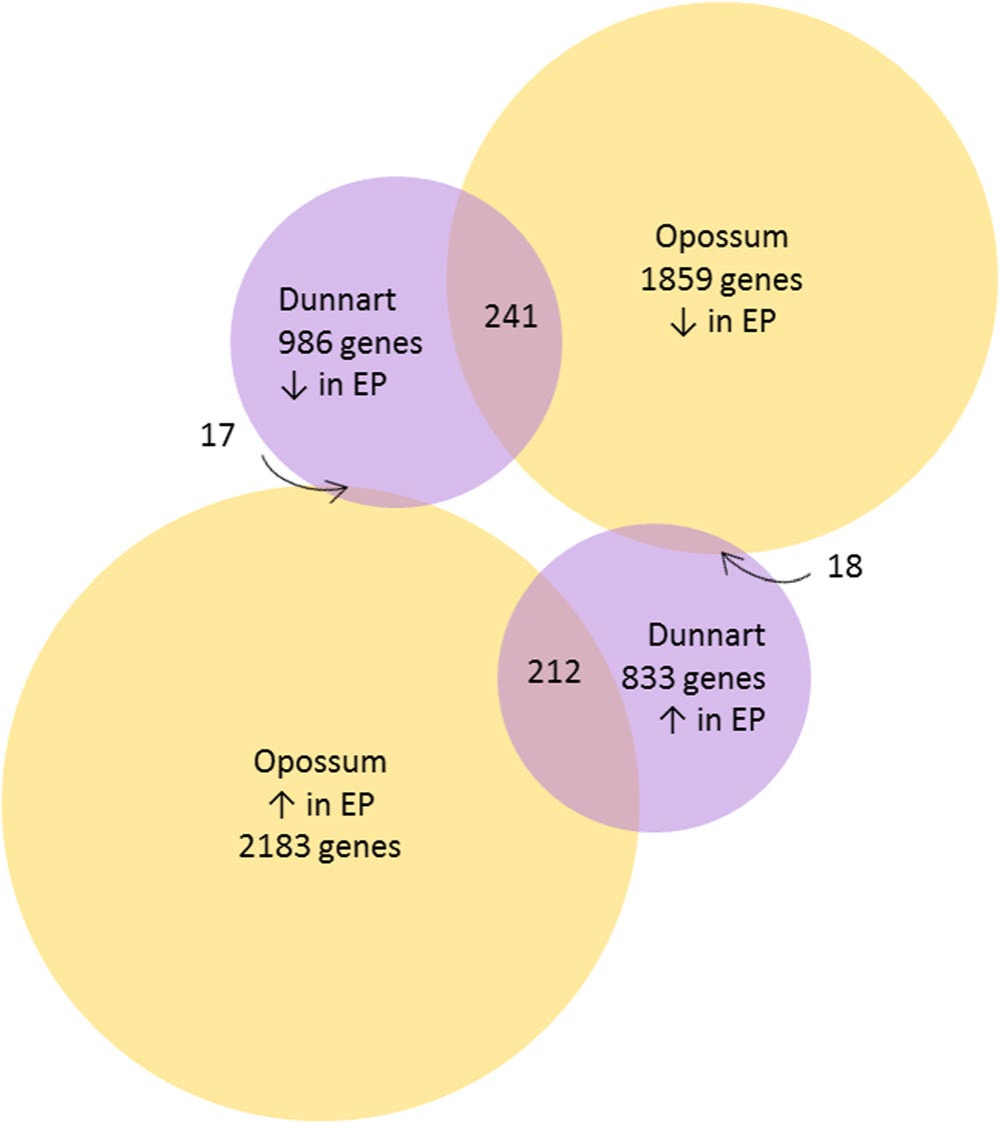
Venn diagram indicating the differentially expressed genes between opossum pre-implantation pregnant and non-pregnant uterus that are also differentially expressed in dunnart pre-implantation pregnancy. EP = early/pre-implantation pregnancy.

Ninety-seven percent of differentially expressed *Monodelphis domestica* (grey short-tailed opossum) genes^18^ between non-pregnant and pre-implantation uterus were shared in the *S. crassicaudata* uterine transcriptome. 20% of the top 50 annotated *M. domestica* pregnancy upregulated genes were upregulated in *S. crassicaudata* pregnancy, and 14% of the top 50 annotated *M. domestica* pregnancy downregulated genes were downregulated in *S. crassicaudata* pregnancy (Supplementary Tables 3 and 4). Of the *M. domestica* genes upregulated in pregnancy, 10% were upregulated in dunnart pregnancy; of the *M. domestica* genes downregulated in pregnancy, 13% were downregulated in dunnart pregnancy. Less than one percent of the differentially regulated opossum genes were differentially regulated in the opposite direction in dunnart (Fig. 1).

Gene ontology clustering analysis using DAVID^33^ indicated an overrepresentation of shared genes between dunnart and opossum that were upregulated during pregnancy, which are involved in a variety of functions, including membrane function, metabolism and biosynthesis, transport and lysosome function, cellular remodelling, motility, apoptosis and cell adhesion, and immunity (Supplementary Table 5). The same clustering analysis indicated an overrepresentation of shared genes downregulated during pregnancy that are involved in morphogenesis and development, transport, cellular motility, protein localization, focal adhesion, cytoskeletal function (laminin and focal adhesion function), and immune roles (Supplementary Table 6). KEGG pathway analysis of shared pregnancy-upregulated genes showed significant enrichment of 16 pathways involved in metabolism, protein processing and export, secretion, and lysosome function, three of which (metabolic pathways, protein export, protein processing in endoplasmic reticulum) survived Benjamini-Hochberg correction (Supplementary Table 7). In contrast, KEGG pathway analysis of downregulated genes during pregnancy showed significant enrichment of 11 pathways involved in axon function, cancer, signalling, metabolism, and receptor interaction, one of which (axon guidance) survived Benjamini-Hochberg correction (Supplementary Table 8).

## Discussion

Our transcriptomic analysis of dunnart uterus reveals differential expression of a range of genes putatively involved in the processes of early pregnancy, prior to implantation of the unshelled conceptus into the lining of the uterus. GO and pathway analyses indicate that there is significant differential regulation of groups of genes involved in metabolism and biosynthesis, and almost one third of the top 50 upregulated genes in pregnancy have these roles (Table 1), an unsurprising result that highlights the importance of these processes in the metabolically active uterus during pregnancy. Our results also point to a role for differential regulation of genes encoding nutrient transporters, cytoskeletal molecules, and immune factors in the uterus to support histotrophy, immunological protection and tissue remodelling required for early development of the embryo. Similar functions have been identified using transcriptomic studies of species representing independent origins of viviparity, indicating that these processes are critical to maintaining pregnancy across taxa^15,32,34,35^.

### Nutrient provisioning to the unimplanted embryo

In marsupials and eutherian mammals, the initial pre-attachment embryonic development is supported by histrotrophes secreted by uterine glands^36^. Following embryonic attachment, nutrient supply typically shifts to haemotrophy (i.e. secretion of material from the maternal blood circulation^4^). Haemotrophic nutrient transfer either occurs through direct embryonic contact with maternal blood, or through diffusion or active transport of haemotrophes from maternal blood, followed by secretion by the uterine epithelium into the uterine lumen^37^. In marsupials, the shift from histotrophic to haemotrophic nutrient transfer typically occurs following rupture of the embryonic shell coat^38^. In *S. crassicaudata*, this shift is accompanied by structural changes to the uterus. Early in *S. crassicaudata* pregnancy (the period at which our pregnant transcriptome samples were collected), uterine stromal glands are abundant and actively secreting^12,24^. As pregnancy progresses, gland abundance decreases and glandular secretion is replaced by secretory activity in the luminal epithelium^12^. We identified a number of genes putatively responsible for nutrient transport to the early conceptus:

#### Histotrophy

Almost one quarter of the top 50 upregulated genes in early *S. crassicaudata* pregnancy have putative transport-associated function, suggesting that nutrient transport underpins histotrophy in supporting the conceptus pre-implantation (Table 1), even before haemotrophic nutrient transport via the placenta. A number of secretion-related genes upregulated in early pregnancy may be associated with glandular secretion of histotrophe (e.g. *AP4S1*, *HYOU1*, *SRPRA*) (Table 5). Early pregnancy involves significant upregulation of nutrient transporter genes, including *APOL6*, involved in cholesterol transport^39^, *PLA2G10*, involved in hydrolysis of fatty acids during pregnancy^40^, and a suite of solute carrier proteins (*SLC*s) involved in transport of nucleoside sugars, ions and anions, glucose, fatty acids, calcium and zinc (Table 5). Upregulation of solute carrier proteins also occurs during pregnancy in the uterus of the viviparous skink *Chalcides ocellatus*^35,41^ and the post-implantation uterus of the marsupial *M. domestica*^15^. Similarly, cathepsin L (*CTSL*), upregulated during pregnancy in *C. ocellatus*^35^ and pigs^42,43^, is also significantly upregulated during pregnancy in *S. crassicaudata* (Table 5). Cathepsins are involved in remodelling of the uterine epithelium, which may enable transport of gases, macromolecules and micronutrients for embryonic development^43^. These molecules are also components of secreted uterine fluid in horses, pigs, sheep and cattle, along with phospholipases^44^. Additionally, cathepsins are present in the mouse and human yolk sac during early pregnancy, where they may degrade proteins to free amino acids for uptake by the fetus^20^, and we suggest that *CTSL* may play a similar role during early pregnancy in the dunnart uterus.

**Table 5:** Significantly up-regulated genes during pregnancy putatively involved in tissue remodelling, immune function, and transport.

Gene symbol	Gene name	Mean pregnant expression	Mean non-pregnant expression	log2 Fold Change	Adjusted P-value	Putative Function
**Tissue remodelling/cytoskeletal function**						
*AKAP9*	A-kinase anchoring protein 9	26.4	11.5	1.5	3.42E-05	Scaffolding
*CADM3*	cell adhesion molecule 3	39.1	2.3	3.9	8.19E-06	Cell-cell adhesion
*CAMSAP3*	Calmodulin Regulated Spectrin Associated Protein Family Member 3	25.9	6.6	2.4	1.92E-04	Microtubule dynamics and organisation
*CD164*	CD164 Molecule	290.5	146.5	1.4	8.12E-05	Cell adhesion
*CTSL*	Cathepsin L	268.6	95.9	1.6	8.38E-04	Proteolytic actvity/transport
EHF*	ETS Homologous Factor	179.1	14.0	3.9	1.83E-11	Epithelial cell differentiation
*FAM110C*	Family With Sequence Similarity 110 Member C	27.1	5.1	2.7	3.40E-04	Epithelial cell migration
*FGFBP1*	fibroblast growth factor binding protein 1	48.1	2.4	3.8	3.56E-06	Cellular migration
FGFR1*	fibroblast growth factor receptor 1	141.4	28.4	2.6	1.03E-10	Cell differentiation
*JPH1*	Junctophilin 1	25.0	5.6	2.4	5.29E-04	Component of junctional complexes
*KIAA1324*	KIAA1324	707.4	39.8	3.8	1.65E-04	Protection against cell death; activated by estrogen
*KMT5A*	Lysine Methyltransferase 5A	59.0	10.8	2.7	7.02E-05	Cell proliferation
*LLGL2*	LLGL2, scribble cell polarity complex component	38.5	10.3	2.2	6.98E-04	Cell migration; epithelial cell polarity
*MAP7*	Microtubule Associated Protein 7	52.8	23.8	1.5	8.20E-05	Epithelial cell differentiation
*MFSD2A*	major facilitator superfamily domain containing 2A	126.7	4.9	3.8	8.17E-04	Fatty acid transport (lysophosphatidylcholine) and placentation
*MPZL3*	Myelin Protein Zero Like 3	20.9	4.7	2.5	4.28E-05	Cell-cell adhesion
*MYO15A*	myosin XVA	13.4	0.7	4.1	3.22E-06	Actin binding
*PCDH1*	protocadherin 1	16.7	5.5	1.9	4.95E-05	Cell adhesion
*PLEKHG6*	Pleckstrin Homology And RhoGEF Domain Containing G6	16.6	3.2	2.6	1.27E-04	Cell morphology
PLA2G10*	Phospholipase A2 Group X	133.1	3.1	5.5	2.32E-20	Lipid hydrolysis
*PLXNB3*	Plexin B3	16.2	5.2	2.1	1.78E-04	Cell growth and migration
*RASSF6*	Ras Association Domain Family Member 6	39.4	6.1	3.0	1.34E-04	Apoptosis
*SPTBN2*	spectrin beta, non-erythrocytic 2	15.1	4.2	2.7	1.83E-04	Cell membrane component
*ST14*	suppression of tumorigenicity 14	45.0	17.9	1.7	1.77E-04	Protease
*TMEM102*	transmembrane protein 102	30.2	9.5	2.0	1.49E-04	Apoptosis
*TMEM79*	transmembrane protein 79	73.2	10.3	3.0	3.74E-04	Epithelial function
*TMIGD2*	Transmembrane And Immunoglobulin Domain Containing 2	7.0	1.6	2.4	3.66E-06	Cell migration and angiogenesis
*TSPAN13*	Tetraspanin 13	1233.9	194.1	2.8	3.51E-04	Signal transduction regulating cell growth
*TUSC2*	tumor suppressor candidate 2	57.4	22.9	1.7	4.15E-05	Apoptosis
ZNF750*	Zinc Finger Protein 750	2.6	0.0	6.0	9.63E-09	Transcription factor mediating cell differentiation
**Immune function**						
*BPI*	Bactericidal/Permeability-Increasing Protein	7973.0	8.2	6.3	9.98E-08	Antimicrobial (gram-negative organisms)
*BPIFB1*	BPI Fold Containing Family B Member 1	67.6	0.1	5.9	7.88E-07	Innate immune response to bacteria
*CD101*	CD101 Molecule	2.6	1.1	1.6	5.77E-04	Inhibition of T-cell proliferation; inhibition of IL2 production
*CD200R1*	CD200 Receptor 1	15.5	6.1	2.5	6.38E-06	Inhibition of inflammation
*GZMA*	Granzyme A	98.6	18.2	2.8	7.96E-06	Lysis of pathogen cells
*HDC*	Histidine Decarboxylase	102.7	0.4	5.9	4.33E-07	Histamine production
*IBTK*	inhibitor of Bruton tyrosine kinase	33.2	18.1	1.2	7.27E-04	B cell development
IDO1*	Indoleamine 2,3-Dioxygenase 1	158.9	3.5	5.2	1.14E-10	Protection of the fetus from maternal immune rejection
*IL17RA*	Interleukin 17 receptor A	52.8	20.3	1.7	3.84E-04	Binding to proinflammatory cytokines
*IL18RAP*	Interleukin 18 Receptor Accessory Protein	76.6	20.9	2.0	6.47E-04	Subunit of proinflammatory cytokine receptor
IL22RA1*	Interleukin 22 receptor subunit alpha 1	127.5	14.6	3.3	1.38E-08	Class II cytokine receptor (Class II cytokines initiate innate immune response)
*ITFG1*	Integrin Alpha FG-GAP Repeat Containing 1	69.5	38.6	1.2	3.73E-05	Modulator of T cell function
*ITGAD*	Integrin Subunit Alpha D	1.3	0.3	2.8	1.21E-04	Leukocyte activity
LY9*	Lymphocyte Antigen 9	2.6	0.1	4.5	2.89E-08	Modulation of immune cell activity (innate and adaptive)
*NKG7*	Natural Killer Cell Granule Protein 7	12.1	5.7	1.6	2.31E-04	Immunity
*PELI3*	Pellino E3 ubiquitin protein ligase family member 3	56.5	16.1	2.0	1.17E-04	Innate immune response
*PRF1*	Perforin 1	5.9	1.1	3.1	8.96E-06	Cell lysis (defense against non-self cells and virus infected cells)
SH2D1B*	SH2 Domain Containing 1B	1.9	0.2	3.3	4.35E-07	Signal transduction in immune cells
*TMEM9B*	TMEM9 Domain Family Member B	54.6	32.6	1.1	7.42E-04	Proinflammatory cytokine production
*TRAT1*	T Cell Receptor Associated Transmembrane Adaptor 1	3.3	0.5	3.0	1.94E-08	T-cell receptor stabilisation
*TRDC*	T Cell Receptor Delta Constant	8.8	1.9	2.6	8.87E-04	T-cell receptor component
*TXK*	TXK Tyrosine Kinase	2.4	0.3	3.1	5.79E-05	Regulation of adaptive immune response
*XCL2*	X-C Motif Chemokine Ligand 2	5.5	1.2	2.5	8.46E-06	Chemotaxis of lymphocytes
*ZNF683*	Zinc Finger Protein 683	11.4	2.1	2.6	2.17E-04	Transcription factor mediating immune function
**Transport**						
*ABCA3*	ATP binding cassette subfamily A member 3	52.5	6.8	3.2	3.07E-05	Transport (lipids)
*AGAP1*	ArfGAP With GTPase Domain, Ankyrin Repeat And PH Domain 1	20.7	10.6	1.4	2.43E-04	Membrane trafficking, cytoskeleton dynamics
*AP3D1*	adaptor related protein complex 3 delta 1 subunit	44.0	21.1	1.4	9.04E-05	Vesicle-mediated transport
*AP4S1*	Adaptor Related Protein Complex 4 Sigma 1 Subunit	21.5	12.4	1.2	2.04E-04	Secretory pathways
APOL6*	apolipoprotein L6	151.1	16.1	3.2	1.36E-14	Lipid movement
*ARRDC4*	Arrestin Domain Containing 4	37.6	6.3	2.9	3.35E-05	Endocytosis
*CTAGE5*	cTAGE family member 5	47.4	20.7	1.6	1.23E-04	Collagen export from the endoplasmic reticulum
*GCC2*	GRIP and coiled-coil domain containing 2	27.0	9.7	1.9	5.68E-04	Vesicle-mediated transport
*GDI2*	GDP dissociation inhibitor 2	220.6	93.8	1.5	7.02E-04	Vesicle-mediated transport
*GJB6*	Gap Junction Protein Beta 6	21.7	2.7	3.0	9.86E-04	Connexin protein that makes up hemichannels of gap junctions allowing transport between cells
GRIN1*	glutamate ionotropic receptor NMDA type subunit 1	370.7	23.2	4.1	5.76E-10	Ion channel
*HOOK2*	hook microtubule tethering protein 2	37.7	14.8	1.8	6.49E-04	Vesicle-mediated transport
*HYOU1*	hypoxia up-regulated 1	221.7	65.4	2.1	1.10E-06	Protein folding and secretion
*KCNK6*	potassium two pore domain channel subfamily K member 6	24.7	5.2	2.6	2.61E-06	Potassium ion transport
*MAL2*	mal, T-cell differentiation protein 2	60.8	13.9	2.4	2.94E-05	Transmembrane protein required for trancytosis through apical cell membrane
MFSD4A*	Major Facilitator Superfamily Domain Containing 4A	12.3	0.2	5.5	2.93E-09	Transmembrane transport
*MFSD8*	major facilitator superfamily domain containing 8	6.2	1.6	2.3	3.49E-05	Membrane protein with transporter domain (rest of the family transports small solutes, this one is unknown)
*MPC1*	mitochondrial pyruvate carrier 1	117.3	43.5	1.7	5.90E-04	Pyruvate transport into mitochondria
*MPC2*	mitochondrial pyruvate carrier 2	114.2	31.2	2.1	1.52E-04	Pyruvate transport into mitochondria
*NAGPA*	N-acetylglucosamine-1-phosphodiester alpha-N-acetylglucosaminidase	15.8	5.2	1.9	6.03E-05	Golgi transport
*NR4A3*	nuclear receptor subfamily 4 group A member 3	14.9	3.2	2.6	5.17E-07	Glucose transport, transcriptional control
*NUP210L*	nucleoporin 210 like	1.7	0.9	1.8	2.07E-04	RNA transport
*NUS1*	NUS1 dehydrodolichyl diphosphate synthase subunit	41.8	18.1	1.6	3.05E-05	Golgi transport
*RAB25*	RAB25, member RAS oncogene family	51.2	15.3	2.1	9.27E-05	Membrane trafficking
*RANBP3L*	RAN binding protein 3 like	19.2	1.1	3.9	2.38E-06	Nucleocytoplasmic transport
*SCNN1A*	sodium channel epithelial 1 alpha subunit	234.0	19.5	3.7	1.20E-05	Sodium ion transport
SEC62*	SEC62 homolog, preprotein translocation factor	223.0	39.3	2.8	3.18E-08	Protein transport through ER
*SFT2D1*	SFT2 domain containing 1	77.4	18.8	2.3	1.49E-05	Golgi transport
*SGSM2*	small G protein signaling modulator 2	12.5	4.6	1.9	8.32E-04	Regulation of membrane trafficking
*SLC16A6*	Solute carrier family 16 member 6	92.4	2.5	5.3	2.52E-07	Lactic acid/ketone
*SLC25A1*	solute carrier family 25 (mitochondrial carrier; citrate transporter), member 1	108.8	27.7	2.1	7.54E-04	Mitochondrial molecule transport
*SLC25A10*	Solute Carrier Family 25 Member 10	33.5	11.9	1.7	9.76E-05	Mitochondrial molecule transport
*SLC26A4*	solute carrier family 26 member 4	35.3	3.7	3.4	2.19E-05	Anion transport (I^−^, Cl^−^, HCO_3_^−^)
*SLC26A9*	solute carrier family 26 member 9	14.3	0.9	4.1	1.43E-06	Anion transport (Cl^−^, HCO_3_^−^)
*SLC27A2*	solute carrier family 27 member 2	180.1	1.6	5.5	1.40E-07	Fatty acid transport
*SLC28A3*	solute carrier family 28 member 3	10.2	0.4	3.6	3.85E-04	Sodium-coupled nucleoside transport;
SLC2A12*	Solute Carrier Family 2 Member 12	82.8	3.4	4.2	9.84E-12	Glucose transport
*SLC30A2*	zinc transporter 2	27.0	0.3	5.1	1.54E-06	Zinc transport
*SLC33A1*	solute carrier family 33 (acetyl-CoA transporter), member 1	193.3	30.3	2.9	1.69E-06	Acetyl-CoA transport
*SLC35A2*	solute carrier family 35 (UDP-galactose transporter), member A2	53.7	18.8	1.9	2.45E-04	Nucleoside sugar transport
*SLC35B1*	solute carrier family 35 member B1	63.7	31.9	1.4	9.57E-05	Nucleoside sugar transport
*SLC35B3*	solute carrier family 35 (adenosine 3′-phospho 5′-phosphosulfate transporter), member B3	21.3	7.2	1.9	1.87E-05	Nucleoside sugar transport
*SLC35C1*	Solute carrier family 35 member C1	63.8	7.5	3.3	2.52E-07	Nucleoside sugar transport
*SLC35D2*	solute carrier family 35 (UDP-GlcNAc/UDP-glucose transporter), member D2	155.8	8.6	4.1	2.09E-07	Nucleoside sugar transport
*SLC35F5*	solute carrier family 35, member F5	64.5	30.7	1.6	4.91E-06	Nucleoside sugar transport
*SLC35G1*	solute carrier family 35, member G1	1.8	0.8	1.6	5.36E-04	Nucleoside sugar transport
*SLC37A1*	solute carrier family 37 member 1	58.6	6.8	3.4	6.99E-07	Sugar-phosphate exchange
*SLC37A2*	solute carrier family 37 member 2	40.2	7.7	2.5	9.81E-04	Sugar-phosphate exchange
*SLC39A11*	solute carrier family 39 member 11	170.3	21.0	3.0	2.24E-04	Zinc transport
*SLC3A2*	solute carrier family 3 (amino acid transporter heavy chain), member 2	154.4	23.5	3.2	3.32E-05	Amino acid transport
*SLC46A3*	solute carrier family 46 member 3	41.9	4.3	3.3	1.45E-06	Small molecule transport
*SLC7A8*	Solute Carrier Family 7 Member 8	66.1	12.7	2.5	1.94E-05	Small and large neutral amino acid transport
*SLC9A2*	solute carrier family 9 member A2	78.1	8.0	3.5	4.30E-05	Na^+^, Li^+^, H^+^, NH_4_^+^transport; regulation of cell pH and volume
*SLC9A4*	solute carrier family 9 member A4	203.5	12.7	3.9	4.74E-07	Na^+^, H^+^, NH_4_^+^ transport; pH regulation
*SLCO4A1*	solute carrier organic anion transporter family member 4A1	37.6	3.7	3.4	3.11E-07	Bicarbonate transport
*SRPRA*	SRP receptor alpha subunit	100.8	44.7	1.5	3.44E-04	Transport of secretory and membrane proteins
STC1*	stanniocalcin 1	3962.9	39.4	6.2	7.96E-14	Calcium and phosphate transport
*TMEM165*	transmembrane protein 165	233.6	15.2	3.6	6.50E-04	Calcium/proton transport; pH homeostasis
*TRAPPC10*	trafficking protein particle complex 10	28.0	13.7	1.4	8.78E-04	Vesicle-mediated transport
*TRPM6*	transient receptor potential cation channel subfamily M member 6	1.2	0.1	3.0	9.64E-04	Magnesium transport
*TRPV6*	Transient Receptor Potential Cation Channel Subfamily V Member 6	33.6	3.3	3.2	1.57E-06	Calcium channel
*ZDHHC3*	zinc finger DHHC-type containing 3	47.6	20.4	1.6	9.73E-05	Mediation of calcium transport
**Other**						
AKR1D1*	Aldo-Keto Reductase Family 1 Member D1	190.3	0.3	7.1	1.45E-11	Steroid hormone reduction
*DHCR7*	7-Dehydrocholesterol Reductase	24.9	9.4	1.8	5.70E-04	Cholesterol biosynthesis
*ELF5*	E74 like ETS transcription factor 5	75.7	2.7	4.3	8.35E-06	Transcriptional regulation in glandular epithelium
*HSD17B7*	Hydroxysteroid 17-Beta Dehydrogenase 7	29.9	7.6	2.3	3.39E-04	Steroid biosynthesis
HSD3B7*	Hydroxy-Delta-5-Steroid Dehydrogenase, 3 Beta- And Steroid Delta-Isomerase 7	2298.1	39.5	4.9	7.01E-10	Bile synthesis from cholesterol; part of enzymatic system biosynthesising steroids
*LVRN*	Laeverin	190.7	1.0	5.1	2.43E-05	Metalloprotease which may be important for placentation
*NAGS*	N-Acetylglutamate Synthase	20.5	7.5	1.7	5.19E-04	Ureagenesis
*PAQR7*	Progestin And AdipoQ Receptor Family Member 7	126.6	11.3	3.4	5.17E-07	Progesterone binding
*PRDM2*	PR/SET Domain 2	55.3	20.9	1.8	1.41E-04	Effector of estrogen action
*SC5D*	Sterol-C5-Desaturase	294.8	13.7	4.3	1.91E-07	Cholesterol biosynthesis

The table displays HUGO Gene Symbol of the best BLAST hit, log2 ratios, and FDR‐adjusted p‐values, along with mean expression values per stage. Mean expression values are normalized transcripts per million (TPM). Only genes with adjusted P-values < 0.001 are shown. * indicates top 100 differentially expressed genes.

#### Macromolecule catabolism

Lysosomal activity is also one of the most significantly upregulated KEGG pathways during pregnancy in *S. crassicaudata* (Table 3). This result indicates that breakdown of macromolecules into small subunits for uterine secretion^41,45^ occurs during the period of receptivity in dunnarts. Such catabolism is probably required during histotrophic nutrition to provide molecules small enough for uptake through the permeable shell coat of the conceptus. Lysosomes and lysosomal-associated genes are also upregulated during pregnancy in the uterine epithelium of both pigs^46^ and viviparous skinks during pregnancy^35,41,45^, and lysosome-associated genes are abundant in the human yolk sac^20^. Increased lysosomal activity is consistent with an increased protein content of luminal fluid in the marsupial uterus pre-implantation^24,47^. Lysosomal activity is also congruent with morphological observations of dark electron-dense vesicles in uterine glandular epithelial cells, which become electron-lucent pre-implantation in *S. crassicaudata*^12,26^. This morphological pattern also occurs during pregnancy in viviparous skinks^45^ and pigs^48^. The lysosomal genes upregulated in pre-implantation *S. crassicaudata* uterus suggests that similar genetic mechanisms mediate nutrient breakdown for histotrophy in diverse viviparous groups.

#### Adenogenesis

Interestingly, both cadherins and the Wnt signaling pathway, involved in mammalian uterine adenogenesis (gland development, which is essential for histotrophy^49^), are down-regulated in the pregnant *S. crassicaudata* uterus (Tables 4, 6). This finding suggests a cessation of gland development in the uterine stroma as pregnancy progresses, which is consistent with a morphological decrease in gland density in the uterine stroma of *S. crassicaudata* during the period of uterine receptivity^12^. Hence, the shift from histotrophic nutrient transfer may begin prior to implantation to allow a rapid shift to haemotrophic nutrient provisioning upon implantation.

**Table 6:** Significantly down-regulated genes during pregnancy putatively involved in tissue remodelling, immune function, and transport.

Gene Symbol	Gene name	Mean pregnant expression	Mean non-pregnant expression	log2 Fold Change	Adjusted P-value	Putative Function
**Tissue remodelling/cytoskeletal function**						
*AATK*	Apoptosis Associated Tyrosine Kinase	0.6	2.7	−1.8	3.62E-05	Apopotosis, cell growth arrest
*ADGRA2*	adhesion G protein-coupled receptor A2	5.8	20.7	−1.4	3.15E-06	Endothelial cell sprouting
ADGRB2*	adhesion G protein-coupled receptor B2	0.1	5.2	−4.3	3.23E-12	Inhibition of angiogenesis
ADGRB2*	adhesion G protein-coupled receptor B2	3.3	27.9	−2.5	6.06E-09	Inhibition of angiogenesis
*AEBP1*	AE Binding Protein 1	8.7	96.6	−3.1	2.94E-05	Transcriptional repression in cell differentiation and growth
*AFAP1L1*	Actin Filament Associated Protein 1 Like 1	0.5	3.7	−2.4	1.50E-05	Podosome and invadosome formation
*ANGPTL1*	Angiopoietin Like 1	0.5	14.9	−3.9	1.33E-06	Vascular endothelial growth factor
*ANTXR1*	Anthrax toxin receptor 1	3.6	41.6	−2.9	5.31E-05	Cell attachment
*ANTXR1*	Anthrax toxin receptor 1	8.6	34.9	−1.6	6.41E-04	Cell attachment
*ANTXR2*	Anthrax toxin receptor 2	12.7	72.2	−1.9	2.90E-04	Extracellular matrix adhesion
*ARVCF*	Armadillo Repeat Gene Deleted In Velocardiofacial Syndrome	3.2	20.5	−2.0	6.82E-05	Adherens junction formation
*ASCL4*	Achaete-Scute Family BHLH Transcription Factor 4	1.2	7.1	−3.1	3.86E-04	Transcription factor involved in cell differentiation
*BOC*	BOC cell adhesion associated, oncogene regulated	3.6	14.3	−2.0	2.69E-06	Cell-cell interactions
C14orf180*	Chromosome 14 Open Reading Frame 180	3.2	17.9	−2.2	3.06E-10	Plasma membrane component
*C14orf37*	Chromosome 14 Open Reading Frame 37	0.3	3.3	−2.2	1.36E-04	Membrane component
*CCDC114*	Coiled-Coil Domain Containing 114	0.7	7.5	−2.6	3.61E-04	Cilial cell function
CDC42EP3*	CDC42 Effector Protein 3	2.6	21.7	−2.6	8.30E-10	Actin cytoskeleton reorganisation
*CDH11/CDH19*	Cadherin 11/Cadherin 19	8.2	54.1	−2.2	1.40E-04	Cell-cell adhesion
*CDH20*	cadherin 20	0.3	6.2	−3.6	2.91E-06	Cell-cell adhesion
*CDHR3*	cadherin related family member 3	0.1	1.6	−3.4	1.37E-04	Cell-cell adhesion
*CEMIP*	cell migration inducing hyaluronan binding protein	2.7	34.2	−2.8	1.80E-05	Hyaluronic acid binding
*CLMP*	CXADR Like Membrane Protein	3.3	18.4	−2.0	6.38E-06	Cell-cell adhesion
*CNKSR2*	Connector Enhancer Of Kinase Suppressor Of Ras 2	0.3	4.6	−3.2	6.09E-06	Signal transduction for cytoskeleton remodelling
CNTN2*	contactin 2	0.0	3.0	−5.7	2.23E-13	Cell adhesion
*COL15A1*	collagen type XV alpha 1 chain	1.3	32.7	−3.8	1.40E-07	Connection of basement membrane to underlying tissues
COL7A1*	collagen type VII alpha 1 chain	0.1	2.6	−4.8	2.66E-18	Anchoring of basement membrane
COL7A1*	collagen type VII alpha 1 chain	0.1	5.7	−5.1	4.44E-10	Anchoring of basement membrane
*CORO6*	Coronin 6	0.1	1.4	−3.3	5.32E-04	Actin binding
*DDIAS*	DNA Damage Induced Apoptosis Suppressor	0.6	3.1	−1.9	3.87E-04	Anti-apoptosis activity
*DST*	Dystonin	2.6	16.0	−1.9	8.49E-06	Cytoskeletal linkages
*DZIP1*	DAZ Interacting Zinc Finger Protein 1	2.2	7.0	−2.0	1.46E-05	Cilium formation
*EFNA5*	ephrin A5	1.5	8.1	−2.4	2.49E-05	Migration and adhesion
*EMILIN1*	elastin microfibril interfacer 1	9.8	93.6	−2.6	6.09E-05	Extracellular matrix glycoprotein
*EPB41L2*	Erythrocyte Membrane Protein Band 4.1 Like 2	12.7	42.7	−1.3	1.44E-04	Cytoskeletal function
*EPHB4*	EPH receptor B4	4.5	20.0	−1.7	2.17E-05	Vascular development
*ERVMER34-1*	Endogenous Retrovirus Group MER34 Member 1	4.8	24.2	−2.1	8.01E-07	May have membrane fusion activity
*FAP*	fibroblast activation protein alpha	3.5	22.3	−1.9	1.67E-05	Tissue remodelling
*FAT4*	FAT atypical cadherin 4	0.5	3.0	−2.1	2.04E-04	Cell polarity
*FBLN7*	Fibulin 7	0.2	2.5	−3.0	3.71E-04	Cell adhesion
*FLRT2*	fibronectin leucine rich transmembrane protein 2	2.0	12.4	−2.2	5.16E-07	Cell adhesion
*FLRT3*	fibronectin leucine rich transmembrane protein 3	1.0	7.7	−2.3	9.63E-04	Cell-cell adhesion and migration
FREM2*	FRAS1 related extracellular matrix protein 2	0.2	1.9	−3.0	2.85E-09	Basement membrane component; epidermal adhesion
*FREM2*	FRAS1 related extracellular matrix protein 2	0.1	0.9	−2.9	1.08E-04	Basement membrane component; epidermal adhesion
*GPC6*	Glypican 6	2.2	16.0	−2.3	4.49E-04	Cell growth and division
*IFT140*	Intraflagellar Transport 140	1.7	8.4	−1.8	3.06E-04	Ciliogenesis
*IGDCC3*	immunoglobulin superfamily DCC subclass member 3	0.3	3.5	−3.0	6.73E-08	Plasma membrane component
*IGFBP5*	insulin like growth factor binding protein 5	5.9	50.8	−2.6	2.20E-05	Cell growth and apoptosis
*ISM1*	Isthmin 1	0.9	6.4	−2.3	2.43E-05	Inhibition of angiogenesis
*ITGA4*	integrin subunit alpha 4	1.2	11.8	−2.7	3.65E-05	Cell migration
*JAM2*	Junctional Adhesion Molecule 2	4.2	29.5	−2.3	1.63E-04	Membrane protein localised to tight junctions
*KANK1*	KN Motif And Ankyrin Repeat Domains 1	5.1	39.0	−2.2	4.70E-05	Cytoskeleton organisation
*KANK4*	KN Motif And Ankyrin Repeat Domains 4	1.0	8.3	−2.5	8.39E-05	Cytoskeleton organisation
*KIF12*	kinesin family member 12	0.1	7.4	−5.4	4.33E-07	Cytoskeleton
KIF26B*	kinesin family member 26B	0.5	10.0	−3.8	4.42E-10	Cytoskeleton
KIF7*	Kinesin Family Member 7	0.4	3.8	−2.7	1.45E-09	Signalling; cilia-associated
KRT77*	Keratin 77	0.1	9.6	−5.3	7.34E-11	Epithelial cell structure
*LAMA3*	Laminin Subunit Alpha 3	1.5	11.1	−2.5	2.26E-04	Basement membrane function
*LRRC49*	Leucine Rich Repeat Containing 49	0.8	5.3	−2.3	6.26E-08	Cytoskeleton
*LTBP1*	latent transforming growth factor beta binding protein 1	8.0	70.9	−2.5	7.96E-06	Extracellular matrix
*MMP16*	matrix metallopeptidase 16	0.6	8.8	−3.2	2.85E-08	Extracellular matrix breakdown
*MPP3*	Membrane Palmitoylated Protein 3	0.2	1.1	−3.2	8.32E-04	Regulation of cell proliferation and cytoskeleton
MUC5AC*	Mucin 5AC, Oligomeric Mucus/Gel-Forming	0.1	58.6	−8.3	4.57E-38	Extracellular matrix
*MYOCD*	myocardin	0.6	4.6	−2.5	7.07E-04	Smooth muscle differentiation
*NDNF*	neuron derived neurotrophic factor	2.1	36.6	−3.5	2.70E-08	Endothelial cell survival
*NEGR1*	neuronal growth regulator 1	0.9	5.9	−2.2	6.83E-04	Cell adhesion
*OLFM4*	Olfactomedin 4	0.3	49.2	−5.0	1.32E-05	Cell adhesion, apoptosis
*PCDH18*	protocadherin 18	1.5	8.5	−2.6	1.01E-04	Cell adhesion
*PCDH7*	protocadherin 7	0.4	2.7	−2.3	5.78E-04	Cell adhesion
*PCDHA13/PCDHA3/PCDHA8/PCDHAC2*	Protocadherin Alpha 13/3/8/AC2	1.3	8.2	−2.4	1.11E-05	Cell adhesion
*PCDHB2*	Protocadherin Beta 2/Protocadherin Beta 5/8	1.2	6.2	−1.9	3.38E-05	Cell adhesion
*PCDHB5/PCDHB8*	Protocadherin Beta 5/8	2.0	10.0	−2.0	1.65E-04	Cell adhesion
*PCDHGA9/B6/B7*	Protocadherin Gamma Subfamily A, 9/B, 6/ B,7	12.9	78.5	−2.0	3.66E-04	Cell adhesion
*PDE1C*	Phosphodiesterase 1C	0.5	3.4	−2.1	6.98E-05	Regulation of proliferation of smooth muscle
*PDZRN3*	PDZ Domain Containing Ring Finger 3	2.3	12.9	−2.0	3.09E-05	Vascular morphogenesis
*PHACTR3*	Phosphatase And Actin Regulator 3	0.2	3.4	−3.2	3.42E-04	Actin regulation
*PKNOX2*	PBX/Knotted 1 Homeobox 2	0.4	3.0	−2.4	1.74E-06	Regulation of cell proliferation
*PLCD3*	Phospholipase C Delta 3	1.1	10.2	−2.5	5.91E-05	Placental development
*PPP1R26*	Protein Phosphatase 1 Regulatory Subunit 26	0.9	4.8	−1.9	2.62E-05	Regulation of cell proliferation
*PRKD3*	Protein Kinase D3	2.8	16.7	−2.1	9.98E-08	Signalling regulating cell proliferation
*PTK7*	protein tyrosine kinase 7 (inactive)	7.5	40.4	−2.0	1.47E-07	Signal transduction for cell reorganisation
*RHOJ*	Ras Homolog Family Member J	2.8	9.6	−1.3	7.09E-04	Regulation of angiogenesis
ROBO1*	Roundabout Guidance Receptor 1	1.8	19.6	−2.7	2.13E-10	Mediation of cellular migration
*RPS6KA2*	ribosomal protein S6 kinase A2	0.5	1.9	−1.7	1.88E-04	Cell growth and differentiation
*SDC3*	syndecan 3	6.4	48.4	−2.3	3.84E-07	Organisation of cytoskeleton
*SGCB*	Sarcoglycan Beta	9.7	38.9	−1.6	9.28E-05	Cytoskeleton organisation
*SGCE*	Sarcoglycan Epsilon	5.6	39.2	−2.2	7.44E-04	Cytoskeleton organisation
SHF *	Src Homology 2 Domain Containing F	0.9	9.4	−2.9	1.23E-12	Regulation of apoptosis
SMOC2*	SPARC related modular calcium binding 2	43.5	491.6	−3.0	6.85E-09	Cell matrix; cell proliferation; angiogenesis
*SPEG*	SPEG Complex Locus	0.4	3.2	−2.3	1.43E-04	Development of myocyte cytoskeleton
*SPEG*	SPEG Complex Locus	1.1	9.9	−2.6	1.92E-04	Development of myocyte cytoskeleton
*STX2*	Syntaxin 2	3.2	14.1	−1.8	1.03E-06	Epithelial morphogenesis
TCTN3*	Tectonic Family Member 3	3.0	16.6	−2.0	1.26E-08	Ciliogenesis
*TGFBR1*	transforming growth factor beta receptor 1	11.6	43.9	−1.5	2.92E-04	Regulation of cell growth
*TNFSF12*	Tumor Necrosis Factor Superfamily Member 12	3.3	17.1	−1.9	3.31E-04	Apopotosis
*TNFSF15*	Tumor Necrosis Factor Superfamily Member 15	1.1	18.0	−3.2	2.54E-05	Apopotosis
*TNMD*	tenomodulin	0.1	4.0	−3.6	5.89E-04	Angiogenesis inhibitor
*TSPAN11*	tetraspanin 11	3.1	24.7	−2.4	2.56E-06	Plasma membrane component
*TSPAN7*	tetraspanin 7	5.9	25.1	−1.7	1.03E-04	Signal transduction for cell development
*VEGFD*	vascular endothelial growth factor D	0.0	1.9	−4.8	1.26E-06	Angiogenesis
*VIT*	vitrin	0.5	7.1	−3.2	5.67E-06	Extracellular matrix
*WTIP*	Wilms tumor 1 interacting protein	4.8	22.7	−1.9	6.54E-04	Cytoskeleton organisation
*ZEB2*		5.2	22.9	−1.5	7.41E-05	Represses transcription of E-cadherin
*ZNF3*	Zinc Finger Protein 3	1.1	6.4	−2.0	2.97E-04	Cell differentiation and proliferation
*ZNF3*	Zinc Finger Protein 3	0.1	2.3	−3.5	3.27E-04	Cell differentiation and proliferation
*ZNF3*	Zinc Finger Protein 3	0.3	3.7	−2.9	4.19E-04	Cell differentiation and proliferation
**Immune function**						
CD200*	CD200 Molecule	10.8	152.6	−3.3	7.12E-12	Immunosuppression, T-cell proliferation
*CD300A*	CD300a Molecule	2.4	11.3	−1.8	2.45E-06	Inhibition of immune response
*CD5*	CD5 molecule	0.4	3.0	−2.5	6.78E-05	T cell regulation
CNTFR*	ciliary neurotrophic factor receptor	1.4	26.2	−3.4	2.94E-10	Interleukin signalling
*CXCL12*	C-X-C motif chemokine ligand 12	2.0	18.7	−2.7	7.33E-08	Immune cell chemoattractant
IFIT5*	Interferon Induced Protein With Tetratricopeptide Repeats 5	1.9	16.1	−2.6	1.27E-08	RNA binding to viral RNAs
IGHA1*	Immunoglobulin Heavy Constant Alpha 1	17.0	1722.2	−5.3	2.01E-08	Major immunoglobulin, infection defence, detecting foreign antigens
*IGHV3-15*	Immunoglobulin Heavy Variable 3-15	1.3	58.5	−4.5	8.59E-07	Antigen recognition
*IGHV3-21*	Immunoglobulin Heavy Variable 3-21	5.8	364.9	−4.5	4.99E-05	Antigen recognition
*IGHV3-23*	Immunoglobulin Heavy Variable 3-23	0.0	57.5	−6.2	3.80E-08	Antigen recognition
*IGHV3-23*	Immunoglobulin Heavy Variable 3-23	1.0	70.2	−5.2	1.46E-07	Antigen recognition
*IGHV3-23*	Immunoglobulin Heavy Variable 3-23	1.1	80.5	−4.7	1.20E-06	Antigen recognition
*IGHV3-23*	Immunoglobulin Heavy Variable 3-23	0.5	22.7	−4.3	1.09E-04	Antigen recognition
IGHV3-23*	Immunoglobulin Heavy Variable 3-23	0.4	40.9	−4.8	4.59E-06	Antigen recognition
IGHV3-74*	Immunoglobulin Heavy Variable 3-74	0.9	54.0	−4.9	8.50E-09	Antigen recognition
IGHV4-28*	Immunoglobulin Heavy Variable 4-28	0.7	99.0	−6.2	3.13E-15	Antigen recognition
*IGKV1-8*	Immunoglobulin Kappa Variable 1-8	0.9	40.6	−3.9	8.94E-04	Antigen recognition
IGKV1D-43*	Immunoglobulin Kappa Variable 1D-43	0.7	181.3	−6.3	2.07E-10	Antigen recognition
*IGKV2-24*	Immunoglobulin Kappa Variable 2-24	1.0	267.1	−5.1	1.69E-05	Antigen recognition
*IGKV2D-29*	Immunoglobulin Kappa Variable 2D-29	0.7	235.3	−5.2	1.20E-05	Antigen recognition
*IGKV2D-30*	Immunoglobulin Kappa Variable 2D-30	0.2	104.2	−5.3	6.44E-06	Antigen recognition
*IGKV3-11*	Immunoglobulin Kappa Variable 3-11	0.2	69.6	−5.1	2.33E-05	Antigen recognition
*IGKV3-11*	Immunoglobulin Kappa Variable 3-11	0.2	17.0	−4.1	5.39E-04	Antigen recognition
IGKV3D-11*	Immunoglobulin Kappa Variable 3D-11	0.0	38.0	−6.5	2.79E-09	Antigen recognition
*IGKV4-1*	Immunoglobulin Kappa Variable 4-1	0.3	114.5	−5.5	2.12E-06	Antigen recognition
*IGLC1*	Immunoglobulin Lambda Constant 1	6.7	908.7	−5.2	1.32E-06	Antigen recognition
*IGLC6*	Immunoglobulin Lambda Constant 6 (Gene/Pseudogene)	0.2	23.6	−4.3	4.31E-04	Antigen recognition
IGLV1-51*	Immunoglobulin Lambda Variable 1-51	0.0	82.6	−6.4	1.08E-08	Antigen recognition
*IGLV4-3*	Immunoglobulin Lambda Variable 4-3	1.0	58.5	−4.4	4.43E-05	Antigen recognition
*IGLV4-69*	Immunoglobulin Lambda Variable 4-69	0.0	49.6	−6.0	1.31E-07	Antigen recognition
*IGLV7-46*	Immunoglobulin Lambda Variable 7-46 (Gene/Pseudogene)	2.3	104.9	−4.0	8.01E-04	Antigen recognition
*IL34*	interleukin 34	1.6	10.9	−2.3	8.14E-06	Cytokine; promotion of inflammation
JCHAIN*	Joining Chain Of Multimeric IgA And IgM	4.6	456.8	−5.3	5.05E-09	Antigen recognition
*LCN2*	Lipocalin 2	10.7	107.8	−2.5	9.36E-04	Innate immunity
*NFATC4*	nuclear factor of activated T-cells 4	1.3	10.9	−2.4	9.56E-04	Expression of cytokines in T cells
*NLRP12*	NLR family pyrin domain containing 12	1.5	7.8	−1.9	9.54E-05	Inflammation
*RIPK2*	Receptor Interacting Serine/Threonine Kinase 2	1.8	5.8	−1.6	4.49E-04	Signalling in immune pathways
*VTCN1*	V-set domain containing T cell activation inhibitor 1	0.4	36.2	−4.9	3.48E-07	Negative regulator of T cell activation and proliferation
**Transport**						
*ABCA7*	ATP Binding Cassette Subfamily A Member 7	0.1	0.9	−2.8	9.89E-04	Transporter activity
*ANO4*	Anoctamin 4	3.9	73.5	−3.3	2.49E-06	Ion channel transport
*ATP2B4*	ATPase plasma membrane Ca2+ transporting 4	6.7	35.4	−1.9	4.75E-05	Calcium transport
*CACNA1D*	calcium voltage-gated channel subunit alpha1 D	0.5	2.9	−2.1	2.12E-06	Calcium channel
*CACNA1D*	calcium voltage-gated channel subunit alpha1 D	0.6	4.9	−2.3	4.75E-05	Calcium channel
*CACNA2D1*	Calcium Voltage-Gated Channel Auxiliary Subunit Alpha2delta 1	2.1	13.6	−2.2	2.07E-04	Calcium channel
*KCNC1*	Potassium Voltage-Gated Channel Subfamily C Member 1	4.2	38.7	−2.7	1.22E-06	Ion channel transport
*KCNH2*	potassium voltage-gated channel subfamily H member 2	0.7	5.5	−2.6	7.78E-08	Ion channel transport
KIF26B*	kinesin family member 26B	0.5	10.0	−3.8	4.42E-10	Vesicle-mediated transport
*SCN2A*	sodium voltage-gated channel alpha subunit 2	0.6	1.4	−2.2	5.97E-04	Sodium channel
*SLC1A3*	solute carrier family 1 member 3	0.8	3.2	−1.5	8.60E-04	Neutral amino acid transport
*SLC22A1*	solute carrier family 22 member 1	0.2	3.9	−3.9	4.00E-07	Cation transport
*SLC27A3*	Solute Carrier Family 27 Member 3	3.0	27.0	−2.6	2.86E-06	Fatty acid transport family but no fatty acid transport activity
*SLC41A3*	solute carrier family 41, member 3	2.1	14.1	−2.3	1.34E-05	Cation transport
*SLC4A5*	solute carrier family 4 (sodium bicarbonate cotransporter), member 5	0.2	1.9	−3.1	2.25E-04	Sodium bicarbonate transport
*SLC9A9*	solute carrier family 9, subfamily A (NHE9, cation proton antiporter 9), member 9	0.6	3.0	−1.9	2.45E-05	Sodium and potassium ion/proton exchanger
SLCO2A1*	solute carrier organic anion transporter family member 2A1	2.2	32.2	−3.4	4.70E-13	Prostaglandin release
*TRPC3*	transient receptor potential cation channel subfamily C member 3	0.1	3.9	−4.0	3.84E-06	Cation channel
**Other**						
CBX2*	Chromobox 2	1.5	13.6	−2.8	1.35E-15	Transcriptional repression
EDN3*	endothelin 3	0.0	11.4	−6.4	7.19E-10	Vasoconstriction
*EDNRA*	endothelin receptor type A	9.1	114.9	−3.0	1.47E-06	Vasoconstriction
HOXA10	Homeobox A10	5.4	39.2	−2.4	1.45E-07	Uterine receptivity
HOXA11	Homeobox A11	7.5	39.8	−1.9	6.53E-05	Uterine receptivity
*IGF2*	Insulin like growth factor 2	2.3	9.8	−3.3	3.60E-05	Growth and development; imprinted gene
*LGR6*	leucine rich repeat containing G protein-coupled receptor 6	0.0	3.3	−5.3	5.40E-07	Glycoprotein hormone receptor
*PDE5A*	Phosphodiesterase 5A	2.0	11.2	−2.0	1.72E-04	Smooth muscle function in vascular system
PTGER3*	Prostaglandin E Receptor 3	1.6	11.3	−2.6	6.98E-10	Receptor for prostaglandin E2; uterine contraction
PTGFR*	Prostaglandin F Receptor	0.1	7.5	−5.2	1.63E-12	Receptor for prostaglandin F2-alpha; uterine contraction
*SOX4*	SRY-box 4	6.1	41.3	−2.2	4.93E-04	Transcriptional control

The table displays HUGO Gene Symbol of the best BLAST hit, log2 ratios, and FDR‐adjusted p‐values, along with mean expression values per stage. Mean expression values are normalized transcripts per million (TPM). Only genes with adjusted P-values <0.001 are shown. * indicates top 100 differentially expressed genes.

### Steroid biosynthesis

The steroid biosynthesis pathway is also significantly enriched in the list of upregulated genes during pregnancy (Table 3). *CYP27A1* (sterol 27-hydroxylase P450) is involved in the conversion of cholesterol to its primary metabolite 27-hydroxycholesterol, after which 27-hydroxycholesterol is converted to bile salt precursors by *HSD3B7* (3-beta-hydroxysteroid dehydrogenase-7); the conversion of the 5-beta-reduction of bile acid intermediates and steroid hormones carrying a delta (4)-3-one structure is effected by *AKR1D1* (aldo-keto reductase family 1 member D1)^50^. All four of these genes are significantly upregulated during pregnancy, especially *AKR1DA* and *HSD3B7*, which are in the top 50 differentially expressed annotated genes (Table 5). While deficiencies in this pathway cause adrenal dysfunction and bile acid reduction^51^, the reasons for their upregulation here is less clear. 27-hydroxycholesterol is a selective modulator of the estrogen receptors^52^, and bile acid intermediates are also nutrient signalling molecules^53^; both functions may be important in the pre-implantation uterus. Linked with this pathway is the upregulation of steroid biosynthesis pathways (Table 5). The production of 7-dehydrocholestrol is followed by a sequence of gene expressions culminating in the expression of 17-beta hydroxysteroid 7 (*HSD17B7*), which is involved in the conversion of steroid precursors to androgens^51^. The upregulation of these pathways may be linked to steroid recruitment mechanisms, but may also be important in other functions during pregnancy, including the transport and utilisation of fatty acids and electrolytes in the pre-attachment phase.

### Immunity

The top five most significantly enriched GO categories in pregnancy downregulated genes are related to immune function (Supplementary Table 2), and 18% of the top 50 downregulated genes during pregnancy have putative immune function (Table 2). Many of these downregulated genes are immunoglobulins that make up subunits of antibodies (Table 6), which may simply reflect a lower relative number of B cells in pregnant uterine tissue. Other genes involved in maternal-fetal tolerance are also downregulated, including *IL34*^54^. This result reflects an important role of the uterus in immunosuppression to prevent maternal rejection of the semi-foreign embryo, even before the invasion of the embryo into the uterine epithelium. The dunnart embryonic shell membrane disintegrates prior to implantation, which in combination with remodelling may place maternal and embryonic tissues in close association^3,10^. The apposition of maternal and fetal tissues has likely driven the evolution of adaptations to ‘hide’ the embryo from the mother’s immune system, despite a lack of tissue invasion at that point in pregnancy. A similar downregulation of some immune genes occurs in the uteri of other vertebrates that lack erosion of maternal epithelia throughout pregnancy e.g.^32,35,55^.

In *S. crassicaudata*, we also observe a large proportion of immune genes upregulated pre-implantation (14% of the top 50, Table 1). In contrast to other marsupial studies, we did not see a change in interleukin-6 gene expression^15,18^, even though interleukin-6 is expressed in other tissues in *S. crassicaudata*^56^. The differences may be because our study focussed on preimplantation pregnancy. In *M. domestica*, immune genes are upregulated at implantation, including a range of inflammatory and wound-healing markers^18^. There is increasing recognition of the importance of the presence of maternal immune factors in the eutherian uterus for embryo implantation and uterine remodelling; the maternal immune response must be precisely regulated for successful mammalian pregnancy^57,58^. Our results allow comparison of both major lineages of marsupials, Australididelphia (*S. crassicaudata*, here) and Didelphimorphia (*M. domestica*^15,18^), and suggest that a delicate balance of up- and down-regulated immune factors was a feature of the pregnant uterus of the most recent common ancestor of therian mammals, exapted for the evolution of viviparity in this lineage. Immune genes of stable expression in *M. domestica*^18^ across pregnancy display the same pattern in *S. crassicaudata* (*CD3D, CD3D, CD3G, CD4, CD68, CD8B, IL4R*). Further examination of gene expression at late stage pregnancy in *S. crassicaudata* is necessary to draw conclusions about the precise immunogenic changes that facilitate implantation and placentation in the dunnart, and whether these mirror the changes seen in the Didelphimorphia. Finally, immune factors prevent pathogenic infection in vertebrate gestational tissues^32,57^, and our dataset identifies several candidate genes responsible for immune defence in the pregnant dunnart uterus (*BPI*, *BPIFB1*, *GZMA* and *PRF1*) (Table 5).

### Remodelling of the pregnant uterus

Differentially regulated *S. crassicaudata* genes are significantly enriched for a number of GO categories related to tissue proliferation, tissue remodelling, and cell membrane components (Supplementary Table 1). The cell adhesion molecule pathway is significantly downregulated as identified by KEGG pathway analysis (Table 4), and more than one third of the top 50 downregulated genes have putative functions associated with cytoskeleton and remodelling (Table 2). Alterations to both cell adhesion and remodelling are expected during the period of receptivity in preparation for implantation, and embryonic implantation in *S. crassicaudata* involves significant morphological and molecular remodelling^12,24,26^. Our findings demonstrate that, as for eutherian mammals^42,59^ and viviparous skinks^35,41,60^, remodelling involves expression changes of cathepsins (*CTSL*), cadherins (e.g. *CDH11*, *CDH20*), and numerous protocadherins (Tables 5 and 6).

Similar expression patterns of remodelling genes across diverse viviparous groups suggest a common suite of molecules is required in preparing the uterus for implantation in live-bearing taxa^60^. Down-regulation of cell adhesion molecules occurs in *S. crassicaudata*, including *JAM2*, which is associated with tight junctions^61,62^. Embryonic attachment in *S. crassicaudata* is invasive, yet unlike many eutherian mammal species with invasive placentation, the invasion involves embryonic erosion of an originally intact uterine epithelium, rather than a loss of cellular adhesion to facilitate invasion^12,24^. In viviparous skinks, reduced lateral cell adhesion makes the uterus more plastic and likely facilitates remodelling^63^. Down-regulation of the cell adhesion pathway may play a similar role in preparing the *S. crassicaudata* uterus for implantation of the embryo.

Several genes that function in angiogenesis and vascular morphogenesis are downregulated in the *S. crassicaudata* uterus during pregnancy (e.g. *ADGRA2, ADGRB2, ANGPTL1, EPHB4, ISM1, PDZRN3, RHOJ, TNMD, VEGFD*; Table 6). This result was unexpected, given the upregulation of angiogenic genes such as *EPAS1*, *HIF1A* and *VEGFA* during pregnancy in skinks and rats e.g.^35,64–66^; however several of these genes are inhibitors, rather than promoters, of angiogenesis e.g. *ISM1*^67^. Their downregulation in *S. crassicaudata* uterus during pregnancy may simply reflect temporality of our sampling: the transcriptome comes from uteri prior to the development of extensive vascularisation during placental formation, and it is possible that embryos do not require much oxygen at this early developmental stage.

Extracellular matrix molecules are down-regulated during early pregnancy in *S. crassicaudata*, including laminin (*LAMA3*), collagens (*COL7A1*, *COL15A1*), fibulin (*FBLN7*), fibronectins (*FLRT2*, *FLRT3*) and receptors (*ITGA4*), keratins (*KRT22*), and elastins (*EMILIN1*) (Table 6). We suggest that uterine receptivity in *S. crassicaudata* involves significant remodelling of the extracellular matrix. Increased expression of laminins^68–70^, fibronectin^71^ and fibronectin receptor *ITGA4*^72^ is associated with uterine receptivity in eutherian mammals. The opposite trend for these molecules in *S. crassicaudata* is unexpected, yet could be explained by differences in alterations to the uterine stroma in marsupial and eutherian pregnancy. In eutherian mammals, increased expression of extracellular matrix molecules is related to cellular differentiation of uterine stromal fibroblasts to decidual cells (decidualisation)^73,74^. This cellular transformation does not occur in *S. crassicaudata*, as marsupials lack decidual cells^73^. In addition, the uterine stroma of *S. crassicaudata* and other marsupials is relatively cell-poor, and uterine receptivity involves a significant reduction in stromal cell abundance^12,27^. Thus, the specific markers of uterine receptivity may differ between viviparous amniotes, as they relate to species-specific uterine cellular processes. Additionally, reduction in extracellular matrix leading up to implantation may help to reduce the diffusion distance between maternal blood vessels and the uterine epithelium. In marsupials, reduction of this diffusion distance is a critical step in preparation for haemotrophic nutrient transfer^37^.

### Uterine receptivity and quiescence

A number of genes differentially expressed in the dunnart uterus are similar to mediators of uterine receptivity in humans. Estrogen and progesterone are the key hormones controlling receptivity of the uterus to an implanting embryo^22^, and our data reveal differential expression of genes binding to and effecting action of these hormones (*PAQR7*; *PRDM2*) in the dunnart uterus just prior to implantation (Table 5). These hormones coordinate morphological and physiological changes in the uterus to promote receptivity, and a number of potential markers of uterine receptivity in eutherians^22^ are differentially expressed in the *S. crassicaudata* uterus. Mucins, which are apically located glycoproteins in the epithelium of the uterus, have anti-adhesive properties, and must be removed from the site of attachment before implantation can take place; dysregulation of mucin expression affects eutherian fertility^22,75,76^. A similar situation is present in marsupials, given that the mucin *MUC5AC* is the most highly downregulated gene in pre-implantation dunnart pregnancy (Table 2), and that *MUC1* increases in the grey opossum uterus after breach of the shell coat^18^. Mucins are also downregulated in the uterus during pregnancy in a viviparous skink^34^. A number of other genes involved in uterine receptivity in humans and mice are also differentially expressed in the dunnart pre-implantation uterus, including the homeobox genes *HOXA10* and *HOXA11*, and phospholipases (*PLA2G10*, *PLA2G3*)^22,77^.

Maintaining quiescence of the uterus (i.e. preventing uterine contraction) is another key requirement for progress of a successful pregnancy. Two of the most significantly downregulated genes in the pregnant dunnart uterus are the prostaglandin receptors *PTGER3* and *PTGFR* (Table 2). The products of these genes likely bind prostaglandins to stimulate myometrial contractions^78^.

### Similarities in early pregnancy between Australididelphia and Didelphimorphia

We identified 97% of the genes that were differentially expressed between non-pregnant and pre-implantation *M. domestica* uterus^18^ in the *S. crassicaudata* uterine transcriptome. This result indicates a substantial overlap in the range of expressed genes between the two species, as expected given that these species derive from a single origin of viviparity. There are many shared genes that are differentially expressed in *M. domestica* and *S. crassicaudata* (at the same stages of pregnancy: non-pregnant uterus compared to pre-implantation uterus) (Supplementary Tables 3 and 4). The overlap indicates that many of the uterine functions identified in *S. crassicaudata* are shared across both major marsupial lineages. For example, remodelling of the uterus is a shared characteristic, with genes involved in extracellular matrix (e.g. cadherin-related genes *FAT4, CDH11, CDH19* and *PCDH11X* down in pregnancy; laminin-related genes *EGFLAM, COL15A1* down in pregnancy), cellular motility (e.g. *FGF1, NRG1, SEMA5B* down in pregnancy; *RAB25, FGFR1, HBEGF* up in pregnancy) and cell adhesion (e.g. *ITGA4, PTK7, TRIP6* up in pregnancy) differentially regulated in both *S. crassicaudata* and *M. domestica*. Histotrophic function is also shared across early pregnancy in marsupials: genes involved in lysosomal transport are upregulated in pregnancy in both *M. domestica* and *S. crassicaudata* (e.g. *ATP6V1B2, AP3D1, TMEM165, TMEM79*), and pathway analysis indicates an overrepresentation of pregnancy-upregulated genes of protein processing and export, secretion, and lysosome function in the shared gene lists between the two species (Supplementary Table 7).

Of the top 50 genes of *M. domestica* that are upregulated during pregnancy, 20% are also upregulated in *S. crassicaudata* early pregnancy. These genes include *ELF5* (*ESE2*), an epithelium-specific transcription factor thought to regulate gene expression in glandular epithelium^79^ and which we postulate may be important in supporting gene expression for glandular secretions; *CTAGE5*, involved in exporting collagen from the endoplasmic reticulum^80^, and therefore possibly important for remodelling of the extracellular matrix; *FGFBP1*, which mediates cellular proliferation and migration^81^; and *LVRN*, which in humans is a trophoblast-specific factor^82^ that may regulate molecules at the interface of maternal and embryonic tissue to facilitate the development of a placenta^83^. The expression of *LVRN* in uterine tissues during early pregnancy in both major marsupial lineages suggests that this molecule may also be involved in initiating placentation at the maternal tissue interface, although further research is required to explore this hypothesis. Of the top 50 *M. domestica* genes downregulated during early pregnancy, 14% are also downregulated in *S. crassicaudata* early pregnancy. These genes include transcription factors (*CBX2*, *SOX4*); the motor-protein encoding gene *KIF26B*; *VTCN1* (*B7-H4*), which negatively regulates T-cell immune responses^84^; and *IGFBP5*, which regulates the action of the insulin-like growth factors that mediate cell growth and also has apoptotic action^85^. Interestingly, transgenic mice that overexpress *IGFBP5* display reduced female fertility^85^, suggesting that the downregulation of this gene may be essential to early pregnancy across mammals.

## Conclusions

Genomic and transcriptomic methods are valuable tools for examining the physiology and evolution of marsupial pregnancy^15,17,18,86,87^. While the *M. domestica* transcriptome identified the importance of immune modulation for successful implantation and placentation in the marsupial uterus^18^, a range of other physiological changes is also required to support the internal incubation of the embryo prior to placentation. Our transcriptome study highlights the importance of such processes, including remodelling of the pre-implantation uterus, uterine quiescence, and nutrient provision via histotrophy prior to the development of the placenta; many of the genes underpinning these functions are shared across the dunnart and the opossum. The *S. crassicaudata* dataset is an ideal complement to the transcriptome of the opossum^15,18^, because these animals represent both major clades of marsupials (Australididelphia and Didelphimorphia, which diverged ~75 Mya^88^), and the cladistic derivation of both groups is similar (within-clade divergence of Dasyuridomorphia and Didelphimorphia both ~30 Mya^88^).

This transcriptome analysis reveals the importance of histotrophic nutrient transport prior to embryo implantation, before nutrient transport function is supplanted by the complex, nutritive placenta. Early pregnancy is a critical time for successful reproduction, and disruption to histotrophy could disrupt embryonic development. 40–50% of human pregnancies fail in the first trimester^21^, most of which is prior to the development of the definitive chorioallantoic placenta^89^. The putative gene functions identified here are similar to those in the pregnant uterus in other amniotes^34,35,90^. The conservation of genes underpinning pre-placental nutrient transport, gestational tissue remodelling, and uterine quiscence in amniote pregnancy is remarkable given that mammals and reptiles represent multiple independent origins of viviparity. Conserved elements underpinning aspects of early eutherian and marsupial pregnancy may provide new information for understanding human pregnancy disorders^91,92^, which is important given the difficulties in studying the human uterus *in vivo*^22^. This work furthers our understanding of the mechanisms underlying the survival of early embryos in our earliest live bearing mammalian ancestors, and highlights the importance of histotrophic nutrition to the embryo prior to the development of the nutritive placenta.

## Methods

### Tissue collection

Animals were held at a temperature-controlled breeding colony at the University of Sydney (in accordance with approved University of Sydney Animal Ethics Committee Protocol 704). Animals were housed either singly or in pairs, in plastic cages, and were provided with nesting boxes, nesting material, and enrichment material. Animals were held under the natural photocycle for Sydney (33°52’ S, 151°12’ E) and fed commercial cat food daily; water was provided *ad libitum*. Vaginal epithelial cells in smears of the urogenital sinus were examined microscopically to monitor estrous cycling of females^93,94^. A large number of cornified epithelial cells in the urine and a sharp increase in body mass defined the peak of oestrous^93,95,96^. Females were then paired with males, and the first day that sperm were detected in urine of the female was designated day 1 after mating^25,95^. Paired females were monitored for signs of pregnancy, including an increase in pouch area and vascularisation, loss of the furred pouch lining, and increase in body mass^93,96^.

Early pregnant (n = 3) and non-pregnant (n = 3) females were euthanised by CO_2_ inhalation, followed by immediate decapitation. The presence of embryos in excised uteri confirmed gestation, and the stage of pregnancy was determined by comparing size and morphology of embryos to the timetable of embryonic development^12^. We specifically targeted early-pregnant animals between days 6–8 of pregnancy, prior to implantation and placentation^12^, the stage of pregnancy where the shelled egg is present in the uterus.

### Transcriptome sequencing and annotation

Uterine samples were homogenised using the 3 mm steel bead TissueLyser II system (Qiagen, Hilden Germany) and QiaShredder (Qiagen). Total RNA was extracted using an RNeasy Plus Mini Kit (Qiagen), which includes an in-built DNAse treatment. RNA concentration and integrity were assessed using a Bioanalyzer (Agilent, Santa Clara CA) and only high quality RNA (RIN > 8) was used for downstream analysis. Samples for transcriptomics were sequenced after Truseq RNA sample prep with on an Illumina HiSeq 2500 with 100 bp paired-end sequencing, at the Ramaciotti Centre for Genomics, Sydney, Australia. Reads from all samples were combined in a *de novo* assembly with Trinity v2.0.4^28^, using the default parameters and the–trimmomatic and–min_kmer_cov 2 options. To assess the assembly completeness we used BUSCO v2.0.1^29^ with the default parameters in the transcriptome mode (-m tran), and searched against the tetrapod set of orthologs (tetrapoda_odb9). We used Kallisto^30^ to estimate abundance and DESeq2^31^ to call differential expression as implemented in the Trinity pipeline. We assessed correlation of gene expression between samples using the PtR script in Trinity. We annotated transcripts and assigned GO terms using the default parameters of the Trinotate pipeline v3.0.2^28^; which allowed us to identify particular gene functions on which to focus our analyses. Graphical representation of enriched GO terms was carried out using the cateGOrizer tool^97^. KEGG pathway analysis of annotated genes was carried out using DAVID version 6.8 (available: http://david.abcc.ncifcrf.gov/home.jsp, last accessed June 2017)^98^, using EASE score of 0.1 and *M. domestica* as background. P-values were Benjamini-Hochberg corrected to account for multiple hypothesis testing.

Differentially expressed genes between non-pregnant and pre-implantation uterus in *M. domestica* were compared to the *S. crassicaudata* uterine gene expression data using discontiguous megablasts optimised for cross-species comparison, using the –task dc-megablast option and the default parameters. *Monodelphis domestica* transcripts^18^ identified as differentially expressed between non-pregnant and mid-gravid (pre-implantation) uterus (adjusted P < 0.001) were searched against the *S. crassicaudata* uterine transcriptome assembly, and the results compared to the *S. crassicaudata* differential gene expression results from DESeq2. Differentially expressed genes shared between the two species were analysed using the DAVID functional annotation tool version 6.8 (available: http://david.abcc.ncifcrf.gov/home.jsp, last accessed November 2017)^33^, with GO_ALL biological process, cellular component and molecular function terms, using *M. domestica* as background. The Functional Annotation Clustering option was used to group significantly enriched GO terms using a modified Fisher’s Exact Test by function and the DAVID Fuzzy clustering algorithm^33^. Grouping was performed using DAVID settings for highest stringency and P-values were Benjamini-Hochberg corrected to account for multiple hypothesis testing. KEGG pathway analysis using DAVID was carried out using an EASE score of 0.1 and Benjamini-Hochberg corrected P-values.

### Data availability statement

All sequence data have been uploaded to GenBank (BioProject ID PRJNA399240).

## Electronic supplementary material

Supplementary Material
