# Author Correction: Transcriptomic changes in the pre-implantation uterus highlight histotrophic nutrition of the developing marsupial embryo

**DOI:** 10.1038/s41598-020-68759-9

**Published:** 2020-07-22

**Authors:** Camilla M. Whittington, Denis O’Meally, Melanie K. Laird, Katherine Belov, Michael B. Thompson, Bronwyn M. McAllan

**Affiliations:** 1 The University of Sydney, Sydney School of Veterinary Science, Camperdown, NSW 2006 Australia; 2 The University of Sydney, School of Life and Environmental Sciences, Camperdown, NSW 2006 Australia; 3 The University of Sydney, School of Medical Sciences, Camperdown, NSW 2006 Australia; 4 Present Address: Center for Gene Therapy, Beckman Research Institute of City of Hope, Duarte, CA 91024 USA

Correction to: *Scientific Reports* https://doi.org/10.1038/s41598-018-20744-z, published online 05 February 2018

In the Supplementary Information file originally published with this Article, Supplementary Table 6 contained duplicated data from Supplementary Table 5. These errors have been corrected in the Supplementary Information that now accompanies the Article.

