# Supplementary material for: Transcriptomic changes in the pre-implantation uterus highlight histotrophic nutrition of the developing marsupial embryo
Source: Sci Rep. 2018 Feb 5;8:2412. doi: 10.1038/s41598-018-20744-z (PMC5799185; doi:10.1038/s41598-018-20744-z)
Supplement: Supplementary file 1 — Supplementary Material [file 41598_2018_20744_MOESM1_ESM.pdf]

Transcriptomic changes in the pre-implantation uterus highlight histotrophic nutrition of the developing marsupial embryo

Camilla M Whittington, Denis O'Meally, Melanie K Laird, Katherine Belov, Michael B Thompson, Bronwyn M McAllan

Supplementary Material

Supplementary Figure 1. Comparison of replicates across pregnancy time points: a) Pearson correlation matrix for pairwise sample comparisons based on log2-transformed expression levels (transcripts per million) of all transcripts with at least 10 reads when summed across all samples. b) Principal Component Analysis plot based on log2-transformed expression levels (transcripts per million) for all transcripts with at least 10 reads when summed across all samples. NP: non-pregnant uterus; EP: early pregnant/pre-implantation uterus.

a.

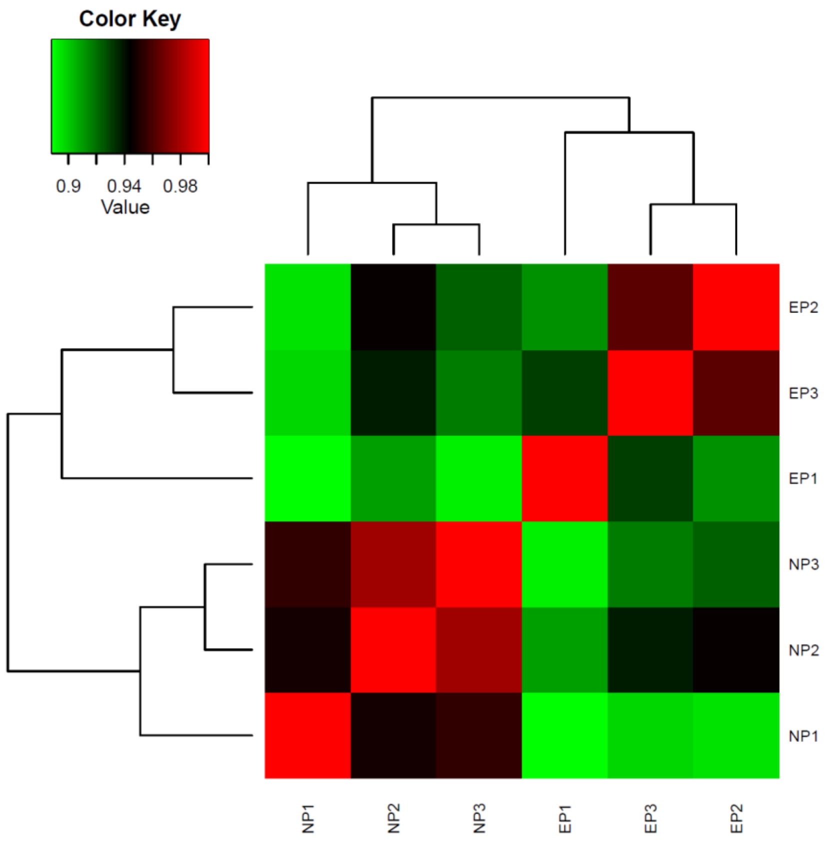

b)

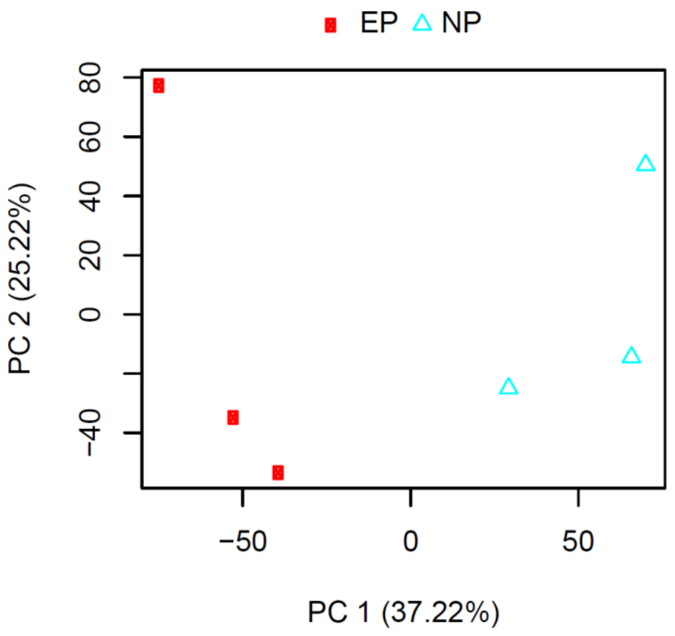

Supplementary Figure 2. Graphical representation of enriched GO categories for genes upregulated during early pregnancy, using cateGOrizer and mapping of Trinotate-derived GO terms (P<0.001) to "GO_slim" ancestor terms by single count. Ancestor terms (x-axis) containing at least two GO terms are shown.

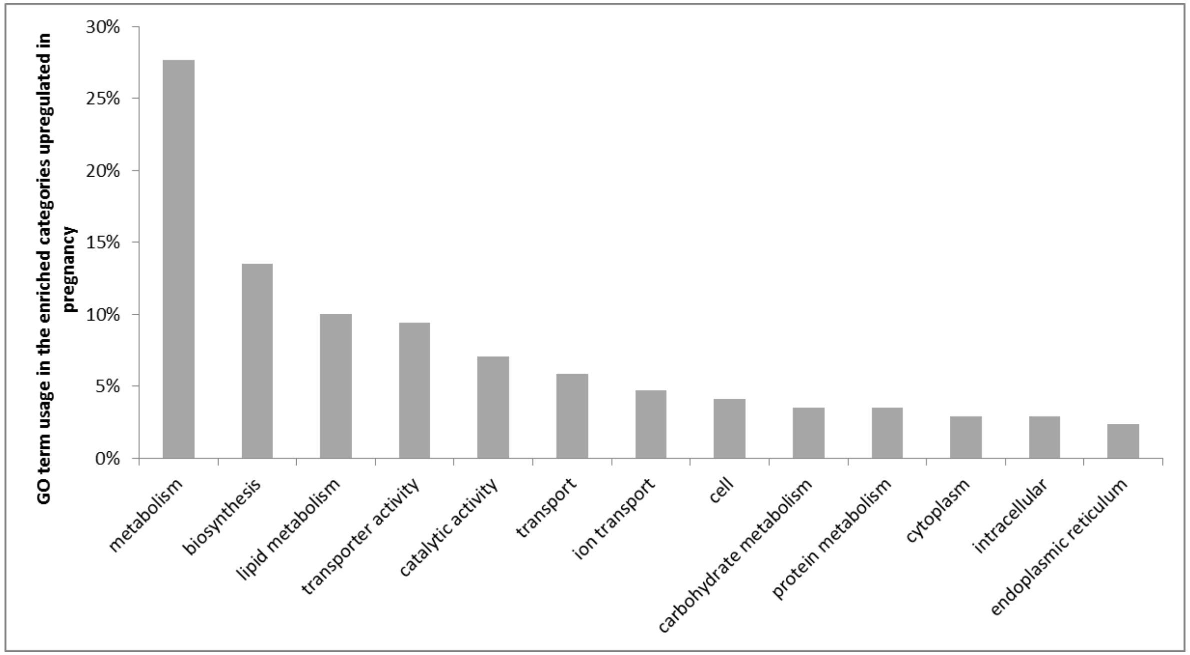

Supplementary Figure 3. Graphical representation of enriched GO categories for genes downregulated during early pregnancy, using cateGOrizer and mapping of Trinotate-derived GO terms (P<0.001) to "GO_slim" ancestor terms by single count. Ancestor terms (x-axis) containing at least two GO terms are shown.

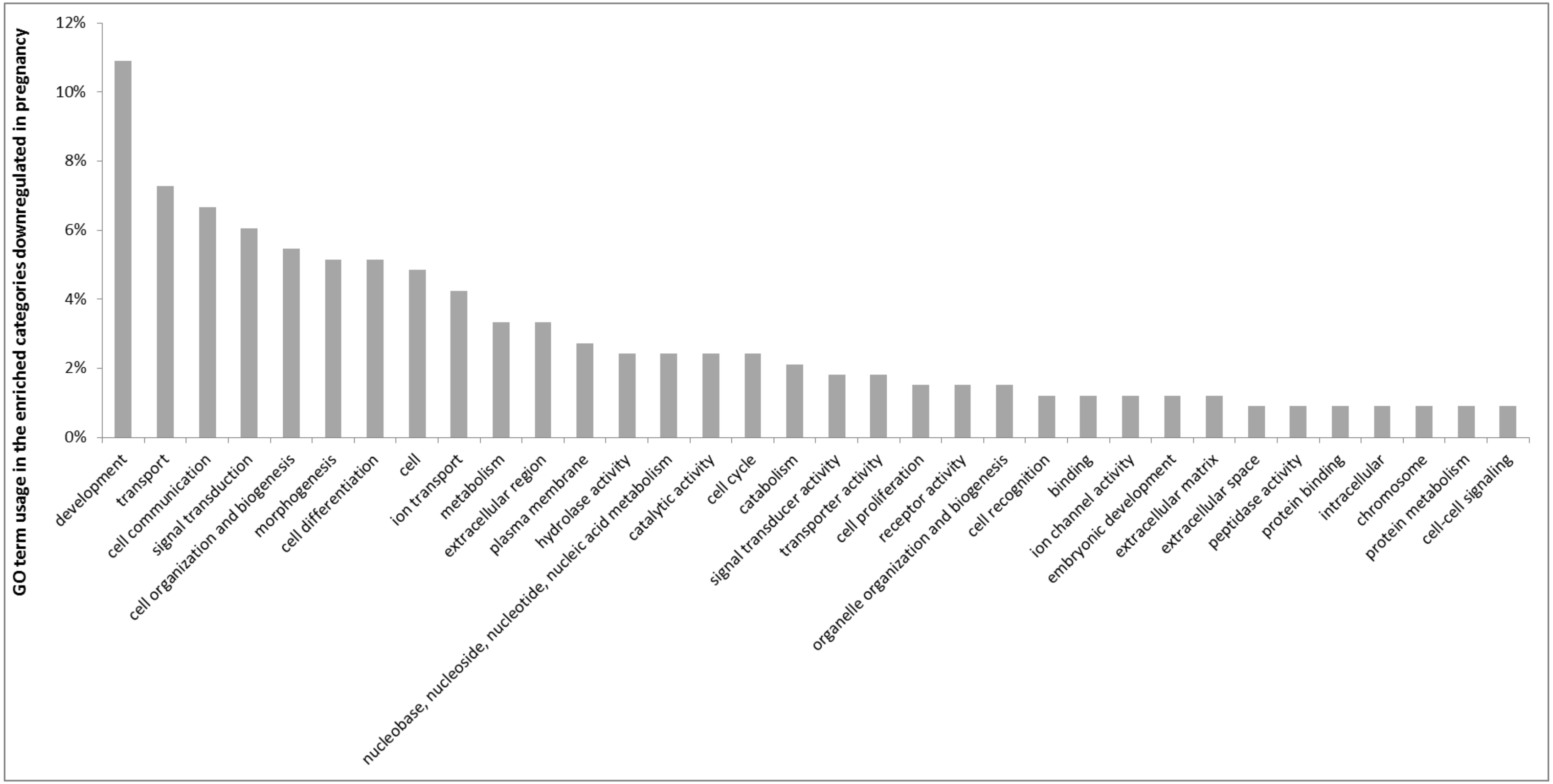

Supplementary Table 1. Gene ontology analysis of genes upregulated during pregnancy (P<0.001) using Trinotate. Significantly enriched GO terms (P<0.001) are shown, grouped by category.		
**GO category**	**GO term**	**P-value**
**Biological Process**		
GO:1901617	organic hydroxy compound biosynthetic process	4.24E-14
GO:0008202	steroid metabolic process	5.19E-13
GO:0006066	alcohol metabolic process	5.57E-13
GO:1901615	organic hydroxy compound metabolic process	1.26E-12
GO:0008610	lipid biosynthetic process	3.21E-12
GO:0006629	lipid metabolic process	4.69E-12
GO:0016125	sterol metabolic process	5.62E-12
GO:0006694	steroid biosynthetic process	6.29E-12
GO:0044283	small molecule biosynthetic process	2.10E-11
GO:0046165	alcohol biosynthetic process	2.90E-11
GO:0008203	cholesterol metabolic process	3.99E-11
GO:0044711	single-organism biosynthetic process	7.69E-10
GO:0044281	small molecule metabolic process	1.89E-09
GO:0016126	sterol biosynthetic process	7.93E-09
GO:0006695	cholesterol biosynthetic process	9.11E-09
GO:0006811	ion transport	5.55E-07
GO:0006777	Mo-molybdopterin cofactor biosynthetic process	7.19E-06
GO:0019720	Mo-molybdopterin cofactor metabolic process	7.19E-06
GO:0090407	organophosphate biosynthetic process	7.95E-06
GO:1901264	carbohydrate derivative transport	1.21E-05
GO:0032324	molybdopterin cofactor biosynthetic process	1.29E-05
GO:0043545	molybdopterin cofactor metabolic process	1.29E-05
GO:0051189	prosthetic group metabolic process	1.29E-05
GO:0072330	monocarboxylic acid biosynthetic process	1.31E-05
GO:0044255	cellular lipid metabolic process	1.33E-05
GO:0019637	organophosphate metabolic process	2.22E-05
GO:0016053	organic acid biosynthetic process	3.59E-05
GO:0046394	carboxylic acid biosynthetic process	3.59E-05
GO:0032787	monocarboxylic acid metabolic process	3.91E-05
GO:0006114	glycerol biosynthetic process	4.23E-05
GO:0019401	alditol biosynthetic process	4.23E-05
GO:0044765	single-organism transport	5.22E-05
GO:0008654	phospholipid biosynthetic process	6.14E-05
GO:0008299	isoprenoid biosynthetic process	7.13E-05
GO:0036498	IRE1-mediated unfolded protein response	8.85E-05
GO:0006820	anion transport	1.12E-04
GO:0006699	bile acid biosynthetic process	1.51E-04
GO:0006817	phosphate ion transport	2.66E-04
GO:0019752	carboxylic acid metabolic process	2.68E-04
GO:0006644	phospholipid metabolic process	2.69E-04
GO:0051186	cofactor metabolic process	2.92E-04
GO:0015711	organic anion transport	3.09E-04
GO:1902578	single-organism localization	3.19E-04
GO:0006720	isoprenoid metabolic process	3.45E-04
GO:0006487	protein N-linked glycosylation	3.52E-04
GO:0019348	dolichol metabolic process	3.87E-04

GO:0015991	ATP hydrolysis coupled proton transport	3.95E-04
GO:0035435	phosphate ion transmembrane transport	4.03E-04
GO:0005975	carbohydrate metabolic process	4.36E-04
GO:0016051	carbohydrate biosynthetic process	4.76E-04
GO:0046364	monosaccharide biosynthetic process	4.88E-04
GO:0015988	energy coupled proton transmembrane transport, against electrochemical gradient	4.98E-04
GO:0044723	single-organism carbohydrate metabolic process	5.12E-04
GO:0009108	coenzyme biosynthetic process	6.18E-04
GO:0015718	monocarboxylic acid transport	6.35E-04
GO:0043436	oxoacid metabolic process	7.28E-04
GO:0030641	regulation of cellular pH	7.28E-04
GO:0051453	regulation of intracellular pH	7.28E-04
GO:0016093	polyprenol metabolic process	7.62E-04
GO:0006082	organic acid metabolic process	8.84E-04
GO:0008206	bile acid metabolic process	9.46E-04
**Cellular component**		
GO:0016021	integral component of membrane	4.70E-18
GO:0031224	intrinsic component of membrane	7.52E-17
GO:0044425	membrane part	2.71E-14
GO:0044432	endoplasmic reticulum part	4.28E-11
GO:0098588	bounding membrane of organelle	1.63E-09
GO:0005789	endoplasmic reticulum membrane	1.97E-09
GO:0098589	membrane region	1.40E-08
GO:0031090	organelle membrane	1.65E-07
GO:0030176	integral component of endoplasmic reticulum membrane	9.90E-06
GO:0019008	molybdopterin synthase complex	1.17E-05
GO:0031227	intrinsic component of endoplasmic reticulum membrane	1.55E-05
GO:0016020	membrane	2.68E-04
GO:0044194	cytolytic granule	3.59E-04
GO:0016324	apical plasma membrane	6.15E-04
GO:0031301	integral component of organelle membrane	7.80E-04
**Molecular function**		
GO:0022857	transmembrane transporter activity	1.31E-08
GO:1901505	carbohydrate derivative transporter activity	3.45E-07
GO:0005215	transporter activity	5.08E-07
GO:0022892	substrate-specific transporter activity	8.44E-07
GO:0022891	substrate-specific transmembrane transporter activity	1.27E-06
GO:0033764	steroid dehydrogenase activity, acting on the CH-OH group of donors, NAD or NADP as acceptor	2.04E-06
GO:0016229	steroid dehydrogenase activity	4.36E-06
GO:0015075	ion transmembrane transporter activity	6.51E-06
GO:1901677	phosphate transmembrane transporter activity	7.30E-06
GO:0022804	active transmembrane transporter activity	9.80E-06
GO:0016614	oxidoreductase activity, acting on CH-OH group of donors	2.28E-05
GO:0015605	organophosphate ester transmembrane transporter activity	2.62E-05
GO:0016491	oxidoreductase activity	4.90E-05
GO:0005338	nucleotide-sugar transmembrane transporter activity	9.24E-05
GO:0016616	oxidoreductase activity, acting on the CH-OH group of donors, NAD or NADP as acceptor	9.35E-05
GO:0008324	cation transmembrane transporter activity	1.36E-04
GO:0004307	ethanolaminephosphotransferase activity	1.61E-04
GO:0016717	oxidoreductase activity, acting on paired donors, with oxidation of a pair of donors resulting in the reduction of molecular oxygen to two molecules of water	1.62E-04

GO:0015932	nucleobase-containing compound transmembrane transporter activity	2.33E-04
GO:0015291	secondary active transmembrane transporter activity	2.47E-04
GO:0004303	estradiol 17-beta-dehydrogenase activity	2.82E-04
GO:0036442	hydrogen-exporting ATPase activity	3.17E-04
GO:0015078	hydrogen ion transmembrane transporter activity	4.17E-04
GO:0008553	hydrogen-exporting ATPase activity, phosphorylative mechanism	6.66E-04
GO:0004745	retinol dehydrogenase activity	8.76E-04
GO:0047035	testosterone dehydrogenase (NAD+) activity	9.92E-04

Supplementary Table 2. Gene ontology analysis of genes downregulated during pregnancy (P<0.001) using Trinotate. Significantly enriched GO terms (P<0.001) are shown, grouped by category.		
**GO category**	**GO term**	**P-value**
**Biological process**		
GO:0006958	complement activation, classical pathway	2.25E-25
GO:0072376	protein activation cascade	3.77E-24
GO:0006956	complement activation	5.60E-24
GO:0006959	humoral immune response	2.57E-19
GO:0002433	immune response-regulating cell surface receptor signaling pathway involved in phagocytosis	3.02E-16
GO:0038096	Fc-gamma receptor signaling pathway involved in phagocytosis	3.02E-16
GO:0038094	Fc-gamma receptor signaling pathway	5.34E-16
GO:0002431	Fc receptor mediated stimulatory signaling pathway	1.35E-15
GO:0007166	cell surface receptor signaling pathway	1.22E-14
GO:0002250	adaptive immune response	1.89E-12
GO:0038095	Fc-epsilon receptor signaling pathway	2.00E-12
GO:0007165	signal transduction	1.24E-11
GO:0050900	leukocyte migration	1.35E-11
GO:0002429	immune response-activating cell surface receptor signaling pathway	2.17E-11
GO:0006910	phagocytosis, recognition	2.48E-11
GO:0002377	immunoglobulin production	2.54E-11
GO:0006898	receptor-mediated endocytosis	8.37E-11
GO:0006955	immune response	1.58E-10
GO:0002768	immune response-regulating cell surface receptor signaling pathway	1.80E-10
GO:0008037	cell recognition	2.09E-10
GO:0050853	B cell receptor signaling pathway	1.23E-09
GO:0048518	positive regulation of biological process	1.28E-09
GO:0010959	regulation of metal ion transport	1.47E-09
GO:0006911	phagocytosis, engulfment	2.26E-09
GO:0038093	Fc receptor signaling pathway	2.31E-09
GO:0009653	anatomical structure morphogenesis	2.52E-09
GO:1901379	regulation of potassium ion transmembrane transport	2.83E-09
GO:0016477	cell migration	3.45E-09
GO:0048731	system development	5.03E-09
GO:0048856	anatomical structure development	7.82E-09
GO:0002440	production of molecular mediator of immune response	8.75E-09
GO:0002253	activation of immune response	1.26E-08
GO:0048870	cell motility	1.71E-08
GO:0051239	regulation of multicellular organismal process	1.72E-08
GO:0002757	immune response-activating signal transduction	2.80E-08
GO:0010324	membrane invagination	3.25E-08
GO:0034765	regulation of ion transmembrane transport	3.42E-08
GO:0032501	multicellular organismal process	3.68E-08
GO:0043266	regulation of potassium ion transport	4.78E-08
GO:0044707	single-multicellular organism process	4.91E-08
GO:0048513	animal organ development	5.74E-08
GO:0044057	regulation of system process	6.89E-08
GO:0048584	positive regulation of response to stimulus	8.19E-08
GO:0048522	positive regulation of cellular process	1.09E-07
GO:0002764	immune response-regulating signaling pathway	1.17E-07

GO:0007155	cell adhesion	1.29E-07
GO:0034762	regulation of transmembrane transport	1.49E-07
GO:0043269	regulation of ion transport	1.88E-07
GO:0040011	locomotion	2.04E-07
GO:0032502	developmental process	2.14E-07
GO:0022610	biological adhesion	3.27E-07
GO:0044700	single organism signaling	3.73E-07
GO:0060065	uterus development	3.73E-07
GO:0042127	regulation of cell proliferation	3.98E-07
GO:0023052	signaling	4.56E-07
GO:1903522	regulation of blood circulation	5.15E-07
GO:0006928	movement of cell or subcellular component	7.19E-07
GO:0044767	single-organism developmental process	1.29E-06
GO:0007156	homophilic cell adhesion via plasma membrane adhesion molecules	1.64E-06
GO:0050871	positive regulation of B cell activation	2.53E-06
GO:0007267	cell-cell signaling	2.72E-06
GO:0035412	regulation of catenin import into nucleus	3.21E-06
GO:0050851	antigen receptor-mediated signaling pathway	3.68E-06
GO:0051240	positive regulation of multicellular organismal process	3.99E-06
GO:0098742	cell-cell adhesion via plasma-membrane adhesion molecules	4.25E-06
GO:0060306	regulation of membrane repolarization	4.52E-06
GO:0048598	embryonic morphogenesis	5.34E-06
GO:0086002	cardiac muscle cell action potential involved in contraction	5.61E-06
GO:0014031	mesenchymal cell development	5.64E-06
GO:0002684	positive regulation of immune system process	6.22E-06
GO:0008016	regulation of heart contraction	6.31E-06
GO:2000026	regulation of multicellular organismal development	6.35E-06
GO:0007507	heart development	8.58E-06
GO:0042692	muscle cell differentiation	8.95E-06
GO:0002027	regulation of heart rate	9.16E-06
GO:1901381	positive regulation of potassium ion transmembrane transport	9.27E-06
GO:0098609	cell-cell adhesion	9.77E-06
GO:0001501	skeletal system development	9.88E-06
GO:0050778	positive regulation of immune response	1.02E-05
GO:0010564	regulation of cell cycle process	1.09E-05
GO:0086001	cardiac muscle cell action potential	1.16E-05
GO:0034767	positive regulation of ion transmembrane transport	1.23E-05
GO:0051094	positive regulation of developmental process	1.23E-05
GO:0043268	positive regulation of potassium ion transport	1.41E-05
GO:0051302	regulation of cell division	1.48E-05
GO:0070509	calcium ion import	1.49E-05
GO:0051965	positive regulation of synapse assembly	1.50E-05
GO:0043270	positive regulation of ion transport	1.79E-05
GO:0001508	action potential	1.79E-05
GO:0006939	smooth muscle contraction	1.81E-05
GO:0048729	tissue morphogenesis	1.94E-05
GO:0014032	neural crest cell development	1.94E-05
GO:0051782	negative regulation of cell division	1.96E-05
GO:0090068	positive regulation of cell cycle process	2.54E-05
GO:0045931	positive regulation of mitotic cell cycle	2.54E-05

GO:0009214	cyclic nucleotide catabolic process	2.66E-05
GO:0007187	G-protein coupled receptor signaling pathway, coupled to cyclic nucleotide second messenger	3.00E-05
GO:0048864	stem cell development	3.06E-05
GO:0007517	muscle organ development	3.20E-05
GO:0051963	regulation of synapse assembly	3.35E-05
GO:0034764	positive regulation of transmembrane transport	3.65E-05
GO:0060429	epithelium development	3.85E-05
GO:0014829	vascular smooth muscle contraction	4.31E-05
GO:0007186	G-protein coupled receptor signaling pathway	4.38E-05
GO:0007188	adenylate cyclase-modulating G-protein coupled receptor signaling pathway	4.38E-05
GO:0009166	nucleotide catabolic process	4.48E-05
GO:0050864	regulation of B cell activation	4.54E-05
GO:0061061	muscle structure development	4.97E-05
GO:0051960	regulation of nervous system development	4.97E-05
GO:0050794	regulation of cellular process	5.04E-05
GO:0050807	regulation of synapse organization	5.49E-05
GO:0008284	positive regulation of cell proliferation	6.07E-05
GO:0009154	purine ribonucleotide catabolic process	6.56E-05
GO:0009888	tissue development	6.59E-05
GO:0051962	positive regulation of nervous system development	7.60E-05
GO:0042742	defense response to bacterium	7.75E-05
GO:0009261	ribonucleotide catabolic process	8.30E-05
GO:0006897	endocytosis	9.36E-05
GO:0007088	regulation of mitotic nuclear division	9.37E-05
GO:0007346	regulation of mitotic cell cycle	9.44E-05
GO:0086010	membrane depolarization during action potential	9.69E-05
GO:0051983	regulation of chromosome segregation	9.87E-05
GO:0006198	cAMP catabolic process	9.92E-05
GO:0060372	regulation of atrial cardiac muscle cell membrane repolarization	1.10E-04
GO:1901292	nucleoside phosphate catabolic process	1.11E-04
GO:0050678	regulation of epithelial cell proliferation	1.12E-04
GO:0008038	neuron recognition	1.24E-04
GO:0051382	kinetochore assembly	1.27E-04
GO:0051783	regulation of nuclear division	1.33E-04
GO:0044763	single-organism cellular process	1.34E-04
GO:0045787	positive regulation of cell cycle	1.36E-04
GO:1901201	regulation of extracellular matrix assembly	1.37E-04
GO:0050793	regulation of developmental process	1.58E-04
GO:0042310	vasoconstriction	1.60E-04
GO:0009187	cyclic nucleotide metabolic process	1.66E-04
GO:0032879	regulation of localization	1.68E-04
GO:0065007	biological regulation	1.70E-04
GO:0050896	response to stimulus	1.70E-04
GO:0009887	animal organ morphogenesis	1.71E-04
GO:0007224	smoothened signaling pathway	1.73E-04
GO:1903524	positive regulation of blood circulation	1.82E-04
GO:0003208	cardiac ventricle morphogenesis	1.82E-04
GO:0007389	pattern specification process	1.88E-04
GO:0003008	system process	1.90E-04
GO:0003215	cardiac right ventricle morphogenesis	1.92E-04

GO:0050789	regulation of biological process	1.94E-04
GO:0060284	regulation of cell development	1.96E-04
GO:0044699	single-organism process	2.03E-04
GO:0050808	synapse organization	2.07E-04
GO:0008285	negative regulation of cell proliferation	2.22E-04
GO:0030856	regulation of epithelial cell differentiation	2.23E-04
GO:0001658	branching involved in ureteric bud morphogenesis	2.54E-04
GO:0051241	negative regulation of multicellular organismal process	2.61E-04
GO:0086012	membrane depolarization during cardiac muscle cell action potential	2.67E-04
GO:0002009	morphogenesis of an epithelium	2.76E-04
GO:0035910	ascending aorta morphogenesis	2.79E-04
GO:0051383	kinetochore organization	3.02E-04
GO:0030198	extracellular matrix organization	3.03E-04
GO:0055012	ventricular cardiac muscle cell differentiation	3.09E-04
GO:0055006	cardiac cell development	3.17E-04
GO:0007409	axonogenesis	3.30E-04
GO:0043062	extracellular structure organization	3.39E-04
GO:0051251	positive regulation of lymphocyte activation	3.47E-04
GO:0010460	positive regulation of heart rate	4.01E-04
GO:0090075	relaxation of muscle	4.26E-04
GO:0051093	negative regulation of developmental process	4.26E-04
GO:0048583	regulation of response to stimulus	4.34E-04
GO:0051924	regulation of calcium ion transport	4.54E-04
GO:0006195	purine nucleotide catabolic process	4.57E-04
GO:0003206	cardiac chamber morphogenesis	4.58E-04
GO:0035987	endodermal cell differentiation	4.73E-04
GO:1901342	regulation of vasculature development	4.73E-04
GO:0035150	regulation of tube size	4.87E-04
GO:0050880	regulation of blood vessel size	4.87E-04
GO:0045595	regulation of cell differentiation	4.92E-04
GO:0071459	protein localization to chromosome, centromeric region	4.95E-04
GO:0007154	cell communication	4.96E-04
GO:0009617	response to bacterium	4.99E-04
GO:0008283	cell proliferation	5.58E-04
GO:0071320	cellular response to cAMP	5.60E-04
GO:0016055	Wnt signaling pathway	5.64E-04
GO:0035413	positive regulation of catenin import into nucleus	6.03E-04
GO:0055001	muscle cell development	6.07E-04
GO:0055013	cardiac muscle cell development	6.50E-04
GO:0030111	regulation of Wnt signaling pathway	6.68E-04
GO:0003012	muscle system process	6.77E-04
GO:0003002	regionalization	6.80E-04
GO:0055002	striated muscle cell development	6.84E-04
GO:0022603	regulation of anatomical structure morphogenesis	6.90E-04
GO:0051726	regulation of cell cycle	7.85E-04
GO:0035239	tube morphogenesis	8.06E-04
GO:0050767	regulation of neurogenesis	8.37E-04
GO:0045839	negative regulation of mitotic nuclear division	8.45E-04
GO:0030326	embryonic limb morphogenesis	8.50E-04
GO:0032989	cellular component morphogenesis	9.21E-04

GO:0060485	mesenchyme development	9.25E-04
GO:0060307	regulation of ventricular cardiac muscle cell membrane repolarization	9.30E-04
GO:0002376	immune system process	9.40E-04
GO:0007413	axonal fasciculation	9.62E-04
GO:0035113	embryonic appendage morphogenesis	9.63E-04
GO:1903054	negative regulation of extracellular matrix organization	9.75E-04
**Cellular component**		
GO:0005576	extracellular region	1.67E-16
GO:0042571	immunoglobulin complex, circulating	3.87E-16
GO:0044459	**plasma membrane part**	5.35E-16
GO:0019814	immunoglobulin complex	1.38E-14
GO:0072562	blood microparticle	2.42E-12
GO:0005615	extracellular space	8.45E-12
GO:0005886	**plasma membrane**	9.79E-12
GO:0005887	integral component of plasma membrane	8.34E-09
GO:0034703	cation channel complex	2.54E-08
GO:0034702	ion channel complex	2.64E-08
GO:0031226	intrinsic component of plasma membrane	2.75E-08
GO:0044425	membrane part	4.08E-08
GO:1902495	transmembrane transporter complex	1.13E-07
GO:1990351	transporter complex	1.63E-07
GO:0031224	intrinsic component of membrane	2.51E-07
GO:0009897	external side of plasma membrane	4.85E-07
GO:0005578	proteinaceous extracellular matrix	1.27E-06
GO:0016021	integral component of membrane	4.56E-06
GO:0044421	extracellular region part	5.26E-06
GO:0008076	voltage-gated potassium channel complex	1.23E-05
GO:0034705	potassium channel complex	1.49E-05
GO:0000776	kinetochore	1.81E-05
GO:0005891	voltage-gated calcium channel complex	1.95E-05
GO:0098552	side of membrane	3.71E-05
GO:0005590	collagen type VII trimer	6.68E-05
GO:0097458	neuron part	7.45E-05
GO:0033267	axon part	8.10E-05
GO:0044295	axonal growth cone	8.73E-05
GO:0000775	chromosome, centromeric region	9.32E-05
GO:0043005	neuron projection	1.68E-04
GO:0044420	extracellular matrix component	1.90E-04
GO:0005604	basement membrane	3.62E-04
GO:0000777	condensed chromosome kinetochore	3.68E-04
GO:1990454	L-type voltage-gated calcium channel complex	3.88E-04
GO:0031225	anchored component of membrane	3.95E-04
GO:0034704	calcium channel complex	4.42E-04
GO:0031012	extracellular matrix	4.60E-04
GO:0098590	plasma membrane region	6.89E-04
GO:0030934	anchoring collagen complex	8.86E-04
**Molecular function**		
GO:0003823	antigen binding	3.13E-37
GO:0004252	serine-type endopeptidase activity	1.68E-12
GO:0008236	serine-type peptidase activity	1.07E-11

GO:0017171	serine hydrolase activity	1.79E-11
GO:0034987	immunoglobulin receptor binding	2.05E-11
GO:0004872	receptor activity	8.50E-10
GO:0060089	molecular transducer activity	2.46E-09
GO:0038023	signaling receptor activity	3.28E-08
GO:0004871	signal transducer activity	9.05E-08
GO:0004888	transmembrane signaling receptor activity	9.40E-08
GO:0004930	G-protein coupled receptor activity	3.61E-06
GO:0017147	Wnt-protein binding	5.29E-05
GO:1904929	coreceptor activity involved in Wnt signaling pathway, planar cell polarity pathway	6.39E-05
GO:0004115	3',5'-cyclic-AMP phosphodiesterase activity	6.73E-05
GO:0004114	3',5'-cyclic-nucleotide phosphodiesterase activity	6.97E-05
GO:0022843	voltage-gated cation channel activity	7.17E-05
GO:0004112	cyclic-nucleotide phosphodiesterase activity	9.27E-05
GO:0030545	receptor regulator activity	1.28E-04
GO:0005244	voltage-gated ion channel activity	1.47E-04
GO:0022832	voltage-gated channel activity	1.47E-04
GO:0045499	chemorepellent activity	2.67E-04
GO:0005102	receptor binding	4.16E-04
GO:0086059	voltage-gated calcium channel activity involved SA node cell action potential	4.18E-04
GO:0022836	gated channel activity	4.94E-04
GO:0004175	endopeptidase activity	5.38E-04
GO:0030546	receptor activator activity	6.60E-04
GO:0005267	potassium channel activity	7.44E-04
GO:0008081	phosphoric diester hydrolase activity	7.82E-04
GO:0030297	transmembrane receptor protein tyrosine kinase activator activity	9.76E-04

Supplementary Table 3. Comparison of differentially expressed genes upregulated in early pregnancy between *Monodelphis domestica* (opossum) and *Sminthopsis crassicaudata* (fat-tailed dunnart)

Opossum upregulated in early pregnancy rank	Opossum upregulated in early pregnancy	Gene Symbol/transcript ID	Expression levels in dunnart early pregnancy compared to non-pregnant
2	ENSMODG00000021489	ABHD3	Up
10	ENSMODG00000016512	PCK1	Up
11	ENSMODG00000007389	ZNF750	Up
23	ENSMODG00000001937	TC2N	Up
26	ENSMODG00000024759	FGFBP1	Up
29	ENSMODG00000013633	LVRN	Up
30	ENSMODG00000025675	ENSMODT00000040104	Up
35	ENSMODG00000009972	ELF5	Up
46	ENSMODG00000011892	CTAGE5	Up
49	ENSMODG00000024056	KCTD14	Up
63	ENSMODG00000020427	ENSMODT00000026009	Up
70	ENSMODG00000024158	GJB6	Up
71	ENSMODG00000016406	DNASE1	Up
73	ENSMODG00000005871	NAGPA	Up
82	ENSMODG00000004556	EDEM1	Up
86	ENSMODG00000014803	ATP6V1C2	Up
89	ENSMODG00000027766	SLC39A11	Up
92	ENSMODG00000002863	FICD	Up
96	ENSMODG00000005464	ACAD8	Up
105	ENSMODG00000003065	PCYT2	Up
108	ENSMODG00000001747	TMEM171	Up
110	ENSMODG00000029505	SLC27A5	Up
112	ENSMODG00000011909	ENSMODT00000015198	Up
122	ENSMODG00000020677	TMEM165	Up
124	ENSMODG00000008334	SLC28A3	Up
127	ENSMODG00000013130	KCNK6	Up
132	ENSMODG00000017184	LRRC26	Up
142	ENSMODG00000025295	MID1IP1	Up
148	ENSMODG00000006872	ARFGAP3	Up
151	ENSMODG00000029422	ENSMODT00000044326	Up
154	ENSMODG00000023911	PAQR7	Up
155	ENSMODG00000019764	HSPA5	Up
156	ENSMODG00000004087	TMEM135	Up
160	ENSMODG00000020578	PICK1	Up
174	ENSMODG00000004376	SLC16A6	Up
175	ENSMODG00000023300	CITED4	Up
187	ENSMODG00000003073	MDH1	Up
208	ENSMODG00000012906	GMPPB	Up
216	ENSMODG00000028383	ENSMODT00000042974	Up
227	ENSMODG00000029803	GLRX	Up
229	ENSMODG00000019840	ENSMODT00000025198	Up
234	ENSMODG00000004576	ENSMODT00000005760	Up
237	ENSMODG00000020949	HSPA13	Up

239	ENSMODG00000007130	SPCS2	Up
241	ENSMODG00000012667	ALG2	Up
244	ENSMODG00000007831	PC	Up
245	ENSMODG00000019574	ATP6V1G1	Up
257	ENSMODG00000007110	HID1	Up
258	ENSMODG00000014990	AOX2	Up
260	ENSMODG00000001049	ENSMODT00000001270	Up
268	ENSMODG00000017269	ENSMODT00000021930	Up
275	ENSMODG00000020424	LMAN1	Up
279	ENSMODG00000004631	AP3D1	Up
280	ENSMODG00000014033	PAFAH2	Up
282	ENSMODG00000017742	CMAS	Up
291	ENSMODG00000009431	ATP6V1H	Up
292	ENSMODG00000028986	ENSMODT00000041991	Up
295	ENSMODG00000003558	GOT1	Up
313	ENSMODG00000016892	TMEM79	Up
319	ENSMODG00000010704	SGSM2	Up
333	ENSMODG00000012664	SEC61B	Up
335	ENSMODG00000015775	SLC33A1	Up
337	ENSMODG00000008033	SPTBN2	Up
339	ENSMODG00000005737	ENSMODT00000007254	Up
357	ENSMODG00000013307	ENSMODT00000016946	Up
366	ENSMODG00000017776	ENSMODT00000022551	Up
380	ENSMODG00000013173	FAM174B	Up
382	ENSMODG00000014811	PDIA6	Up
384	ENSMODG00000016783	SLCO4A1	Up
388	ENSMODG00000013049	IL22RA1	Up
389	ENSMODG00000025745	BPI	Up
402	ENSMODG00000020086	ELL2	Up
411	ENSMODG00000016096	SEC62	Up
432	ENSMODG00000001149	ENSMODT00000001398	Up
436	ENSMODG00000018400	IBTK	Up
440	ENSMODG00000011530	CALR	Up
441	ENSMODG00000002317	OTUB2	Up
457	ENSMODG00000007498	UCP2	Up
462	ENSMODG00000006516	ENSMODT00000008247	Up
475	ENSMODG00000002333	ABHD6	Up
483	ENSMODG00000010088	SYVN1	Up
487	ENSMODG00000008145	RNF121	Up
500	ENSMODG00000013285	DPAGT1	Up
513	ENSMODG00000002182	KIAA1324	Up
515	ENSMODG00000006552	ENTPD5	Up
516	ENSMODG00000013280	C2CD2L	Up
521	ENSMODG00000005497	TRABD	Up
522	ENSMODG00000012166	ENSMODT00000015522	Up
536	ENSMODG00000014091	SLC30A2	Up
540	ENSMODG00000011389	MGAT2	Up
541	ENSMODG00000003980	ENSMODT00000004996	Up
554	ENSMODG00000013117	SPINT2	Up
555	ENSMODG00000025751	ENSMODT00000040198	Up
568	ENSMODG00000016033	HDLBP	Up
569	ENSMODG00000003907	ZFYVE27	Up

576	ENSMODG00000011701	HBEGF	Up
595	ENSMODG00000016955	RAB25	Up
597	ENSMODG00000006471	GALNT3	Up
600	ENSMODG00000015439	ENSMODT00000019618	Up
610	ENSMODG00000012957	LONP2	Up
615	ENSMODG00000008253	ENSMODT00000010450	Up
634	ENSMODG00000019878	SLC35C1	Up
637	ENSMODG00000029016	ENSMODT00000042453	Up
654	ENSMODG00000028440	ENSMODT00000043357	Up
655	ENSMODG00000010048	SLC35A2	Up
660	ENSMODG00000025689	SFT2D1	Up
678	ENSMODG00000016888	GLMP	Up
697	ENSMODG00000023271	MPC1	Up
703	ENSMODG00000001018	ENSMODT00000001240	Up
707	ENSMODG00000000393	ENSMODT00000000482	Up
713	ENSMODG00000007544	TMEM9B	Up
729	ENSMODG00000011098	LACTB	Up
735	ENSMODG00000012475	KMT5A	Up
743	ENSMODG00000007725	ENSMODT00000009783	Up
771	ENSMODG00000008149	MTFR1	Up
780	ENSMODG00000007786	TM9SF2	Up
797	ENSMODG00000006936	ENSMODT00000008767	Up
800	ENSMODG00000001551	ENSMODT00000001933	Up
805	ENSMODG00000018506	ENSMODT00000023489	Up
861	ENSMODG00000021023	SLC37A1	Up
864	ENSMODG00000015034	CAMSAP3	Up
873	ENSMODG00000007477	LLGL2	Up
886	ENSMODG00000004298	NR4A2	Up
887	ENSMODG00000003505	ENTPD7	Up
892	ENSMODG00000013534	MPZL3	Up
900	ENSMODG00000016207	ADA	Up
967	ENSMODG00000002082	ENSMODT00000002589	Up
993	ENSMODG00000019181	RASSF6	Up
994	ENSMODG00000010128	SMIM19	Up
1019	ENSMODG00000010248	EBP	Up
1035	ENSMODG00000004533	ENSMODT00000005714	Up
1042	ENSMODG00000003824	Mar-02	Up
1052	ENSMODG00000010897	KIAA2013	Up
1056	ENSMODG00000009152	SLC46A3	Up
1069	ENSMODG00000001874	PRSS8	Up
1074	ENSMODG00000005215	GDAP2	Up
1079	ENSMODG00000018054	PLEKHG6	Up
1083	ENSMODG00000001335	TMCO1	Up
1087	ENSMODG00000001338	OVGP1	Up
1092	ENSMODG00000013318	HYOU1	Up
1107	ENSMODG00000017512	SLC2A12	Up
1111	ENSMODG00000014843	ENSMODT00000018900	Up
1127	ENSMODG00000020405	TARS	Up
1172	ENSMODG00000025494	ENSMODT00000039879	Up
1195	ENSMODG00000015616	RNF181	Up
1198	ENSMODG00000012883	ITFG1	Up
1200	ENSMODG00000020200	OSBP	Up

1225	ENSMODG00000004392	ALAD	Up
1227	ENSMODG00000011589	MFSD6	Up
1243	ENSMODG00000018503	COX7A2	Up
1246	ENSMODG00000009610	PLA2G3	Up
1257	ENSMODG00000021308	ZMAT3	Up
1266	ENSMODG00000009825	ATP6V1B2	Up
1271	ENSMODG00000010647	FGFR1	Up
1276	ENSMODG00000011281	WDR44	Up
1283	ENSMODG00000014530	FDX1	Up
1289	ENSMODG00000007832	KDELR2	Up
1293	ENSMODG00000016353	SLC26A4	Up
1310	ENSMODG00000024936	ENSMODT00000037894	Up
1311	ENSMODG00000012796	ARRDC4	Up
1335	ENSMODG00000012865	ENSMODT00000016393	Up
1340	ENSMODG00000012130	TUSC2	Up
1343	ENSMODG00000025676	ENSMODT00000040105	Up
1396	ENSMODG00000001580	MFSD11	Up
1398	ENSMODG00000009940	CCDC47	Up
1417	ENSMODG00000017799	NUS1	Up
1426	ENSMODG00000016356	DPM1	Up
1440	ENSMODG00000018634	PLEKHB2	Up
1444	ENSMODG00000002920	ENSMODT00000003634	Up
1476	ENSMODG00000010863	PLPBP	Up
1497	ENSMODG00000019527	SRXN1	Up
1498	ENSMODG00000006643	MIER2	Up
1515	ENSMODG00000018416	ADIPOR2	Up
1530	ENSMODG00000009941	ADPGK	Up
1538	ENSMODG00000012826	FAM177A1	Up
1543	ENSMODG00000009989	EHF	Up
1595	ENSMODG00000000508	MMAA	Up
1598	ENSMODG00000002434	SLC9A2	Up
1660	ENSMODG00000018058	SCNN1A	Up
1661	ENSMODG00000000412	C11orf54	Up
1663	ENSMODG00000027638	ENSMODT00000042476	Up
1673	ENSMODG00000000983	EPHB6	Up
1686	ENSMODG00000010471	PCDH1	Up
1692	ENSMODG00000011248	ZDHHC3	Up
1711	ENSMODG00000001738	HSD3B7	Up
1712	ENSMODG00000013297	CCDC129	Up
1718	ENSMODG00000005175	ENSMODT00000006506	Up
1720	ENSMODG00000015241	ABHD5	Up
1730	ENSMODG00000023369	SLC31A1	Up
1731	ENSMODG00000003566	MMADHC	Up
1751	ENSMODG00000017579	AP4S1	Up
1762	ENSMODG00000028424	ENSMODT00000016018	Up
1773	ENSMODG00000023342	DOLK	Up
1775	ENSMODG00000006950	JPH1	Up
1779	ENSMODG00000014252	FAM110C	Up
1816	ENSMODG00000020355	RANBP3L	Up
1836	ENSMODG00000002787	MPC2	Up
1841	ENSMODG00000017363	AK2	Up
1857	ENSMODG00000027317	ENSMODT00000042023	Up

1868	ENSMODG00000015800	SELENOI	Up
1877	ENSMODG00000015908	ENSMODT00000020204	Up
1940	ENSMODG00000018427	ENSMODT00000023385	Up
1959	ENSMODG00000005064	ST14	Up
1988	ENSMODG00000007513	FADS3	Up
2007	ENSMODG00000015571	MREG	Up
2010	ENSMODG00000019459	MOCS2	Up
2039	ENSMODG00000005274	ENSMODT00000006639	Up
2044	ENSMODG00000006673	CPEB3	Up
2080	ENSMODG00000003518	C18orf32	Up
2101	ENSMODG00000021351	TSFM	Up
2104	ENSMODG00000007494	ENSMODT00000009482	Up
2118	ENSMODG00000007925	SIGMAR1	Up
309	ENSMODG00000009929	SLITRK6	Down
474	ENSMODG00000001007	CD38	Down
563	ENSMODG00000020487	TMTC2	Down
977	ENSMODG00000010737	SORCS1	Down
1017	ENSMODG00000023888	ENSMODT00000018506	Down
1085	ENSMODG00000003553	LYPD6	Down
1145	ENSMODG00000020685	DDIT4L	Down
1183	ENSMODG00000023889	ENSMODT00000020783	Down
1194	ENSMODG00000014588	ENSMODT00000018554	Down
1268	ENSMODG00000000894	CYB5R1	Down
1280	ENSMODG00000017345	SLC27A3	Down
1321	ENSMODG00000012207	H2AFY2	Down
1360	ENSMODG00000006103	FAAH2	Down
1524	ENSMODG00000028106	ENSMODT00000020785	Down
1636	ENSMODG00000000655	ENSMODT00000000802	Down
1837	ENSMODG00000023243	AMIGO1	Down
1974	ENSMODG00000014577	ENSMODT00000018538	Down
844	ENSMODG00000004378	ERMN	No change
177	ENSMODG00000012674	WFIKKN2	No change
251	ENSMODG00000000704	TMEM163	No change
496	ENSMODG00000028042	ARTN	No change
714	ENSMODG00000003864	TTC22	No change
274	ENSMODG00000003829	DMRTA1	No change
297	ENSMODG00000007561	TMEM55A	No change
165	ENSMODG00000011125	INA	No change
52	ENSMODG00000006339	ENSMODT00000008026	No change
792	ENSMODG00000012702	ABO	No change
54	ENSMODG00000005797	TMEM114	No change
1503	ENSMODG00000015794	MCCD1	No change
84	ENSMODG00000015572	PLB1	No change
1064	ENSMODG00000009553	SFTPC	No change
1258	ENSMODG00000000715	ACMSD	No change
1579	ENSMODG00000011550	MSTN	No change
1418	ENSMODG00000011393	ELOVL3	No change
2136	ENSMODG00000029229	ENSMODT00000041820	No change
1892	ENSMODG00000003927	MODO-UB	No change
1621	ENSMODG00000014883	ATP6V0A1	No change
719	ENSMODG00000015789	CRELD2	No change
1385	ENSMODG00000014115	ENSMODT00000017976	No change

2059	ENSMODG00000016165	YKT6	No change
188	ENSMODG00000025084	MGAT3	No change
172	ENSMODG00000013757	FKBP5	No change
1882	ENSMODG00000013774	CEP170B	No change
1486	ENSMODG00000013955	RSRP1	No change
1802	ENSMODG00000013961	TMEM50A	No change
243	ENSMODG00000000328	ENSMODT00000000399	No change
604	ENSMODG00000008556	RAB2A	No change
1760	ENSMODG00000008966	FAM83F	No change
1274	ENSMODG00000000340	ENSMODT00000000413	No change
59	ENSMODG00000008689	LIPC	No change
839	ENSMODG00000012139	PEPD	No change
1513	ENSMODG00000021423	PSMG2	No change
1737	ENSMODG00000011590	ENSMODT00000014770	No change
392	ENSMODG00000000788	GADD45A	No change
1846	ENSMODG00000010434	UROS	No change
1864	ENSMODG00000015637	NRBP1	No change
125	ENSMODG00000015638	NUPR1	No change
590	ENSMODG00000023373	HDHD3	No change
931	ENSMODG00000008269	DOCK9	No change
532	ENSMODG00000013780	PSD4	No change
439	ENSMODG00000001640	MCCC2	No change
1130	ENSMODG00000005259	TEKT5	No change
338	ENSMODG00000020661	CISD2	No change
320	ENSMODG00000011708	BET1	No change
2127	ENSMODG00000023932	MINOS1	No change
508	ENSMODG00000010507	DNAJC10	No change
803	ENSMODG00000001917	CKAP4	No change
249	ENSMODG00000002234	HSP90B1	No change
2160	ENSMODG00000019046	ENSMODT00000039458	No change
214	ENSMODG00000001904	ENSMODT00000002372	No change
2005	ENSMODG00000012079	LRRC8B	No change
1748	ENSMODG00000003519	SLC25A28	No change
1207	ENSMODG00000005552	CDK9	No change
370	ENSMODG00000011657	UBA5	No change
1632	ENSMODG00000002852	CBR4	No change
1237	ENSMODG00000014518	AARS	No change
1102	ENSMODG00000010588	ENSMODT00000013506	No change
1901	ENSMODG00000024615	ENSMODT00000036459	No change
1902	ENSMODG00000016549	VAPB	No change
831	ENSMODG00000015321	CHN2	No change
444	ENSMODG00000019312	DHRS13	No change
1188	ENSMODG00000010429	ABT1	No change
1921	ENSMODG00000020979	ETS2	No change
1917	ENSMODG00000013316	EPB41L4A	No change
287	ENSMODG00000003441	SLC30A7	No change
1854	ENSMODG00000005427	GSTZ1	No change
2169	ENSMODG00000012978	TCTA	No change
2000	ENSMODG00000021304	MFN1	No change
1300	ENSMODG00000018452	ENSMODT00000023418	No change
286	ENSMODG00000011796	GFPT1	No change
2159	ENSMODG00000015275	NOD1	No change

725	ENSMODG00000007316	ENSMODT00000009257	No change
987	ENSMODG00000006549	CDC34	No change
640	ENSMODG00000003046	MFAP3L	No change
837	ENSMODG00000004132	UQCR11	No change
394	ENSMODG00000000471	CCDC149	No change
1977	ENSMODG00000005167	GAMT	No change
756	ENSMODG00000009838	DENND4A	No change
2151	ENSMODG00000000432	HHIP	No change
833	ENSMODG00000002039	NSUN2	No change
13	ENSMODG00000002043	SRD5A1	No change
1803	ENSMODG00000013238	ENSMODT00000016867	No change
1143	ENSMODG00000014524	CAPN7	No change
1908	ENSMODG00000008458	YBEY	No change
715	ENSMODG00000002757	PADI2	No change
141	ENSMODG00000018414	BCKDHB	No change
1602	ENSMODG00000009391	MIEF1	No change
1100	ENSMODG00000011819	AAK1	No change
755	ENSMODG00000012678	TOB1	No change
505	ENSMODG00000018580	ARFIP2	No change
235	ENSMODG00000013272	REEP5	No change
718	ENSMODG00000011006	ENSMODT00000014032	No change
1819	ENSMODG00000000817	CREB3L3	No change
1126	ENSMODG00000011288	CDCP1	No change
34	ENSMODG00000001897	FER1L6	No change
539	ENSMODG00000006314	MYOF	No change
498	ENSMODG00000010231	LARS	No change
1679	ENSMODG00000008949	RAB17	No change
479	ENSMODG00000018340	TEAD4	No change
376	ENSMODG00000002543	KLHDC7A	No change
331	ENSMODG00000012925	ENSMODT00000016472	No change
973	ENSMODG00000006336	COX6C	No change
299	ENSMODG00000008326	COPG1	No change
750	ENSMODG00000018420	ELOVL4	No change
1474	ENSMODG00000002793	ATP13A2	No change
42	ENSMODG00000002104	SLC41A2	No change
622	ENSMODG00000006048	GRHL2	No change
170	ENSMODG00000006976	UNC13A	No change
93	ENSMODG00000015478	FAM3C	No change
1270	ENSMODG00000016373	ENSMODT00000020821	No change
492	ENSMODG00000005438	SERPINB11	No change
2074	ENSMODG00000019794	RALGPS1	No change
1224	ENSMODG00000001566	JMJD6	No change
1250	ENSMODG00000021168	ENSMODT00000026931	No change
1981	ENSMODG00000021076	URB1	No change
1856	ENSMODG00000007615	RPS6KB2	No change
1699	ENSMODG00000005328	IL17RC	No change
503	ENSMODG00000014942	SLC35E1	No change
236	ENSMODG00000023930	TMEM229B	No change
212	ENSMODG00000014129	ENSMODT00000017990	No change
344	ENSMODG00000023755	SDHAF1	No change
2119	ENSMODG00000008489	LSS	No change
2004	ENSMODG00000019805	ENSMODT00000025157	No change

1867	ENSMODG00000003440	SPIRE2	No change
1124	ENSMODG00000024310	SMCO4	No change
1541	ENSMODG00000002076	SELENOT	No change
1761	ENSMODG00000021273	ENSMODT00000027068	No change
1605	ENSMODG00000016316	DLD	No change
915	ENSMODG00000019234	ENSMODT00000024434	No change
1639	ENSMODG00000018936	ANXA5	No change
1853	ENSMODG00000021428	AFG3L2	No change
1677	ENSMODG00000000598	APBA3	No change
976	ENSMODG00000015226	RAB35	No change
1915	ENSMODG00000016612	SREBF2	No change
894	ENSMODG00000001912	MYO5C	No change
624	ENSMODG00000009100	DNAJC1	No change
980	ENSMODG00000013513	APOPT1	No change
1115	ENSMODG00000009992	VTI1B	No change
981	ENSMODG00000009629	PEA15	No change
1559	ENSMODG00000005990	ACBD3	No change
1054	ENSMODG00000024111	GPR158	No change
525	ENSMODG00000004890	TMEM45B	No change
1365	ENSMODG00000010661	VPS26A	No change
1783	ENSMODG00000021589	ND6	No change
1734	ENSMODG00000016070	SKIL	No change
173	ENSMODG00000007357	EDEM3	No change
1312	ENSMODG00000013490	MED29	No change
1627	ENSMODG00000004068	SAMD12	No change
1769	ENSMODG00000003425	PTTG1IP	No change
1430	ENSMODG00000002873	NMNAT1	No change
1409	ENSMODG00000025570	LZIC	No change
749	ENSMODG00000013228	ZDHHC9	No change
395	ENSMODG00000009865	NPTN	No change
2137	ENSMODG00000004599	C16orf74	No change
1159	ENSMODG00000009622	ENSMODT00000012248	No change
272	ENSMODG00000027962	ENSMODT00000042314	No change
2149	ENSMODG00000000524	GGA1	No change
1801	ENSMODG00000019884	PEX16	No change
1435	ENSMODG00000017820	ZSCAN29	No change
2072	ENSMODG00000008403	RAB43	No change
1160	ENSMODG00000015284	ZNRF2	No change
301	ENSMODG00000012001	PCSK6	No change
123	ENSMODG00000000991	CLPTM1L	No change
1331	ENSMODG00000025484	ENSMODT00000039866	No change
1407	ENSMODG00000006618	CDIP1	No change
581	ENSMODG00000011490	APBB3	No change
1794	ENSMODG00000001063	TICAM1	No change
788	ENSMODG00000006289	ENSMODT00000007960	No change
164	ENSMODG00000027397	TMEM229A	No change
740	ENSMODG00000008586	PARVA	No change
461	ENSMODG00000013036	SLC25A17	No change
1024	ENSMODG00000016445	TFAP2C	No change
2093	ENSMODG00000008265	ENSMODT00000010469	No change
255	ENSMODG00000027607	TMEM241	No change
1767	ENSMODG00000016992	ENSMODT00000021571	No change

731	ENSMODG00000008117	RER1	No change
765	ENSMODG00000003626	HPGD	No change
1152	ENSMODG00000004700	SIKE1	No change
78	ENSMODG00000000405	ENSMODT00000000495	No change
19	ENSMODG00000010350	FAM84A	No change
1878	ENSMODG00000016560	ACO2	No change
853	ENSMODG00000000459	NAV1	No change
1508	ENSMODG00000015260	TUBA4A	No change
1842	ENSMODG00000019379	HDGFL3	No change
1649	ENSMODG00000011038	EPB41	No change
1811	ENSMODG00000001150	ENSMODT00000001395	No change
620	ENSMODG00000017555	QSOX2	No change
2146	ENSMODG00000029406	POP4	No change
2148	ENSMODG00000006622	ZNF410	No change
242	ENSMODG00000017851	CCNDBP1	No change
431	ENSMODG00000017853	TMEM62	No change
315	ENSMODG00000013329	SLC12A2	No change
1880	ENSMODG00000015378	ENSMODT00000019547	No change
2054	ENSMODG00000012996	ADAMTSL2	No change
1770	ENSMODG00000011952	UQCC3	No change
478	ENSMODG00000019126	RASL10B	No change
952	ENSMODG00000010697	SRRD	No change
996	ENSMODG00000010767	CDK16	No change
192	ENSMODG00000003000	ENSMODT00000003727	No change
912	ENSMODG00000018164	ATP6V1A	No change
1317	ENSMODG00000008568	IDH3G	No change
1429	ENSMODG00000008581	SSR4	No change
616	ENSMODG00000002274	SNTB1	No change
1445	ENSMODG00000019356	ENSMODT00000024580	No change
1684	ENSMODG00000003555	ENSMODT00000006516	No change
1431	ENSMODG00000005970	SLC27A4	No change
723	ENSMODG00000004078	ACER2	No change
1336	ENSMODG00000015604	MAT2A	No change
5	ENSMODG00000021320	NCEH1	No change
1566	ENSMODG00000001835	ENSMODT00000002285	No change
528	ENSMODG00000009336	GPR180	No change
1997	ENSMODG00000015055	COA3	No change
342	ENSMODG00000009997	APIP	No change
1007	ENSMODG00000010015	PDHX	No change
2009	ENSMODG00000005066	ESF1	No change
1807	ENSMODG00000005082	ENSMODT00000006386	No change
1499	ENSMODG00000019022	CNPY3	No change
689	ENSMODG00000001507	EDNRB	No change
847	ENSMODG00000020407	NPR3	No change
1616	ENSMODG00000023738	B3GALT4	No change
1231	ENSMODG00000007926	MBTPS2	No change
1423	ENSMODG00000005766	PPP6R2	No change
2037	ENSMODG00000013436	SNX2	No change
419	ENSMODG00000007747	SAT1	No change
425	ENSMODG00000003507	SLC8B1	No change
681	ENSMODG00000010743	TRIP4	No change
935	ENSMODG00000001021	FAM160A1	No change

675	ENSMODG00000004883	ENSMODT00000006142	No change
2097	ENSMODG00000023229	ENSMODT00000030580	No change
371	ENSMODG00000012975	SETD3	No change
1990	ENSMODG00000003570	ENSMODT00000004455	No change
1414	ENSMODG00000003712	SLC35A3	No change
2085	ENSMODG00000004823	C5orf22	No change
1372	ENSMODG00000009651	LIMK2	No change
193	ENSMODG00000008832	WNK2	No change
1590	ENSMODG00000014237	SLC16A7	No change
1980	ENSMODG00000019090	MOB1B	No change
491	ENSMODG00000005594	FPGS	No change
1397	ENSMODG00000019237	POLDIP2	No change
499	ENSMODG00000009956	CAT	No change
2057	ENSMODG00000006670	MIGA2	No change
1490	ENSMODG00000006913	ASNA1	No change
726	ENSMODG00000010196	POLB	No change
2166	ENSMODG00000010087	CYB561	No change
2077	ENSMODG00000019233	IFT20	No change
520	ENSMODG00000005346	NIT1	No change
659	ENSMODG00000003997	SUCO	No change
1354	ENSMODG00000007732	EEF2K	No change
1323	ENSMODG00000021095	CASK	No change
545	ENSMODG00000020418	GOLPH3	No change
764	ENSMODG00000003270	NANS	No change
372	ENSMODG00000001829	ALCAM	No change
939	ENSMODG00000019662	ENSMODT00000024965	No change
445	ENSMODG00000016709	OSBPL2	No change
951	ENSMODG00000018802	ELOVL5	No change
1932	ENSMODG00000002880	ENSMODT00000003575	No change
1655	ENSMODG00000024970	C12orf73	No change
1965	ENSMODG00000017430	BSDC1	No change
1583	ENSMODG00000006727	UBFD1	No change
1339	ENSMODG00000005229	B4GALT3	No change
1073	ENSMODG00000011284	POLE4	No change
2124	ENSMODG00000001242	RAVER2	No change
954	ENSMODG00000029106	CHCHD2	No change
449	ENSMODG00000021009	HLCS	No change
1668	ENSMODG00000001795	CDK18	No change
2114	ENSMODG00000007305	MIF4GD	No change
318	ENSMODG00000001084	UAP1	No change
233	ENSMODG00000010705	PLPP5	No change
224	ENSMODG00000006582	ENSMODT00000008327	No change
1259	ENSMODG00000023205	ENSMODT00000030482	No change
427	ENSMODG00000021002	DOPEY2	No change
1580	ENSMODG00000004845	ENSMODT00000038463	No change
1309	ENSMODG00000003620	VLDLR	No change
1165	ENSMODG00000012424	COPZ2	No change
1984	ENSMODG00000001481	SLAIN1	No change
1876	ENSMODG00000010450	ENSMODT00000013340	No change
1992	ENSMODG00000010191	ENSMODT00000012991	No change
1034	ENSMODG00000015417	PUM3	No change
1852	ENSMODG00000007018	MRPS25	No change

494	ENSMODG00000009860	CCDC22	No change
582	ENSMODG00000018829	PRUNE1	No change
579	ENSMODG00000000408	STEAP3	No change
585	ENSMODG00000003024	CLCN3	No change
1568	ENSMODG00000000098	NDUFC1	No change
1576	ENSMODG00000010504	ENSMODT00000013401	No change
562	ENSMODG00000006053	FAM20B	No change
1798	ENSMODG00000011771	ENSMODT00000015016	No change
405	ENSMODG00000010888	ENSMODT00000013883	No change
822	ENSMODG00000011632	ENSMODT00000014838	No change
1441	ENSMODG00000002150	TXNRD1	No change
1287	ENSMODG00000015746	CERK	No change
209	ENSMODG00000009003	ENSMODT00000011445	No change
567	ENSMODG00000019586	NDUFAF2	No change
1438	ENSMODG00000010681	HKDC1	No change
285	ENSMODG00000011662	C1orf27	No change
1442	ENSMODG00000012307	PGRMC2	No change
1613	ENSMODG00000018841	LYSMD1	No change
1533	ENSMODG00000004547	LRCH4	No change
205	ENSMODG00000004565	FBXO24	No change
130	ENSMODG00000013383	ENSMODT00000017045	No change
1644	ENSMODG00000012773	ENSMODT00000016276	No change
785	ENSMODG00000024536	ENSMODT00000036104	No change
435	ENSMODG00000003093	TDRD7	No change
1795	ENSMODG00000017751	ENSMODT00000022522	No change
277	ENSMODG00000007596	ENSMODT00000009685	No change
519	ENSMODG00000011103	DSC2	No change
407	ENSMODG00000016597	PRADC1	No change
517	ENSMODG00000005316	HSDL2	No change
1771	ENSMODG00000021523	ENSMODT00000027373	No change
1393	ENSMODG00000009305	ENSMODT00000011846	No change
963	ENSMODG00000016382	ADCY9	No change
325	ENSMODG00000015505	EHD3	No change
883	ENSMODG00000016465	IPPK	No change
834	ENSMODG00000007415	SLCO5A1	No change
2038	ENSMODG00000021041	SOD1	No change
1373	ENSMODG00000016720	ATP5C1	No change
961	ENSMODG00000014930	GPALPP1	No change
762	ENSMODG00000009024	ASCC2	No change
1299	ENSMODG00000009005	ATOX1	No change
1383	ENSMODG00000017880	ADPRHL2	No change
1203	ENSMODG00000012115	ETF1	No change
905	ENSMODG00000023370	RNF183	No change
1146	ENSMODG00000011211	WBP1L	No change
1302	ENSMODG00000004961	MOGS	No change
1913	ENSMODG00000015819	ACADL	No change
2183	ENSMODG00000027819	ENSMODT00000043176	No change
1264	ENSMODG00000002258	TMEM192	No change
222	ENSMODG00000005781	ABAT	No change
1179	ENSMODG00000020582	CYB5D2	No change
2075	ENSMODG00000021059	CRYZL1	No change
1028	ENSMODG00000003928	BMP2	No change

1456	ENSMODG00000016019	NDUFS1	No change
617	ENSMODG00000003299	ENSMODT00000004115	No change
804	ENSMODG00000004040	IGHMBP2	No change
742	ENSMODG00000018012	SEC63	No change
722	ENSMODG00000004418	WIPI1	No change
74	ENSMODG00000015563	TMEM169	No change
757	ENSMODG00000015562	ENSMODT00000019772	No change
102	ENSMODG00000015568	ENSMODT00000019777	No change
1634	ENSMODG00000009870	GPHN	No change
1091	ENSMODG00000011252	ENSMODT00000014436	No change
159	ENSMODG00000011336	ENSMODT00000014439	No change
197	ENSMODG00000011489	ENSMODT00000014636	No change
1859	ENSMODG00000009183	TMEM68	No change
606	ENSMODG00000011967	ENSMODT00000015271	No change
1217	ENSMODG00000011303	CLCN5	No change
2020	ENSMODG00000004778	PEMT	No change
198	ENSMODG00000010043	ALG5	No change
2147	ENSMODG00000000839	NRDC	No change
2158	ENSMODG00000004722	LSM7	No change
401	ENSMODG00000000209	TBC1D9	No change
1337	ENSMODG00000008134	ENSMODT00000010307	No change
200	ENSMODG00000016011	DNAJB9	No change
1117	ENSMODG00000024563	THAP5	No change
863	ENSMODG00000000608	ENSMODT00000000744	No change
1741	ENSMODG00000014684	TSPAN33	No change
1961	ENSMODG00000006802	TSPO	No change
1082	ENSMODG00000012618	TEX10	No change
1703	ENSMODG00000001031	GATB	No change
1998	ENSMODG00000021293	DNAJC19	No change
578	ENSMODG00000004366	LIMA1	No change
69	ENSMODG00000015711	SLC5A6	No change
537	ENSMODG00000013465	HERPUD1	No change
1696	ENSMODG00000004795	ENSMODT00000006030	No change
1068	ENSMODG00000001197	COA5	No change
649	ENSMODG00000001209	UNC50	No change
61	ENSMODG00000001179	ENSMODT00000039577	No change
88	ENSMODG00000002966	HNF1A	No change
1947	ENSMODG00000018132	GPR63	No change
1413	ENSMODG00000015913	SCLY	No change
1989	ENSMODG00000000141	NDUFAF1	No change
782	ENSMODG00000009380	ATF4	No change
972	ENSMODG00000021328	NDUFS8	No change
571	ENSMODG00000025788	AKTIP	No change
650	ENSMODG00000005393	SLC12A4	No change
2178	ENSMODG00000009315	ENSMODT00000011858	No change
559	ENSMODG00000011675	ALAS1	No change
2129	ENSMODG00000015649	ZNF513	No change
1625	ENSMODG00000015661	SNX17	No change
1789	ENSMODG00000014995	PNPLA2	No change
840	ENSMODG00000025756	DPM2	No change
1428	ENSMODG00000017909	CAPN3	No change
1370	ENSMODG00000007169	OSBPL5	No change

1986	ENSMODG00000001220	ENSMODT00000001495	No change
2019	ENSMODG00000011421	RHPN1	No change
1752	ENSMODG00000013621	ENSMODT00000017355	No change
1334	ENSMODG00000018535	ENSMODT00000023527	No change
1233	ENSMODG00000022599	ENSMODT00000028461	No change
1910	ENSMODG00000016821	NAXE	No change
1628	ENSMODG00000017349	RABL6	No change
238	ENSMODG00000002844	ENSMODT00000003531	No change
1492	ENSMODG00000020687	ENSMODT00000026342	No change
76	ENSMODG00000019585	ELOVL7	No change
456	ENSMODG00000013739	PCSK7	No change
1532	ENSMODG00000023241	ENSMODT00000030631	No change
97	ENSMODG00000018386	PGM3	No change
933	ENSMODG00000003168	COX6A1	No change
417	ENSMODG00000015809	HADHB	No change
866	ENSMODG00000015815	HADHA	No change
842	ENSMODG00000000216	ENSMODT00000000260	No change
529	ENSMODG00000001846	ENSMODT00000002300	No change
1710	ENSMODG00000003266	CHMP4A	No change
774	ENSMODG00000010676	XPNPEP1	No change
1273	ENSMODG00000003354	OMA1	No change
587	ENSMODG00000025527	ENSMODT00000039915	No change
2003	ENSMODG00000012117	RHOF	No change
514	ENSMODG00000018599	COPB2	No change
1603	ENSMODG00000007869	GANAB	No change
596	ENSMODG00000006951	CHRNB1	No change
1693	ENSMODG00000011577	VPS29	No change
1099	ENSMODG00000001966	NMRK1	No change
795	ENSMODG00000021235	GK5	No change
460	ENSMODG00000007864	TPRA1	No change
1065	ENSMODG00000020697	SRP72	No change
1362	ENSMODG00000018930	SLC35B2	No change
1521	ENSMODG00000018288	ILDR1	No change
256	ENSMODG00000007982	SEC61A1	No change
1536	ENSMODG00000015313	ABCB6	No change
215	ENSMODG00000009530	CD8B	No change
1812	ENSMODG00000009685	ENSMODT00000012334	No change
269	ENSMODG00000003588	DBT	No change
1520	ENSMODG00000000968	PIGF	No change
2027	ENSMODG00000008997	UBL4A	No change
852	ENSMODG00000013770	PAX8	No change
1890	ENSMODG00000019089	ACADM	No change
104	ENSMODG00000003308	HOOK1	No change
639	ENSMODG00000007058	CARS	No change
98	ENSMODG00000018810	GSTA4	No change
1419	ENSMODG00000005094	CAPN2	No change
1954	ENSMODG00000012212	GLRX2	No change
1883	ENSMODG00000002472	PDCD6IP	No change
1647	ENSMODG00000001268	C19orf70	No change
950	ENSMODG00000004595	FOXRED1	No change
1949	ENSMODG00000025062	RNF152	No change
1263	ENSMODG00000018587	SMPD1	No change

1443	ENSMODG00000017915	GANC	No change
359	ENSMODG00000017917	ENSMODT00000022728	No change
941	ENSMODG00000019289	SDF2	No change
1558	ENSMODG00000011066	MECR	No change
1985	ENSMODG00000007116	NUP50	No change
920	ENSMODG00000014976	EVI5L	No change
1157	ENSMODG00000012240	RHPN2	No change
1116	ENSMODG00000018110	COPS7A	No change
662	ENSMODG00000014227	SAR1B	No change
1278	ENSMODG00000015474	SLC30A6	No change
223	ENSMODG00000005007	TXNDC11	No change
995	ENSMODG00000010579	DUSP11	No change
1439	ENSMODG00000019222	SPTLC1	No change
85	ENSMODG00000009729	ST8SIA6	No change
1582	ENSMODG00000012312	RBM6	No change
1637	ENSMODG00000001490	ENSMODT00000001852	No change
116	ENSMODG00000000241	AQP5	No change
504	ENSMODG00000009980	SPART	No change
653	ENSMODG00000015969	ENSMODT00000020285	No change
1279	ENSMODG00000010022	TXNDC5	No change
985	ENSMODG00000015131	MPHOSPH6	No change
1866	ENSMODG00000004047	STAM2	No change
1574	ENSMODG00000021031	ENSMODT00000026768	No change
1346	ENSMODG00000017583	SEC16A	No change
741	ENSMODG00000001012	ENSMODT00000001231	No change
1481	ENSMODG00000016770	ECHDC3	No change
1137	ENSMODG00000009257	CLDN10	No change
464	ENSMODG00000002983	SPPL3	No change
1103	ENSMODG00000012053	SEC31A	No change
1404	ENSMODG00000001243	FAF1	No change
1834	ENSMODG00000016904	ENSMODT00000021468	No change
2045	ENSMODG00000015725	CGREF1	No change
413	ENSMODG00000003102	ENSMODT00000003865	No change
1206	ENSMODG00000015026	ENSMODT00000019128	No change
361	ENSMODG00000012091	ORAI1	No change
1315	ENSMODG00000009273	SCAMP2	No change
1458	ENSMODG00000013505	RSPRY1	No change
310	ENSMODG00000012525	EIF2B1	No change
1256	ENSMODG00000012393	TIMM21	No change
118	ENSMODG00000005312	SERPINB12	No change
1964	ENSMODG00000016939	CHMP2B	No change
1635	ENSMODG00000014160	ELP6	No change
248	ENSMODG00000004601	TIRAP	No change
1624	ENSMODG00000025472	ENSMODT00000039849	No change
1675	ENSMODG00000017106	ENSMODT00000021719	No change
33	ENSMODG00000018800	GCLC	No change
1462	ENSMODG00000013511	ARL2BP	No change
1248	ENSMODG00000019642	ADAMTS6	No change
1586	ENSMODG00000017806	TP53BP1	No change
2177	ENSMODG00000022724	MFSD5	No change
406	ENSMODG00000012740	DEDD2	No change
1292	ENSMODG00000005957	LARP4B	No change

1511	ENSMODG00000000602	MRPL54	No change
1702	ENSMODG00000015041	OCEL1	No change
880	ENSMODG00000011116	SHROOM4	No change
1254	ENSMODG00000000546	CDC42EP1	No change
501	ENSMODG00000017970	NECAP1	No change
975	ENSMODG00000025664	SRP9	No change
753	ENSMODG00000013527	CDKN1A	No change
1872	ENSMODG00000004957	WDSUB1	No change
901	ENSMODG00000002664	TP53INP2	No change
1239	ENSMODG00000019452	FURIN	No change
623	ENSMODG00000001631	ENSMODT00000002037	No change
564	ENSMODG00000007003	ENSMODT00000008855	No change
1366	ENSMODG00000008685	SEC23IP	No change
570	ENSMODG00000013697	CTSA	No change
45	ENSMODG00000007754	STARD10	No change
781	ENSMODG00000020741	KLHL8	No change
629	ENSMODG00000008182	HMCES	No change
877	ENSMODG00000019914	ENSMODT00000025287	No change
1584	ENSMODG00000019916	NDUFS3	No change
527	ENSMODG00000016574	PMM1	No change
2021	ENSMODG00000024824	GPR1	No change
809	ENSMODG00000006927	DOHH	No change
262	ENSMODG00000017698	SDF2L1	No change
1473	ENSMODG00000001998	ENSMODT00000002488	No change
690	ENSMODG00000013630	ENSMODT00000017366	No change
1526	ENSMODG00000015949	FZD5	No change
311	ENSMODG00000004641	KIAA0513	No change
210	ENSMODG00000019500	REM1	No change
180	ENSMODG00000018730	TMEM184B	No change
766	ENSMODG00000015140	IMPDH1	No change
2100	ENSMODG00000020574	TMEM33	No change
1545	ENSMODG00000000501	GALNT1	No change
1350	ENSMODG00000013493	TCERG1L	No change
938	ENSMODG00000011943	ENSMODT00000015240	No change
910	ENSMODG00000018206	COX17	No change
278	ENSMODG00000006858	ENSMODT00000008666	No change
1755	ENSMODG00000006867	NAT9	No change
458	ENSMODG00000003028	IGFBPL1	No change
835	ENSMODG00000013008	ENSMODT00000016569	No change
1791	ENSMODG00000020720	RAP1GDS1	No change
428	ENSMODG00000025753	ENSMODT00000040200	No change
720	ENSMODG00000025754	ENSMODT00000040201	No change
997	ENSMODG00000000049	ENSMODT00000000051	No change
1170	ENSMODG00000012227	SH2B3	No change
770	ENSMODG00000015892	ECI1	No change
1714	ENSMODG00000014792	RAB5C	No change
1166	ENSMODG00000002868	ERP29	No change
2049	ENSMODG00000013475	UQCRC1	No change
1869	ENSMODG00000009484	UBL7	No change
232	ENSMODG00000016437	ENSMODT00000020895	No change
1790	ENSMODG00000005844	ENSMODT00000007377	No change
709	ENSMODG00000011054	SLC47A1	No change

1178	ENSMODG00000015269	PRKAB1	No change
911	ENSMODG00000000314	RPN2	No change
28	ENSMODG00000016494	PAPSS2	No change
1818	ENSMODG00000006410	TSG101	No change
114	ENSMODG00000015149	NEU1	No change
1758	ENSMODG00000018578	ENSMODT00000023588	No change
1329	ENSMODG00000024930	CHCHD7	No change
1186	ENSMODG00000019141	RAD51D	No change
2153	ENSMODG00000014723	NT5C3B	No change
1066	ENSMODG00000000879	QDPR	No change
14	ENSMODG00000023829	PADI1	No change
24	ENSMODG00000006107	ENSMODT00000007720	No change
17	ENSMODG00000006778	ENSMODT00000008569	No change
213	ENSMODG00000007768	ACOT9	No change
1307	ENSMODG00000011827	TEX264	No change
808	ENSMODG00000008305	CLINT1	No change
1295	ENSMODG00000014107	NDUFA4	No change
1012	ENSMODG00000004994	THOP1	No change
1164	ENSMODG00000019473	ESM1	No change
1230	ENSMODG00000002382	PRDX1	No change
1356	ENSMODG00000003845	ENSMODT00000039414	No change
891	ENSMODG00000005518	TOR2A	No change
1658	ENSMODG00000000529	SCP2	No change
1838	ENSMODG00000020226	TMEM161B	No change
1027	ENSMODG00000025418	ENSMODT00000039706	No change
1128	ENSMODG00000018705	SEC22B	No change
603	ENSMODG00000013388	CAPN12	No change
913	ENSMODG00000001837	BCKDK	No change
465	ENSMODG00000021350	EEF1AKMT3	No change
2040	ENSMODG00000019002	MEA1	No change
1135	ENSMODG00000019007	PPP2R5D	No change
1344	ENSMODG00000000326	GTPBP6	No change
577	ENSMODG00000015948	EPHX2	No change
290	ENSMODG00000025518	TMEM40	No change
1076	ENSMODG00000019208	USO1	No change
83	ENSMODG00000028788	ENSMODT00000042151	No change
630	ENSMODG00000006431	EXOC6	No change
1768	ENSMODG00000000891	AP5Z1	No change
1053	ENSMODG00000006429	ALDH6A1	No change
264	ENSMODG00000013560	TMPRSS4	No change
1433	ENSMODG00000017916	KIAA0319L	No change
1849	ENSMODG00000000212	SPINT1	No change
412	ENSMODG00000019231	NLK	No change
1411	ENSMODG00000006698	UVRAG	No change
627	ENSMODG00000004414	ENSMODT00000005553	No change
1005	ENSMODG00000015827	ENSMODT00000020100	No change
1175	ENSMODG00000020570	LIMCH1	No change
1408	ENSMODG00000001387	EIF5B	No change
784	ENSMODG00000007605	ITGB4	No change
4	ENSMODG00000025044	BHLHA15	No change
812	ENSMODG00000000399	RPRD1A	No change
1572	ENSMODG00000019219	SDAD1	No change

228	ENSMODG00000015696	ENSMODT00000019938	No change
161	ENSMODG00000010648	INTS6	No change
121	ENSMODG00000018157	SIDT1	No change
948	ENSMODG00000013760	SIDT2	No change
2061	ENSMODG00000021011	ATP5J	No change
1088	ENSMODG00000017988	ENSMODT00000022824	No change
1114	ENSMODG00000008789	FAM120A	No change
1026	ENSMODG00000011335	HARS	No change
1633	ENSMODG00000005371	EMC3	No change
1013	ENSMODG00000015898	ENSMODT00000020189	No change
312	ENSMODG00000007301	PANK1	No change
1436	ENSMODG00000001470	CMPK1	No change
2050	ENSMODG00000010232	UPF3B	No change
1645	ENSMODG00000011519	FARSA	No change
1919	ENSMODG00000009282	ULK3	No change
176	ENSMODG00000001868	TMEM65	No change
1941	ENSMODG00000021514	AQP4	No change
998	ENSMODG00000005649	ST6GALNAC6	No change
2133	ENSMODG00000023396	SCAF1	No change
748	ENSMODG00000024098	ENSMODT00000034611	No change
859	ENSMODG00000013804	ENSMODT00000017575	No change
1199	ENSMODG00000024451	ENSMODT00000035796	No change
565	ENSMODG00000004672	DUSP10	No change
1534	ENSMODG00000005059	KIAA1468	No change
1707	ENSMODG00000007937	CLPB	No change
1840	ENSMODG00000019010	DCTN6	No change
1705	ENSMODG00000013951	SYF2	No change
1142	ENSMODG00000014961	NGLY1	No change
1080	ENSMODG00000018967	YIPF3	No change
1993	ENSMODG00000025616	ENSMODT00000040026	No change
1048	ENSMODG00000003196	EIF2B5	No change
1108	ENSMODG00000006819	SCNN1B	No change
641	ENSMODG00000014773	DYNLL2	No change
2172	ENSMODG00000013603	IRF3	No change
696	ENSMODG00000018329	PRMT8	No change
1202	ENSMODG00000002114	ENSMODT00000002630	No change
914	ENSMODG00000015658	ENSMODT00000019893	No change
353	ENSMODG00000015962	ENSMODT00000020277	No change
876	ENSMODG00000011073	TTI2	No change
303	ENSMODG00000011688	DNAJA4	No change
526	ENSMODG00000025076	CLDN8	No change
289	ENSMODG00000015612	ATIC	No change
1158	ENSMODG00000008735	ADAM10	No change
857	ENSMODG00000022965	SEC22C	No change
106	ENSMODG00000014400	ENSMODT00000018330	No change
467	ENSMODG00000017648	PI4KA	No change
1592	ENSMODG00000017641	SNAP29	No change
1617	ENSMODG00000028727	ENSMODT00000042007	No change
727	ENSMODG00000002206	TDG	No change
2142	ENSMODG00000008015	TTC1	No change
355	ENSMODG00000000867	CLRN2	No change
1643	ENSMODG00000006583	ENSMODT00000008329	No change

558	ENSMODG00000015693	CAD	No change
1906	ENSMODG00000008037	COX10	No change
1352	ENSMODG00000004926	NUDCD1	No change
1665	ENSMODG00000020996	LTN1	No change
1809	ENSMODG00000013491	UBE4A	No change
1047	ENSMODG00000021035	CSTB	No change
1552	ENSMODG00000019834	COMMD9	No change
201	ENSMODG00000020701	ENSMODT00000026357	No change
1787	ENSMODG00000014194	ARHGEF10	No change
308	ENSMODG00000013984	PDLIM4	No change
1944	ENSMODG00000015503	ENSMODT00000019698	No change
1405	ENSMODG00000019239	TMEM199	No change
1121	ENSMODG00000001693	GHITM	No change
580	ENSMODG00000000549	PPARGC1A	No change
642	ENSMODG00000028712	ENSMODT00000041954	No change
712	ENSMODG00000021111	XK	No change
2091	ENSMODG00000010461	CYC1	No change
734	ENSMODG00000003919	FERMT1	No change
1437	ENSMODG00000018581	PIK3CB	No change
613	ENSMODG00000018156	PTPN6	No change
573	ENSMODG00000010070	MRPL40	No change
1485	ENSMODG00000015607	VAMP8	No change
100	ENSMODG00000003771	HOMER3	No change
1766	ENSMODG00000027876	ENSMODT00000043875	No change
1931	ENSMODG00000016793	HAGHL	No change
1390	ENSMODG00000007576	MRPL48	No change
1037	ENSMODG00000010565	MROH1	No change
254	ENSMODG00000024924	ENSMODT00000037821	No change
149	ENSMODG00000025346	ENSMODT00000039386	No change
1972	ENSMODG00000001310	MAN2A1	No change
918	ENSMODG00000005282	UFC1	No change
2041	ENSMODG00000014661	PLIN2	No change
1750	ENSMODG00000018130	GTPBP8	No change
983	ENSMODG00000006519	PHYHD1	No change
1581	ENSMODG00000008964	ENSMODT00000011392	No change
1614	ENSMODG00000018456	BCL2L13	No change
263	ENSMODG00000001956	TMEM144	No change
1749	ENSMODG00000011653	TDP2	No change
621	ENSMODG00000025622	ACOT13	No change
163	ENSMODG00000017923	TMEM87A	No change
885	ENSMODG00000006373	ENSMODT00000008067	No change
1907	ENSMODG00000005770	ENSMODT00000007290	No change
1156	ENSMODG00000025070	ENSMODT00000022910	No change
549	ENSMODG00000017065	CPOX	No change
1516	ENSMODG00000000723	OSBPL3	No change
1955	ENSMODG00000006265	MKRN2OS	No change
2116	ENSMODG00000000470	ENSMODT00000000573	No change
2139	ENSMODG00000006407	ZDHHC12	No change
420	ENSMODG00000001351	ATP5F1	No change
1698	ENSMODG00000013897	UBE2D4	No change
1600	ENSMODG00000027736	ENSMODT00000043028	No change
899	ENSMODG00000002896	SFT2D2	No change

1401	ENSMODG00000001933	GNAT2	No change
391	ENSMODG00000015399	SLC1A1	No change
1058	ENSMODG00000017127	ZBTB7B	No change
816	ENSMODG00000017212	TMEM27	No change
1630	ENSMODG00000019501	HDDC3	No change
1500	ENSMODG00000009405	ENSMODT00000011971	No change
1596	ENSMODG00000015384	NDUFA5	No change
1948	ENSMODG00000025600	ENSMODT00000040010	No change
575	ENSMODG00000020289	ENSMODT00000025830	No change
231	ENSMODG00000023128	FBXO48	No change
1510	ENSMODG00000003083	FLVCR1	No change
1119	ENSMODG00000000256	CHPF	No change
1759	ENSMODG00000003937	ABCB8	No change
1097	ENSMODG00000010134	ZFPL1	No change
574	ENSMODG00000016735	GATA3	No change
2144	ENSMODG00000014823	VWA8	No change
2055	ENSMODG00000000346	SEC24C	No change
2076	ENSMODG00000000573	TOP1	No change
668	ENSMODG00000015263	GOLGA3	No change
1049	ENSMODG00000001963	DMBX1	No change
348	ENSMODG00000028847	SH3BGRL2	No change
1131	ENSMODG00000010030	ENSMODT00000012790	No change
1112	ENSMODG00000019422	GTSF1	No change
1010	ENSMODG00000006296	CPTP	No change
306	ENSMODG00000005424	THYN1	No change
1920	ENSMODG00000006810	TOR1A	No change
903	ENSMODG00000002406	FAM3D	No change
354	ENSMODG00000028962	ENSMODT00000042325	No change
819	ENSMODG00000015682	TBX6	No change
455	ENSMODG00000000527	RNPEP	No change
250	ENSMODG00000020306	PTGER4	No change
115	ENSMODG00000007557	NRIP3	No change
137	ENSMODG00000021272	SERP1	No change
226	ENSMODG00000014153	SLC35B4	No change
153	ENSMODG00000003669	ENSMODT00000004593	No change
1284	ENSMODG00000015367	HEATR5B	No change
628	ENSMODG00000009925	TFE3	No change
350	ENSMODG00000020688	LAMTOR3	No change
2108	ENSMODG00000011210	MED4	No change
1363	ENSMODG00000002334	KLHL2	No change
2017	ENSMODG00000009520	ENSMODT00000012117	No change
390	ENSMODG00000020599	OSTC	No change
1167	ENSMODG00000007734	EXOC5	No change
2014	ENSMODG00000003084	GTDC1	No change
692	ENSMODG00000009053	NPAS1	No change
158	ENSMODG00000006220	CHMP4C	No change
979	ENSMODG00000013269	RAB33A	No change
906	ENSMODG00000013790	ENSMODT00000017562	No change
2016	ENSMODG00000013793	MRPL55	No change
930	ENSMODG00000021216	ENSMODT00000026992	No change
1361	ENSMODG00000027492	ENSMODT00000043170	No change
1482	ENSMODG00000027691	ENSMODT00000042400	No change

442	ENSMODG00000027774	ENSMODT00000042051	No change
67	ENSMODG00000021329	CHKA	No change
133	ENSMODG00000011124	DSC3	No change
2065	ENSMODG00000005024	ZNHIT1	No change
1810	ENSMODG00000006588	ENSMODT00000008338	No change
1924	ENSMODG00000016745	STUB1	No change
638	ENSMODG00000016748	JMJD8	No change
836	ENSMODG00000017788	PPIP5K1	No change
477	ENSMODG00000024926	ENSMODT00000004216	No change
162	ENSMODG00000011370	GBF1	No change
953	ENSMODG00000000158	CHP1	No change
806	ENSMODG00000000421	CTBS	No change
2175	ENSMODG00000018708	PDE4DIP	No change
1008	ENSMODG00000003687	ENSMODT00000004616	No change
1551	ENSMODG00000017634	SPATA5L1	No change
1971	ENSMODG00000005314	ATP5D	No change
270	ENSMODG00000010528	WDFY2	No change
926	ENSMODG00000002312	RBM41	No change
2094	ENSMODG00000008369	SGCG	No change
1925	ENSMODG00000021323	FNDC3B	No change
1776	ENSMODG00000001404	MAP2K3	No change
698	ENSMODG00000019346	PAFAH1B3	No change
1388	ENSMODG00000018415	WNT5B	No change
2162	ENSMODG00000008913	GXYLT1	No change
1399	ENSMODG00000004055	ENSMODT00000005095	No change
1870	ENSMODG00000025017	ENSMODT00000038186	No change
1897	ENSMODG00000017991	ENSMODT00000022827	No change
1002	ENSMODG00000017103	KRTCAP2	No change
136	ENSMODG00000016705	HRH3	No change
1777	ENSMODG00000015070	ENSMODT00000019179	No change
609	ENSMODG00000011328	HK2	No change
1281	ENSMODG00000012545	ATP6V0A2	No change
605	ENSMODG00000021357	YIPF1	No change
204	ENSMODG00000027768	CREG1	No change
1394	ENSMODG00000002176	ENSMODT00000002702	No change
50	ENSMODG00000013086	NIPAL3	No change
1328	ENSMODG00000027679	ENSMODT00000043947	No change
2096	ENSMODG00000018855	MUT	No change
556	ENSMODG00000003995	CPT1A	No change
246	ENSMODG00000023825	DIO3	No change
2066	ENSMODG00000007497	ACTN2	No change
645	ENSMODG00000006646	SLC2A4	No change
851	ENSMODG00000010390	PDCD4	No change
1461	ENSMODG00000010572	CWF19L1	No change
1938	ENSMODG00000028495	ENSMODT00000042442	No change
1228	ENSMODG00000028179	ENSMODT00000042595	No change
1900	ENSMODG00000001665	EMC4	No change
1744	ENSMODG00000003127	ENSMODT00000003898	No change
884	ENSMODG00000019248	SLC46A1	No change
36	ENSMODG00000002506	IGF1	No change
988	ENSMODG00000006356	SCCPDH	No change
1138	ENSMODG00000010176	SLC20A2	No change

336	ENSMODG00000021538	QPCTL	No change
1043	ENSMODG00000015674	GDPD3	No change
1793	ENSMODG00000007717	SLC25A3	No change
875	ENSMODG00000001089	BTG2	No change
113	ENSMODG00000029263	VOPP1	No change
1050	ENSMODG00000010719	ADCK5	No change
1358	ENSMODG00000020265	EMB	No change
358	ENSMODG00000010259	TBC1D25	No change
1325	ENSMODG00000017696	ENSMODT00000022449	No change
1650	ENSMODG00000023573	ENSMODT00000032079	No change
830	ENSMODG00000009643	EIF2AK3	No change
1848	ENSMODG00000012300	ENSMODT00000015692	No change
1132	ENSMODG00000006632	SH3GLB2	No change
801	ENSMODG00000000512	ENSMODT00000000626	No change
1843	ENSMODG00000004397	GLTP	No change
1403	ENSMODG00000003296	ENSMODT00000004114	No change
855	ENSMODG00000016501	ENSMODT00000020964	No change
1638	ENSMODG00000027379	ENSMODT00000044158	No change
1070	ENSMODG00000002154	SARS	No change
38	ENSMODG00000014088	SULT2B1	No change
1211	ENSMODG00000021267	COMMD2	No change
79	ENSMODG00000014301	RNFT1	No change
1171	ENSMODG00000028882	ENSMODT00000042620	No change
1694	ENSMODG00000007135	NARF	No change
1929	ENSMODG00000008558	ENSMODT00000010850	No change
1685	ENSMODG00000024156	MRPL57	No change
1015	ENSMODG00000002658	ABHD17B	No change
196	ENSMODG00000025629	YIPF2	No change
1338	ENSMODG00000028657	TIMM29	No change
902	ENSMODG00000003822	AP1G2	No change
203	ENSMODG00000008097	STX5	No change
945	ENSMODG00000005949	TMED10	No change
1039	ENSMODG00000003692	PYROXD2	No change
1979	ENSMODG00000018744	PDZK1	No change
682	ENSMODG00000009108	SLC1A5	No change
838	ENSMODG00000004290	TMEM129	No change
2047	ENSMODG00000027860	C2CD4D	No change
1193	ENSMODG00000016842	YTHDF1	No change
1122	ENSMODG00000011455	OSGEPL1	No change
1569	ENSMODG00000009191	COG2	No change
1304	ENSMODG00000028377	ENSMODT00000042224	No change
383	ENSMODG00000005444	ENSMODT00000006862	No change
643	ENSMODG00000001011	MCFD2	No change
710	ENSMODG00000007774	METTL9	No change
450	ENSMODG00000012640	ERP44	No change
1861	ENSMODG00000012645	STX17	No change
1120	ENSMODG00000018265	ENSMODT00000023178	No change
1345	ENSMODG00000021439	NAPG	No change
829	ENSMODG00000008052	ENSMODT00000010211	No change
2112	ENSMODG00000018116	COQ3	No change
544	ENSMODG00000019196	ADAP2	No change
1722	ENSMODG00000023233	FAM212B	No change

1333	ENSMODG00000008482	MIPEP	No change
2165	ENSMODG00000013404	SCN10A	No change
1833	ENSMODG00000002978	EXOSC3	No change
1716	ENSMODG00000021057	ENSMODT00000026798	No change
484	ENSMODG00000027659	ENSMODT00000043933	No change
927	ENSMODG00000025569	ENSMODT00000039965	No change
430	ENSMODG00000020865	PVR	No change
493	ENSMODG00000014324	ENSMODT00000018230	No change
1412	ENSMODG00000005934	TMEM63A	No change
1369	ENSMODG00000014505	GNPNAT1	No change
2115	ENSMODG00000018261	CD69	No change
1213	ENSMODG00000007015	NUCB2	No change
1860	ENSMODG00000003953	ENSMODT00000004959	No change
362	ENSMODG00000010950	CAB39L	No change
1301	ENSMODG00000006085	METTL3	No change
2073	ENSMODG00000016381	ATP9A	No change
870	ENSMODG00000004436	ENSMODT00000005583	No change
189	ENSMODG00000024159	GJB2	No change
684	ENSMODG00000007459	MTR	No change
1347	ENSMODG00000011304	RNF144B	No change
1229	ENSMODG00000012495	SELENOK	No change
732	ENSMODG00000024655	TPMT	No change
1642	ENSMODG00000005790	ATG4D	No change
589	ENSMODG00000004596	ENSMODT00000005782	No change
181	ENSMODG00000003782	SPCS3	No change
482	ENSMODG00000003483	FKBP8	No change
2030	ENSMODG00000004302	LAMTOR4	No change
542	ENSMODG00000004355	C7orf43	No change
676	ENSMODG00000021113	PRRG1	No change
1885	ENSMODG00000015000	BZW1	No change
1518	ENSMODG00000005864	AZIN1	No change
1618	ENSMODG00000009069	NUDT1	No change
144	ENSMODG00000007690	OSGIN2	No change
1884	ENSMODG00000025007	C8orf59	No change
547	ENSMODG00000000088	MCU	No change
490	ENSMODG00000020781	CDS1	No change
811	ENSMODG00000008824	FAR1	No change
1708	ENSMODG00000017373	RNF19B	No change
658	ENSMODG00000016708	MCRIP2	No change
1219	ENSMODG00000010127	VPS53	No change
2107	ENSMODG00000009895	ENSMODT00000012612	No change
186	ENSMODG00000009263	ENSMODT00000011791	No change
1148	ENSMODG00000023865	ENSMODT00000033574	No change
179	ENSMODG00000019338	ENSMODT00000024560	No change
733	ENSMODG00000020089	RHOBTB3	No change
1450	ENSMODG00000016470	BICDL2	No change
91	ENSMODG00000017474	ENSMODT00000022185	No change
1449	ENSMODG00000005399	VIPAS39	No change
882	ENSMODG00000000957	SLC36A4	No change
99	ENSMODG00000001284	LRAT	No change
841	ENSMODG00000006271	IMPA1	No change
1395	ENSMODG00000015767	SLC35F6	No change

871	ENSMODG00000029080	ENSMODT00000043432	No change
1945	ENSMODG00000012414	GTF2B	No change
368	ENSMODG00000005925	ENSMODT00000007475	No change
1348	ENSMODG00000003286	ENSMODT00000004103	No change
1125	ENSMODG00000019919	MTCH2	No change
1222	ENSMODG00000015747	SSR3	No change
685	ENSMODG00000019741	NEK6	No change
1240	ENSMODG00000020995	RWDD2B	No change
919	ENSMODG00000020994	ENSMODT00000026726	No change
1689	ENSMODG00000001558	PPP1R15B	No change
775	ENSMODG00000016959	LAMTOR2	No change
1221	ENSMODG00000013496	C6orf89	No change
2013	ENSMODG00000008834	SPRTN	No change
751	ENSMODG00000015479	SPAST	No change
1724	ENSMODG00000019147	LIG3	No change
548	ENSMODG00000005505	SEC13	No change
453	ENSMODG00000008995	NIPSNAP1	No change
68	ENSMODG00000021034	PDXK	No change
2132	ENSMODG00000028315	ENSMODT00000042977	No change
773	ENSMODG00000018000	MFAP5	No change
2051	ENSMODG00000004390	MRPS14	No change
1209	ENSMODG00000004383	CACYBP	No change
1045	ENSMODG00000016651	ENSMODT00000021147	No change
958	ENSMODG00000004372	COLGALT1	No change
252	ENSMODG00000015438	CADPS2	No change
693	ENSMODG00000009217	SPHK1	No change
1678	ENSMODG00000004016	GUCD1	No change
1969	ENSMODG00000020504	TOLLIP	No change
437	ENSMODG00000024112	TMEM134	No change
1021	ENSMODG00000004594	PIK3AP1	No change
2084	ENSMODG00000001465	EPHA1	No change
1962	ENSMODG00000014397	H2AFY	No change
332	ENSMODG00000005131	SLC11A2	No change
923	ENSMODG00000011648	TWF2	No change
1742	ENSMODG00000005417	VPS26B	No change
1381	ENSMODG00000016192	GNPTG	No change
374	ENSMODG00000018269	SLC15A2	No change
1484	ENSMODG00000029412	ENSMODT00000042672	No change
2026	ENSMODG00000009139	POMP	No change
381	ENSMODG00000005687	SEC23B	No change
2109	ENSMODG00000021448	NDUFV2	No change
949	ENSMODG00000020554	LIAS	No change
1903	ENSMODG00000010082	CHRNB3	No change
25	ENSMODG00000004560	SLC31A2	No change
821	ENSMODG00000027994	ENSMODT00000042960	No change
1733	ENSMODG00000002590	TECR	No change
960	ENSMODG00000002596	NDUFB7	No change
1141	ENSMODG00000005838	AP1M2	No change
543	ENSMODG00000001033	MYDGF	No change
194	ENSMODG00000001796	WDR72	No change
2089	ENSMODG00000020576	ENSMODT00000026197	No change
546	ENSMODG00000005772	FLVCR2	No change

849	ENSMODG00000013494	ZFP36	No change
1391	ENSMODG00000010149	UFD1	No change
1933	ENSMODG00000005101	ZCCHC2	No change
1589	ENSMODG00000010668	ERLIN1	No change
190	ENSMODG00000013485	CPNE2	No change
202	ENSMODG00000002586	CD247	No change
410	ENSMODG00000002336	DDX24	No change
916	ENSMODG00000024704	TLR5	No change
1251	ENSMODG00000019371	WHAMM	No change
398	ENSMODG00000008017	ENSMODT00000010171	No change
2024	ENSMODG00000008032	ENSMODT00000010188	No change
266	ENSMODG00000008042	ENSMODT00000010199	No change
970	ENSMODG00000013053	IFNLR1	No change
1821	ENSMODG00000017650	TRIT1	No change
1032	ENSMODG00000001087	ARFIP1	No change
119	ENSMODG00000017299	CREB3L4	No change
1715	ENSMODG00000016010	ENSMODT00000020336	No change
1804	ENSMODG00000008865	TIAL1	No change
680	ENSMODG00000010819	PPIB	No change
860	ENSMODG00000016643	DERL2	No change
1415	ENSMODG00000024981	PIGC	No change
1062	ENSMODG00000019013	SARAF	No change
1472	ENSMODG00000014189	SH3BGRL3	No change
276	ENSMODG00000006389	UEVLD	No change
1994	ENSMODG00000025013	ENSMODT00000038159	No change
473	ENSMODG00000020676	SRD5A3	No change
345	ENSMODG00000015634	GLT1D1	No change
75	ENSMODG00000019434	TMED3	No change
824	ENSMODG00000006663	DCTN5	No change
1851	ENSMODG00000001421	DHRS7B	No change
798	ENSMODG00000018137	UFL1	No change
225	ENSMODG00000020460	RAB27B	No change
1316	ENSMODG00000008861	AP1B1	No change
832	ENSMODG00000003063	PIP5K1B	No change
426	ENSMODG00000005388	SLC4A10	No change
1261	ENSMODG00000011236	ENSMODT00000014317	No change
1591	ENSMODG00000025707	ENSMODT00000040145	No change
1495	ENSMODG00000009307	ENSMODT00000011849	No change
518	ENSMODG00000011886	SLC23A1	No change
22	ENSMODG00000014275	ENSMODT00000018167	No change
858	ENSMODG00000014371	ENSMODT00000018297	No change
2081	ENSMODG00000004613	FAM129B	No change
2028	ENSMODG00000013328	SLC37A4	No change
1341	ENSMODG00000028289	ENSMODT00000044099	No change
1531	ENSMODG00000002266	ENSMODT00000002822	No change
206	ENSMODG00000020434	MALT1	No change
917	ENSMODG00000006592	COQ6	No change
672	ENSMODG00000011782	ABHD14B	No change
799	ENSMODG00000017550	STX7	No change
351	ENSMODG00000019875	SYT13	No change
360	ENSMODG00000004494	MVK	No change
1162	ENSMODG00000004591	COX4I1	No change

730	ENSMODG00000015368	SPAM1	No change
31	ENSMODG00000002443	MFSD9	No change
1290	ENSMODG00000013146	WARS	No change
1197	ENSMODG00000003506	DYM	No change
1174	ENSMODG00000003880	ENSMODT00000004853	No change
429	ENSMODG00000010381	ENSMODT00000013252	No change
1546	ENSMODG00000014125	ENSMODT00000017988	No change
143	ENSMODG00000020480	ACSS3	No change
669	ENSMODG00000007023	RGP1	No change
1898	ENSMODG00000025407	ENSMODT00000039644	No change
777	ENSMODG00000000035	DDIT4	No change
744	ENSMODG00000019014	PEX6	No change
982	ENSMODG00000019018	GNMT	No change
1881	ENSMODG00000011407	SNRNP27	No change
1139	ENSMODG00000005504	OVOL2	No change
1525	ENSMODG00000009873	PSMC5	No change
72	ENSMODG00000004320	ENSMODT00000005434	No change
145	ENSMODG00000004916	ENSMODT00000006177	No change
700	ENSMODG00000009549	COPA	No change
711	ENSMODG00000018326	CRACR2A	No change
1447	ENSMODG00000000067	MICU1	No change
1894	ENSMODG00000027579	ENSMODT00000041829	No change
446	ENSMODG00000008167	HIST2H2AC	No change
480	ENSMODG00000018761	ENSMODT00000023805	No change
867	ENSMODG00000023584	ENSMODT00000032124	No change
1305	ENSMODG00000029743	ENSMODT00000043668	No change
37	ENSMODG00000004387	GJB1	No change
869	ENSMODG00000021330	ENSMODT00000027137	No change
705	ENSMODG00000001955	OSTF1	No change
635	ENSMODG00000016412	SLX4	No change
1457	ENSMODG00000006616	SSU72	No change
1951	ENSMODG00000002824	CORO1C	No change
409	ENSMODG00000011603	ATG4A	No change
307	ENSMODG00000002801	SMC5	No change
1786	ENSMODG00000003838	HIST1H4E	No change
971	ENSMODG00000015862	IER3	No change
818	ENSMODG00000028102	ENSMODT00000042670	No change
1129	ENSMODG00000004843	GADD45B	No change
1813	ENSMODG00000009337	SCRN3	No change
1609	ENSMODG00000019005	GTF2E2	No change
796	ENSMODG00000004746	SYBU	No change
2098	ENSMODG00000016707	METTL26	No change
893	ENSMODG00000015064	ENSMODT00000019174	No change
240	ENSMODG00000005890	SLC24A3	No change
12	ENSMODG00000021226	SLC16A14	No change
1093	ENSMODG00000005235	ITGB6	No change
1832	ENSMODG00000004947	MAB21L3	No change
2012	ENSMODG00000024090	PRR15L	No change
58	ENSMODG00000015137	ATP13A4	No change
1817	ENSMODG00000017522	SLC18B1	No change
1548	ENSMODG00000014416	CHCHD3	No change
1728	ENSMODG00000004534	CARS2	No change

1161	ENSMODG00000007262	GBA2	No change
557	ENSMODG00000007277	CREB3	No change
1416	ENSMODG00000005473	MINK1	No change
928	ENSMODG00000013278	ENSMODT00000016911	No change
701	ENSMODG00000011238	NMT1	No change
2157	ENSMODG00000009892	RREB1	No change
1578	ENSMODG00000018380	OSBPL11	No change
1824	ENSMODG00000010474	GOLGA7	No change
2078	ENSMODG00000018854	ENSMODT00000023940	No change
814	ENSMODG00000011199	SPCS1	No change
969	ENSMODG00000011221	GLT8D1	No change
1318	ENSMODG00000005698	R3HDM4	No change
1535	ENSMODG00000016579	OAT	No change
1089	ENSMODG00000003488	EMC9	No change
317	ENSMODG00000011852	ANXA4	No change
1384	ENSMODG00000006878	TMEM104	No change
1823	ENSMODG00000014243	SEC24A	No change
1001	ENSMODG00000003735	GPCPD1	No change
1253	ENSMODG00000010038	GLOD4	No change
1374	ENSMODG00000020445	NARS	No change
1382	ENSMODG00000017168	ENSMODT00000021795	No change
1214	ENSMODG00000007137	ATP5H	No change
1173	ENSMODG00000004508	RAB3GAP2	No change
488	ENSMODG00000005120	C19orf66	No change
898	ENSMODG00000016750	RMND1	No change
283	ENSMODG00000005486	ENSMODT00000006921	No change
1055	ENSMODG00000005960	ENSMODT00000038324	No change
1371	ENSMODG00000010479	ALG11	No change
986	ENSMODG00000019053	VPS37A	No change
1695	ENSMODG00000003287	ENSMODT00000004102	No change
1392	ENSMODG00000016577	DESI1	No change
807	ENSMODG00000013695	MIEF2	No change
1109	ENSMODG00000009687	KDELR3	No change
1424	ENSMODG00000005748	PTGES2	No change
1071	ENSMODG00000003521	CDC14A	No change
598	ENSMODG00000001127	TRIM2	No change
2025	ENSMODG00000006776	PTGES	No change
150	ENSMODG00000009349	ERN1	No change
471	ENSMODG00000010307	COG6	No change
1565	ENSMODG00000018273	ANKRD6	No change
199	ENSMODG00000012190	SAR1A	No change
140	ENSMODG00000014967	ENSMODT00000019053	No change
745	ENSMODG00000013913	REXO2	No change
304	ENSMODG00000006534	NIPAL2	No change
1463	ENSMODG00000006605	NIPSNAP2	No change
1774	ENSMODG00000012891	HMGCL	No change
404	ENSMODG00000018554	DBR1	No change
1765	ENSMODG00000018322	DIRC2	No change
168	ENSMODG00000021346	OS9	No change
1935	ENSMODG00000002607	UXS1	No change
1970	ENSMODG00000014006	ENSMODT00000017843	No change
2033	ENSMODG00000021546	ERCC1	No change

373	ENSMODG00000008079	ENSMODT00000010245	No change
1540	ENSMODG00000003111	RNF10	No change
946	ENSMODG00000018405	FAM46A	No change
768	ENSMODG00000001194	CDKN2C	No change
1226	ENSMODG00000003871	ZDHHC15	No change
1493	ENSMODG00000004155	PLAA	No change
321	ENSMODG00000023331	ENSMODT00000010885	No change
890	ENSMODG00000017760	PDIA3	No change
888	ENSMODG00000016386	PIK3CG	No change
253	ENSMODG00000023582	ENSMODT00000032119	No change
790	ENSMODG00000028015	ENSMODT00000043011	No change
974	ENSMODG00000006915	GRIN2C	No change
1827	ENSMODG00000013411	CEP120	No change
962	ENSMODG00000009253	PAQR5	No change
44	ENSMODG00000007666	ENSMODT00000009712	No change
2174	ENSMODG00000016804	PCMT1	No change
56	ENSMODG00000009861	SLC24A1	No change
1528	ENSMODG00000016367	COG5	No change
1570	ENSMODG00000006958	ENSMODT00000008795	No change
1468	ENSMODG00000019232	TMEM97	No change
683	ENSMODG00000010689	ENSMODT00000013638	No change
1267	ENSMODG00000018443	ADA2	No change
632	ENSMODG00000016110	FAHD1	No change
327	ENSMODG00000016115	HAGH	No change
1507	ENSMODG00000015575	FOSL2	No change
1181	ENSMODG00000010779	SFMBT1	No change
211	ENSMODG00000005928	PLA2G12A	No change
968	ENSMODG00000017146	PBXIP1	No change
1891	ENSMODG00000015287	CSNK2B	No change
1123	ENSMODG00000006987	TMCC1	No change
314	ENSMODG00000015078	GMPPA	No change
1191	ENSMODG00000011620	ENSMODT00000014812	No change
1169	ENSMODG00000014956	SNAPC3	No change
1573	ENSMODG00000021044	ENSMODT00000026782	No change
185	ENSMODG00000004442	SUMF1	No change
1467	ENSMODG00000010979	FBXL22	No change
868	ENSMODG00000020983	DSCR3	No change
957	ENSMODG00000001109	NDRG1	No change
1826	ENSMODG00000012622	VPS41	No change
1446	ENSMODG00000017215	HAX1	No change
1291	ENSMODG00000007637	PCCA	No change
956	ENSMODG00000008095	RAB7A	No change
588	ENSMODG00000013916	SPDEF	No change
1031	ENSMODG00000019479	GPX8	No change
1402	ENSMODG00000014891	AGPAT1	No change
1978	ENSMODG00000004083	TMEM161A	No change
397	ENSMODG00000003854	FRRS1	No change
2103	ENSMODG00000004024	SNRPD3	No change
1704	ENSMODG00000017882	HAUS2	No change
2140	ENSMODG00000005689	PHF10	No change
1899	ENSMODG00000006633	HMOX2	No change
329	ENSMODG00000002976	TMEM206	No change

7	ENSMODG00000017506	ENSMODT00000022221	No change
1094	ENSMODG00000004777	BCAR3	No change
1690	ENSMODG00000011011	USP3	No change
2086	ENSMODG00000002293	IL1R2	No change
463	ENSMODG00000010558	MON1B	No change
687	ENSMODG00000020752	TMPRSS11E	No change
1597	ENSMODG00000008266	ENSMODT00000010470	No change
1389	ENSMODG00000004804	ENSMODT00000006038	No change
1659	ENSMODG00000004814	RAB24	No change
1075	ENSMODG00000020464	ENSMODT00000026061	No change
1044	ENSMODG00000024628	ENSMODT00000036518	No change
1537	ENSMODG00000017024	DAP3	No change
2171	ENSMODG00000021445	RALBP1	No change
965	ENSMODG00000028284	ENSMODT00000042385	No change
1577	ENSMODG00000014388	ENSMODT00000018314	No change
1234	ENSMODG00000014390	TIMM8B	No change
1020	ENSMODG00000029204	SRP14	No change
1180	ENSMODG00000017831	ZUFSP	No change
1059	ENSMODG00000021075	EVA1C	No change
1587	ENSMODG00000012936	ENSMODT00000028460	No change
502	ENSMODG00000023450	ENSMODT00000031488	No change
1496	ENSMODG00000023579	ENSMODT00000032092	No change
593	ENSMODG00000000481	CPT2	No change
1996	ENSMODG00000012614	PSMB3	No change
2001	ENSMODG00000008371	RAB1B	No change
219	ENSMODG00000020204	STX3	No change
793	ENSMODG00000019465	NDUFS4	No change
134	ENSMODG00000023261	FAM217B	No change
702	ENSMODG00000002730	SLC30A1	No change
451	ENSMODG00000012288	NUDT19	No change
531	ENSMODG00000015046	LURAP1L	No change
1727	ENSMODG00000006250	GDE1	No change
195	ENSMODG00000000876	ADIPOR1	No change
386	ENSMODG00000003340	RAB1A	No change
1313	ENSMODG00000013641	AP3S1	No change
626	ENSMODG00000012281	COQ10B	No change
2106	ENSMODG00000006112	RNASEK	No change
601	ENSMODG00000015822	MET	No change
1041	ENSMODG00000017946	EHD4	No change
736	ENSMODG00000010348	CLPX	No change
739	ENSMODG00000009786	ECI2	No change
1265	ENSMODG00000001128	PPIF	No change
874	ENSMODG00000007315	FN3KRP	No change
1746	ENSMODG00000005494	POLR2E	No change
1654	ENSMODG00000008388	DUSP22	No change
708	ENSMODG00000003635	ENSMODT00000004544	No change
90	ENSMODG00000008848	RHOU	No change
2163	ENSMODG00000007126	MRPL58	No change
896	ENSMODG00000018805	GOLPH3L	No change
511	ENSMODG00000021457	L3MBTL4	No change
15	ENSMODG00000020079	PCSK1	No change
1025	ENSMODG00000016799	ENTPD4	No change

1422	ENSMODG00000004718	LINGO3	No change
810	ENSMODG00000011736	DUSP7	No change
1406	ENSMODG00000019510	CIB1	No change
688	ENSMODG00000006585	PSPH	No change
1825	ENSMODG00000017665	UBE2L5P	No change
1687	ENSMODG00000017036	UCKL1	No change
1709	ENSMODG00000008075	NUDT2	No change
1133	ENSMODG00000004610	GNB2	No change
889	ENSMODG00000014659	ENSMODT00000018649	No change
1729	ENSMODG00000009480	RNF103	No change
77	ENSMODG00000002974	ATP6V0B	No change
908	ENSMODG00000006641	PRKCSH	No change
1215	ENSMODG00000016193	TSR3	No change
854	ENSMODG00000008851	ENSMODT00000039733	No change
1646	ENSMODG00000002205	TAF13	No change
2087	ENSMODG00000004291	ALG14	No change
2145	ENSMODG00000019309	PLAC8	No change
1740	ENSMODG00000019311	COPS4	No change
1796	ENSMODG00000006974	UQCRB	No change
1262	ENSMODG00000019692	ENSMODT00000025004	No change
1615	ENSMODG00000011576	INPP1	No change
138	ENSMODG00000015750	TMEM214	No change
1593	ENSMODG00000020416	MTMR12	No change
1922	ENSMODG00000020894	TSR2	No change
1451	ENSMODG00000002770	ENSMODT00000003442	No change
1512	ENSMODG00000005754	C9orf16	No change
1459	ENSMODG00000006330	MAP2K6	No change
1887	ENSMODG00000015005	ENSMODT00000019100	No change
1425	ENSMODG00000017090	SERINC2	No change
1454	ENSMODG00000006970	ELOC	No change
802	ENSMODG00000005358	SLC25A11	No change
2155	ENSMODG00000012301	RBM5	No change
1743	ENSMODG00000001831	NDUFB9	No change
1607	ENSMODG00000018112	ATG3	No change
1504	ENSMODG00000002281	MARVELD3	No change
1701	ENSMODG00000025790	BCAS1	No change
1772	ENSMODG00000018989	MRPL2	No change
2134	ENSMODG00000028634	ZNF16	No change
618	ENSMODG00000003729	COPE	No change
1905	ENSMODG00000024486	ENY2	No change
1009	ENSMODG00000006609	CLDN7	No change
2023	ENSMODG00000020205	MRPL16	No change
1210	ENSMODG00000019701	MRPS36	No change
1063	ENSMODG00000017216	ENSMODT00000021850	No change
760	ENSMODG00000017592	ENSMODT00000022327	No change
1040	ENSMODG00000022598	ENSMODT00000028458	No change
1410	ENSMODG00000011270	KDM1B	No change
763	ENSMODG00000003934	TRIM7	No change
1780	ENSMODG00000017925	EPS8	No change
1828	ENSMODG00000013861	SRRM1	No change
1656	ENSMODG00000003152	ENSMODT00000003923	No change
126	ENSMODG00000011191	TSTA3	No change

111	ENSMODG00000014743	CNMD	No change
1539	ENSMODG00000006399	MBD4	No change
895	ENSMODG00000005873	ENSMODT00000007412	No change
1176	ENSMODG00000007050	NAGA	No change
53	ENSMODG00000009245	MPI	No change
940	ENSMODG00000011051	DUSP26	No change
2056	ENSMODG00000028168	COX5A	No change
691	ENSMODG00000028213	ENSMODT00000042961	No change
614	ENSMODG00000012386	CYB5A	No change
1554	ENSMODG00000014934	ENSMODT00000019013	No change
1517	ENSMODG00000021282	ENSMODT00000027081	No change
1110	ENSMODG00000002175	GOLGA5	No change
184	ENSMODG00000020887	ALDH4A1	No change
599	ENSMODG00000006876	PLSCR3	No change
1561	ENSMODG00000002179	DERL1	No change
826	ENSMODG00000020514	BDH1	No change
673	ENSMODG00000011438	MFSD1	No change
633	ENSMODG00000018283	ENSMODT00000023201	No change
978	ENSMODG00000000835	RAB3GAP1	No change
538	ENSMODG00000025398	ENSMODT00000039605	No change
1855	ENSMODG00000011946	GEMIN2	No change
1562	ENSMODG00000023529	ENSMODT00000031851	No change
1514	ENSMODG00000024823	ENSMODT00000037373	No change
1491	ENSMODG00000000394	ENSMODT00000000483	No change
523	ENSMODG00000012349	VPS37B	No change
1220	ENSMODG00000017801	GOLT1B	No change
552	ENSMODG00000001175	TMEM131L	No change
9	ENSMODG00000002807	ENSMODT00000003483	No change
584	ENSMODG00000011094	ENSMODT00000034521	No change
356	ENSMODG00000018328	PDIA5	No change
1379	ENSMODG00000009571	PEX19	No change
1502	ENSMODG00000016060	COX5B	No change
305	ENSMODG00000020976	BACH1	No change
1726	ENSMODG00000003708	ENSMODT00000004643	No change
497	ENSMODG00000017667	YDJC	No change
791	ENSMODG00000017367	RINT1	No change
679	ENSMODG00000007427	PDK3	No change
686	ENSMODG00000014239	SLC39A7	No change
1060	ENSMODG00000002012	ENSMODT00000002502	No change
2179	ENSMODG00000017711	ETNK1	No change
1575	ENSMODG00000014918	MLX	No change
535	ENSMODG00000008595	ENSMODT00000010917	No change
1501	ENSMODG00000014308	VAC14	No change
2102	ENSMODG00000018750	PEX11B	No change
2164	ENSMODG00000003472	PSME2	No change
2095	ENSMODG00000001548	DRAM2	No change
2128	ENSMODG00000000043	DNAJB12	No change
2002	ENSMODG00000024091	RPP38	No change
1190	ENSMODG00000015049	NR2F6	No change
1957	ENSMODG00000016402	TRAP1	No change
1475	ENSMODG00000017209	CA5B	No change
1218	ENSMODG00000021339	CDK2AP2	No change

1629	ENSMODG00000010209	ENSMODT00000013014	No change
408	ENSMODG00000011446	NDUFA2	No change
1983	ENSMODG00000025684	HIST2H3PS2	No change
1612	ENSMODG00000023588	ENSMODT00000032145	No change
2154	ENSMODG00000021263	TM4SF18	No change
2079	ENSMODG00000010421	SSFA2	No change
2182	ENSMODG00000004839	ENSMODT00000006084	No change
1464	ENSMODG00000003406	UBE2G2	No change
363	ENSMODG00000009810	RAB11A	No change
424	ENSMODG00000018833	CDC42SE1	No change
1967	ENSMODG00000002682	NCOA4	No change
2006	ENSMODG00000021336	NDUFV1	No change
369	ENSMODG00000019704	SERINC5	No change
1303	ENSMODG00000014672	ENSMODT00000018673	No change
1666	ENSMODG00000025729	USMG5	No change
671	ENSMODG00000023025	ENSMODT00000029470	No change
1797	ENSMODG00000025416	CGGBP1	No change
2031	ENSMODG00000005479	ENSMODT00000006915	No change
347	ENSMODG00000013064	GRHL3	No change
1232	ENSMODG00000028092	ENSMODT00000042906	No change
1324	ENSMODG00000016095	NDUFB10	No change
1719	ENSMODG00000016077	ENSMODT00000020430	No change
1277	ENSMODG00000003449	SCYL3	No change
1862	ENSMODG00000004039	LENG1	No change
1814	ENSMODG00000009093	CHMP7	No change
1648	ENSMODG00000017025	ENSMODT00000021613	No change
476	ENSMODG00000017244	ENSMODT00000021900	No change
1713	ENSMODG00000011871	TMEM92	No change
1799	ENSMODG00000004627	TM9SF3	No change
1096	ENSMODG00000005635	ENSMODT00000007117	No change
2032	ENSMODG00000021025	NDUFV3	No change
2083	ENSMODG00000019148	COX18	No change
1681	ENSMODG00000003584	FBXO8	No change
218	ENSMODG00000010695	STX12	No change
41	ENSMODG00000008203	AGTPBP1	No change
2048	ENSMODG00000019602	AGBL1	No change
152	ENSMODG00000015602	ENSMODT00000019817	No change
330	ENSMODG00000017563	SCFD1	No change
64	ENSMODG00000000800	BRD9	No change
1105	ENSMODG00000017736	INPP5B	No change
147	ENSMODG00000011470	ACSL4	No change
856	ENSMODG00000013640	NDUFB2	No change
2088	ENSMODG00000002486	PDE6D	No change
379	ENSMODG00000013443	GORASP1	No change
469	ENSMODG00000020690	DAPP1	No change
789	ENSMODG00000008828	ENSMODT00000011208	No change
1664	ENSMODG00000006458	ENDOG	No change
1072	ENSMODG00000015993	TRIM26	No change
647	ENSMODG00000004402	BSPRY	No change
1555	ENSMODG00000016226	PDCD10	No change
57	ENSMODG00000016222	SERPINI1	No change
1098	ENSMODG00000015080	CALU	No change

1236	ENSMODG00000012274	ABHD18	No change
843	ENSMODG00000005918	ENSMODT00000007470	No change
1046	ENSMODG00000008153	ATP6V0D2	No change
2156	ENSMODG00000007438	TSEN54	No change
1095	ENSMODG00000014766	NKIRAS2	No change
759	ENSMODG00000016536	ENSMODT00000021010	No change
1269	ENSMODG00000007321	ENSMODT00000009262	No change
191	ENSMODG00000019497	HM13	No change
509	ENSMODG00000024782	GORASP2	No change
1958	ENSMODG00000014706	IAH1	No change
1916	ENSMODG00000018950	VEGFA	No change
865	ENSMODG00000013679	ACOT8	No change
2063	ENSMODG00000017786	LDHB	No change
6	ENSMODG00000010129	CROT	No change
1934	ENSMODG00000019853	ATP5B	No change
1975	ENSMODG00000004243	TMUB1	No change
1506	ENSMODG00000001865	EDEM2	No change
2170	ENSMODG00000004328	C9orf72	No change
259	ENSMODG00000020555	SMIM14	No change
1753	ENSMODG00000007838	COPS5	No change
990	ENSMODG00000013906	DBNL	No change
1421	ENSMODG00000004563	PDXDC1	No change
302	ENSMODG00000000185	ARF4	No change
728	ENSMODG00000019697	ENSMODT00000025013	No change
1874	ENSMODG00000023902	ENSMODT00000033736	No change
1051	ENSMODG00000000207	RHOV	No change
1326	ENSMODG00000000907	MPND	No change
403	ENSMODG00000009116	ENSMODT00000011588	No change
881	ENSMODG00000018706	LGSN	No change
1061	ENSMODG00000006474	STK3	No change
1483	ENSMODG00000003895	ENSMODT00000004876	No change
293	ENSMODG00000009751	SCYL1	No change
1367	ENSMODG00000009062	FAM83H	No change
828	ENSMODG00000001308	LONP1	No change
611	ENSMODG00000009731	ENSMODT00000012387	No change
591	ENSMODG00000019423	CCDC125	No change
1241	ENSMODG00000023773	CD14	No change
1308	ENSMODG00000010090	ENSMODT00000012866	No change
433	ENSMODG00000002362	RNF128	No change
1669	ENSMODG00000016146	TCEA3	No change
612	ENSMODG00000017726	ST8SIA1	No change
934	ENSMODG00000001864	PDIA4	No change
644	ENSMODG00000009042	SEC61G	No change
128	ENSMODG00000006901	NPL	No change
937	ENSMODG00000013566	ENSMODT00000017287	No change
2110	ENSMODG00000010853	MED18	No change
1351	ENSMODG00000002070	EIF2D	No change
721	ENSMODG00000001027	GRAMD2B	No change
1523	ENSMODG00000001143	ENSMODT00000001388	No change
343	ENSMODG00000008172	ZDHHC24	No change
2138	ENSMODG00000014424	MRPS35	No change
1030	ENSMODG00000010423	ATG16L1	No change

1177	ENSMODG00000017499	HBS1L	No change
592	ENSMODG00000002931	SLC6A9	No change
1306	ENSMODG00000006640	ENSMODT00000008400	No change
1914	ENSMODG00000019082	TYW3	No change
1567	ENSMODG00000013962	COX6B1	No change
1723	ENSMODG00000013778	PPARD	No change
786	ENSMODG00000017141	ENSMODT00000021765	No change
1928	ENSMODG00000025335	ENSMODT00000003056	No change
846	ENSMODG00000009267	MPP1	No change
1342	ENSMODG00000019463	FST	No change
1863	ENSMODG00000025408	TXNDC12	No change
416	ENSMODG00000003053	ENSMODT00000003792	No change
1364	ENSMODG00000012747	BDKRB1	No change
572	ENSMODG00000005619	DAD1	No change
1355	ENSMODG00000007268	ESRP1	No change
334	ENSMODG00000010846	SESN2	No change
220	ENSMODG00000020958	C2CD2	No change
166	ENSMODG00000009820	SLC26A2	No change
1560	ENSMODG00000002100	SLC24A4	No change
955	ENSMODG00000018256	TMEM52B	No change
1519	ENSMODG00000020398	AMACR	No change
1208	ENSMODG00000004277	ENSMODT00000005382	No change
1943	ENSMODG00000010300	SLC39A9	No change
221	ENSMODG00000008869	ENSMODT00000011264	No change
1489	ENSMODG00000008368	IPCEF1	No change
393	ENSMODG00000024846	PHLDA3	No change
1973	ENSMODG00000011636	GTF2A2	No change
1909	ENSMODG00000018501	ASB13	No change
1488	ENSMODG00000003122	ENSMODT00000003891	No change
288	ENSMODG00000007607	ENSMODT00000009623	No change
1820	ENSMODG00000009176	SCAMP5	No change
2131	ENSMODG00000001410	TMEM11	No change
1455	ENSMODG00000002345	TBC1D8B	No change
936	ENSMODG00000002678	ENSMODT00000003316	No change
2015	ENSMODG00000015317	BAG6	No change
171	ENSMODG00000012066	PALM3	No change
1285	ENSMODG00000019058	MICU3	No change
1912	ENSMODG00000002140	FKBP1B	No change
1101	ENSMODG00000021532	IRF2BP1	No change
2161	ENSMODG00000017989	CEP57L1	No change
909	ENSMODG00000017990	SESN1	No change
468	ENSMODG00000016006	ALPL	No change
396	ENSMODG00000018929	NFKBIE	No change
1322	ENSMODG00000008074	LAMTOR1	No change
1735	ENSMODG00000005236	JAGN1	No change
1858	ENSMODG00000009055	ENSMODT00000011513	No change
1288	ENSMODG00000011543	GRN	No change
1808	ENSMODG00000015138	SLC44A4	No change
1272	ENSMODG00000016148	NPC1L1	No change
699	ENSMODG00000021497	C18orf8	No change
717	ENSMODG00000019361	NMB	No change
1623	ENSMODG00000007323	RAP1B	No change

704	ENSMODG00000002862	GRHPR	No change
646	ENSMODG00000016934	VGLL3	No change
39	ENSMODG00000018522	SLC17A5	No change
466	ENSMODG00000020454	TXNL1	No change
664	ENSMODG00000004468	TSC22D4	No change
533	ENSMODG00000020649	SCFD2	No change
481	ENSMODG00000024852	ENSMODT00000037490	No change
924	ENSMODG00000016289	SLC5A11	No change
1286	ENSMODG00000013188	THY1	No change
2064	ENSMODG00000007422	RNF2	No change
510	ENSMODG00000007719	FAM214B	No change
1886	ENSMODG00000015672	AACS	No change
2121	ENSMODG00000008099	EIF2AK1	No change
217	ENSMODG00000018960	SEC24D	No change
1923	ENSMODG00000019758	GOLGA1	No change
1235	ENSMODG00000007201	ENSMODT00000009101	No change
378	ENSMODG00000009540	BMP1	No change
1470	ENSMODG00000011352	SACM1L	No change
677	ENSMODG00000003455	PGPEP1	No change
1330	ENSMODG00000020410	SUB1	No change
560	ENSMODG00000019678	BNIP3	No change
506	ENSMODG00000003106	NECAP2	No change
1067	ENSMODG00000021092	MAOB	No change
1432	ENSMODG00000029504	SNX12	No change
1739	ENSMODG00000005285	VPS4B	No change
2120	ENSMODG00000018142	MANEA	No change
1662	ENSMODG00000019488	UNC45A	No change
1544	ENSMODG00000005182	NDUFS7	No change
1205	ENSMODG00000000370	VSTM5	No change
1016	ENSMODG00000003917	ENSMODT00000004900	No change
117	ENSMODG00000013132	SLC25A29	No change
1871	ENSMODG00000020241	COX7C	No change
1594	ENSMODG00000021533	FOXA3	No change
1829	ENSMODG00000010874	TRAPPC8	No change
51	ENSMODG00000019888	CREB3L1	No change
1611	ENSMODG00000002350	PRPF38B	No change
813	ENSMODG00000012519	DDX55	No change
1676	ENSMODG00000011188	PAIP2B	No change
1550	ENSMODG00000025630	ENSMODT00000040041	No change
1332	ENSMODG00000007434	NAA38	No change
146	ENSMODG00000018869	SELENBP1	No change
1942	ENSMODG00000004798	ENSMODT00000006032	No change
2150	ENSMODG00000005376	AHSA1	No change
273	ENSMODG00000028246	SSR1	No change
1477	ENSMODG00000020867	ENSMODT00000026559	No change
1378	ENSMODG00000003825	AQP3	No change
752	ENSMODG00000007384	NADSYN1	No change
1155	ENSMODG00000010913	PIN4	No change
787	ENSMODG00000015090	NDUFB3	No change
754	ENSMODG00000006869	GIPC3	No change
1784	ENSMODG00000015678	MPV17	No change
60	ENSMODG00000002771	ENSMODT00000003443	No change

109	ENSMODG00000015718	PREB	No change
2090	ENSMODG00000010014	TIMM22	No change
636	ENSMODG00000013979	SLC22A4	No change
625	ENSMODG00000000915	TMEM183A	No change
1747	ENSMODG00000006058	TMEM208	No change
1247	ENSMODG00000004559	ENSMODT00000005738	No change
550	ENSMODG00000008204	SRP68	No change
352	ENSMODG00000006613	TPD52	No change
794	ENSMODG00000028739	TMEM255B	No change
296	ENSMODG00000017691	BCAT1	No change
1785	ENSMODG00000016394	UBB	No change
716	ENSMODG00000002902	MRPL12	No change
1651	ENSMODG00000014348	TXNDC15	No change
48	ENSMODG00000022790	ENSMODT00000028822	No change
1036	ENSMODG00000021052	CLIC6	No change
1805	ENSMODG00000020610	PAPSS1	No change
414	ENSMODG00000013139	SLC25A47	No change
1653	ENSMODG00000018871	PSMB4	No change
1991	ENSMODG00000002142	CBX4	No change
2043	ENSMODG00000002302	ALG3	No change
300	ENSMODG00000015182	SND1	No change
1896	ENSMODG00000010826	ATPIF1	No change
769	ENSMODG00000015328	CNPPD1	No change
2181	ENSMODG00000018036	HACE1	No change
2111	ENSMODG00000012094	NPRL2	No change
1147	ENSMODG00000011979	SIL1	No change
1452	ENSMODG00000002614	ACSS2	No change
107	ENSMODG00000018338	ENSMODT00000023270	No change
1038	ENSMODG00000013440	TSKS	No change
667	ENSMODG00000000522	SLC10A7	No change
1778	ENSMODG00000001820	TMC8	No change
434	ENSMODG00000015719	FEM1C	No change
1571	ENSMODG00000010108	TM7SF2	No change
1275	ENSMODG00000001077	EPCAM	No change
1736	ENSMODG00000013211	C14orf166	No change
1033	ENSMODG00000017848	TRAPPC3	No change
922	ENSMODG00000009083	ENSMODT00000011542	No change
1904	ENSMODG00000014904	FAM32A	No change
1018	ENSMODG00000016875	BEND7	No change
1245	ENSMODG00000015062	CMC1	No change
1757	ENSMODG00000011840	FBXO33	No change
1242	ENSMODG00000008822	FAM114A2	No change
1081	ENSMODG00000008225	ENSMODT00000010416	No change
1557	ENSMODG00000016950	TPRKB	No change
2099	ENSMODG00000008067	CPNE3	No change
2117	ENSMODG00000018619	TXNRD2	No change
767	ENSMODG00000015468	YIPF4	No change
1968	ENSMODG00000001323	ENSMODT00000001632	No change
1956	ENSMODG00000005573	PSMB1	No change
2070	ENSMODG00000003136	IFI30	No change
94	ENSMODG00000002930	FRMPD1	No change
271	ENSMODG00000014425	ENSMODT00000018675	No change

1976	ENSMODG00000020889	HSD17B10	No change
1788	ENSMODG00000007179	FXR2	No change
1926	ENSMODG00000003984	TSEN34	No change
2058	ENSMODG00000021531	MYPOP	No change
1946	ENSMODG00000015526	ENSMODT00000019726	No change
1465	ENSMODG00000005432	DPP4	No change
1294	ENSMODG00000011290	ESD	No change
247	ENSMODG00000018353	UMPS	No change
316	ENSMODG00000017889	SNAP23	No change
2060	ENSMODG00000008537	TIMD4	No change
566	ENSMODG00000013432	ARCN1	No change
2141	ENSMODG00000027658	CNIH3	No change
1255	ENSMODG00000024538	ENSMODT00000014226	No change
921	ENSMODG00000021477	ENSMODT00000027315	No change
747	ENSMODG00000002417	ENSMODT00000003001	No change
18	ENSMODG00000017569	ENPP3	No change
2176	ENSMODG00000011245	DCAKD	No change
1601	ENSMODG00000019666	GATD1	No change
761	ENSMODG00000016848	ARFGAP1	No change
1509	ENSMODG00000015724	PRRC1	No change
1529	ENSMODG00000001336	FBXO7	No change
1839	ENSMODG00000008847	SLC25A12	No change
131	ENSMODG00000014998	ENSMODT00000019092	No change
1118	ENSMODG00000002922	OASL	No change
1953	ENSMODG00000019761	ENSMODT00000025100	No change
670	ENSMODG00000008170	ENSMODT00000037587	No change
1023	ENSMODG00000004530	ARL8B	No change
512	ENSMODG00000007020	FADS6	No change
1831	ENSMODG00000009181	ENSMODT00000011675	No change
1553	ENSMODG00000001343	ENSMODT00000001655	No change
364	ENSMODG00000014461	SYTL1	No change
507	ENSMODG00000014382	ERGIC2	No change
1960	ENSMODG00000009473	ENSMODT00000012060	No change
1086	ENSMODG00000012296	ATP5G1	No change
1078	ENSMODG00000010707	ENSMODT00000013663	No change
845	ENSMODG00000011283	ENSMODT00000014379	No change
1620	ENSMODG00000013437	WDR48	No change
1815	ENSMODG00000003224	ENSMODT00000004010	No change
1014	ENSMODG00000011628	ARPC3	No change
1754	ENSMODG00000025281	ENSMODT00000039177	No change
2130	ENSMODG00000001772	ENSMODT00000002212	No change
489	ENSMODG00000010287	YIPF5	No change
1792	ENSMODG00000006711	GPR137	No change
1298	ENSMODG00000020739	HSD17B11	No change
1599	ENSMODG00000017183	NRBF2	No change
1223	ENSMODG00000006756	ENSMODT00000008538	No change
81	ENSMODG00000007897	ENSMODT00000010015	No change
459	ENSMODG00000018493	SRPRB	No change
2042	ENSMODG00000024910	ELMOD2	No change
1296	ENSMODG00000009276	ENSMODT00000011810	No change
1168	ENSMODG00000028247	ENSMODT00000042253	No change
872	ENSMODG00000008763	DNASE1L1	No change

1377	ENSMODG00000007671	PLD3	No change
1004	ENSMODG00000015551	PUS1	No change
989	ENSMODG00000006900	CREM	No change
1930	ENSMODG00000000930	TAPT1	No change
1982	ENSMODG00000013476	CETP	No change
1000	ENSMODG00000007363	WRAP53	No change
1732	ENSMODG00000012802	STARD3	No change
1420	ENSMODG00000006498	ACADVL	No change
1725	ENSMODG00000007983	PROM2	No change
2008	ENSMODG00000008181	NAA35	No change
1003	ENSMODG00000020628	TEC	No change
1006	ENSMODG00000020852	PPP1R37	No change
1479	ENSMODG00000024148	ENSMODT00000034928	No change
1201	ENSMODG00000027366	ENSMODT00000043880	No change
2113	ENSMODG00000015481	DPY30	No change
1140	ENSMODG00000013696	SLC37A3	No change
999	ENSMODG00000003470	ELL	No change
1721	ENSMODG00000016765	MTHFD1L	No change
1657	ENSMODG00000011190	ACBD4	No change
964	ENSMODG00000018816	TMEM14A	No change
2167	ENSMODG00000013680	MDH2	No change
1359	ENSMODG00000000403	ENSMODT00000000494	No change
666	ENSMODG00000014644	TMEM38B	No change
1952	ENSMODG00000020100	ENSMODT00000025561	No change
1249	ENSMODG00000013126	YIF1B	No change
1706	ENSMODG00000010543	CHUK	No change
2036	ENSMODG00000014089	BAK1	No change
1549	ENSMODG00000006897	MFSD12	No change
1427	ENSMODG00000001145	ANXA11	No change
324	ENSMODG00000016964	SSR2	No change
656	ENSMODG00000023125	B3GNT2	No change
1764	ENSMODG00000007799	PRDX4	No change
328	ENSMODG00000008102	VAT1L	No change
1889	ENSMODG00000016068	GFER	No change
1204	ENSMODG00000007037	ENSMODT00000008897	No change
561	ENSMODG00000007030	NDUFA6	No change
879	ENSMODG00000003237	SMYD2	No change
551	ENSMODG00000021165	ACAA1	No change
1400	ENSMODG00000027567	ENSMODT00000043320	No change
959	ENSMODG00000004337	TMED9	No change
657	ENSMODG00000004100	STT3A	No change
1057	ENSMODG00000017225	NOXA1	No change
326	ENSMODG00000020850	TRAPPC6A	No change
1641	ENSMODG00000010344	TANGO2	No change
443	ENSMODG00000018192	TMEM39A	No change
1380	ENSMODG00000007560	TRAPPC1	No change
1800	ENSMODG00000019589	MRPS11	No change
1873	ENSMODG00000016966	ENSMODT00000021542	No change
1349	ENSMODG00000013171	TRIM29	No change
1844	ENSMODG00000000244	KDELR1	No change
1963	ENSMODG00000005207	ENSMODT00000006549	No change
1674	ENSMODG00000003679	APLF	No change

1527	ENSMODG00000000653	CYCS	No change
1667	ENSMODG00000003691	ENSMODT00000004619	No change
2062	ENSMODG00000014422	KDF1	No change
486	ENSMODG00000001017	BST1	No change
1104	ENSMODG00000010485	FUOM	No change
663	ENSMODG00000006782	COG7	No change
1682	ENSMODG00000021300	NDUFB5	No change
1987	ENSMODG00000019862	HSD17B12	No change
1850	ENSMODG00000009795	MEGF11	No change
62	ENSMODG00000014640	FA2H	No change
665	ENSMODG00000007777	RHOD	No change
2029	ENSMODG00000000660	AP3M1	No change
758	ENSMODG00000011202	SRM	No change
2168	ENSMODG00000016018	PNPLA8	No change
447	ENSMODG00000012923	MDFI	No change
2035	ENSMODG00000013429	NFKBIB	No change
661	ENSMODG00000007335	COPB1	No change
1683	ENSMODG00000025740	C14orf2	No change
1478	ENSMODG00000021013	MRPL39	No change
1327	ENSMODG00000010522	HGH1	No change
55	ENSMODG00000014340	SLC7A4	No change
2067	ENSMODG00000008817	NAPA	No change
1680	ENSMODG00000013742	GYS1	No change
1893	ENSMODG00000021192	FARSB	No change
1319	ENSMODG00000000423	SEPSECS	No change
1631	ENSMODG00000021182	PSMD13	No change
20	ENSMODG00000028660	ENSMODT00000042428	No change
1763	ENSMODG00000000342	COX7A2L	No change
583	ENSMODG00000004861	TLR3	No change
1640	ENSMODG00000019656	TRAPPC13	No change
2122	ENSMODG00000018491	NDUFB11	No change
230	ENSMODG00000019845	DERL3	No change
1375	ENSMODG00000000980	ATF6	No change
1153	ENSMODG00000007908	GALT	No change
169	ENSMODG00000018777	COL21A1	No change
1192	ENSMODG00000005158	PPAN	No change
452	ENSMODG00000014742	CNP	No change
1353	ENSMODG00000009809	CD59	No change
1911	ENSMODG00000009961	PIGH	No change
16	ENSMODG00000016629	CDH26	No change
294	ENSMODG00000027578	FXYD4	No change
966	ENSMODG00000000329	PKP1	No change
1966	ENSMODG00000009373	TGDS	No change
815	ENSMODG00000015669	EIF2B4	No change
772	ENSMODG00000013504	ENSMODT00000017208	No change
400	ENSMODG00000011041	MBD2	No change
1835	ENSMODG00000009287	ST3GAL5	No change
1691	ENSMODG00000018804	ENSA	No change
1494	ENSMODG00000006181	SLC16A13	No change
827	ENSMODG00000003423	FASN	No change
1260	ENSMODG00000009585	AAGAB	No change
1077	ENSMODG00000007931	ENSMODT00000010061	No change

897	ENSMODG00000018053	ENSMODT00000022900	No change
1781	ENSMODG00000008238	FAM213B	No change
1022	ENSMODG00000007737	SCUBE2	No change
737	ENSMODG00000003437	ENSMODT00000004292	No change
1622	ENSMODG00000007809	VCP	No change
947	ENSMODG00000003595	ACAA2	No change
534	ENSMODG00000016129	ENSMODT00000020502	No change
907	ENSMODG00000006188	ATP6V0E1	No change
2082	ENSMODG00000005068	C19orf25	No change
2123	ENSMODG00000006523	ENSMODT00000008257	No change
746	ENSMODG00000011808	SYTL4	No change
422	ENSMODG00000011087	RPS27L	No change
929	ENSMODG00000010347	MRPS31	No change
1029	ENSMODG00000014738	ACLY	No change
2105	ENSMODG00000010120	CHRNA6	No change
485	ENSMODG00000003056	ARL1	No change
415	ENSMODG00000025624	ENSMODT00000040035	No change
341	ENSMODG00000012032	SELENOS	No change
779	ENSMODG00000001647	EMC7	No change
1453	ENSMODG00000013535	ZFYVE21	No change
1357	ENSMODG00000018300	FAM162A	No change
157	ENSMODG00000019776	ENSMODT00000025122	No change
1149	ENSMODG00000022818	ENSMODT00000028933	No change
387	ENSMODG00000001812	ENSMODT00000002256	No change
1936	ENSMODG00000000168	MRPS16	No change
602	ENSMODG00000007962	SLC25A35	No change
850	ENSMODG00000017578	MED23	No change
1738	ENSMODG00000012989	PSMD3	No change
1212	ENSMODG00000011377	C1orf210	No change
1196	ENSMODG00000005470	DDRGK1	No change
418	ENSMODG00000014753	GRHL1	No change
1368	ENSMODG00000014173	SETD6	No change
607	ENSMODG00000000474	SOD3	No change
1847	ENSMODG00000017744	ENSMODT00000022513	No change
2069	ENSMODG00000011324	ELOVL1	No change
1090	ENSMODG00000003076	SIRT7	No change
1588	ENSMODG00000019308	COQ2	No change
1	ENSMODG00000010106	OLAH	No change
1084	ENSMODG00000007134	MPDU1	No change
2022	ENSMODG00000001467	ENSMODT00000001818	No change
1672	ENSMODG00000008561	MRPL22	No change
101	ENSMODG00000024656	GREM2	No change
1252	ENSMODG00000013054	RBX1	No change
1134	ENSMODG00000004365	SLC6A14	No change
823	ENSMODG00000004895	GDPD2	No change
1888	ENSMODG00000007636	GALK1	No change
1626	ENSMODG00000015383	ASB15	No change
1606	ENSMODG00000006201	RPL26	No change
942	ENSMODG00000024773	IL19	No change
2173	ENSMODG00000016264	BCHE	No change
65	ENSMODG00000017400	S100A6	No change
2143	ENSMODG00000024174	VPS25	No change

470	ENSMODG00000021358	DIO1	No change
1282	ENSMODG00000000517	TIMM17A	No change
1154	ENSMODG00000004118	NDUFA3	No change
943	ENSMODG00000018271	ENSMODT00000023187	No change
1314	ENSMODG00000001890	ENSMODT00000002351	No change
1927	ENSMODG00000023928	TOMM7	No change
1387	ENSMODG00000015338	NHEJ1	No change
724	ENSMODG00000002423	GALK2	No change
80	ENSMODG00000006453	RAB3D	No change
2135	ENSMODG00000000990	UCHL3	No change
32	ENSMODG00000009333	ENSMODT00000011884	No change
399	ENSMODG00000005676	ENSMODT00000007179	No change
1830	ENSMODG00000020228	CCNH	No change
322	ENSMODG00000005322	NTM	No change
1184	ENSMODG00000005197	ENSMODT00000006534	No change
365	ENSMODG00000011566	HIBCH	No change
40	ENSMODG00000015547	LIX1	No change
1547	ENSMODG00000013536	HSD17B4	No change
783	ENSMODG00000009457	MTHFD1	No change
825	ENSMODG00000006608	C2CD4C	No change
1688	ENSMODG00000024700	ENSMODT00000036812	No change
1297	ENSMODG00000013335	ANKRD9	No change
524	ENSMODG00000011427	TSC22D3	No change
27	ENSMODG00000019249	SLC13A2	No change
1113	ENSMODG00000005036	CPNE9	No change
423	ENSMODG00000001985	GPR61	No change
904	ENSMODG00000019251	FOXN1	No change
1671	ENSMODG00000018229	NDUFB4	No change
1879	ENSMODG00000028368	ENSMODT00000042134	No change
1320	ENSMODG00000013849	UQCRQ	No change
1995	ENSMODG00000029468	ENSMODT00000043615	No change
1460	ENSMODG00000015717	TMEM219	No change
2011	ENSMODG00000016608	PRELID3B	No change
377	ENSMODG00000021193	MOGAT1	No change
1604	ENSMODG00000009915	SLC5A1	No change
2046	ENSMODG00000004183	CCDC81	No change
619	ENSMODG00000010069	ENSMODT00000012840	No change
21	ENSMODG00000016838	SLC17A9	No change
1619	ENSMODG00000019682	NDUFA8	No change
95	ENSMODG00000001509	ENSMODT00000001875	No change
778	ENSMODG00000004017	ZFYVE28	No change
608	ENSMODG00000018918	ADAD1	No change
1756	ENSMODG00000016023	ZNRD1	No change
925	ENSMODG00000016119	NUBP2	No change
47	ENSMODG00000000190	CHAC1	No change
2126	ENSMODG00000006544	FABP5	No change
346	ENSMODG00000002270	UNC79	No change
2092	ENSMODG00000010245	ENSMODT00000013073	No change
776	ENSMODG00000014875	DNAJC15	No change
1487	ENSMODG00000016872	TSACC	No change
694	ENSMODG00000015807	MYL1	No change
1480	ENSMODG00000008008	TMEM223	No change

820	ENSMODG00000011325	ENSMODT00000014426	No change
135	ENSMODG00000004064	TNFRSF11B	No change
472	ENSMODG00000007996	LRP2	No change
738	ENSMODG00000007382	EFNB3	No change
1806	ENSMODG00000025554	APOO	No change
87	ENSMODG00000000460	LGI2	No change
139	ENSMODG00000015428	VIL1	No change
648	ENSMODG00000009049	TMEM160	No change
1610	ENSMODG00000009502	PHYHIP	No change
1466	ENSMODG00000012445	ENSMODT00000015868	No change
265	ENSMODG00000006696	GP2	No change
1918	ENSMODG00000025496	ATP5J2	No change
1505	ENSMODG00000018011	NTF3	No change
984	ENSMODG00000018928	ENSMODT00000024036	No change
2071	ENSMODG00000002648	TMEM53	No change
1189	ENSMODG00000012200	NGFR	No change
1469	ENSMODG00000025658	ENSMODT00000040079	No change
349	ENSMODG00000028755	ENSMODT00000043963	No change
1182	ENSMODG00000008816	GPER1	No change
367	ENSMODG00000004721	ENSMODT00000005940	No change
340	ENSMODG00000011832	NAGS	No change
1585	ENSMODG00000018298	CCDC58	No change
178	ENSMODG00000008158	MAP7D2	No change
1434	ENSMODG00000009801	KIAA1549L	No change
1011	ENSMODG00000005419	MOV10L1	No change
1564	ENSMODG00000017683	SORD	No change
448	ENSMODG00000006216	PNPLA3	No change
1238	ENSMODG00000011975	ENSMODT00000015279	No change
1865	ENSMODG00000004680	PTGR1	No change
651	ENSMODG00000020748	SLC10A6	No change
594	ENSMODG00000025143	ENSMODT00000025935	No change
586	ENSMODG00000020962	FAM3B	No change
992	ENSMODG00000011032	CA12	No change
1608	ENSMODG00000014819	NOG	No change
1937	ENSMODG00000006841	USH1C	No change
284	ENSMODG00000003446	SLC25A34	No change
454	ENSMODG00000011062	CCK	No change
1845	ENSMODG00000017434	PEX7	No change
695	ENSMODG00000007815	OTOA	No change
932	ENSMODG00000000380	SCTR	No change
817	ENSMODG00000010442	ELOVL2	No change
438	ENSMODG00000001756	TMEM174	No change
1999	ENSMODG00000002830	CHMP4B	No change
298	ENSMODG00000005642	RBBP9	No change
1875	ENSMODG00000016704	WFIKKN1	No change
182	ENSMODG00000017413	ENSMODT00000022114	No change
1745	ENSMODG00000012807	PSMA6	No change
267	ENSMODG00000012352	SATB2	No change
120	ENSMODG00000001462	DRD5	No change
530	ENSMODG00000016042	HS3ST6	No change
3	ENSMODG00000028694	C8orf22	Not expressed
8	ENSMODG00000027463	CCL20	Not expressed

43	ENSMODG00000025559	ENSMODT00000039952	Not expressed
66	ENSMODG00000019571	RLBP1	Not expressed
103	ENSMODG00000011282	ENSMODT00000014392	Not expressed
129	ENSMODG00000018553	A4GNT	Not expressed
167	ENSMODG00000027883	ENSMODT00000025112	Not expressed
183	ENSMODG00000029764	ENSMODT00000043261	Not expressed
207	ENSMODG00000018097	TMPRSS7	Not expressed
261	ENSMODG00000023793	ENSMODT00000033225	Not expressed
281	ENSMODG00000005850	GABRE	Not expressed
323	ENSMODG00000017898	MGST1	Not expressed
375	ENSMODG00000002518	NPPC	Not expressed
385	ENSMODG00000012353	ENSMODT00000015759	Not expressed
421	ENSMODG00000028749	ENSMODT00000043113	Not expressed
495	ENSMODG00000029415	ENSMODT00000044352	Not expressed
553	ENSMODG00000003138	ENSMODT00000003908	Not expressed
631	ENSMODG00000029679	ENSMODT00000041856	Not expressed
652	ENSMODG00000001022	LRG1	Not expressed
674	ENSMODG00000019515	IL31RA	Not expressed
706	ENSMODG00000004363	FUT1	Not expressed
848	ENSMODG00000001877	ZPLD1	Not expressed
862	ENSMODG00000021104	OTC	Not expressed
878	ENSMODG00000007387	IL22	Not expressed
944	ENSMODG00000007499	ENSMODT00000009486	Not expressed
991	ENSMODG00000018285	AKAP3	Not expressed
1106	ENSMODG00000029663	ENSMODT00000043474	Not expressed
1136	ENSMODG00000026362	mdo-mir-1549	Not expressed
1144	ENSMODG00000027942	ENSMODT00000043759	Not expressed
1150	ENSMODG00000009807	ENSMODT00000012483	Not expressed
1151	ENSMODG00000017815	RFX6	Not expressed
1163	ENSMODG00000023018	U2	Not expressed
1185	ENSMODG00000009904	ENSMODT00000012620	Not expressed
1187	ENSMODG00000029685	ENSMODT00000043483	Not expressed
1216	ENSMODG00000012236	ENSMODT00000015608	Not expressed
1244	ENSMODG00000005821	ENSMODT00000037414	Not expressed
1376	ENSMODG00000025243	ENSMODT00000039081	Not expressed
1386	ENSMODG00000005863	MIOX	Not expressed
1448	ENSMODG00000015135	ENSMODT00000019257	Not expressed
1471	ENSMODG00000023794	ENSMODT00000033229	Not expressed
1522	ENSMODG00000009113	ENSMODT00000011585	Not expressed
1542	ENSMODG00000001869	ENSMODT00000002326	Not expressed
1556	ENSMODG00000001216	ENSMODT00000001486	Not expressed
1563	ENSMODG00000017508	ENSMODT00000022224	Not expressed
1652	ENSMODG00000004999	MTNR1A	Not expressed
1670	ENSMODG00000019628	FAM159B	Not expressed
1697	ENSMODG00000000768	ENSMODT00000000936	Not expressed
1700	ENSMODG00000023133	ENSMODT00000030116	Not expressed
1717	ENSMODG00000001355	BPIFC	Not expressed
1782	ENSMODG00000023007	U3	Not expressed
1822	ENSMODG00000022854	ENSMODT00000029099	Not expressed
1895	ENSMODG00000013396	ENSMODT00000017060	Not expressed
1939	ENSMODG00000021851	U5	Not expressed
1950	ENSMODG00000004800	SYCP1	Not expressed

2018	ENSMODG00000021559	ENSMODT00000027414	Not expressed
2034	ENSMODG00000015803	ADGRF3	Not expressed
2052	ENSMODG00000025785	CCDC196	Not expressed
2053	ENSMODG00000001067	KEL	Not expressed
2068	ENSMODG00000014086	ENSMODT00000017939	Not expressed
2125	ENSMODG00000024677	ENSMODT00000036702	Not expressed
2152	ENSMODG00000027005	U7	Not expressed
2180	ENSMODG00000024903	ENSMODT00000037729	Not expressed

Supplementary Table 4. Comparison of differentially expressed genes in downregulated in early pregnancy between *Monodelphis domestica* (opossum) and *Sminthopsis crassicaudata* (fat-tailed dunnart)			
**Opossum downregulated in early pregnancy rank**	**Opossum downregulated in early pregnancy**	**Gene Symbol/transcript ID**	**Expression levels in dunnart early pregnancy compared to non-pregnant**
1	ENSMODG00000005170	VTCN1	Down
4	ENSMODG00000015536	IGFBP5	Down
5	ENSMODG00000006420	KIF26B	Down
8	ENSMODG00000014370	ENSMODT00000018288	Down
20	ENSMODG00000002137	CBX2	Down
38	ENSMODG00000011338	SOX4	Down
47	ENSMODG00000000555	DPP10	Down
53	ENSMODG00000006490	NINL	Down
58	ENSMODG00000010271	IGDCC3	Down
79	ENSMODG00000008171	AMPD3	Down
80	ENSMODG00000019481	CCNO	Down
81	ENSMODG00000000922	DEUP1	Down
83	ENSMODG00000020725	PKD2	Down
84	ENSMODG00000005162	ENSMODT00000006484	Down
105	ENSMODG00000009399	ENSMODT00000011965	Down
108	ENSMODG00000018597	FOXL2	Down
113	ENSMODG00000018927	BBS7	Down
121	ENSMODG00000020909	CACNA1G	Down
124	ENSMODG00000007807	C14orf37	Down
128	ENSMODG00000012059	TBX3	Down
135	ENSMODG00000012390	TSHZ3	Down
137	ENSMODG00000004834	TRIP6	Down
139	ENSMODG00000016954	MEX3A	Down
146	ENSMODG00000011187	GREB1	Down
149	ENSMODG00000015403	GLIS3	Down
159	ENSMODG00000015983	CROCC2	Down
168	ENSMODG00000010094	WDR35	Down
170	ENSMODG00000019579	TNFSF15	Down
175	ENSMODG00000021243	CHST2	Down
187	ENSMODG00000006821	KCNIP1	Down
189	ENSMODG00000018141	P3H3	Down
191	ENSMODG00000001830	NOVA1	Down
195	ENSMODG00000005213	ZC4H2	Down
200	ENSMODG00000006068	SALL2	Down
206	ENSMODG00000020406	LGR5	Down
212	ENSMODG00000007977	CNTFR	Down
228	ENSMODG00000015171	POLE	Down
238	ENSMODG00000027595	ENSMODT00000042630	Down
244	ENSMODG00000014680	SMO	Down
258	ENSMODG00000019334	EFNA5	Down
260	ENSMODG00000029068	ENSMODT00000042441	Down
285	ENSMODG00000004966	ANO4	Down
286	ENSMODG00000021281	IGSF10	Down

291	ENSMODG00000006174	GPR153	Down
294	ENSMODG00000000954	KCNMA1	Down
297	ENSMODG00000009606	GFRA1	Down
310	ENSMODG00000007626	SLC41A3	Down
317	ENSMODG00000004976	TCTN3	Down
319	ENSMODG00000017938	RERG	Down
324	ENSMODG00000005727	PDE4A	Down
332	ENSMODG00000007524	ST5	Down
336	ENSMODG00000000911	ENSMODT00000001105	Down
339	ENSMODG00000019416	ARNT2	Down
351	ENSMODG00000014607	RFTN1	Down
352	ENSMODG00000016857	ROBO1	Down
358	ENSMODG00000012412	AR	Down
363	ENSMODG00000020495	ENSMODT00000028832	Down
364	ENSMODG00000010717	ENSMODT00000013675	Down
371	ENSMODG00000004739	EPHB4	Down
375	ENSMODG00000024012	RIOX1	Down
380	ENSMODG00000019643	CNTRL	Down
389	ENSMODG00000007585	CNTROB	Down
395	ENSMODG00000003592	LRRC61	Down
417	ENSMODG00000029358	COL15A1	Down
422	ENSMODG00000019403	CEMIP	Down
428	ENSMODG00000004898	DPP6	Down
430	ENSMODG00000020789	PRDM8	Down
436	ENSMODG00000001539	KANK4	Down
445	ENSMODG00000009971	ENSMODT00000012715	Down
451	ENSMODG00000016774	GATA5	Down
471	ENSMODG00000022528	ENSMODT00000028382	Down
477	ENSMODG00000004260	AXIN2	Down
480	ENSMODG00000011907	KAZALD1	Down
485	ENSMODG00000002143	C3	Down
489	ENSMODG00000006708	TTYH2	Down
493	ENSMODG00000018753	LIX1L	Down
503	ENSMODG00000008346	CDC42EP3	Down
509	ENSMODG00000016811	ENSMODT00000021361	Down
511	ENSMODG00000011437	POLE2	Down
516	ENSMODG00000001735	MYC	Down
548	ENSMODG00000009017	LRRC49	Down
557	ENSMODG00000006996	CDH11	Down
558	ENSMODG00000003807	CRTAC1	Down
571	ENSMODG00000018140	BOC	Down
572	ENSMODG00000000044	ENSMODT00000000047	Down
576	ENSMODG00000019535	ANGPT4	Down
581	ENSMODG00000021440	APCDD1	Down
587	ENSMODG00000002714	GLI3	Down
613	ENSMODG00000027338	ENSMODT00000041800	Down
627	ENSMODG00000000658	EDNRA	Down
629	ENSMODG00000017022	CACNA2D1	Down
633	ENSMODG00000016160	IFT140	Down
636	ENSMODG00000017586	AKAP7	Down
640	ENSMODG00000000646	LGR6	Down
642	ENSMODG00000001085	MFAP4	Down

646	ENSMODG00000012068	LRRK1	Down
654	ENSMODG00000015051	WNK4	Down
656	ENSMODG00000007265	RIBC2	Down
658	ENSMODG00000011222	PLCD3	Down
670	ENSMODG00000018676	POU6F1	Down
685	ENSMODG00000005011	FBN3	Down
688	ENSMODG00000005807	SMOC2	Down
699	ENSMODG00000016176	AEBP1	Down
700	ENSMODG00000020329	EGFLAM	Down
703	ENSMODG00000019899	C11orf49	Down
704	ENSMODG00000016375	GLIS2	Down
705	ENSMODG00000016420	CLUAP1	Down
708	ENSMODG00000003704	GPM6A	Down
710	ENSMODG00000002891	WSCD2	Down
715	ENSMODG00000003798	KCNH2	Down
720	ENSMODG00000017473	PCDH11X	Down
723	ENSMODG00000001183	SFRP2	Down
725	ENSMODG00000001074	MAPK7	Down
731	ENSMODG00000016355	NMRAL1	Down
750	ENSMODG00000018290	CNR1	Down
754	ENSMODG00000013409	ENSMODT00000030103	Down
755	ENSMODG00000015165	SPEG	Down
766	ENSMODG00000025001	DZIP1	Down
773	ENSMODG00000001806	ENSMODT00000002234	Down
779	ENSMODG00000021156	GLS2	Down
785	ENSMODG00000004657	ST3GAL4	Down
787	ENSMODG00000019548	KIF7	Down
799	ENSMODG00000021299	USP13	Down
801	ENSMODG00000010122	ZFP36L1	Down
806	ENSMODG00000019560	PLK2	Down
807	ENSMODG00000002910	DTL	Down
812	ENSMODG00000018325	SEMA5B	Down
824	ENSMODG00000009640	ATRNL1	Down
846	ENSMODG00000027600	ENSMODT00000042810	Down
856	ENSMODG00000006893	CMTM3	Down
858	ENSMODG00000023763	ENSMODT00000033131	Down
859	ENSMODG00000024882	ENSMODT00000037615	Down
871	ENSMODG00000001046	GPNMB	Down
874	ENSMODG00000002061	ENSMODT00000038235	Down
883	ENSMODG00000000453	HOXA11	Down
889	ENSMODG00000006695	ZC2HC1A	Down
897	ENSMODG00000015190	MEOX1	Down
902	ENSMODG00000015258	TMEM237	Down
908	ENSMODG00000007852	LTBP4	Down
909	ENSMODG00000007007	NPR2	Down
910	ENSMODG00000008578	PCNX4	Down
916	ENSMODG00000015701	KCNN2	Down
937	ENSMODG00000012511	NTNG2	Down
952	ENSMODG00000014746	GRIA4	Down
996	ENSMODG00000016434	ENSMODT00000020891	Down
1014	ENSMODG00000017056	SDC3	Down
1031	ENSMODG00000015212	MPP3	Down

1033	ENSMODG00000028733	ENSMODT00000042341	Down
1038	ENSMODG00000018014	SOBP	Down
1045	ENSMODG00000003263	PDE4C	Down
1048	ENSMODG00000011920	WTIP	Down
1067	ENSMODG00000013551	CCDC102A	Down
1081	ENSMODG00000018983	PTK7	Down
1084	ENSMODG00000010173	SNAI2	Down
1090	ENSMODG00000002526	PTCH2	Down
1099	ENSMODG00000029083	ENSMODT00000042608	Down
1102	ENSMODG00000010369	ITGA4	Down
1104	ENSMODG00000023321	TMEM200A	Down
1117	ENSMODG00000018291	CASR	Down
1143	ENSMODG00000006686	ENSMODT00000008452	Down
1165	ENSMODG00000016387	SALL4	Down
1166	ENSMODG00000016890	CHTF18	Down
1168	ENSMODG00000011014	RECQL4	Down
1169	ENSMODG00000004633	DBN1	Down
1175	ENSMODG00000009646	WT1	Down
1177	ENSMODG00000029105	FGF1	Down
1182	ENSMODG00000003856	DSCC1	Down
1195	ENSMODG00000018775	DST	Down
1196	ENSMODG00000002325	VSTM4	Down
1198	ENSMODG00000006347	KANK2	Down
1201	ENSMODG00000014540	NOTCH3	Down
1203	ENSMODG00000024674	ENSMODT00000036672	Down
1206	ENSMODG00000009785	GFRA2	Down
1227	ENSMODG00000029552	ENSMODT00000044039	Down
1233	ENSMODG00000001161	LEPR	Down
1255	ENSMODG00000001152	MCM5	Down
1282	ENSMODG00000013125	DAAM2	Down
1290	ENSMODG00000010970	ENSMODT00000013998	Down
1294	ENSMODG00000025641	CEP72	Down
1295	ENSMODG00000025217	ENSMODT00000039019	Down
1296	ENSMODG00000018769	MTMR11	Down
1319	ENSMODG00000015125	RBMS3	Down
1325	ENSMODG00000000463	HOXA10	Down
1332	ENSMODG00000006871	CPQ	Down
1337	ENSMODG00000002992	NFATC4	Down
1339	ENSMODG00000001731	EVC	Down
1341	ENSMODG00000003513	FLRT2	Down
1351	ENSMODG00000029253	LUZP2	Down
1354	ENSMODG00000000109	TBC1D19	Down
1376	ENSMODG00000006887	CRISPLD1	Down
1378	ENSMODG00000006537	SCN1A	Down
1388	ENSMODG00000014319	MTSS1L	Down
1404	ENSMODG00000005255	PAK3	Down
1405	ENSMODG00000019904	ENSMODT00000025273	Down
1431	ENSMODG00000018107	PIANP	Down
1445	ENSMODG00000029749	ENSMODT00000043150	Down
1449	ENSMODG00000007972	ENSMODT00000010119	Down
1465	ENSMODG00000007847	ZEB1	Down
1486	ENSMODG00000018877	FAT4	Down

1500	ENSMODG00000007648	PELI2	Down
1509	ENSMODG00000002290	MTBP	Down
1524	ENSMODG00000020425	TRHDE	Down
1529	ENSMODG00000018947	PDE5A	Down
1537	ENSMODG00000010536	SPIDR	Down
1543	ENSMODG00000015831	P2RY1	Down
1545	ENSMODG00000015042	MPDZ	Down
1566	ENSMODG00000010600	HIC1	Down
1579	ENSMODG00000000976	ENSMODT00000001187	Down
1587	ENSMODG00000018498	SLCO2A1	Down
1589	ENSMODG00000018766	SV2A	Down
1594	ENSMODG00000024040	ENSMODT00000034274	Down
1599	ENSMODG00000015598	STX2	Down
1605	ENSMODG00000009157	SLC8A1	Down
1612	ENSMODG00000003977	ENPP2	Down
1635	ENSMODG00000024585	ENSMODT00000036306	Down
1636	ENSMODG00000010848	ADGRA2	Down
1641	ENSMODG00000006642	SDCCAG8	Down
1647	ENSMODG00000015872	PTH2R	Down
1651	ENSMODG00000003548	HAND2	Down
1662	ENSMODG00000014926	RARB	Down
1669	ENSMODG00000002942	DPT	Down
1691	ENSMODG00000003766	ASB5	Down
1692	ENSMODG00000015361	WNT10A	Down
1695	ENSMODG00000011719	DRP2	Down
1720	ENSMODG00000021451	MTCL1	Down
1723	ENSMODG00000020475	ENSMODT00000026096	Down
1724	ENSMODG00000021408	CDH19	Down
1743	ENSMODG00000018991	NRG1	Down
1758	ENSMODG00000021426	SPIRE1	Down
1759	ENSMODG00000013389	BBS2	Down
1763	ENSMODG00000010808	KLHL14	Down
1765	ENSMODG00000011587	NFIX	Down
1778	ENSMODG00000018418	TTK	Down
1779	ENSMODG00000004496	ENSMODT00000005667	Down
1783	ENSMODG00000015435	LTBP1	Down
1798	ENSMODG00000002620	KIF2C	Down
1810	ENSMODG00000003959	PDZRN3	Down
1811	ENSMODG00000019261	ENSMODT00000024469	Down
1821	ENSMODG00000009510	OPRK1	Down
1835	ENSMODG00000028217	ENSMODT00000044079	Down
1855	ENSMODG00000003112	ZEB2	Down
213	ENSMODG00000025395	TMEM164	Up
217	ENSMODG00000012864	NETO2	Up
331	ENSMODG00000013831	SCUBE3	Up
398	ENSMODG00000002320	SLC27A2	Up
524	ENSMODG00000014007	MYO15A	Up
590	ENSMODG00000002444	DIAPH2	Up
599	ENSMODG00000007620	CARNS1	Up
683	ENSMODG00000020379	AGXT2	Up
797	ENSMODG00000010160	CADM3	Up
1174	ENSMODG00000023262	ENSMODT00000030744	Up

1246	ENSMODG00000001431	ACSBG2	Up
1379	ENSMODG00000015375	TTLL4	Up
1618	ENSMODG00000014079	ENSMODT00000017938	Up
1706	ENSMODG00000009240	ESR2	Up
1726	ENSMODG00000001207	LRRC6	Up
1793	ENSMODG00000027572	ENSMODT00000042647	Up
1827	ENSMODG00000002933	ENSMODT00000003648	Up
1837	ENSMODG00000007773	SPTBN4	Up
2	ENSMODG00000014286	COL11A2	No change
3	ENSMODG00000000183	LBP	No change
6	ENSMODG00000017856	HS3ST5	No change
7	ENSMODG00000001434	SCEL	No change
9	ENSMODG00000008390	NPTX2	No change
10	ENSMODG00000016292	ENSMODT00000020722	No change
11	ENSMODG00000008428	TNFRSF19	No change
12	ENSMODG00000012401	WNT5A	No change
13	ENSMODG00000014384	PLXNB1	No change
14	ENSMODG00000014333	FGD3	No change
15	ENSMODG00000010096	PPP2R2B	No change
16	ENSMODG00000003160	TGM1	No change
17	ENSMODG00000008143	GOLM1	No change
18	ENSMODG00000022855	ENSMODT00000029111	No change
19	ENSMODG00000023112	ARL4C	No change
21	ENSMODG00000003284	CDH3	No change
22	ENSMODG00000003819	MAPK4	No change
23	ENSMODG00000002866	PPP2R2C	No change
24	ENSMODG00000013454	PIM1	No change
25	ENSMODG00000020603	LEF1	No change
26	ENSMODG00000007603	ALDH1L1	No change
27	ENSMODG00000008413	EFCC1	No change
28	ENSMODG00000004385	NDE1	No change
29	ENSMODG00000004194	ABCC1	No change
30	ENSMODG00000005538	WNT11	No change
31	ENSMODG00000020671	KIT	No change
32	ENSMODG00000020674	SLC39A8	No change
33	ENSMODG00000013273	ETV5	No change
34	ENSMODG00000007280	PDE3B	No change
35	ENSMODG00000017728	CLVS2	No change
36	ENSMODG00000013586	PNPLA1	No change
37	ENSMODG00000010764	ENSMODT00000013735	No change
39	ENSMODG00000008498	FAM20C	No change
40	ENSMODG00000019295	RAB34	No change
41	ENSMODG00000010024	IGSF9	No change
42	ENSMODG00000001204	DNAJC6	No change
43	ENSMODG00000003165	EML5	No change
44	ENSMODG00000012167	ENSMODT00000015523	No change
45	ENSMODG00000016753	LAMA5	No change
46	ENSMODG00000004723	ABCA4	No change
48	ENSMODG00000024416	LRRN1	No change
50	ENSMODG00000008745	SIPA1L2	No change
51	ENSMODG00000005525	SUSD1	No change
52	ENSMODG00000010795	ASXL3	No change

54	ENSMODG00000016837	CCDC3	No change
55	ENSMODG00000018351	NRIP2	No change
56	ENSMODG00000008641	ALDH1A2	No change
57	ENSMODG00000014164	NDRG4	No change
59	ENSMODG00000012283	SEMA3F	No change
60	ENSMODG00000027597	ENSMODT00000043238	No change
61	ENSMODG00000014736	PLCL2	No change
62	ENSMODG00000010505	FZD1	No change
63	ENSMODG00000014827	ZNF385D	No change
64	ENSMODG00000017647	MYCL	No change
65	ENSMODG00000000401	LPAR3	No change
66	ENSMODG00000006840	MATN2	No change
67	ENSMODG00000021517	CDH2	No change
68	ENSMODG00000005826	RBFOX1	No change
69	ENSMODG00000005626	PAPPA2	No change
70	ENSMODG00000029138	ENSMODT00000042628	No change
71	ENSMODG00000000708	KCTD17	No change
72	ENSMODG00000001368	SP4	No change
73	ENSMODG00000003412	ZBTB7C	No change
74	ENSMODG00000009548	MPPED2	No change
75	ENSMODG00000011428	FZD2	No change
76	ENSMODG00000017097	RHOBTB1	No change
77	ENSMODG00000002970	ANKH	No change
78	ENSMODG00000014943	PSIP1	No change
82	ENSMODG00000028613	C11orf97	No change
85	ENSMODG00000008094	KIF24	No change
86	ENSMODG00000003575	RND3	No change
87	ENSMODG00000006244	LTBP2	No change
88	ENSMODG00000018215	FAM234B	No change
89	ENSMODG00000020249	HAPLN1	No change
91	ENSMODG00000003526	KIF5C	No change
92	ENSMODG00000004667	TENM4	No change
93	ENSMODG00000023254	C20orf85	No change
94	ENSMODG00000006619	CCDC151	No change
95	ENSMODG00000009808	PML	No change
96	ENSMODG00000015619	PIWIL1	No change
97	ENSMODG00000019079	PRAG1	No change
98	ENSMODG00000005251	UGT8	No change
99	ENSMODG00000016104	FAM13C	No change
100	ENSMODG00000007165	SLIT3	No change
101	ENSMODG00000012607	PCGF2	No change
102	ENSMODG00000020423	CCBE1	No change
103	ENSMODG00000001741	TRIB1	No change
104	ENSMODG00000006352	CBFB	No change
106	ENSMODG00000001859	SLC41A1	No change
107	ENSMODG00000001982	AP3B2	No change
109	ENSMODG00000013667	ADSSL1	No change
110	ENSMODG00000008914	OTUD1	No change
111	ENSMODG00000001716	EVC2	No change
112	ENSMODG00000012765	ENSMODT00000016266	No change
114	ENSMODG00000004676	KIRREL3	No change
115	ENSMODG00000008525	FGFR2	No change

116	ENSMODG00000007264	ATP1B2	No change
117	ENSMODG00000020935	KRT7	No change
118	ENSMODG00000017492	ENSMODT00000022204	No change
119	ENSMODG00000008625	RASSF10	No change
120	ENSMODG00000002419	C3orf67	No change
122	ENSMODG00000017333	FOXO6	No change
123	ENSMODG00000019478	CDC20B	No change
125	ENSMODG00000011835	APBA2	No change
126	ENSMODG00000020670	PDGFRA	No change
127	ENSMODG00000004634	FAM92B	No change
129	ENSMODG00000003958	COL27A1	No change
130	ENSMODG00000009614	HR	No change
131	ENSMODG00000001422	SCG5	No change
132	ENSMODG00000016217	NRTN	No change
133	ENSMODG00000017145	RAI2	No change
134	ENSMODG00000000596	TTLL7	No change
136	ENSMODG00000008896	ENSMODT00000011304	No change
138	ENSMODG00000000330	IVD	No change
140	ENSMODG00000000449	HOXA13	No change
141	ENSMODG00000000925	DYNC2H1	No change
142	ENSMODG00000015159	GADL1	No change
143	ENSMODG00000019395	IL16	No change
144	ENSMODG00000007431	TMEM67	No change
145	ENSMODG00000020368	PPP1R32	No change
147	ENSMODG00000016919	FAM107B	No change
148	ENSMODG00000017408	KIAA1522	No change
150	ENSMODG00000010733	ENSMODT00000013692	No change
151	ENSMODG00000001292	TIMP3	No change
152	ENSMODG00000028520	ENSMODT00000042200	No change
153	ENSMODG00000020620	NPNT	No change
154	ENSMODG00000009758	SFI1	No change
155	ENSMODG00000025488	ZBBX	No change
156	ENSMODG00000021112	LANCL3	No change
157	ENSMODG00000006984	ENSMODT00000008828	No change
158	ENSMODG00000024473	FJX1	No change
160	ENSMODG00000019734	CRB2	No change
161	ENSMODG00000012254	CUX2	No change
162	ENSMODG00000007986	EDARADD	No change
163	ENSMODG00000019380	BNC1	No change
164	ENSMODG00000019472	ENSMODT00000024722	No change
165	ENSMODG00000010772	CDK6	No change
166	ENSMODG00000003251	CPN1	No change
167	ENSMODG00000000245	GLI2	No change
169	ENSMODG00000018545	MB21D1	No change
171	ENSMODG00000002180	ENSMODT00000002707	No change
172	ENSMODG00000012124	REEP2	No change
173	ENSMODG00000008336	FGF9	No change
174	ENSMODG00000012367	ZNF507	No change
176	ENSMODG00000002618	ST6GAL2	No change
177	ENSMODG00000025710	ENSMODT00000040154	No change
178	ENSMODG00000016377	TFAP4	No change
179	ENSMODG00000017553	MOXD1	No change

180	ENSMODG00000008513	DKK3	No change
181	ENSMODG00000003477	RETREG1	No change
182	ENSMODG00000018129	KLHL32	No change
183	ENSMODG00000021051	RUNX1	No change
184	ENSMODG00000013022	KLHDC8B	No change
185	ENSMODG00000009034	LARP6	No change
186	ENSMODG00000001388	ITGB8	No change
188	ENSMODG00000009976	IGSF8	No change
190	ENSMODG00000009283	RHBDF2	No change
192	ENSMODG00000001045	ZNF608	No change
193	ENSMODG00000009884	SLC6A4	No change
194	ENSMODG00000000574	GPR37L1	No change
196	ENSMODG00000009155	GALNT2	No change
197	ENSMODG00000009623	ANKRD13B	No change
198	ENSMODG00000017574	INPP5E	No change
199	ENSMODG00000017877	COL8A2	No change
201	ENSMODG00000015523	LBH	No change
202	ENSMODG00000013460	SNCAIP	No change
203	ENSMODG00000004944	DNAAF1	No change
204	ENSMODG00000020906	MYCBPAP	No change
205	ENSMODG00000009618	CACNB2	No change
207	ENSMODG00000010244	IGDCC4	No change
208	ENSMODG00000010667	DTNA	No change
209	ENSMODG00000017780	MAN1A1	No change
210	ENSMODG00000017245	SLC26A5	No change
211	ENSMODG00000003143	TMOD1	No change
214	ENSMODG00000007439	NRP1	No change
215	ENSMODG00000001370	SPATA6	No change
216	ENSMODG00000015295	GPC4	No change
218	ENSMODG00000014019	FGF13	No change
219	ENSMODG00000014705	ELMOD1	No change
220	ENSMODG00000002659	C3orf70	No change
221	ENSMODG00000020376	PRLR	No change
222	ENSMODG00000010734	FAM124A	No change
224	ENSMODG00000017623	L3MBTL3	No change
225	ENSMODG00000028100	LRRC27	No change
226	ENSMODG00000001212	RET	No change
227	ENSMODG00000007971	ENSMODT00000010120	No change
229	ENSMODG00000006079	OLFML1	No change
230	ENSMODG00000016879	RAB32	No change
231	ENSMODG00000006427	DLG4	No change
232	ENSMODG00000018362	CEP162	No change
233	ENSMODG00000029084	ENSMODT00000041974	No change
234	ENSMODG00000017570	COL16A1	No change
235	ENSMODG00000018314	ENSMODT00000023242	No change
236	ENSMODG00000013187	ENSMODT00000016807	No change
237	ENSMODG00000004499	CDON	No change
239	ENSMODG00000005461	LZTS3	No change
240	ENSMODG00000003582	PRRX1	No change
241	ENSMODG00000006766	GABRP	No change
242	ENSMODG00000021269	PFN2	No change
243	ENSMODG00000004939	TMEM74	No change

245	ENSMODG00000004229	MCM7	No change
246	ENSMODG00000025411	BBS12	No change
247	ENSMODG00000008268	TTPA	No change
248	ENSMODG00000008897	FAM81A	No change
249	ENSMODG00000009388	EMX2	No change
250	ENSMODG00000021492	GATA6	No change
251	ENSMODG00000002474	SPOCK3	No change
252	ENSMODG00000004940	PLS3	No change
253	ENSMODG00000013654	SSC4D	No change
254	ENSMODG00000001133	RASD2	No change
255	ENSMODG00000002842	SEMA6D	No change
256	ENSMODG00000017476	AHI1	No change
257	ENSMODG00000003098	SH3TC1	No change
259	ENSMODG00000013653	RAB19	No change
261	ENSMODG00000005482	ENSMODT00000006940	No change
262	ENSMODG00000010526	NEDD9	No change
263	ENSMODG00000010272	RUNDC3B	No change
264	ENSMODG00000019410	NAB2	No change
265	ENSMODG00000008531	ENSMODT00000010824	No change
266	ENSMODG00000019377	TM6SF1	No change
267	ENSMODG00000001196	WNT2B	No change
268	ENSMODG00000023920	SFN	No change
269	ENSMODG00000012089	PSTPIP1	No change
270	ENSMODG00000005342	ENSMODT00000006728	No change
271	ENSMODG00000017264	ASB9	No change
272	ENSMODG00000004124	DCTD	No change
273	ENSMODG00000020128	ENSMODT00000025605	No change
274	ENSMODG00000008351	ASPH	No change
275	ENSMODG00000014314	NCAM1	No change
276	ENSMODG00000007385	LMO1	No change
277	ENSMODG00000017211	LRRC17	No change
278	ENSMODG00000016040	NRCAM	No change
279	ENSMODG00000014910	PRRT1	No change
280	ENSMODG00000016202	RIMS4	No change
281	ENSMODG00000004815	SLC46A2	No change
282	ENSMODG00000027531	ENSMODT00000043033	No change
283	ENSMODG00000007813	FBF1	No change
284	ENSMODG00000017948	DLGAP3	No change
287	ENSMODG00000014758	KLF11	No change
288	ENSMODG00000008302	MN1	No change
289	ENSMODG00000004544	ENSMODT00000005722	No change
290	ENSMODG00000013033	SARDH	No change
292	ENSMODG00000021278	MED12L	No change
293	ENSMODG00000024684	ENSMODT00000036736	No change
296	ENSMODG00000014851	HLF	No change
298	ENSMODG00000019447	DNMT3B	No change
299	ENSMODG00000013636	KIF26A	No change
300	ENSMODG00000015192	PTPRD	No change
301	ENSMODG00000008605	EMID1	No change
302	ENSMODG00000023071	ENSMODT00000029739	No change
303	ENSMODG00000017625	EGLN3	No change
304	ENSMODG00000013107	ENSMODT00000016704	No change

305	ENSMODG00000015705	TRIM36	No change
306	ENSMODG00000001523	ADAMTS16	No change
307	ENSMODG00000002360	DCBLD2	No change
308	ENSMODG00000019066	MICALL1	No change
311	ENSMODG00000002718	ENSMODT00000003366	No change
312	ENSMODG00000015095	IL1RAP	No change
313	ENSMODG00000012577	SRCIN1	No change
314	ENSMODG00000010980	UNC5D	No change
315	ENSMODG00000019252	ENSMODT00000024460	No change
316	ENSMODG00000007642	ENSMODT00000009674	No change
318	ENSMODG00000012697	KIAA1551	No change
320	ENSMODG00000000092	TYRO3	No change
321	ENSMODG00000005452	TSTD1	No change
322	ENSMODG00000011471	ENSMODT00000014605	No change
323	ENSMODG00000013416	PHLDB1	No change
325	ENSMODG00000001828	MTSS1	No change
326	ENSMODG00000002751	SLC25A24	No change
327	ENSMODG00000017905	FYN	No change
328	ENSMODG00000005818	DACT2	No change
329	ENSMODG00000013600	ORAI2	No change
330	ENSMODG00000010745	MIIP	No change
333	ENSMODG00000006146	STC2	No change
334	ENSMODG00000014532	SH3BP5	No change
335	ENSMODG00000003795	ENSMODT00000004746	No change
337	ENSMODG00000001398	RFX2	No change
338	ENSMODG00000010516	GRK3	No change
340	ENSMODG00000004698	LRP2BP	No change
341	ENSMODG00000007628	CALB1	No change
342	ENSMODG00000000104	MAML2	No change
343	ENSMODG00000020525	C4orf19	No change
344	ENSMODG00000003396	MEIS1	No change
345	ENSMODG00000027824	ENSMODT00000043713	No change
346	ENSMODG00000007447	ENSMODT00000036224	No change
347	ENSMODG00000013180	RPGRIP1L	No change
348	ENSMODG00000015316	GPR37	No change
349	ENSMODG00000024021	RPRML	No change
350	ENSMODG00000018720	RAB23	No change
353	ENSMODG00000009045	FLT3	No change
354	ENSMODG00000019449	BLM	No change
355	ENSMODG00000009958	ATP1A2	No change
356	ENSMODG00000020444	GLIPR1L1	No change
357	ENSMODG00000009882	POC1B	No change
359	ENSMODG00000003315	ENSMODT00000004139	No change
360	ENSMODG00000017448	MARCKSL1	No change
361	ENSMODG00000000012	SPOCK2	No change
362	ENSMODG00000005062	ENSMODT00000006356	No change
365	ENSMODG00000028233	ENSMODT00000043488	No change
366	ENSMODG00000018569	ESYT3	No change
367	ENSMODG00000009889	ENSMODT00000012603	No change
368	ENSMODG00000018631	ENSMODT00000023648	No change
369	ENSMODG00000005256	EBF4	No change
370	ENSMODG00000020182	DTX4	No change

372	ENSMODG00000012335	MST1R	No change
373	ENSMODG00000001264	LARGE1	No change
374	ENSMODG00000002847	LRP1B	No change
376	ENSMODG00000024523	WDR90	No change
377	ENSMODG00000001088	ST3GAL1	No change
378	ENSMODG00000010585	FCHSD1	No change
379	ENSMODG00000008607	MAP10	No change
381	ENSMODG00000008675	ZNF395	No change
382	ENSMODG00000017230	CCDC30	No change
383	ENSMODG00000015099	GMNC	No change
384	ENSMODG00000008005	ADHFE1	No change
385	ENSMODG00000007407	CHST15	No change
386	ENSMODG00000005078	PAK1	No change
387	ENSMODG00000016980	ENSMODT00000021555	No change
388	ENSMODG00000002722	PTPRG	No change
390	ENSMODG00000017204	ENSMODT00000035586	No change
391	ENSMODG00000010747	STOX1	No change
392	ENSMODG00000005475	NGB	No change
393	ENSMODG00000001080	ENSMODT00000001311	No change
394	ENSMODG00000013846	LIMK1	No change
396	ENSMODG00000002485	LAMB3	No change
397	ENSMODG00000008701	FZD3	No change
399	ENSMODG00000012220	COL13A1	No change
400	ENSMODG00000019039	PCM1	No change
401	ENSMODG00000021462	TGIF1	No change
402	ENSMODG00000028035	ENSMODT00000042540	No change
403	ENSMODG00000006257	PLPPR3	No change
404	ENSMODG00000017096	ZNF362	No change
405	ENSMODG00000001108	UHRF1	No change
406	ENSMODG00000000232	SDK1	No change
407	ENSMODG00000006902	PI15	No change
408	ENSMODG00000012120	INTU	No change
409	ENSMODG00000002005	EFEMP1	No change
410	ENSMODG00000019829	PAMR1	No change
411	ENSMODG00000013541	TNFAIP8	No change
412	ENSMODG00000019898	LRP4	No change
413	ENSMODG00000009522	IGFBP3	No change
414	ENSMODG00000024533	ENSMODT00000036075	No change
415	ENSMODG00000011703	WDHD1	No change
416	ENSMODG00000011495	MYL2	No change
418	ENSMODG00000018935	ENSMODT00000024045	No change
419	ENSMODG00000008718	SLC29A3	No change
420	ENSMODG00000015511	ENSMODT00000019709	No change
421	ENSMODG00000020538	KLHL5	No change
423	ENSMODG00000014038	ARHGAP33	No change
424	ENSMODG00000015094	ANO8	No change
425	ENSMODG00000019920	AGBL2	No change
426	ENSMODG00000024066	FAM237B	No change
427	ENSMODG00000016133	MECOM	No change
429	ENSMODG00000010926	DAPK2	No change
431	ENSMODG00000010393	PARP10	No change
432	ENSMODG00000024114	ENSMODT00000034699	No change

433	ENSMODG00000009362	GPR155	No change
434	ENSMODG00000008271	ENSMODT00000010477	No change
435	ENSMODG00000017881	LAMA4	No change
437	ENSMODG00000016642	ENSMODT00000021134	No change
438	ENSMODG00000016001	MDH1B	No change
439	ENSMODG00000015388	STK36	No change
440	ENSMODG00000004891	PKHD1L1	No change
441	ENSMODG00000000247	SRC	No change
442	ENSMODG00000006660	ARHGEF40	No change
444	ENSMODG00000012209	LRP3	No change
446	ENSMODG00000016680	DPYSL2	No change
447	ENSMODG00000002282	CD55	No change
449	ENSMODG00000003357	DTX1	No change
450	ENSMODG00000016860	CHRNA4	No change
452	ENSMODG00000000995	OLFML2B	No change
453	ENSMODG00000006856	ENSMODT00000008663	No change
454	ENSMODG00000023435	ENSMODT00000031422	No change
455	ENSMODG00000020835	CADM4	No change
456	ENSMODG00000021179	MEGF6	No change
457	ENSMODG00000006601	RGL3	No change
458	ENSMODG00000008291	GGH	No change
459	ENSMODG00000019050	SLC7A2	No change
460	ENSMODG00000000206	RIT2	No change
461	ENSMODG00000003309	EPHA2	No change
462	ENSMODG00000012570	ARHGAP23	No change
463	ENSMODG00000014739	ENSMODT00000018757	No change
464	ENSMODG00000017687	NCOA7	No change
465	ENSMODG00000000006	INAVA	No change
466	ENSMODG00000001144	ENSMODT00000001391	No change
467	ENSMODG00000004209	ENSMODT00000005300	No change
468	ENSMODG00000006971	ENSMODT00000008812	No change
469	ENSMODG00000010440	CFAP69	No change
470	ENSMODG00000006180	TDRD5	No change
472	ENSMODG00000007138	PLEKHA7	No change
473	ENSMODG00000025181	ENSMODT00000038890	No change
474	ENSMODG00000007653	ARHGEF17	No change
475	ENSMODG00000023614	ENSMODT00000032291	No change
476	ENSMODG00000020350	ENSMODT00000025903	No change
478	ENSMODG00000016101	EPHA8	No change
479	ENSMODG00000016002	PASK	No change
481	ENSMODG00000013732	HIP1	No change
482	ENSMODG00000000987	PROM1	No change
483	ENSMODG00000020394	RAI14	No change
484	ENSMODG00000003443	CCDC57	No change
486	ENSMODG00000003606	TNFAIP6	No change
487	ENSMODG00000012131	WDR66	No change
488	ENSMODG00000008517	PDGFA	No change
490	ENSMODG00000017516	TCF21	No change
491	ENSMODG00000009529	ARL14EP	No change
492	ENSMODG00000027318	ENSMODT00000042611	No change
494	ENSMODG00000002766	TERF2	No change
495	ENSMODG00000000975	KHDRBS3	No change

496	ENSMODG00000013104	EVL	No change
497	ENSMODG00000002769	PTCH1	No change
498	ENSMODG00000023651	ENSMODT00000032504	No change
499	ENSMODG00000010899	RBM24	No change
500	ENSMODG00000012118	ENSMODT00000015457	No change
501	ENSMODG00000013154	BEGAIN	No change
502	ENSMODG00000012112	DNAH7	No change
504	ENSMODG00000009072	NAV2	No change
505	ENSMODG00000011624	CIB2	No change
506	ENSMODG00000017852	FRK	No change
507	ENSMODG00000016601	CTSZ	No change
508	ENSMODG00000001102	CC2D2A	No change
510	ENSMODG00000010833	EBPL	No change
512	ENSMODG00000019155	TMEM132E	No change
513	ENSMODG00000001008	MOV10	No change
514	ENSMODG00000005361	ENSMODT00000006752	No change
515	ENSMODG00000008126	USP44	No change
517	ENSMODG00000006623	TP73	No change
518	ENSMODG00000017783	FAM184A	No change
519	ENSMODG00000001182	INPP4A	No change
520	ENSMODG00000015065	CCDC136	No change
521	ENSMODG00000023649	ENSMODT00000032486	No change
522	ENSMODG00000012037	CHSY1	No change
523	ENSMODG00000018508	AMOTL2	No change
525	ENSMODG00000008623	FRMD3	No change
526	ENSMODG00000006108	ENSMODT00000007722	No change
527	ENSMODG00000015389	IQUB	No change
528	ENSMODG00000010864	RNF125	No change
529	ENSMODG00000010153	ENSMODT00000012944	No change
530	ENSMODG00000003438	FBXL7	No change
531	ENSMODG00000000713	ADGRL2	No change
532	ENSMODG00000011134	ULK4	No change
533	ENSMODG00000011476	SEMA3G	No change
534	ENSMODG00000020254	ATP6AP1L	No change
535	ENSMODG00000007423	DNAH2	No change
536	ENSMODG00000015150	BRCA1	No change
537	ENSMODG00000005845	RIPOR1	No change
538	ENSMODG00000015744	TIPARP	No change
539	ENSMODG00000003898	LRRN4	No change
540	ENSMODG00000000582	ADGRA3	No change
541	ENSMODG00000020516	DUSP6	No change
542	ENSMODG00000011456	KLHDC1	No change
543	ENSMODG00000021189	EPHA4	No change
544	ENSMODG00000029424	ENSMODT00000031563	No change
545	ENSMODG00000020580	SHISA3	No change
546	ENSMODG00000007032	ERI2	No change
547	ENSMODG00000000989	MCM6	No change
549	ENSMODG00000018218	HEBP1	No change
550	ENSMODG00000002477	TRAF3IP1	No change
551	ENSMODG00000004394	SPON2	No change
552	ENSMODG00000015728	MEGF10	No change
553	ENSMODG00000021279	P2RY14	No change

554	ENSMODG00000012284	ENSMODT00000015673	No change
555	ENSMODG00000009823	PRKRA	No change
556	ENSMODG00000021509	ZNF521	No change
559	ENSMODG00000020895	FGD1	No change
560	ENSMODG00000017817	OSCP1	No change
561	ENSMODG00000007891	MCM2	No change
562	ENSMODG00000001723	PDGFC	No change
563	ENSMODG00000025440	ENSMODT00000039814	No change
564	ENSMODG00000006236	ZNF500	No change
565	ENSMODG00000002241	ENSMODT00000002788	No change
566	ENSMODG00000014734	TSPOAP1	No change
567	ENSMODG00000003264	LRIG1	No change
568	ENSMODG00000004060	ENSMODT00000036028	No change
569	ENSMODG00000007360	TUB	No change
573	ENSMODG00000014244	ENSMODT00000018130	No change
574	ENSMODG00000027554	ENSMODT00000043276	No change
575	ENSMODG00000027792	ENSMODT00000031686	No change
577	ENSMODG00000008835	UGGT2	No change
578	ENSMODG00000014420	NR0B2	No change
579	ENSMODG00000003537	LYPD6B	No change
580	ENSMODG00000011413	LCP1	No change
582	ENSMODG00000003219	IL1RAPL2	No change
583	ENSMODG00000014294	MAP4	No change
584	ENSMODG00000007483	ENSMODT00000009467	No change
585	ENSMODG00000016710	ITIH2	No change
586	ENSMODG00000004326	CNN3	No change
588	ENSMODG00000007188	ENSMODT00000009084	No change
589	ENSMODG00000011507	ENSMODT00000014652	No change
591	ENSMODG00000004445	NONO	No change
592	ENSMODG00000009661	CORO6	No change
593	ENSMODG00000002823	PRMT6	No change
594	ENSMODG00000023326	RSPO1	No change
595	ENSMODG00000019259	ALDOC	No change
596	ENSMODG00000000162	IQCE	No change
597	ENSMODG00000014595	ENSMODT00000018565	No change
598	ENSMODG00000020393	PTPRR	No change
600	ENSMODG00000004997	ENSMODT00000006278	No change
601	ENSMODG00000004673	OXTR	No change
602	ENSMODG00000023431	ENSMODT00000031386	No change
604	ENSMODG00000001072	PDE4B	No change
605	ENSMODG00000000675	ENSMODT00000000825	No change
606	ENSMODG00000011673	ENSMODT00000014894	No change
607	ENSMODG00000007000	RGS6	No change
608	ENSMODG00000004306	ENSMODT00000034994	No change
609	ENSMODG00000015434	NTN3	No change
610	ENSMODG00000016551	BHLHE41	No change
611	ENSMODG00000004216	ENSMODT00000005304	No change
612	ENSMODG00000003939	SVEP1	No change
614	ENSMODG00000006454	FGF22	No change
615	ENSMODG00000012186	B3GNT4	No change
616	ENSMODG00000001166	SPRED1	No change
617	ENSMODG00000027334	ENSMODT00000042182	No change

618	ENSMODG00000006536	KLK11	No change
619	ENSMODG00000015187	ETV4	No change
620	ENSMODG00000020172	LPXN	No change
621	ENSMODG00000002788	NEK2	No change
622	ENSMODG00000011918	PARD6G	No change
623	ENSMODG00000023555	ENSMODT00000031982	No change
624	ENSMODG00000014047	KIFC3	No change
625	ENSMODG00000008747	KRT4	No change
626	ENSMODG00000020450	GLIPR1	No change
628	ENSMODG00000016052	NRP2	No change
630	ENSMODG00000017298	CLIC3	No change
631	ENSMODG00000000577	BMF	No change
632	ENSMODG00000023088	ENSMODT00000029867	No change
634	ENSMODG00000005196	ENSMODT00000006536	No change
635	ENSMODG00000008293	TMCC3	No change
637	ENSMODG00000021205	DOCK10	No change
638	ENSMODG00000018890	SPRY1	No change
639	ENSMODG00000007871	CTC1	No change
641	ENSMODG00000002085	GDF10	No change
643	ENSMODG00000006353	GDF7	No change
644	ENSMODG00000005741	DENND6B	No change
645	ENSMODG00000004489	CSMD3	No change
647	ENSMODG00000003055	BATF3	No change
648	ENSMODG00000017519	NLGN4X	No change
649	ENSMODG00000028335	ENSMODT00000044202	No change
650	ENSMODG00000013220	OLFM1	No change
651	ENSMODG00000001537	AGR3	No change
652	ENSMODG00000005679	SH3TC2	No change
653	ENSMODG00000024658	ENSMODT00000036594	No change
655	ENSMODG00000006231	ENSMODT00000037922	No change
657	ENSMODG00000017425	CLCN4	No change
659	ENSMODG00000019042	TRERF1	No change
660	ENSMODG00000008837	SNX33	No change
661	ENSMODG00000013177	TNS4	No change
662	ENSMODG00000016393	TNNI3	No change
663	ENSMODG00000010567	EDN1	No change
664	ENSMODG00000017618	AKAP6	No change
665	ENSMODG00000017293	RUFY2	No change
666	ENSMODG00000009162	CDCA7	No change
667	ENSMODG00000016969	FBXO41	No change
668	ENSMODG00000001652	Sep-09	No change
669	ENSMODG00000019209	RAB11FIP4	No change
671	ENSMODG00000013865	CADM1	No change
672	ENSMODG00000003849	DHCR24	No change
673	ENSMODG00000019226	FAM13A	No change
674	ENSMODG00000000535	ENSMODT00000000659	No change
675	ENSMODG00000000348	DENND2C	No change
676	ENSMODG00000021485	GREB1L	No change
677	ENSMODG00000006468	KIAA1614	No change
678	ENSMODG00000008842	NINJ1	No change
679	ENSMODG00000024629	GCNT1	No change
680	ENSMODG00000007502	CPXM2	No change

681	ENSMODG00000013467	ANKMY1	No change
682	ENSMODG00000015648	TMEM132C	No change
684	ENSMODG00000025700	ENSMODT00000040137	No change
686	ENSMODG00000017411	SYNC	No change
687	ENSMODG00000006207	TMC7	No change
689	ENSMODG00000004080	ENSMODT00000005129	No change
690	ENSMODG00000029805	ENSMODT00000041857	No change
691	ENSMODG00000008200	ENSMODT00000010387	No change
692	ENSMODG00000018383	RWDD2A	No change
693	ENSMODG00000019555	RHCG	No change
694	ENSMODG00000023131	COL26A1	No change
695	ENSMODG00000012242	B4GALNT2	No change
696	ENSMODG00000008691	FBXO16	No change
697	ENSMODG00000028875	ENSMODT00000043968	No change
698	ENSMODG00000005096	PTGFRN	No change
701	ENSMODG00000003387	KANK1	No change
702	ENSMODG00000011584	COL4A5	No change
706	ENSMODG00000010443	PDE1A	No change
707	ENSMODG00000020710	ENSMODT00000026369	No change
709	ENSMODG00000002595	RECK	No change
711	ENSMODG00000015971	DOCK4	No change
712	ENSMODG00000007528	SLC26A7	No change
713	ENSMODG00000000351	CFAP221	No change
714	ENSMODG00000016639	WSCD1	No change
716	ENSMODG00000019457	ASXL1	No change
717	ENSMODG00000003366	ENSMODT00000004203	No change
718	ENSMODG00000013882	CDH22	No change
719	ENSMODG00000006774	EFCAB6	No change
721	ENSMODG00000021248	ENSMODT00000027035	No change
722	ENSMODG00000003447	EPC2	No change
724	ENSMODG00000014774	ZNF385C	No change
726	ENSMODG00000018698	CYP27C1	No change
727	ENSMODG00000020645	SPATA18	No change
728	ENSMODG00000005351	CPLX2	No change
729	ENSMODG00000007713	FCHSD2	No change
730	ENSMODG00000006750	PPP1R3C	No change
732	ENSMODG00000025606	PLEKHG4B	No change
733	ENSMODG00000015966	KIF17	No change
734	ENSMODG00000005359	ZFPM2	No change
735	ENSMODG00000013975	NPHS1	No change
737	ENSMODG00000019503	TTLL13P	No change
738	ENSMODG00000011537	ENSMODT00000014708	No change
739	ENSMODG00000001473	ENSMODT00000001826	No change
740	ENSMODG00000004819	DISP1	No change
741	ENSMODG00000004950	HSDL1	No change
742	ENSMODG00000016310	FZD7	No change
743	ENSMODG00000016186	SDC4	No change
744	ENSMODG00000000268	RAD51	No change
745	ENSMODG00000003911	MARVELD1	No change
746	ENSMODG00000005317	CAMK2D	No change
747	ENSMODG00000014161	WDR62	No change
748	ENSMODG00000009511	ENSMODT00000012105	No change

749	ENSMODG00000015906	UBXN10	No change
751	ENSMODG00000018378	SLC6A12	No change
752	ENSMODG00000008795	WASF3	No change
753	ENSMODG00000005835	ENSMODT00000007371	No change
756	ENSMODG00000025055	GALNT7	No change
757	ENSMODG00000016195	BAIAP3	No change
758	ENSMODG00000004236	IFT74	No change
759	ENSMODG00000015238	UHRF2	No change
760	ENSMODG00000023937	KCNJ16	No change
761	ENSMODG00000009211	PTGIR	No change
762	ENSMODG00000018968	ENSMODT00000024151	No change
763	ENSMODG00000014305	ENSMODT00000018207	No change
764	ENSMODG00000011549	TCTN1	No change
765	ENSMODG00000007284	FBLN1	No change
767	ENSMODG00000021446	TWSG1	No change
768	ENSMODG00000001926	NUAK1	No change
769	ENSMODG00000004086	ENSMODT00000005136	No change
770	ENSMODG00000010064	FAM171A1	No change
771	ENSMODG00000004287	ENSMODT00000005394	No change
772	ENSMODG00000008425	CYFIP2	No change
774	ENSMODG00000020465	E2F7	No change
775	ENSMODG00000020396	ENSMODT00000025964	No change
777	ENSMODG00000009542	ENSMODT00000012147	No change
778	ENSMODG00000001730	ENSMODT00000002164	No change
780	ENSMODG00000025403	MCIDAS	No change
781	ENSMODG00000016005	ADAM23	No change
782	ENSMODG00000009230	LIF	No change
783	ENSMODG00000014069	ITPR3	No change
784	ENSMODG00000000464	KNSTRN	No change
786	ENSMODG00000014986	FREM1	No change
788	ENSMODG00000002667	FOLH1	No change
789	ENSMODG00000000724	TST	No change
790	ENSMODG00000001282	KCND3	No change
791	ENSMODG00000012394	UBA7	No change
792	ENSMODG00000022866	LPAR4	No change
793	ENSMODG00000011322	MBOAT1	No change
794	ENSMODG00000011449	TNNC1	No change
795	ENSMODG00000004413	RGS14	No change
796	ENSMODG00000015471	PTPRZ1	No change
798	ENSMODG00000010682	DGAT1	No change
800	ENSMODG00000002870	GPR161	No change
802	ENSMODG00000002557	CLCN2	No change
803	ENSMODG00000021407	DSEL	No change
804	ENSMODG00000010591	ENSMODT00000013509	No change
805	ENSMODG00000006125	ENSMODT00000007740	No change
808	ENSMODG00000003569	ENSMODT00000004453	No change
809	ENSMODG00000007857	CENPJ	No change
810	ENSMODG00000011360	GJC1	No change
811	ENSMODG00000003690	COL14A1	No change
813	ENSMODG00000017084	ADGRG2	No change
814	ENSMODG00000000906	COMTD1	No change
815	ENSMODG00000020648	RASL11B	No change

816	ENSMODG00000013904	ENSMODT00000017714	No change
817	ENSMODG00000002074	PSRC1	No change
818	ENSMODG00000017488	MYB	No change
819	ENSMODG00000003377	DAB1	No change
820	ENSMODG00000002297	HECW1	No change
821	ENSMODG00000006227	TMC5	No change
822	ENSMODG00000020619	CORIN	No change
823	ENSMODG00000008702	GALNT10	No change
825	ENSMODG00000020869	ENSMODT00000026561	No change
826	ENSMODG00000014882	TPM4	No change
827	ENSMODG00000002538	FHL2	No change
828	ENSMODG00000007159	TTC9	No change
830	ENSMODG00000000217	PIWIL4	No change
831	ENSMODG00000015093	ENSMODT00000019209	No change
832	ENSMODG00000004069	KCNS3	No change
833	ENSMODG00000021455	ARHGAP28	No change
834	ENSMODG00000016114	EPHB2	No change
835	ENSMODG00000021256	CPA3	No change
836	ENSMODG00000007630	CHST13	No change
837	ENSMODG00000008144	TEX9	No change
838	ENSMODG00000002089	RBFOX3	No change
839	ENSMODG00000004924	BTBD3	No change
840	ENSMODG00000009620	SMAD6	No change
841	ENSMODG00000020564	NSUN7	No change
842	ENSMODG00000014040	EXTL1	No change
843	ENSMODG00000015829	CCHCR1	No change
844	ENSMODG00000019401	CFAP161	No change
845	ENSMODG00000018397	B4GALNT3	No change
847	ENSMODG00000006106	SHANK3	No change
848	ENSMODG00000004433	FGFRL1	No change
849	ENSMODG00000020369	LRRC10B	No change
850	ENSMODG00000006980	GDF6	No change
851	ENSMODG00000024106	TRIM13	No change
853	ENSMODG00000008806	CDKL4	No change
854	ENSMODG00000014335	ENSMODT00000018249	No change
855	ENSMODG00000017371	ARHGAP6	No change
857	ENSMODG00000025309	ZBED2	No change
860	ENSMODG00000010363	MAK	No change
861	ENSMODG00000027874	ENSMODT00000043334	No change
862	ENSMODG00000024019	PLEKHB1	No change
863	ENSMODG00000019604	ASTN2	No change
864	ENSMODG00000016756	FBXL16	No change
865	ENSMODG00000003813	NEIL3	No change
866	ENSMODG00000016956	KIAA1324L	No change
867	ENSMODG00000011099	SPNS2	No change
868	ENSMODG00000016489	SGMS1	No change
869	ENSMODG00000027983	ENSMODT00000042022	No change
870	ENSMODG00000027755	ENSMODT00000043065	No change
872	ENSMODG00000028627	ENSMODT00000007321	No change
873	ENSMODG00000008306	FOXJ1	No change
875	ENSMODG00000019158	B3GNT3	No change
876	ENSMODG00000016334	ENSMODT00000020770	No change

877	ENSMODG00000029059	ENSMODT00000042837	No change
878	ENSMODG00000002117	CHST11	No change
879	ENSMODG00000016203	KCNK15	No change
880	ENSMODG00000000034	PCDH7	No change
881	ENSMODG00000020456	ENSMODT00000026049	No change
884	ENSMODG00000007354	POLA1	No change
885	ENSMODG00000003525	SLC24A2	No change
886	ENSMODG00000012246	CEP89	No change
887	ENSMODG00000014671	LDHD	No change
888	ENSMODG00000006462	IFT122	No change
890	ENSMODG00000017995	PHC1	No change
891	ENSMODG00000012760	ENSMODT00000016259	No change
892	ENSMODG00000005722	ENSMODT00000007235	No change
893	ENSMODG00000019027	MFHAS1	No change
894	ENSMODG00000001399	SLC5A9	No change
895	ENSMODG00000000951	WDR78	No change
896	ENSMODG00000001932	PCSK5	No change
898	ENSMODG00000001783	EML6	No change
899	ENSMODG00000002716	ENSMODT00000003365	No change
900	ENSMODG00000001827	CSF1	No change
901	ENSMODG00000017143	MAGI2	No change
903	ENSMODG00000018736	BCL9	No change
904	ENSMODG00000025337	HPDL	No change
905	ENSMODG00000014073	ARHGEF6	No change
906	ENSMODG00000004065	DPYD	No change
907	ENSMODG00000015077	ABHD8	No change
913	ENSMODG00000001528	ENSMODT00000003546	No change
914	ENSMODG00000014526	CEP41	No change
915	ENSMODG00000005067	TNFSF13B	No change
917	ENSMODG00000004171	WWC2	No change
918	ENSMODG00000028175	ENSMODT00000042582	No change
919	ENSMODG00000002905	ENSMODT00000003616	No change
920	ENSMODG00000004650	EDA	No change
921	ENSMODG00000000407	ZFP36L2	No change
922	ENSMODG00000012291	ENSMODT00000015676	No change
923	ENSMODG00000015539	TOGARAM2	No change
924	ENSMODG00000006626	AKT3	No change
925	ENSMODG00000017058	TCEA2	No change
926	ENSMODG00000007441	SULF1	No change
927	ENSMODG00000012038	KCTD15	No change
928	ENSMODG00000010787	KCNRG	No change
929	ENSMODG00000002402	FGF7	No change
930	ENSMODG00000008922	SIN3A	No change
931	ENSMODG00000016900	SHPRH	No change
932	ENSMODG00000011540	ADAMTS7	No change
933	ENSMODG00000007051	SLCO2B1	No change
934	ENSMODG00000019409	NEMP1	No change
935	ENSMODG00000008918	ENSMODT00000001705	No change
936	ENSMODG00000025139	ENSMODT00000003717	No change
938	ENSMODG00000000509	PGR	No change
939	ENSMODG00000010497	CDK14	No change
940	ENSMODG00000012988	ZNF423	No change

941	ENSMODG00000003380	SPRED2	No change
942	ENSMODG00000029139	ENSMODT00000043248	No change
944	ENSMODG00000009914	NBEA	No change
945	ENSMODG00000003401	DMRT1	No change
946	ENSMODG00000011044	TMEM200B	No change
947	ENSMODG00000009057	MAPK15	No change
948	ENSMODG00000016823	COL9A3	No change
949	ENSMODG00000007333	ENSMODT00000009276	No change
950	ENSMODG00000006701	CEP170	No change
951	ENSMODG00000008640	SIX1	No change
953	ENSMODG00000000583	TTC29	No change
954	ENSMODG00000014104	SYNGAP1	No change
955	ENSMODG00000016180	ENSMODT00000020581	No change
956	ENSMODG00000016232	SLC2A10	No change
957	ENSMODG00000002604	SERTAD4	No change
958	ENSMODG00000015348	CFAP65	No change
959	ENSMODG00000001769	ENSMODT00000002208	No change
960	ENSMODG00000019542	WDR93	No change
961	ENSMODG00000010683	CD83	No change
962	ENSMODG00000020402	RXFP3	No change
963	ENSMODG00000020822	VSIG10L	No change
964	ENSMODG00000011608	ENSMODT00000014793	No change
965	ENSMODG00000013085	LRFN5	No change
967	ENSMODG00000014802	GHDC	No change
968	ENSMODG00000019857	ENSMODT00000025217	No change
969	ENSMODG00000009125	ENSMODT00000011629	No change
970	ENSMODG00000018714	KHDRBS2	No change
971	ENSMODG00000012172	NPFFR1	No change
972	ENSMODG00000004153	CHL1	No change
973	ENSMODG00000023922	CEP112	No change
974	ENSMODG00000012155	LAMC3	No change
976	ENSMODG00000024705	PIEZO2	No change
977	ENSMODG00000006020	SLC9A5	No change
978	ENSMODG00000018688	ZNF697	No change
979	ENSMODG00000013138	TMEM136	No change
980	ENSMODG00000002327	ENSMODT00000002892	No change
981	ENSMODG00000006400	RRAD	No change
983	ENSMODG00000016471	PRKG1	No change
984	ENSMODG00000018576	ENSMODT00000023586	No change
985	ENSMODG00000027333	ENSMODT00000043040	No change
986	ENSMODG00000006320	FBXO43	No change
987	ENSMODG00000007823	ENSMODT00000009994	No change
988	ENSMODG00000006795	SLC35E2B	No change
989	ENSMODG00000009524	N4BP2L1	No change
990	ENSMODG00000009575	CBX6	No change
991	ENSMODG00000015875	NRM	No change
992	ENSMODG00000013660	INF2	No change
993	ENSMODG00000010665	FAM171B	No change
994	ENSMODG00000018357	NT5E	No change
995	ENSMODG00000013463	LRFN1	No change
997	ENSMODG00000005257	EFNA2	No change
998	ENSMODG00000015017	CNTNAP1	No change

999	ENSMODG00000017155	KCNN3	No change
1000	ENSMODG00000016636	STK38L	No change
1001	ENSMODG00000024864	ENSMODT00000037538	No change
1002	ENSMODG00000008712	TOX	No change
1003	ENSMODG00000005819	CNGA2	No change
1004	ENSMODG00000016982	PROS1	No change
1005	ENSMODG00000024048	SPATA7	No change
1006	ENSMODG00000013593	SH2B2	No change
1007	ENSMODG00000011374	TIE1	No change
1009	ENSMODG00000024135	ENSMODT00000034864	No change
1010	ENSMODG00000016687	ITIH5	No change
1011	ENSMODG00000015494	ENSMODT00000019685	No change
1012	ENSMODG00000013569	TMPRSS13	No change
1013	ENSMODG00000016665	AXIN1	No change
1015	ENSMODG00000010496	OAZ2	No change
1016	ENSMODG00000024064	ENSMODT00000034372	No change
1017	ENSMODG00000014306	CDC25A	No change
1018	ENSMODG00000001903	ENSMODT00000002373	No change
1019	ENSMODG00000018186	IGSF11	No change
1020	ENSMODG00000013599	CCDC13	No change
1021	ENSMODG00000023129	ENSMODT00000030096	No change
1022	ENSMODG00000012308	CCDC62	No change
1023	ENSMODG00000020821	IGLON5	No change
1024	ENSMODG00000017486	ENSMODT00000022195	No change
1025	ENSMODG00000029694	ENSMODT00000043489	No change
1026	ENSMODG00000019170	KCNN1	No change
1027	ENSMODG00000010941	KIFC2	No change
1028	ENSMODG00000013723	TBXAS1	No change
1029	ENSMODG00000018321	CGA	No change
1030	ENSMODG00000008311	TCF12	No change
1032	ENSMODG00000010852	ENSMODT00000031583	No change
1034	ENSMODG00000010243	RAMP3	No change
1035	ENSMODG00000006358	PALM	No change
1036	ENSMODG00000021148	ZNF641	No change
1037	ENSMODG00000024644	SPTSSB	No change
1039	ENSMODG00000017954	GJB4	No change
1040	ENSMODG00000010488	TLCD2	No change
1041	ENSMODG00000010810	ENSMODT00000013802	No change
1042	ENSMODG00000024055	ENSMODT00000034336	No change
1043	ENSMODG00000015755	ERBB4	No change
1044	ENSMODG00000014238	TDRP	No change
1047	ENSMODG00000020476	PTPRQ	No change
1049	ENSMODG00000014863	STXBP4	No change
1050	ENSMODG00000000731	SLIT2	No change
1051	ENSMODG00000008456	KIF27	No change
1052	ENSMODG00000021106	ENSMODT00000026856	No change
1053	ENSMODG00000013839	ENSMODT00000017620	No change
1054	ENSMODG00000006002	ZC2HC1C	No change
1055	ENSMODG00000016683	LRRC71	No change
1056	ENSMODG00000013886	SLC20A1	No change
1057	ENSMODG00000025438	CENPQ	No change
1058	ENSMODG00000010373	TRIB2	No change

1059	ENSMODG00000022952	IL4R	No change
1060	ENSMODG00000008244	MKX	No change
1061	ENSMODG00000011536	HVCN1	No change
1062	ENSMODG00000029453	ENSMODT00000032512	No change
1063	ENSMODG00000007327	WNT7B	No change
1064	ENSMODG00000024755	G0S2	No change
1065	ENSMODG00000011726	P2RX7	No change
1066	ENSMODG00000019719	HOMER1	No change
1068	ENSMODG00000009089	RAPGEF4	No change
1069	ENSMODG00000016895	GNG13	No change
1070	ENSMODG00000005363	GRIN2A	No change
1071	ENSMODG00000007534	C11orf16	No change
1072	ENSMODG00000011874	BRINP3	No change
1073	ENSMODG00000021245	C3orf58	No change
1074	ENSMODG00000005668	GFOD2	No change
1075	ENSMODG00000012368	CBX1	No change
1076	ENSMODG00000012880	ATP13A5	No change
1077	ENSMODG00000005300	MIDN	No change
1078	ENSMODG00000008441	PACS1	No change
1079	ENSMODG00000000378	MCOLN3	No change
1080	ENSMODG00000001972	FBXO32	No change
1082	ENSMODG00000005606	DZANK1	No change
1083	ENSMODG00000024993	ENSMODT00000038103	No change
1085	ENSMODG00000015790	PLCH1	No change
1086	ENSMODG00000004471	TRPV4	No change
1087	ENSMODG00000017602	EPB41L2	No change
1088	ENSMODG00000003544	KIFAP3	No change
1089	ENSMODG00000012616	ENSMODT00000016074	No change
1091	ENSMODG00000011035	LRRC56	No change
1092	ENSMODG00000015823	KANSL1L	No change
1093	ENSMODG00000027306	ENSMODT00000042057	No change
1094	ENSMODG00000009229	RHOBTB2	No change
1095	ENSMODG00000019350	UNG	No change
1096	ENSMODG00000014205	ANKK1	No change
1097	ENSMODG00000022740	ENSMODT00000023654	No change
1098	ENSMODG00000017545	TMEM39B	No change
1100	ENSMODG00000028693	ENSMODT00000044347	No change
1101	ENSMODG00000013533	ENSMODT00000017250	No change
1103	ENSMODG00000010674	ZSWIM2	No change
1105	ENSMODG00000010417	STEAP2	No change
1106	ENSMODG00000011609	ENSMODT00000014795	No change
1107	ENSMODG00000014611	EXPH5	No change
1108	ENSMODG00000005201	ENSMODT00000006548	No change
1109	ENSMODG00000014731	KBTBD3	No change
1111	ENSMODG00000015975	C2orf54	No change
1112	ENSMODG00000004897	AGTR2	No change
1113	ENSMODG00000020433	ZNF532	No change
1114	ENSMODG00000006883	HS3ST2	No change
1115	ENSMODG00000003676	SASS6	No change
1116	ENSMODG00000018979	DNPH1	No change
1118	ENSMODG00000006548	ENSMODT00000008289	No change
1119	ENSMODG00000000833	FOXRED2	No change

1120	ENSMODG00000024484	ENSMODT00000000175	No change
1121	ENSMODG00000025074	ENSMODT00000038383	No change
1122	ENSMODG00000017223	ENSMODT00000021858	No change
1123	ENSMODG00000009212	GRB10	No change
1124	ENSMODG00000015965	SCARA3	No change
1125	ENSMODG00000007509	CHD3	No change
1126	ENSMODG00000025439	ENSMODT00000039813	No change
1127	ENSMODG00000003527	HMGB2	No change
1128	ENSMODG00000019385	SAXO2	No change
1129	ENSMODG00000007824	CSPP1	No change
1130	ENSMODG00000023601	ENSMODT00000032231	No change
1131	ENSMODG00000029802	ENSMODT00000041852	No change
1132	ENSMODG00000011579	DNAH1	No change
1133	ENSMODG00000027882	MANSC1	No change
1134	ENSMODG00000005242	BCL2	No change
1135	ENSMODG00000009534	OVOL1	No change
1136	ENSMODG00000013439	IFT46	No change
1137	ENSMODG00000008399	NIPAL4	No change
1138	ENSMODG00000009707	IQSEC2	No change
1139	ENSMODG00000018997	WRN	No change
1140	ENSMODG00000010828	CFAP58	No change
1141	ENSMODG00000018423	TNK2	No change
1142	ENSMODG00000010184	EFCAB1	No change
1144	ENSMODG00000019392	STARD5	No change
1145	ENSMODG00000011556	TBC1D2B	No change
1146	ENSMODG00000007006	NR2C2AP	No change
1147	ENSMODG00000013606	WEE2	No change
1148	ENSMODG00000001352	ROR1	No change
1149	ENSMODG00000024630	ARL14	No change
1150	ENSMODG00000004516	BHLHE40	No change
1151	ENSMODG00000005984	SLC6A1	No change
1152	ENSMODG00000028273	ENSMODT00000042499	No change
1153	ENSMODG00000016481	CLDN9	No change
1154	ENSMODG00000021461	DLGAP1	No change
1155	ENSMODG00000018603	RBP1	No change
1156	ENSMODG00000012396	NUP214	No change
1157	ENSMODG00000015555	DNAH6	No change
1158	ENSMODG00000011962	SLC16A2	No change
1159	ENSMODG00000018839	SEMA6C	No change
1160	ENSMODG00000013060	MDGA2	No change
1161	ENSMODG00000021459	EPB41L3	No change
1162	ENSMODG00000004294	MOB3B	No change
1163	ENSMODG00000021283	ENSMODT00000027082	No change
1164	ENSMODG00000010872	ENSMODT00000013864	No change
1167	ENSMODG00000018592	CNGA4	No change
1170	ENSMODG00000015160	TMEM106A	No change
1171	ENSMODG00000004096	MCF2L	No change
1172	ENSMODG00000014786	KCNH8	No change
1173	ENSMODG00000017836	RSPH4A	No change
1176	ENSMODG00000013963	IRF1	No change
1178	ENSMODG00000010741	DDX50	No change
1179	ENSMODG00000028123	ENSMODT00000042338	No change

1180	ENSMODG00000020932	C12orf66	No change
1181	ENSMODG00000001881	SOCS3	No change
1183	ENSMODG00000017795	SLC35F1	No change
1184	ENSMODG00000011390	LZTFL1	No change
1185	ENSMODG00000014269	FAM19A2	No change
1186	ENSMODG00000002789	ENSMODT00000003464	No change
1188	ENSMODG00000008779	ZNF541	No change
1189	ENSMODG00000011699	IFT81	No change
1190	ENSMODG00000009748	ENSMODT00000012410	No change
1191	ENSMODG00000009924	NEO1	No change
1192	ENSMODG00000020651	ENSMODT00000026297	No change
1193	ENSMODG00000001318	TSGA10	No change
1194	ENSMODG00000028434	PRR29	No change
1197	ENSMODG00000005747	ENSMODT00000007265	No change
1199	ENSMODG00000020593	GNPDA2	No change
1200	ENSMODG00000019194	AREG	No change
1202	ENSMODG00000006689	TPPP2	No change
1204	ENSMODG00000000950	CHAF1A	No change
1205	ENSMODG00000016728	CCDC170	No change
1207	ENSMODG00000023655	ENSMODT00000032538	No change
1208	ENSMODG00000014261	ETV1	No change
1209	ENSMODG00000021295	CCDC39	No change
1210	ENSMODG00000001116	ZMIZ1	No change
1211	ENSMODG00000019166	ASIC2	No change
1212	ENSMODG00000016298	PTGIS	No change
1213	ENSMODG00000000771	GNG12	No change
1214	ENSMODG00000021247	ENSMODT00000027034	No change
1215	ENSMODG00000013366	P4HTM	No change
1216	ENSMODG00000028438	ENSMODT00000044232	No change
1218	ENSMODG00000001770	LPCAT4	No change
1219	ENSMODG00000008248	MRVI1	No change
1220	ENSMODG00000014346	ZNF711	No change
1221	ENSMODG00000017846	DSE	No change
1222	ENSMODG00000013192	IRX3	No change
1223	ENSMODG00000014614	ENSMODT00000018593	No change
1224	ENSMODG00000002495	C1orf87	No change
1225	ENSMODG00000004388	TCHP	No change
1226	ENSMODG00000025449	ENSMODT00000039824	No change
1228	ENSMODG00000016759	ENSMODT00000021286	No change
1229	ENSMODG00000003991	CNTN3	No change
1230	ENSMODG00000012344	ENSMODT00000034502	No change
1232	ENSMODG00000000977	EPN2	No change
1234	ENSMODG00000001519	HGNC:18790	No change
1235	ENSMODG00000002216	ARHGAP22	No change
1237	ENSMODG00000015595	ELMOD3	No change
1238	ENSMODG00000013788	PLD4	No change
1239	ENSMODG00000020627	INTS12	No change
1240	ENSMODG00000013820	WNT9A	No change
1241	ENSMODG00000003787	JPH4	No change
1242	ENSMODG00000014964	ENSMODT00000019049	No change
1243	ENSMODG00000013914	ELMO2	No change
1244	ENSMODG00000007397	P4HA3	No change

1245	ENSMODG00000000964	TBC1D4	No change
1247	ENSMODG00000002961	GCGR	No change
1248	ENSMODG00000008497	GAL3ST3	No change
1249	ENSMODG00000003495	GAS1	No change
1250	ENSMODG00000007141	ENSMODT00000009030	No change
1251	ENSMODG00000005488	PANX2	No change
1252	ENSMODG00000019740	PDE8B	No change
1253	ENSMODG00000025553	CXorf58	No change
1254	ENSMODG00000014358	YPEL1	No change
1256	ENSMODG00000002081	ADCY2	No change
1257	ENSMODG00000011228	KIF15	No change
1258	ENSMODG00000015073	P3H2	No change
1259	ENSMODG00000016810	ENSMODT00000029546	No change
1260	ENSMODG00000009947	DCLK1	No change
1261	ENSMODG00000009702	ENSMODT00000012352	No change
1262	ENSMODG00000000910	LDB2	No change
1263	ENSMODG00000001915	RAB7B	No change
1264	ENSMODG00000007957	ELK3	No change
1265	ENSMODG00000023484	ENSMODT00000031662	No change
1266	ENSMODG00000023762	ENSMODT00000033130	No change
1267	ENSMODG00000011897	SCGN	No change
1268	ENSMODG00000022738	ENSMODT00000028704	No change
1269	ENSMODG00000017849	NT5DC1	No change
1271	ENSMODG00000022791	ENSMODT00000028824	No change
1272	ENSMODG00000006952	NCF1	No change
1273	ENSMODG00000010500	ATP7B	No change
1274	ENSMODG00000016474	ENSMODT00000020935	No change
1275	ENSMODG00000002980	ATP1B1	No change
1276	ENSMODG00000002632	GNE	No change
1277	ENSMODG00000013205	ENSMODT00000016828	No change
1278	ENSMODG00000001739	GLRB	No change
1279	ENSMODG00000011864	MPP2	No change
1280	ENSMODG00000024719	PID1	No change
1281	ENSMODG00000003002	NME7	No change
1283	ENSMODG00000010814	ENSMODT00000013796	No change
1284	ENSMODG00000015482	SLC11A1	No change
1285	ENSMODG00000018585	APBB1	No change
1286	ENSMODG00000016675	CDH4	No change
1287	ENSMODG00000025493	IQCK	No change
1288	ENSMODG00000014204	TBX2	No change
1289	ENSMODG00000008683	ENSMODT00000011025	No change
1291	ENSMODG00000003500	ENSMODT00000004370	No change
1292	ENSMODG00000017552	PRKD1	No change
1293	ENSMODG00000011588	RASSF2	No change
1297	ENSMODG00000010797	CX3CR1	No change
1298	ENSMODG00000003611	ENSMODT00000004505	No change
1299	ENSMODG00000012696	C19orf12	No change
1300	ENSMODG00000017112	ENSMODT00000035493	No change
1301	ENSMODG00000009737	PDGFRB	No change
1302	ENSMODG00000015617	IFT172	No change
1303	ENSMODG00000017104	RTKN2	No change
1304	ENSMODG00000025604	C18orf54	No change

1305	ENSMODG00000029314	ENSMODT00000041907	No change
1306	ENSMODG00000029580	ENSMODT00000041885	No change
1307	ENSMODG00000028633	ENSMODT00000044137	No change
1308	ENSMODG00000005524	ARHGAP45	No change
1309	ENSMODG00000012999	UBASH3B	No change
1310	ENSMODG00000020370	SPEF2	No change
1311	ENSMODG00000007286	EYA1	No change
1312	ENSMODG00000018822	MCM3	No change
1314	ENSMODG00000011518	DCDC2	No change
1315	ENSMODG00000000558	CEP126	No change
1316	ENSMODG00000009625	RCN1	No change
1317	ENSMODG00000019201	CDKL2	No change
1318	ENSMODG00000004554	CEP128	No change
1321	ENSMODG00000001722	METTL7B	No change
1322	ENSMODG00000025613	ALKAL1	No change
1323	ENSMODG00000013516	CX3CL1	No change
1324	ENSMODG00000002850	FAM161A	No change
1326	ENSMODG00000006138	ESPN	No change
1327	ENSMODG00000024466	ENSMODT00000035830	No change
1328	ENSMODG00000019281	SLCO6A1	No change
1329	ENSMODG00000023057	ENSMODT00000029661	No change
1330	ENSMODG00000009313	F8	No change
1331	ENSMODG00000006110	KIAA1644	No change
1333	ENSMODG00000000544	ENSMODT00000000672	No change
1334	ENSMODG00000001886	MLLT1	No change
1335	ENSMODG00000018208	MAATS1	No change
1336	ENSMODG00000002197	C1orf194	No change
1338	ENSMODG00000013202	IRX5	No change
1340	ENSMODG00000014900	BNC2	No change
1342	ENSMODG00000010201	ZNF385B	No change
1343	ENSMODG00000025744	SNCB	No change
1344	ENSMODG00000004323	CNTN4	No change
1345	ENSMODG00000014092	ZBTB9	No change
1346	ENSMODG00000020105	YPEL4	No change
1347	ENSMODG00000027314	ENSMODT00000044309	No change
1348	ENSMODG00000020970	LCA5L	No change
1349	ENSMODG00000025188	ENSMODT00000038922	No change
1350	ENSMODG00000008206	IL17D	No change
1352	ENSMODG00000014201	CDKL3	No change
1353	ENSMODG00000020391	TTC23L	No change
1355	ENSMODG00000008343	CNKSR3	No change
1357	ENSMODG00000013801	ENSMODT00000033493	No change
1358	ENSMODG00000001607	SOSTDC1	No change
1359	ENSMODG00000012835	AK7	No change
1360	ENSMODG00000007743	TRIM47	No change
1361	ENSMODG00000005306	SPAG17	No change
1362	ENSMODG00000011978	PQLC1	No change
1363	ENSMODG00000007627	AHNAK	No change
1364	ENSMODG00000015141	RND2	No change
1365	ENSMODG00000007406	ENSMODT00000032157	No change
1366	ENSMODG00000001694	CTSO	No change
1367	ENSMODG00000018909	ENPP4	No change

1368	ENSMODG00000025216	ENSMODT00000038991	No change
1369	ENSMODG00000023054	ENSMODT00000029644	No change
1370	ENSMODG00000010225	PLAT	No change
1371	ENSMODG00000015231	BICDL1	No change
1373	ENSMODG00000007021	ARRB1	No change
1374	ENSMODG00000023566	ZNF672	No change
1375	ENSMODG00000002315	LRRC18	No change
1377	ENSMODG00000020672	NFKB1	No change
1380	ENSMODG00000012360	SKAP1	No change
1381	ENSMODG00000012967	ADAMTS13	No change
1382	ENSMODG00000007592	TAGAP	No change
1383	ENSMODG00000015035	SPOCK1	No change
1384	ENSMODG00000023443	ENSMODT00000031450	No change
1385	ENSMODG00000018409	ENSMODT00000023364	No change
1386	ENSMODG00000019064	C22orf23	No change
1387	ENSMODG00000011830	CPNE4	No change
1389	ENSMODG00000008812	ENSMODT00000011188	No change
1390	ENSMODG00000005026	LRCH2	No change
1391	ENSMODG00000014032	DRC7	No change
1392	ENSMODG00000002368	IL1RL1	No change
1393	ENSMODG00000006907	ENSMODT00000008728	No change
1394	ENSMODG00000002007	LYSMD2	No change
1395	ENSMODG00000018664	ENSMODT00000023695	No change
1396	ENSMODG00000001191	RGS4	No change
1397	ENSMODG00000007519	IQCA1	No change
1398	ENSMODG00000004959	SUSD4	No change
1399	ENSMODG00000012875	GRB7	No change
1400	ENSMODG00000017323	EGFL6	No change
1401	ENSMODG00000004824	LMNB2	No change
1402	ENSMODG00000009999	DPYSL3	No change
1403	ENSMODG00000018403	TPBG	No change
1406	ENSMODG00000002582	CEP152	No change
1407	ENSMODG00000003320	FAM131C	No change
1408	ENSMODG00000001129	MSH6	No change
1409	ENSMODG00000018656	ENSMODT00000023673	No change
1410	ENSMODG00000014859	CAVIN1	No change
1411	ENSMODG00000017673	ENSMODT00000022424	No change
1412	ENSMODG00000027725	ENSMODT00000044255	No change
1413	ENSMODG00000014463	CXCL14	No change
1414	ENSMODG00000012355	TRAIP	No change
1415	ENSMODG00000013030	ENSMODT00000016594	No change
1416	ENSMODG00000008852	SNUPN	No change
1417	ENSMODG00000019728	ARSB	No change
1418	ENSMODG00000006788	PDP2	No change
1419	ENSMODG00000014913	CCDC171	No change
1420	ENSMODG00000022830	ENSMODT00000002014	No change
1421	ENSMODG00000006659	ENSMODT00000033246	No change
1422	ENSMODG00000004719	ENSMODT00000038732	No change
1423	ENSMODG00000025317	CCDC24	No change
1424	ENSMODG00000012717	BICD1	No change
1425	ENSMODG00000024702	ENSMODT00000036824	No change
1426	ENSMODG00000006434	QKI	No change

1427	ENSMODG00000013824	STX1A	No change
1428	ENSMODG00000020875	ENSMODT00000026567	No change
1429	ENSMODG00000025516	PDPN	No change
1430	ENSMODG00000008260	GMDS	No change
1432	ENSMODG00000012400	FAM212A	No change
1433	ENSMODG00000001788	CEP250	No change
1434	ENSMODG00000005440	FANCD2	No change
1435	ENSMODG00000007652	PDZD9	No change
1436	ENSMODG00000015029	CDH13	No change
1437	ENSMODG00000008223	ENSMODT00000010417	No change
1438	ENSMODG00000004435	SLC44A3	No change
1439	ENSMODG00000025670	TTLL6	No change
1440	ENSMODG00000005106	ENSMODT00000038140	No change
1441	ENSMODG00000017956	AK9	No change
1442	ENSMODG00000016345	RIPOR3	No change
1443	ENSMODG00000007346	NALCN	No change
1444	ENSMODG00000015621	RGMB	No change
1446	ENSMODG00000005145	TTF2	No change
1447	ENSMODG00000004135	CHEK1	No change
1448	ENSMODG00000002995	SORCS2	No change
1450	ENSMODG00000020825	ENSMODT00000026511	No change
1451	ENSMODG00000019867	ENSMODT00000025230	No change
1452	ENSMODG00000006413	MXRA8	No change
1453	ENSMODG00000012598	MLLT6	No change
1455	ENSMODG00000014263	ALKAL2	No change
1456	ENSMODG00000014692	ENSMODT00000018696	No change
1457	ENSMODG00000011996	TGFBR3	No change
1458	ENSMODG00000019165	JAK3	No change
1459	ENSMODG00000012853	KIF14	No change
1460	ENSMODG00000025702	ENSMODT00000040140	No change
1461	ENSMODG00000000564	PLAU	No change
1462	ENSMODG00000003433	C1orf112	No change
1463	ENSMODG00000002027	ATAD2	No change
1464	ENSMODG00000009932	CSGALNACT1	No change
1466	ENSMODG00000002435	TLL1	No change
1467	ENSMODG00000011485	PHF7	No change
1468	ENSMODG00000027504	ENSMODT00000043953	No change
1469	ENSMODG00000013193	SLCO3A1	No change
1470	ENSMODG00000014713	ENSMODT00000018727	No change
1471	ENSMODG00000016141	ENSMODT00000020516	No change
1472	ENSMODG00000000818	ENSMODT00000000993	No change
1473	ENSMODG00000019483	ENSMODT00000024735	No change
1474	ENSMODG00000015869	DNAJC27	No change
1475	ENSMODG00000006620	ZNF219	No change
1476	ENSMODG00000011018	DSG3	No change
1477	ENSMODG00000016868	ENSMODT00000021421	No change
1478	ENSMODG00000012687	CACNB1	No change
1480	ENSMODG00000003131	PGM5	No change
1481	ENSMODG00000001866	STXBP6	No change
1482	ENSMODG00000007964	MYBL1	No change
1483	ENSMODG00000003675	ENSMODT00000004602	No change
1484	ENSMODG00000013005	C3orf62	No change

1485	ENSMODG00000001871	BIRC5	No change
1487	ENSMODG00000000575	ENSMODT00000000705	No change
1488	ENSMODG00000008340	RNF157	No change
1489	ENSMODG00000013160	TRIM9	No change
1490	ENSMODG00000007016	ACSM3	No change
1491	ENSMODG00000005113	APC2	No change
1492	ENSMODG00000012238	ANKRD44	No change
1493	ENSMODG00000024870	GPBAR1	No change
1494	ENSMODG00000004138	FMNL2	No change
1495	ENSMODG00000017934	METTL24	No change
1496	ENSMODG00000024555	GPR85	No change
1497	ENSMODG00000016388	CCDC71L	No change
1498	ENSMODG00000023304	ENSMODT00000030952	No change
1499	ENSMODG00000003291	CENPF	No change
1501	ENSMODG00000024672	ENSMODT00000036669	No change
1502	ENSMODG00000027535	ENSMODT00000044130	No change
1503	ENSMODG00000012655	ENSMODT00000032647	No change
1504	ENSMODG00000005415	SAMD15	No change
1505	ENSMODG00000008942	PXDC1	No change
1506	ENSMODG00000001679	ZNF629	No change
1507	ENSMODG00000014667	MBOAT2	No change
1508	ENSMODG00000000164	EXD1	No change
1510	ENSMODG00000020809	ANXA3	No change
1511	ENSMODG00000013448	TMEM25	No change
1512	ENSMODG00000008136	RFX7	No change
1513	ENSMODG00000025468	ENSMODT00000039845	No change
1514	ENSMODG00000017674	KIAA0408	No change
1515	ENSMODG00000009761	AFAP1L2	No change
1516	ENSMODG00000024450	CHST1	No change
1517	ENSMODG00000029569	ENSMODT00000042569	No change
1518	ENSMODG00000010580	PHACTR1	No change
1519	ENSMODG00000007343	ENSMODT00000009286	No change
1520	ENSMODG00000001262	RASGEF1A	No change
1521	ENSMODG00000003830	PCSK9	No change
1522	ENSMODG00000007332	C1orf21	No change
1523	ENSMODG00000001302	RBM46	No change
1525	ENSMODG00000024731	TLNRD1	No change
1526	ENSMODG00000015229	ARF5	No change
1527	ENSMODG00000023422	ENSMODT00000031359	No change
1528	ENSMODG00000003060	TTC8	No change
1530	ENSMODG00000018190	B4GALT4	No change
1531	ENSMODG00000020429	RAX	No change
1532	ENSMODG00000028083	ENSMODT00000041801	No change
1533	ENSMODG00000001208	RGS5	No change
1534	ENSMODG00000006291	ENSMODT00000007961	No change
1535	ENSMODG00000017144	SCML2	No change
1536	ENSMODG00000011406	SLC40A1	No change
1538	ENSMODG00000006308	SH3PXD2B	No change
1539	ENSMODG00000023861	ENSMODT00000015936	No change
1540	ENSMODG00000008610	NUP210	No change
1541	ENSMODG00000004572	IRF8	No change
1544	ENSMODG00000008876	RGS10	No change

1546	ENSMODG00000025297	ENSMODT00000039192	No change
1547	ENSMODG00000013582	ZBTB47	No change
1548	ENSMODG00000010092	CDK20	No change
1549	ENSMODG00000002168	CCDC40	No change
1550	ENSMODG00000013677	ZNF197	No change
1551	ENSMODG00000019200	ATAD5	No change
1552	ENSMODG00000024917	ENSMODT00000037805	No change
1553	ENSMODG00000012145	ADGRL1	No change
1554	ENSMODG00000003469	DOK7	No change
1555	ENSMODG00000008339	SLC29A2	No change
1556	ENSMODG00000009345	ADAMTS2	No change
1557	ENSMODG00000011948	C2orf50	No change
1558	ENSMODG00000013521	DPYSL4	No change
1559	ENSMODG00000016922	CADM2	No change
1560	ENSMODG00000006256	SCUBE1	No change
1561	ENSMODG00000011594	CDKN3	No change
1562	ENSMODG00000006435	FSTL3	No change
1563	ENSMODG00000015677	LXN	No change
1564	ENSMODG00000018406	RAD52	No change
1565	ENSMODG00000001484	DOCK7	No change
1567	ENSMODG00000004660	GIGYF1	No change
1568	ENSMODG00000015337	TAOK3	No change
1569	ENSMODG00000015887	ADCY3	No change
1570	ENSMODG00000009486	ENO4	No change
1571	ENSMODG00000006132	TMLHE	No change
1572	ENSMODG00000029483	ENSMODT00000043193	No change
1573	ENSMODG00000010384	CERKL	No change
1574	ENSMODG00000008232	ARMC4	No change
1575	ENSMODG00000012337	IL17RD	No change
1576	ENSMODG00000014508	SYDE1	No change
1577	ENSMODG00000020367	IL7R	No change
1578	ENSMODG00000008022	ENSMODT00000010178	No change
1580	ENSMODG00000025678	HLA-DOB	No change
1581	ENSMODG00000016421	DKK1	No change
1582	ENSMODG00000018145	GNB3	No change
1583	ENSMODG00000016771	PLEKHG1	No change
1584	ENSMODG00000006817	PLD5	No change
1585	ENSMODG00000011492	ENSMODT00000014635	No change
1586	ENSMODG00000014214	JADE2	No change
1588	ENSMODG00000028756	CD74	No change
1590	ENSMODG00000020853	ENSMODT00000026543	No change
1591	ENSMODG00000010447	ENSMODT00000013334	No change
1592	ENSMODG00000012706	ENSMODT00000016189	No change
1593	ENSMODG00000025428	ENSMODT00000039744	No change
1595	ENSMODG00000000126	ITPKA	No change
1596	ENSMODG00000000712	MMP1	No change
1597	ENSMODG00000000996	PPFIA4	No change
1598	ENSMODG00000000320	ENSMODT00000000390	No change
1600	ENSMODG00000012263	PLK4	No change
1601	ENSMODG00000017396	ENSMODT00000022090	No change
1602	ENSMODG00000017660	ENSMODT00000022416	No change
1603	ENSMODG00000023349	GJB3	No change

1604	ENSMODG00000014894	HSH2D	No change
1607	ENSMODG00000009147	SKIDA1	No change
1608	ENSMODG00000020101	SLC43A1	No change
1609	ENSMODG00000007414	STK33	No change
1610	ENSMODG00000006443	GINS1	No change
1611	ENSMODG00000002357	RNF213	No change
1613	ENSMODG00000027776	ENSMODT00000042640	No change
1614	ENSMODG00000008437	ENSMODT00000010698	No change
1615	ENSMODG00000014583	RSAD2	No change
1616	ENSMODG00000009376	ANO3	No change
1617	ENSMODG00000021223	DNER	No change
1619	ENSMODG00000002305	ENSMODT00000002867	No change
1620	ENSMODG00000017792	CEP85L	No change
1621	ENSMODG00000028429	ENSMODT00000044029	No change
1622	ENSMODG00000000772	ARHGAP10	No change
1623	ENSMODG00000028185	PRIMA1	No change
1624	ENSMODG00000007681	VWA3A	No change
1625	ENSMODG00000014378	MYT1L	No change
1626	ENSMODG00000018713	FMO5	No change
1627	ENSMODG00000011623	FAM69A	No change
1628	ENSMODG00000010895	EFNB1	No change
1629	ENSMODG00000005865	FRMD1	No change
1630	ENSMODG00000013427	SNX24	No change
1631	ENSMODG00000017132	ENSMODT00000030902	No change
1632	ENSMODG00000011690	ARHGAP25	No change
1633	ENSMODG00000007810	NUMBL	No change
1634	ENSMODG00000008186	ENSMODT00000010367	No change
1637	ENSMODG00000005683	ENSMODT00000038525	No change
1638	ENSMODG00000010195	ADCY1	No change
1639	ENSMODG00000009743	STRA6	No change
1640	ENSMODG00000023654	ENSMODT00000005857	No change
1642	ENSMODG00000013182	ST8SIA2	No change
1643	ENSMODG00000017591	NOTCH1	No change
1644	ENSMODG00000024029	ENSMODT00000034232	No change
1645	ENSMODG00000005384	JAM3	No change
1646	ENSMODG00000019838	C11orf74	No change
1648	ENSMODG00000001260	SGK2	No change
1649	ENSMODG00000019092	DCK	No change
1650	ENSMODG00000010436	ENSMODT00000013319	No change
1652	ENSMODG00000025706	CD34	No change
1654	ENSMODG00000018552	SLC2A13	No change
1655	ENSMODG00000010338	ADAM22	No change
1656	ENSMODG00000023648	ENSMODT00000032467	No change
1657	ENSMODG00000003610	MYO1F	No change
1658	ENSMODG00000000556	ADGRB1	No change
1659	ENSMODG00000014978	ENSMODT00000019068	No change
1660	ENSMODG00000008416	CYP2S1	No change
1661	ENSMODG00000008392	PCNT	No change
1663	ENSMODG00000013614	SEMA6A	No change
1664	ENSMODG00000012786	PRUNE2	No change
1665	ENSMODG00000018153	LRRC23	No change
1667	ENSMODG00000000065	DPY19L1	No change

1668	ENSMODG00000003703	RIF1	No change
1670	ENSMODG00000027484	ENSMODT00000042947	No change
1671	ENSMODG00000020914	SKI	No change
1673	ENSMODG00000019822	CD44	No change
1674	ENSMODG00000008196	IFT88	No change
1676	ENSMODG00000015931	KLHL30	No change
1677	ENSMODG00000011207	TIGD5	No change
1678	ENSMODG00000023532	ENSMODT00000031859	No change
1679	ENSMODG00000019495	ENSMODT00000024749	No change
1680	ENSMODG00000009441	ENSMODT00000012022	No change
1681	ENSMODG00000007379	PCYT1B	No change
1682	ENSMODG00000016657	ARHGDIG	No change
1683	ENSMODG00000015873	ENSMODT00000020160	No change
1684	ENSMODG00000008530	PIK3R5	No change
1685	ENSMODG00000014045	DRC3	No change
1686	ENSMODG00000008454	ENSMODT00000010848	No change
1687	ENSMODG00000005370	ENSMODT00000032375	No change
1688	ENSMODG00000012182	LRRC43	No change
1689	ENSMODG00000028505	ENSMODT00000042243	No change
1690	ENSMODG00000020659	LNX1	No change
1693	ENSMODG00000027925	ENSMODT00000006640	No change
1694	ENSMODG00000000667	IL2RB	No change
1696	ENSMODG00000006439	ABHD12	No change
1697	ENSMODG00000006433	NPHP4	No change
1698	ENSMODG00000005868	DNM1	No change
1699	ENSMODG00000019294	ENSMODT00000024508	No change
1700	ENSMODG00000025171	ENSMODT00000038864	No change
1701	ENSMODG00000008210	ENSMODT00000034115	No change
1702	ENSMODG00000012473	LRRC46	No change
1703	ENSMODG00000018906	TDRKH	No change
1704	ENSMODG00000002254	SLC26A11	No change
1705	ENSMODG00000016655	RGS11	No change
1707	ENSMODG00000019263	SYK	No change
1708	ENSMODG00000022672	ENSMODT00000028544	No change
1709	ENSMODG00000020258	SSBP2	No change
1710	ENSMODG00000011223	ENSMODT00000014299	No change
1711	ENSMODG00000014778	VEZF1	No change
1712	ENSMODG00000008587	DHRS7	No change
1713	ENSMODG00000014716	FKBP10	No change
1714	ENSMODG00000018147	CFAP44	No change
1715	ENSMODG00000017091	ENSMODT00000021703	No change
1717	ENSMODG00000017886	FAM229B	No change
1718	ENSMODG00000006780	HECTD2	No change
1719	ENSMODG00000025177	ENSMODT00000016070	No change
1721	ENSMODG00000009987	TMEM72	No change
1722	ENSMODG00000013102	LRFN2	No change
1725	ENSMODG00000003688	FRMD4B	No change
1727	ENSMODG00000002014	TIMP2	No change
1728	ENSMODG00000022793	ENSMODT00000015706	No change
1729	ENSMODG00000013866	NCOA5	No change
1730	ENSMODG00000023765	OAS2	No change
1731	ENSMODG00000015914	TRIM35	No change

1732	ENSMODG00000002637	BAHCC1	No change
1733	ENSMODG00000021071	C21orf62	No change
1734	ENSMODG00000029236	ENSMODT00000042351	No change
1735	ENSMODG00000015116	FGF12	No change
1736	ENSMODG00000004416	WDR31	No change
1737	ENSMODG00000021398	RTTN	No change
1738	ENSMODG00000020872	MINDY4	No change
1739	ENSMODG00000006295	ENSMODT00000007976	No change
1740	ENSMODG00000012903	IP6K1	No change
1741	ENSMODG00000010227	ENSMODT00000034428	No change
1742	ENSMODG00000029447	FAM216A	No change
1744	ENSMODG00000007617	RSPH3	No change
1745	ENSMODG00000007698	ALOXE3	No change
1746	ENSMODG00000011405	DNAAF2	No change
1747	ENSMODG00000001245	IFT52	No change
1748	ENSMODG00000012591	ENSMODT00000016046	No change
1749	ENSMODG00000021259	HPS3	No change
1750	ENSMODG00000012063	CACNA2D2	No change
1751	ENSMODG00000015499	ENSMODT00000019693	No change
1752	ENSMODG00000015369	PRKAG3	No change
1753	ENSMODG00000013344	ENSMODT00000016996	No change
1754	ENSMODG00000014437	PPFIBP1	No change
1755	ENSMODG00000017362	ENSMODT00000022045	No change
1756	ENSMODG00000028546	MORC1	No change
1757	ENSMODG00000001412	ENSMODT00000030061	No change
1760	ENSMODG00000007723	ENSMODT00000038205	No change
1761	ENSMODG00000014663	MTA1	No change
1762	ENSMODG00000013382	S1PR3	No change
1764	ENSMODG00000008508	NCAPH	No change
1766	ENSMODG00000001497	TGFB1I1	No change
1767	ENSMODG00000006555	ENSMODT00000008298	No change
1768	ENSMODG00000011617	ENSMODT00000029157	No change
1769	ENSMODG00000016301	B4GALT5	No change
1770	ENSMODG00000027670	ENSMODT00000043258	No change
1771	ENSMODG00000005153	RNF44	No change
1772	ENSMODG00000016438	TTLL10	No change
1773	ENSMODG00000010309	ENSMODT00000013161	No change
1774	ENSMODG00000001274	MEIS2	No change
1775	ENSMODG00000016265	SLITRK3	No change
1776	ENSMODG00000007190	ENSMODT00000009090	No change
1777	ENSMODG00000000278	PANX1	No change
1780	ENSMODG00000001357	CAPS	No change
1781	ENSMODG00000008706	PRKCH	No change
1782	ENSMODG00000020360	TMEM138	No change
1784	ENSMODG00000005798	ENSMODT00000007322	No change
1785	ENSMODG00000000181	TMEM9	No change
1786	ENSMODG00000009609	SMAD3	No change
1787	ENSMODG00000022842	ENSMODT00000029052	No change
1788	ENSMODG00000017644	SLC28A2	No change
1789	ENSMODG00000018264	ENSMODT00000023179	No change
1790	ENSMODG00000009366	ENSMODT00000011922	No change
1791	ENSMODG00000002646	CHRND	No change

1792	ENSMODG00000016330	SLC26A3	No change
1794	ENSMODG00000010633	ENSMODT00000013563	No change
1795	ENSMODG00000003042	ATF3	No change
1796	ENSMODG00000002544	PARPBP	No change
1797	ENSMODG00000004038	ATOH8	No change
1799	ENSMODG00000024047	GPR65	No change
1800	ENSMODG00000004259	ZFPM1	No change
1801	ENSMODG00000015011	ENSMODT00000019108	No change
1802	ENSMODG00000019111	HEATR6	No change
1803	ENSMODG00000008659	WNT7A	No change
1804	ENSMODG00000014710	IRF5	No change
1805	ENSMODG00000012656	ENSMODT00000016127	No change
1806	ENSMODG00000007452	CFAP100	No change
1807	ENSMODG00000008090	KIAA0586	No change
1808	ENSMODG00000023052	ENSMODT00000014932	No change
1809	ENSMODG00000019524	MAP3K1	No change
1812	ENSMODG00000002782	NIP7	No change
1813	ENSMODG00000011969	SGCA	No change
1814	ENSMODG00000020898	XYLT2	No change
1815	ENSMODG00000006293	ENSMODT00000007964	No change
1816	ENSMODG00000017883	TEKT2	No change
1817	ENSMODG00000019221	WSB1	No change
1818	ENSMODG00000012338	HOXB5	No change
1819	ENSMODG00000017719	FHL3	No change
1820	ENSMODG00000029035	ENSMODT00000043694	No change
1822	ENSMODG00000018312	CCND2	No change
1823	ENSMODG00000002287	FILIP1L	No change
1824	ENSMODG00000011110	ENSMODT00000034187	No change
1825	ENSMODG00000014687	ENSMODT00000041824	No change
1826	ENSMODG00000013809	ENSMODT00000017581	No change
1828	ENSMODG00000005703	ENSMODT00000007208	No change
1829	ENSMODG00000007489	TOGARAM1	No change
1830	ENSMODG00000002087	MAPKAPK2	No change
1831	ENSMODG00000018560	ENSMODT00000023565	No change
1832	ENSMODG00000018200	ADPRH	No change
1833	ENSMODG00000013210	IRX6	No change
1834	ENSMODG00000000233	ENSMODT00000000287	No change
1836	ENSMODG00000010406	ENSMODT00000013281	No change
1838	ENSMODG00000018899	FGF2	No change
1839	ENSMODG00000005994	MIXL1	No change
1840	ENSMODG00000000354	WDR63	No change
1842	ENSMODG00000018394	CCDC77	No change
1843	ENSMODG00000014329	ENSMODT00000019336	No change
1844	ENSMODG00000001514	USP1	No change
1845	ENSMODG00000025573	MORN1	No change
1846	ENSMODG00000012040	ENSMODT00000028809	No change
1847	ENSMODG00000024203	ENSMODT00000035148	No change
1848	ENSMODG00000016554	ENSMODT00000021030	No change
1849	ENSMODG00000011529	FAM171A2	No change
1850	ENSMODG00000003164	SELP	No change
1851	ENSMODG00000016212	GOLIM4	No change
1852	ENSMODG00000007565	ENSMODT00000009573	No change

1853	ENSMODG00000013152	TOP2A	No change
1856	ENSMODG00000012543	TRIM14	No change
1857	ENSMODG00000015761	DPYSL5	No change
1858	ENSMODG00000019921	FNBP4	No change
1859	ENSMODG00000012850	CNTN1	No change
49	ENSMODG00000015625	ENSMODT00000019852	Not expressed
90	ENSMODG00000023951	ENSMODT00000033937	Not expressed
223	ENSMODG00000024794	NWD2	Not expressed
295	ENSMODG00000021084	ENSMODT00000026831	Not expressed
309	ENSMODG00000019900	ENSMODT00000025269	Not expressed
443	ENSMODG00000001106	KCNK12	Not expressed
448	ENSMODG00000023085	ENSMODT00000029830	Not expressed
570	ENSMODG00000016913	ENSMODT00000021474	Not expressed
603	ENSMODG00000014394	PLPPR1	Not expressed
736	ENSMODG00000009329	SLC2A5	Not expressed
776	ENSMODG00000019390	TMC3	Not expressed
829	ENSMODG00000013898	ENSMODT00000017707	Not expressed
852	ENSMODG00000007979	ENSMODT00000010129	Not expressed
882	ENSMODG00000002636	ENSMODT00000003266	Not expressed
911	ENSMODG00000005972	KCTD19	Not expressed
912	ENSMODG00000020531	ENSMODT00000026144	Not expressed
943	ENSMODG00000012885	ENSMODT00000016415	Not expressed
966	ENSMODG00000012060	ENSMODT00000015390	Not expressed
975	ENSMODG00000021162	CRHR2	Not expressed
982	ENSMODG00000005707	WDR27	Not expressed
1008	ENSMODG00000007923	ENSMODT00000038560	Not expressed
1046	ENSMODG00000019508	NRSN2	Not expressed
1110	ENSMODG00000019607	ENSMODT00000024898	Not expressed
1187	ENSMODG00000007199	IL1RAPL1	Not expressed
1217	ENSMODG00000003565	SDSL	Not expressed
1231	ENSMODG00000028314	SPATS1	Not expressed
1236	ENSMODG00000021159	ENSMODT00000026921	Not expressed
1270	ENSMODG00000009416	ENSMODT00000011985	Not expressed
1313	ENSMODG00000005879	LRIT3	Not expressed
1320	ENSMODG00000017943	ENSMODT00000022767	Not expressed
1356	ENSMODG00000006938	ENSMODT00000008769	Not expressed
1372	ENSMODG00000024493	ENSMODT00000035950	Not expressed
1454	ENSMODG00000005729	ALPK1	Not expressed
1479	ENSMODG00000002674	ENSMODT00000003312	Not expressed
1542	ENSMODG00000011665	ENSMODT00000014882	Not expressed
1606	ENSMODG00000009252	GM2A	Not expressed
1653	ENSMODG00000029375	ENSMODT00000042021	Not expressed
1666	ENSMODG00000009237	NELL1	Not expressed
1672	ENSMODG00000020992	NCAM2	Not expressed
1675	ENSMODG00000003100	ENSMODT00000001913	Not expressed
1716	ENSMODG00000013543	SCN2B	Not expressed
1841	ENSMODG00000024367	ENSMODT00000035523	Not expressed
1854	ENSMODG00000006599	ENSMODT00000008351	Not expressed

Supplementary Table 5. Cluster analysis of shared upregulated uterine genes in early pregnancy compared to non-reproductive state between *Monodelphis domestica* and *Sminthopsis crassicaudata*.								
Annotation Cluster 1	Lysosome function	Enrichment Score	2.32					
Category	Term	Count	%	PValue	Genes	Fold Enrichment	Benjamini	FDR
GOTERM_CC_ALL	GO:0005765~lysosomal membrane	9	4.86	1.94E-04	ENSMODG00000020677, ENSMODG00000014091, ENSMODG00000004631, ENSMODG00000009825, ENSMODG00000019574, ENSMODG00000005215, ENSMODG00000009431, ENSMODG00000016892, ENSMODG00000014803	5.62	4.41E-03	0.26
GOTERM_CC_ALL	GO:0098852~lytic vacuole membrane	9	4.86	1.94E-04	ENSMODG00000020677, ENSMODG00000014091, ENSMODG00000004631, ENSMODG00000009825, ENSMODG00000019574, ENSMODG00000005215, ENSMODG00000009431, ENSMODG00000016892, ENSMODG00000014803	5.62	4.41E-03	0.26
GOTERM_CC_ALL	GO:0005774~vacuolar membrane	9	4.86	1.12E-02	ENSMODG00000020677, ENSMODG00000014091, ENSMODG00000004631, ENSMODG00000009825, ENSMODG00000019574, ENSMODG00000005215, ENSMODG00000009431, ENSMODG00000016892, ENSMODG00000014803	2.95	1.30E-01	13.78
GOTERM_CC_ALL	GO:0044437~vacuolar part	9	4.86	1.44E-02	ENSMODG00000020677, ENSMODG00000014091, ENSMODG00000004631, ENSMODG00000009825, ENSMODG00000019574, ENSMODG00000005215, ENSMODG00000009431, ENSMODG00000016892, ENSMODG00000014803	2.82	1.36E-01	17.38
GOTERM_CC_ALL	GO:0098805~whole membrane	9	4.86	4.04E-01	ENSMODG00000020677, ENSMODG00000014091, ENSMODG00000004631, ENSMODG00000009825, ENSMODG00000019574, ENSMODG00000005215, ENSMODG00000009431, ENSMODG00000016892, ENSMODG00000014803	1.27	9.37E-01	99.89
Annotation Cluster 2	Metabolism	Enrichment Score	2.06					
Category	Term	Count	%	PValue	Genes	Fold Enrichment	Benjamini	FDR
GOTERM_BP_ALL	GO:0019348~dolichol metabolic process	3	1.62	2.01E-03	ENSMODG00000017799, ENSMODG00000016356, ENSMODG00000013285	42.09	2.94E-01	3.38
GOTERM_BP_ALL	GO:0016093~polyprenol metabolic process	3	1.62	2.79E-03	ENSMODG00000017799, ENSMODG00000016356, ENSMODG00000013285	36.08	2.83E-01	4.66
GOTERM_BP_ALL	GO:0006720~isoprenoid metabolic process	3	1.62	1.17E-01	ENSMODG00000017799, ENSMODG00000016356, ENSMODG00000013285	5.05	9.58E-01	88.17
Annotation Cluster 3	Proton transport	Enrichment Score	1.94					
Category	Term	Count	%	PValue	Genes	Fold Enrichment	Benjamini	FDR
GOTERM_CC_ALL	GO:0033176~proton-transporting V-type ATPase complex	4	2.16	2.05E-03	ENSMODG00000009825, ENSMODG00000019574, ENSMODG00000009431, ENSMODG00000014803	15.44	3.45E-02	2.67
GOTERM_CC_ALL	GO:0016469~proton-transporting two-sector ATPase complex	4	2.16	9.12E-03	ENSMODG00000009825, ENSMODG00000019574, ENSMODG00000009431, ENSMODG00000014803	9.18	1.12E-01	11.37
GOTERM_MF_ALL	GO:0016820~hydrolase activity, acting on acid anhydrides, catalyzing transmembrane movement of substances	4	2.16	8.02E-02	ENSMODG00000009825, ENSMODG00000019574, ENSMODG00000009431, ENSMODG00000014803	3.93	8.12E-01	69.35
Annotation Cluster 4	Exosome function	Enrichment Score	1.87					
Category	Term	Count	%	PValue	Genes	Fold Enrichment	Benjamini	FDR
GOTERM_CC_ALL	GO:0070062~extracellular exosome	33	17.84	1.27E-02	ENSMODG00000016955, ENSMODG00000005064, ENSMODG00000007832, ENSMODG00000004392, ENSMODG00000013318, ENSMODG00000016406, ENSMODG00000017184, ENSMODG00000019574, ENSMODG00000002182, ENSMODG00000006552, ENSMODG00000029803, ENSMODG00000014811, ENSMODG00000002333, ENSMODG00000007786, ENSMODG00000020949, ENSMODG00000019764, ENSMODG00000003558, ENSMODG00000012883, ENSMODG00000016512, ENSMODG00000009152, ENSMODG00000016353, ENSMODG00000012906, ENSMODG00000018058, ENSMODG00000007110, ENSMODG00000000393, ENSMODG00000010863, ENSMODG00000003073, ENSMODG00000014803, ENSMODG00000017363, ENSMODG00000020424, ENSMODG00000009825, ENSMODG00000020405, ENSMODG00000009431	1.52	1.40E-01	15.45

GOTERM_CC_ALL	GO:1903561~extracellular vesicle	33	17.84	1.38E-02	ENSMODG00000016955, ENSMODG00000005064, ENSMODG00000007832, ENSMODG00000004392, ENSMODG00000013318, ENSMODG00000016406, ENSMODG00000017184, ENSMODG00000019574, ENSMODG00000002182, ENSMODG00000006552, ENSMODG00000029803, ENSMODG00000014811, ENSMODG00000002333, ENSMODG00000007786, ENSMODG00000020949, ENSMODG00000019764, ENSMODG00000003558, ENSMODG00000012883, ENSMODG00000016512, ENSMODG00000009152, ENSMODG00000016353, ENSMODG00000012906, ENSMODG00000018058, ENSMODG00000007110, ENSMODG00000000393, ENSMODG00000010863, ENSMODG00000003073, ENSMODG00000014803, ENSMODG00000017363, ENSMODG00000020424, ENSMODG00000009825, ENSMODG00000020405, ENSMODG00000009431	1.51	1.41E-01	16.78
GOTERM_CC_ALL	GO:0043230~extracellular organelle	33	17.84	1.40E-02	ENSMODG00000016955, ENSMODG00000005064, ENSMODG00000007832, ENSMODG00000004392, ENSMODG00000013318, ENSMODG00000016406, ENSMODG00000017184, ENSMODG00000019574, ENSMODG00000002182, ENSMODG00000006552, ENSMODG00000029803, ENSMODG00000014811, ENSMODG00000002333, ENSMODG00000007786, ENSMODG00000020949, ENSMODG00000019764, ENSMODG00000003558, ENSMODG00000012883, ENSMODG00000016512, ENSMODG00000009152, ENSMODG00000016353, ENSMODG00000012906, ENSMODG00000018058, ENSMODG00000007110, ENSMODG00000000393, ENSMODG00000010863, ENSMODG00000003073, ENSMODG00000014803, ENSMODG00000017363, ENSMODG00000020424, ENSMODG00000009825, ENSMODG00000020405, ENSMODG00000009431	1.51	1.37E-01	16.89
Annotation Cluster 5	Metabolism	Enrichment Score	1.61					
Category	Term	Count	%	PValue	Genes	Fold Enrichment	Benjamini	FDR
GOTERM_BP_ALL	GO:0019752~carboxylic acid metabolic process	12	6.49	1.95E-02	ENSMODG00000009610, ENSMODG00000007831, ENSMODG00000005464, ENSMODG00000029505, ENSMODG00000003558, ENSMODG00000025295, ENSMODG00000020405, ENSMODG00000016512, ENSMODG00000002787, ENSMODG00000012957, ENSMODG00000023271, ENSMODG00000003073	2.21	6.17E-01	28.58
GOTERM_BP_ALL	GO:0043436~oxoacid metabolic process	12	6.49	1.98E-02	ENSMODG00000009610, ENSMODG00000007831, ENSMODG00000005464, ENSMODG00000029505, ENSMODG00000003558, ENSMODG00000025295, ENSMODG00000020405, ENSMODG00000016512, ENSMODG00000002787, ENSMODG00000012957, ENSMODG00000023271, ENSMODG00000003073	2.20	6.13E-01	28.93
GOTERM_BP_ALL	GO:0006082~organic acid metabolic process	12	6.49	3.81E-02	ENSMODG00000009610, ENSMODG00000007831, ENSMODG00000005464, ENSMODG00000029505, ENSMODG00000003558, ENSMODG00000025295, ENSMODG00000020405, ENSMODG00000016512, ENSMODG00000002787, ENSMODG00000012957, ENSMODG00000023271, ENSMODG00000003073	1.98	7.65E-01	48.54
Annotation Cluster 6	Transport	Enrichment Score	1.60					
Category	Term	Count	%	PValue	Genes	Fold Enrichment	Benjamini	FDR
GOTERM_BP_ALL	GO:1903513~endoplasmic reticulum to cytosol transport	3	1.62	2.07E-02	ENSMODG00000010088, ENSMODG00000012664, ENSMODG00000004556	13.29	6.21E-01	30.07
GOTERM_BP_ALL	GO:0030970~retrograde protein transport, ER to cytosol	3	1.62	2.07E-02	ENSMODG00000010088, ENSMODG00000012664, ENSMODG00000004556	13.29	6.21E-01	30.07
GOTERM_BP_ALL	GO:0032527~protein exit from endoplasmic reticulum	3	1.62	3.73E-02	ENSMODG00000010088, ENSMODG00000012664, ENSMODG00000004556	9.71	7.71E-01	47.79
Annotation Cluster 7	Glycosylation	Enrichment Score	1.53					
Category	Term	Count	%	PValue	Genes	Fold Enrichment	Benjamini	FDR
GOTERM_BP_ALL	GO:0006486~protein glycosylation	7	3.78	1.49E-02	ENSMODG00000010088, ENSMODG00000017799, ENSMODG00000020677, ENSMODG00000012667, ENSMODG00000016356, ENSMODG00000013285, ENSMODG00000004556	3.49	5.48E-01	22.66
GOTERM_BP_ALL	GO:0043413~macromolecule glycosylation	7	3.78	1.49E-02	ENSMODG00000010088, ENSMODG00000017799, ENSMODG00000020677, ENSMODG00000012667, ENSMODG00000016356, ENSMODG00000013285, ENSMODG00000004556	3.49	5.48E-01	22.66
GOTERM_BP_ALL	GO:0009101~glycoprotein biosynthetic process	7	3.78	4.51E-02	ENSMODG00000010088, ENSMODG00000017799, ENSMODG00000020677, ENSMODG00000012667, ENSMODG00000016356, ENSMODG00000013285, ENSMODG00000004556	2.69	7.91E-01	54.55

GOTERM_BP_ALL	GO:0009100~glycoprotein metabolic process	7	3.78	7.32E-02	ENSMODG00000010088, ENSMODG00000017799, ENSMODG00000020677, ENSMODG00000012667, ENSMODG00000016356, ENSMODG00000013285, ENSMODG00000004556	2.38	9.06E-01	72.72
Annotation Cluster 8	Respone to unfolded protein	Enrichment Score	1.51					
Category	Term	Count	%	PValue	Genes	Fold Enrichment	Benjamini	FDR
GOTERM_BP_ALL	GO:0030968~endoplasmic reticulum unfolded protein response	4	2.16	2.21E-02	ENSMODG00000010088, ENSMODG00000019764, ENSMODG00000008145, ENSMODG00000004556	6.60	6.37E-01	31.79
GOTERM_BP_ALL	GO:0034620~cellular response to unfolded protein	4	2.16	2.33E-02	ENSMODG00000010088, ENSMODG00000019764, ENSMODG00000008145, ENSMODG00000004556	6.48	6.39E-01	33.16
GOTERM_BP_ALL	GO:0006986~response to unfolded protein	4	2.16	2.70E-02	ENSMODG00000010088, ENSMODG00000019764, ENSMODG00000008145, ENSMODG00000004556	6.12	6.85E-01	37.32
GOTERM_BP_ALL	GO:0035967~cellular response to topologically incorrect protein	4	2.16	4.13E-02	ENSMODG00000010088, ENSMODG00000019764, ENSMODG00000008145, ENSMODG00000004556	5.18	7.73E-01	51.34
GOTERM_BP_ALL	GO:0035966~response to topologically incorrect protein	4	2.16	4.96E-02	ENSMODG00000010088, ENSMODG00000019764, ENSMODG00000008145, ENSMODG00000004556	4.81	8.17E-01	58.08
Annotation Cluster 9	Proton transport	Enrichment Score	1.40					
Category	Term	Count	%	PValue	Genes	Fold Enrichment	Benjamini	FDR
GOTERM_CC_ALL	GO:0033180~proton-transporting V-type ATPase, V1 domain	3	1.62	4.64E-03	ENSMODG00000009825, ENSMODG00000009431, ENSMODG00000014803	28.31	6.81E-02	5.94
GOTERM_CC_ALL	GO:0033178~proton-transporting two-sector ATPase complex, catalytic domain	3	1.62	1.29E-02	ENSMODG00000009825, ENSMODG00000009431, ENSMODG00000014803	16.98	1.38E-01	15.74
GOTERM_BP_ALL	GO:0015988~energy coupled proton transmembrane transport, against electrochemical gradient	3	1.62	4.27E-02	ENSMODG00000009825, ENSMODG00000009431, ENSMODG00000014803	9.02	7.79E-01	52.60
GOTERM_BP_ALL	GO:0090662~ATP hydrolysis coupled transmembrane transport	3	1.62	4.27E-02	ENSMODG00000009825, ENSMODG00000009431, ENSMODG00000014803	9.02	7.79E-01	52.60
GOTERM_BP_ALL	GO:0015991~ATP hydrolysis coupled proton transport	3	1.62	4.27E-02	ENSMODG00000009825, ENSMODG00000009431, ENSMODG00000014803	9.02	7.79E-01	52.60
GOTERM_BP_ALL	GO:1902600~hydrogen ion transmembrane transport	3	1.62	9.48E-02	ENSMODG00000009825, ENSMODG00000009431, ENSMODG00000014803	5.74	9.38E-01	81.78
GOTERM_BP_ALL	GO:0015992~proton transport	3	1.62	1.21E-01	ENSMODG00000009825, ENSMODG00000009431, ENSMODG00000014803	4.95	9.59E-01	89.03
GOTERM_BP_ALL	GO:0006818~hydrogen transport	3	1.62	1.25E-01	ENSMODG00000009825, ENSMODG00000009431, ENSMODG00000014803	4.86	9.60E-01	89.84
Annotation Cluster 10	Cation transport	Enrichment Score	0.83					
Category	Term	Count	%	PValue	Genes	Fold Enrichment	Benjamini	FDR
GOTERM_MF_ALL	GO:0036442~hydrogen-exporting ATPase activity	3	1.62	2.28E-02	ENSMODG00000019574, ENSMODG00000009431, ENSMODG00000014803	12.64	5.71E-01	27.83
GOTERM_MF_ALL	GO:0042625~ATPase coupled ion transmembrane transporter activity	3	1.62	9.39E-02	ENSMODG00000019574, ENSMODG00000009431, ENSMODG00000014803	5.77	8.36E-01	75.20
GOTERM_MF_ALL	GO:0019829~cation-transporting ATPase activity	3	1.62	9.39E-02	ENSMODG00000019574, ENSMODG00000009431, ENSMODG00000014803	5.77	8.36E-01	75.20
GOTERM_MF_ALL	GO:0022853~active ion transmembrane transporter activity	3	1.62	1.85E-01	ENSMODG00000019574, ENSMODG00000009431, ENSMODG00000014803	3.79	9.45E-01	94.47
GOTERM_MF_ALL	GO:0042626~ATPase activity, coupled to transmembrane movement of substances	3	1.62	2.51E-01	ENSMODG00000019574, ENSMODG00000009431, ENSMODG00000014803	3.09	9.58E-01	98.32
GOTERM_MF_ALL	GO:0015399~primary active transmembrane transporter activity	3	1.62	2.76E-01	ENSMODG00000019574, ENSMODG00000009431, ENSMODG00000014803	2.88	9.63E-01	98.96
GOTERM_MF_ALL	GO:0015405~P-P-bond-hydrolysis-driven transmembrane transporter activity	3	1.62	2.76E-01	ENSMODG00000019574, ENSMODG00000009431, ENSMODG00000014803	2.88	9.63E-01	98.96
GOTERM_MF_ALL	GO:0043492~ATPase activity, coupled to movement of substances	3	1.62	3.13E-01	ENSMODG00000019574, ENSMODG00000009431, ENSMODG00000014803	2.63	9.70E-01	99.51
Annotation Cluster 11	GTPase function	Enrichment Score	0.82					
Category	Term	Count	%	PValue	Genes	Fold Enrichment	Benjamini	FDR
GOTERM_MF_ALL	GO:0017016~Ras GTPase binding	3	1.62	1.49E-01	ENSMODG00000020355, ENSMODG00000010704, ENSMODG00000007477	4.35	9.28E-01	89.85
GOTERM_MF_ALL	GO:0031267~small GTPase binding	3	1.62	1.49E-01	ENSMODG00000020355, ENSMODG00000010704, ENSMODG00000007477	4.35	9.28E-01	89.85
GOTERM_MF_ALL	GO:0051020~GTPase binding	3	1.62	1.53E-01	ENSMODG00000020355, ENSMODG00000010704, ENSMODG00000007477	4.28	9.27E-01	90.49
Annotation Cluster 12	Metabolism	Enrichment Score	0.74					
Category	Term	Count	%	PValue	Genes	Fold Enrichment	Benjamini	FDR
GOTERM_BP_ALL	GO:0046496~nicotinamide nucleotide metabolic process	3	1.62	1.70E-01	ENSMODG00000002787, ENSMODG00000023271, ENSMODG00000003073	4.01	9.77E-01	95.87
GOTERM_BP_ALL	GO:0019362~pyridine nucleotide metabolic process	3	1.62	1.70E-01	ENSMODG00000002787, ENSMODG00000023271, ENSMODG00000003073	4.01	9.77E-01	95.87
GOTERM_BP_ALL	GO:0072524~pyridine-containing compound metabolic process	3	1.62	1.78E-01	ENSMODG00000002787, ENSMODG00000023271, ENSMODG00000003073	3.89	9.79E-01	96.53

GOTERM_BP_ALL	GO:0006733~oxidoreduction coenzyme metabolic process	3	1.62	2.21E-01	ENSMODG00000002787, ENSMODG00000023271, ENSMODG00000003073	3.37	9.89E-01	98.61
Annotation Cluster 13	Response to stimulus	Enrichment Score	0.72					
Category	Term	Count	%	PValue	Genes	Fold Enrichment	Benjamini	FDR
GOTERM_BP_ALL	GO:0031668~cellular response to extracellular stimulus	4	2.16	1.19E-01	ENSMODG00000019764, ENSMODG00000004298, ENSMODG00000016512, ENSMODG00000002182	3.30	9.58E-01	88.60
GOTERM_BP_ALL	GO:0071496~cellular response to external stimulus	4	2.16	2.07E-01	ENSMODG00000019764, ENSMODG00000004298, ENSMODG00000016512, ENSMODG00000002182	2.53	9.86E-01	98.11
GOTERM_BP_ALL	GO:0009991~response to extracellular stimulus	4	2.16	2.89E-01	ENSMODG00000019764, ENSMODG00000004298, ENSMODG00000016512, ENSMODG00000002182	2.12	9.95E-01	99.71
Annotation Cluster 14	Nucleotide binding	Enrichment Score	0.66					
Category	Term	Count	%	PValue	Genes	Fold Enrichment	Benjamini	FDR
GOTERM_MF_ALL	GO:0005525~GTP binding	6	3.24	2.00E-01	ENSMODG00000016955, ENSMODG00000004576, ENSMODG00000008253, ENSMODG00000016512, ENSMODG00000024936, ENSMODG00000000508	1.92	9.49E-01	95.74
GOTERM_MF_ALL	GO:0032561~guanyl ribonucleotide binding	6	3.24	2.26E-01	ENSMODG00000016955, ENSMODG00000004576, ENSMODG00000008253, ENSMODG00000016512, ENSMODG00000024936, ENSMODG00000000508	1.83	9.56E-01	97.34
GOTERM_MF_ALL	GO:0019001~guanyl nucleotide binding	6	3.24	2.28E-01	ENSMODG00000016955, ENSMODG00000004576, ENSMODG00000008253, ENSMODG00000016512, ENSMODG00000024936, ENSMODG00000000508	1.82	9.54E-01	97.43
Annotation Cluster 15	Nutrient response	Enrichment Score	0.51					
Category	Term	Count	%	PValue	Genes	Fold Enrichment	Benjamini	FDR
GOTERM_BP_ALL	GO:0009267~cellular response to starvation	3	1.62	2.30E-01	ENSMODG00000019764, ENSMODG00000016512, ENSMODG00000002182	3.28	9.90E-01	98.86
GOTERM_BP_ALL	GO:0042594~response to starvation	3	1.62	2.70E-01	ENSMODG00000019764, ENSMODG00000016512, ENSMODG00000002182	2.94	9.94E-01	99.53
GOTERM_BP_ALL	GO:0031669~cellular response to nutrient levels	3	1.62	2.91E-01	ENSMODG00000019764, ENSMODG00000016512, ENSMODG00000002182	2.78	9.95E-01	99.72
GOTERM_BP_ALL	GO:0031667~response to nutrient levels	3	1.62	5.16E-01	ENSMODG00000019764, ENSMODG00000016512, ENSMODG00000002182	1.73	9.99E-01	100.00
Annotation Cluster 16	Catabolism	Enrichment Score	0.48					
Category	Term	Count	%	PValue	Genes	Fold Enrichment	Benjamini	FDR
GOTERM_BP_ALL	GO:0043161~proteasome-mediated ubiquitin-dependent protein catabolic process	6	3.24	1.56E-01	ENSMODG00000010088, ENSMODG00000008145, ENSMODG00000009940, ENSMODG00000012664, ENSMODG00000015616, ENSMODG00000004556	2.10	9.72E-01	94.48
GOTERM_BP_ALL	GO:0010498~proteasomal protein catabolic process	6	3.24	1.93E-01	ENSMODG00000010088, ENSMODG00000008145, ENSMODG00000009940, ENSMODG00000012664, ENSMODG00000015616, ENSMODG00000004556	1.94	9.84E-01	97.45
GOTERM_BP_ALL	GO:0006511~ubiquitin-dependent protein catabolic process	6	3.24	4.87E-01	ENSMODG00000010088, ENSMODG00000008145, ENSMODG00000009940, ENSMODG00000012664, ENSMODG00000015616, ENSMODG00000004556	1.30	9.99E-01	100.00
GOTERM_BP_ALL	GO:0019941~modification-dependent protein catabolic process	6	3.24	5.03E-01	ENSMODG00000010088, ENSMODG00000008145, ENSMODG00000009940, ENSMODG00000012664, ENSMODG00000015616, ENSMODG00000004556	1.28	9.99E-01	100.00
GOTERM_BP_ALL	GO:0043632~modification-dependent macromolecule catabolic process	6	3.24	5.18E-01	ENSMODG00000010088, ENSMODG00000008145, ENSMODG00000009940, ENSMODG00000012664, ENSMODG00000015616, ENSMODG00000004556	1.25	9.99E-01	100.00
Annotation Cluster 17	Ion transport	Enrichment Score	0.48					
Category	Term	Count	%	PValue	Genes	Fold Enrichment	Benjamini	FDR
GOTERM_MF_ALL	GO:0005216~ion channel activity	6	3.24	2.98E-01	ENSMODG00000001049, ENSMODG00000013130, ENSMODG00000018058, ENSMODG00000016353, ENSMODG00000001335, ENSMODG00000001018	1.64	9.69E-01	99.34
GOTERM_MF_ALL	GO:0022838~substrate-specific channel activity	6	3.24	3.16E-01	ENSMODG00000001049, ENSMODG00000013130, ENSMODG00000018058, ENSMODG00000016353, ENSMODG00000001335, ENSMODG00000001018	1.60	9.69E-01	99.54
GOTERM_MF_ALL	GO:0022803~passive transmembrane transporter activity	6	3.24	3.52E-01	ENSMODG00000001049, ENSMODG00000013130, ENSMODG00000018058, ENSMODG00000016353, ENSMODG00000001335, ENSMODG00000001018	1.53	9.76E-01	99.78
GOTERM_MF_ALL	GO:0015267~channel activity	6	3.24	3.52E-01	ENSMODG00000001049, ENSMODG00000013130, ENSMODG00000018058, ENSMODG00000016353, ENSMODG00000001335, ENSMODG00000001018	1.53	9.76E-01	99.78
Annotation Cluster 18	Proteolysis	Enrichment Score	0.42					
Category	Term	Count	%	PValue	Genes	Fold Enrichment	Benjamini	FDR

GOTERM_BP_ALL	GO:0051603~proteolysis involved in cellular protein catabolic process	8	4.32	3.09E-01	ENSMODG00000010088, ENSMODG00000019764, ENSMODG00000008145, ENSMODG00000009940, ENSMODG00000012664, ENSMODG00000015616, ENSMODG00000004556, ENSMODG00000012957	1.45	9.96E-01	99.82
GOTERM_BP_ALL	GO:0044257~cellular protein catabolic process	8	4.32	3.55E-01	ENSMODG00000010088, ENSMODG00000019764, ENSMODG00000008145, ENSMODG00000009940, ENSMODG00000012664, ENSMODG00000015616, ENSMODG00000004556, ENSMODG00000012957	1.38	9.97E-01	99.94
GOTERM_BP_ALL	GO:0030163~protein catabolic process	8	4.32	5.17E-01	ENSMODG00000010088, ENSMODG00000019764, ENSMODG00000008145, ENSMODG00000009940, ENSMODG00000012664, ENSMODG00000015616, ENSMODG00000004556, ENSMODG00000012957	1.18	9.99E-01	100.00
Annotation Cluster 19	Hypoxia	Enrichment Score	0.40					
Category	Term	Count	%	PValue	Genes	Fold Enrichment	Benjamini	FDR
GOTERM_BP_ALL	GO:0001666~response to hypoxia	3	1.62	3.86E-01	ENSMODG00000013318, ENSMODG00000007498, ENSMODG00000004298	2.23	9.98E-01	99.98
GOTERM_BP_ALL	GO:0036293~response to decreased oxygen levels	3	1.62	3.90E-01	ENSMODG00000013318, ENSMODG00000007498, ENSMODG00000004298	2.22	9.98E-01	99.98
GOTERM_BP_ALL	GO:0070482~response to oxygen levels	3	1.62	4.07E-01	ENSMODG00000013318, ENSMODG00000007498, ENSMODG00000004298	2.14	9.98E-01	99.99
Annotation Cluster 20	Immunity	Enrichment Score	0.39					
Category	Term	Count	%	PValue	Genes	Fold Enrichment	Benjamini	FDR
GOTERM_BP_ALL	GO:0098792~xenophagy	3	1.62	2.17E-01	ENSMODG00000024759, ENSMODG00000019878, ENSMODG00000003073	3.41	9.88E-01	98.48
GOTERM_BP_ALL	GO:0002230~positive regulation of defense response to virus by host	3	1.62	2.39E-01	ENSMODG00000024759, ENSMODG00000019878, ENSMODG00000003073	3.20	9.91E-01	99.06
GOTERM_BP_ALL	GO:0050691~regulation of defense response to virus by host	3	1.62	2.56E-01	ENSMODG00000024759, ENSMODG00000019878, ENSMODG00000003073	3.04	9.93E-01	99.37
GOTERM_BP_ALL	GO:0098780~response to mitochondrial depolarisation	3	1.62	3.09E-01	ENSMODG00000024759, ENSMODG00000019878, ENSMODG00000003073	2.66	9.96E-01	99.82
GOTERM_BP_ALL	GO:0098779~mitophagy in response to mitochondrial depolarization	3	1.62	3.09E-01	ENSMODG00000024759, ENSMODG00000019878, ENSMODG00000003073	2.66	9.96E-01	99.82
GOTERM_BP_ALL	GO:0000423~macromitophagy	3	1.62	3.18E-01	ENSMODG00000024759, ENSMODG00000019878, ENSMODG00000003073	2.60	9.96E-01	99.85
GOTERM_BP_ALL	GO:0050688~regulation of defense response to virus	3	1.62	3.22E-01	ENSMODG00000024759, ENSMODG00000019878, ENSMODG00000003073	2.58	9.96E-01	99.87
GOTERM_BP_ALL	GO:0000422~mitophagy	3	1.62	4.35E-01	ENSMODG00000024759, ENSMODG00000019878, ENSMODG00000003073	2.02	9.99E-01	99.99
GOTERM_BP_ALL	GO:0061726~mitochondrion disassembly	3	1.62	4.35E-01	ENSMODG00000024759, ENSMODG00000019878, ENSMODG00000003073	2.02	9.99E-01	99.99
GOTERM_BP_ALL	GO:0002831~regulation of response to biotic stimulus	3	1.62	4.59E-01	ENSMODG00000024759, ENSMODG00000019878, ENSMODG00000003073	1.93	9.99E-01	100.00
GOTERM_BP_ALL	GO:1903008~organelle disassembly	3	1.62	4.94E-01	ENSMODG00000024759, ENSMODG00000019878, ENSMODG00000003073	1.80	9.99E-01	100.00
GOTERM_BP_ALL	GO:0051607~defense response to virus	3	1.62	5.31E-01	ENSMODG00000024759, ENSMODG00000019878, ENSMODG00000003073	1.68	9.99E-01	100.00
GOTERM_BP_ALL	GO:0009615~response to virus	3	1.62	6.59E-01	ENSMODG00000024759, ENSMODG00000019878, ENSMODG00000003073	1.33	1.00E+00	100.00
GOTERM_BP_ALL	GO:0002697~regulation of immune effector process	3	1.62	7.39E-01	ENSMODG00000024759, ENSMODG00000019878, ENSMODG00000003073	1.14	1.00E+00	100.00
GOTERM_BP_ALL	GO:0031347~regulation of defense response	3	1.62	9.22E-01	ENSMODG00000024759, ENSMODG00000019878, ENSMODG00000003073	0.72	1.00E+00	100.00
Annotation Cluster 21	Apoptosis	Enrichment Score	0.37					
Category	Term	Count	%	PValue	Genes	Fold Enrichment	Benjamini	FDR
GOTERM_BP_ALL	GO:0043523~regulation of neuron apoptotic process	3	1.62	3.39E-01	ENSMODG00000013318, ENSMODG00000004298, ENSMODG00000007925	2.48	9.97E-01	99.92
GOTERM_BP_ALL	GO:1901214~regulation of neuron death	3	1.62	4.55E-01	ENSMODG00000013318, ENSMODG00000004298, ENSMODG00000007925	1.94	9.99E-01	100.00
GOTERM_BP_ALL	GO:0070997~neuron death	3	1.62	4.94E-01	ENSMODG00000013318, ENSMODG00000004298, ENSMODG00000007925	1.80	9.99E-01	100.00
Annotation Cluster 22	Nucleic acid binding	Enrichment Score	0.37					
Category	Term	Count	%	PValue	Genes	Fold Enrichment	Benjamini	FDR
GOTERM_MF_ALL	GO:0035639~purine ribonucleoside triphosphate binding	17	9.19	4.08E-01	ENSMODG00000016955, ENSMODG00000007831, ENSMODG00000013318, ENSMODG00000000983, ENSMODG00000012957, ENSMODG00000000508, ENSMODG00000017363, ENSMODG00000020949, ENSMODG00000019764, ENSMODG00000017776, ENSMODG00000004576, ENSMODG00000010647, ENSMODG00000008253, ENSMODG00000009825, ENSMODG00000020405, ENSMODG00000016512, ENSMODG00000024936	1.14	9.85E-01	99.94
GOTERM_MF_ALL	GO:0032550~purine ribonucleoside binding	17	9.19	4.16E-01	ENSMODG00000016955, ENSMODG00000007831, ENSMODG00000013318, ENSMODG00000000983, ENSMODG00000012957, ENSMODG00000000508, ENSMODG00000017363, ENSMODG00000020949, ENSMODG00000019764, ENSMODG00000017776, ENSMODG00000004576, ENSMODG00000010647, ENSMODG00000008253, ENSMODG00000009825, ENSMODG00000020405, ENSMODG00000016512, ENSMODG00000024936	1.13	9.85E-01	99.95

GOTERM_MF_ALL	GO:0001883~purine nucleoside binding	17	9.19	4.17E-01	ENSMODG00000016955, ENSMODG00000007831, ENSMODG00000013318, ENSMODG00000000983, ENSMODG00000012957, ENSMODG00000000508, ENSMODG00000017363, ENSMODG00000020949, ENSMODG00000019764, ENSMODG00000017776, ENSMODG00000004576, ENSMODG00000010647, ENSMODG00000008253, ENSMODG00000009825, ENSMODG00000020405, ENSMODG00000016512, ENSMODG00000024936	1.13	9.85E-01	99.95
GOTERM_MF_ALL	GO:0032549~ribonucleoside binding	17	9.19	4.20E-01	ENSMODG00000016955, ENSMODG00000007831, ENSMODG00000013318, ENSMODG00000000983, ENSMODG00000012957, ENSMODG00000000508, ENSMODG00000017363, ENSMODG00000020949, ENSMODG00000019764, ENSMODG00000017776, ENSMODG00000004576, ENSMODG00000010647, ENSMODG00000008253, ENSMODG00000009825, ENSMODG00000020405, ENSMODG00000016512, ENSMODG00000024936	1.13	9.84E-01	99.95
GOTERM_MF_ALL	GO:0001882~nucleoside binding	17	9.19	4.25E-01	ENSMODG00000016955, ENSMODG00000007831, ENSMODG00000013318, ENSMODG00000000983, ENSMODG00000012957, ENSMODG00000000508, ENSMODG00000017363, ENSMODG00000020949, ENSMODG00000019764, ENSMODG00000017776, ENSMODG00000004576, ENSMODG00000010647, ENSMODG00000008253, ENSMODG00000009825, ENSMODG00000020405, ENSMODG00000016512, ENSMODG00000024936	1.13	9.84E-01	99.96
GOTERM_MF_ALL	GO:0032555~purine ribonucleotide binding	17	9.19	4.42E-01	ENSMODG00000016955, ENSMODG00000007831, ENSMODG00000013318, ENSMODG00000000983, ENSMODG00000012957, ENSMODG00000000508, ENSMODG00000017363, ENSMODG00000020949, ENSMODG00000019764, ENSMODG00000017776, ENSMODG00000004576, ENSMODG00000010647, ENSMODG00000008253, ENSMODG00000009825, ENSMODG00000020405, ENSMODG00000016512, ENSMODG00000024936	1.12	9.85E-01	99.97
GOTERM_MF_ALL	GO:0017076~purine nucleotide binding	17	9.19	4.50E-01	ENSMODG00000016955, ENSMODG00000007831, ENSMODG00000013318, ENSMODG00000000983, ENSMODG00000012957, ENSMODG00000000508, ENSMODG00000017363, ENSMODG00000020949, ENSMODG00000019764, ENSMODG00000017776, ENSMODG00000004576, ENSMODG00000010647, ENSMODG00000008253, ENSMODG00000009825, ENSMODG00000020405, ENSMODG00000016512, ENSMODG00000024936	1.11	9.85E-01	99.98
GOTERM_MF_ALL	GO:0032553~ribonucleotide binding	17	9.19	4.57E-01	ENSMODG00000016955, ENSMODG00000007831, ENSMODG00000013318, ENSMODG00000000983, ENSMODG00000012957, ENSMODG00000000508, ENSMODG00000017363, ENSMODG00000020949, ENSMODG00000019764, ENSMODG00000017776, ENSMODG00000004576, ENSMODG00000010647, ENSMODG00000008253, ENSMODG00000009825, ENSMODG00000020405, ENSMODG00000016512, ENSMODG00000024936	1.11	9.83E-01	99.98
Annotation Cluster 23	Endocrine function	Enrichment Score	0.35					
Category	Term	Count	%	PValue	Genes	Fold Enrichment	Benjamini	FDR
GOTERM_BP_ALL	GO:0046887~positive regulation of hormone secretion	3	1.62	1.45E-01	ENSMODG00000010647, ENSMODG00000013280, ENSMODG00000002787	4.43	9.68E-01	93.16
GOTERM_BP_ALL	GO:0046883~regulation of hormone secretion	3	1.62	4.11E-01	ENSMODG00000010647, ENSMODG00000013280, ENSMODG00000002787	2.12	9.98E-01	99.99
GOTERM_BP_ALL	GO:0046879~hormone secretion	3	1.62	5.38E-01	ENSMODG00000010647, ENSMODG00000013280, ENSMODG00000002787	1.66	9.99E-01	100.00
GOTERM_BP_ALL	GO:1903532~positive regulation of secretion by cell	3	1.62	5.69E-01	ENSMODG00000010647, ENSMODG00000013280, ENSMODG00000002787	1.57	1.00E+00	100.00
GOTERM_BP_ALL	GO:0051047~positive regulation of secretion	3	1.62	5.99E-01	ENSMODG00000010647, ENSMODG00000013280, ENSMODG00000002787	1.49	1.00E+00	100.00
GOTERM_BP_ALL	GO:0023061~signal release	3	1.62	7.39E-01	ENSMODG00000010647, ENSMODG00000013280, ENSMODG00000002787	1.14	1.00E+00	100.00
Annotation Cluster 24	Cell motility	Enrichment Score	0.31					
Category	Term	Count	%	PValue	Genes	Fold Enrichment	Benjamini	FDR
GOTERM_BP_ALL	GO:0010634~positive regulation of epithelial cell migration	3	1.62	2.04E-01	ENSMODG00000016955, ENSMODG00000010647, ENSMODG00000011701	3.56	9.86E-01	97.98
GOTERM_BP_ALL	GO:0010632~regulation of epithelial cell migration	3	1.62	4.11E-01	ENSMODG00000016955, ENSMODG00000010647, ENSMODG00000011701	2.12	9.98E-01	99.99
GOTERM_BP_ALL	GO:0010631~epithelial cell migration	3	1.62	5.79E-01	ENSMODG00000016955, ENSMODG00000010647, ENSMODG00000011701	1.54	1.00E+00	100.00
GOTERM_BP_ALL	GO:0090132~epithelium migration	3	1.62	5.86E-01	ENSMODG00000016955, ENSMODG00000010647, ENSMODG00000011701	1.52	1.00E+00	100.00
GOTERM_BP_ALL	GO:0090130~tissue migration	3	1.62	5.99E-01	ENSMODG00000016955, ENSMODG00000010647, ENSMODG00000011701	1.49	1.00E+00	100.00
GOTERM_BP_ALL	GO:0001667~ameboidal-type cell migration	3	1.62	7.75E-01	ENSMODG00000016955, ENSMODG00000010647, ENSMODG00000011701	1.06	1.00E+00	100.00
Annotation Cluster 25	Response to stimulus	Enrichment Score	0.27					
Category	Term	Count	%	PValue	Genes	Fold Enrichment	Benjamini	FDR

GOTERM_BP_ALL	GO:0098542~defense response to other organism	5	2.70	3.17E-01	ENSMODG00000024759, ENSMODG00000019878, ENSMODG00000013049, ENSMODG00000012130, ENSMODG00000003073	1.75	9.96E-01	99.85
GOTERM_BP_ALL	GO:0043207~response to external biotic stimulus	5	2.70	7.03E-01	ENSMODG00000024759, ENSMODG00000019878, ENSMODG00000013049, ENSMODG00000012130, ENSMODG00000003073	1.05	1.00E+00	100.00
GOTERM_BP_ALL	GO:0051707~response to other organism	5	2.70	7.03E-01	ENSMODG00000024759, ENSMODG00000019878, ENSMODG00000013049, ENSMODG00000012130, ENSMODG00000003073	1.05	1.00E+00	100.00
Annotation Cluster 26	Immunity	Enrichment Score	0.27					
Category	Term	Count	%	PValue	Genes	Fold Enrichment	Benjamini	FDR
GOTERM_BP_ALL	GO:0050792~regulation of viral process	4	2.16	3.63E-01	ENSMODG00000024759, ENSMODG00000007831, ENSMODG00000019878, ENSMODG00000003073	1.85	9.97E-01	99.96
GOTERM_BP_ALL	GO:0043903~regulation of symbiosis, encompassing mutualism through parasitism	4	2.16	4.08E-01	ENSMODG00000024759, ENSMODG00000007831, ENSMODG00000019878, ENSMODG00000003073	1.72	9.98E-01	99.99
GOTERM_BP_ALL	GO:0043900~regulation of multi-organism process	4	2.16	5.82E-01	ENSMODG00000024759, ENSMODG00000007831, ENSMODG00000019878, ENSMODG00000003073	1.32	1.00E+00	100.00
GOTERM_BP_ALL	GO:0016032~viral process	4	2.16	7.37E-01	ENSMODG00000024759, ENSMODG00000007831, ENSMODG00000019878, ENSMODG00000003073	1.05	1.00E+00	100.00
GOTERM_BP_ALL	GO:0044764~multi-organism cellular process	4	2.16	7.42E-01	ENSMODG00000024759, ENSMODG00000007831, ENSMODG00000019878, ENSMODG00000003073	1.04	1.00E+00	100.00
Annotation Cluster 27	Cell motility	Enrichment Score	0.23					
Category	Term	Count	%	PValue	Genes	Fold Enrichment	Benjamini	FDR
GOTERM_BP_ALL	GO:0030335~positive regulation of cell migration	4	2.16	5.74E-01	ENSMODG00000016955, ENSMODG00000019764, ENSMODG00000010647, ENSMODG00000011701	1.34	1.00E+00	100.00
GOTERM_BP_ALL	GO:2000147~positive regulation of cell motility	4	2.16	5.85E-01	ENSMODG00000016955, ENSMODG00000019764, ENSMODG00000010647, ENSMODG00000011701	1.32	1.00E+00	100.00
GOTERM_BP_ALL	GO:0051272~positive regulation of cellular component movement	4	2.16	5.98E-01	ENSMODG00000016955, ENSMODG00000019764, ENSMODG00000010647, ENSMODG00000011701	1.29	1.00E+00	100.00
GOTERM_BP_ALL	GO:0040017~positive regulation of locomotion	4	2.16	6.06E-01	ENSMODG00000016955, ENSMODG00000019764, ENSMODG00000010647, ENSMODG00000011701	1.28	1.00E+00	100.00
Annotation Cluster 28	Hydrolase activity	Enrichment Score	0.22					
Category	Term	Count	%	PValue	Genes	Fold Enrichment	Benjamini	FDR
GOTERM_MF_ALL	GO:0017111~nucleoside-triphosphatase activity	7	3.78	5.59E-01	ENSMODG00000019764, ENSMODG00000004576, ENSMODG00000019574, ENSMODG00000009431, ENSMODG00000012957, ENSMODG00000014803, ENSMODG00000000508	1.16	9.91E-01	100.00
GOTERM_MF_ALL	GO:0016462~pyrophosphatase activity	7	3.78	6.17E-01	ENSMODG00000019764, ENSMODG00000004576, ENSMODG00000019574, ENSMODG00000009431, ENSMODG00000012957, ENSMODG00000014803, ENSMODG00000000508	1.09	9.95E-01	100.00
GOTERM_MF_ALL	GO:0016818~hydrolase activity, acting on acid anhydrides, in phosphorus-containing anhydrides	7	3.78	6.20E-01	ENSMODG00000019764, ENSMODG00000004576, ENSMODG00000019574, ENSMODG00000009431, ENSMODG00000012957, ENSMODG00000014803, ENSMODG00000000508	1.09	9.94E-01	100.00
Annotation Cluster 29	Metabolism	Enrichment Score	0.19					
Category	Term	Count	%	PValue	Genes	Fold Enrichment	Benjamini	FDR
GOTERM_BP_ALL	GO:0009205~purine ribonucleoside triphosphate metabolic process	3	1.62	4.55E-01	ENSMODG00000017363, ENSMODG00000019459, ENSMODG00000009825	1.94	9.99E-01	100.00
GOTERM_BP_ALL	GO:0009199~ribonucleoside triphosphate metabolic process	3	1.62	4.75E-01	ENSMODG00000017363, ENSMODG00000019459, ENSMODG00000009825	1.87	9.99E-01	100.00
GOTERM_BP_ALL	GO:0009144~purine nucleoside triphosphate metabolic process	3	1.62	4.75E-01	ENSMODG00000017363, ENSMODG00000019459, ENSMODG00000009825	1.87	9.99E-01	100.00
GOTERM_BP_ALL	GO:0009141~nucleoside triphosphate metabolic process	3	1.62	5.45E-01	ENSMODG00000017363, ENSMODG00000019459, ENSMODG00000009825	1.64	9.99E-01	100.00
GOTERM_BP_ALL	GO:0046128~purine ribonucleoside metabolic process	3	1.62	6.02E-01	ENSMODG00000017363, ENSMODG00000019459, ENSMODG00000009825	1.48	1.00E+00	100.00
GOTERM_BP_ALL	GO:0042278~purine nucleoside metabolic process	3	1.62	6.12E-01	ENSMODG00000017363, ENSMODG00000019459, ENSMODG00000009825	1.45	1.00E+00	100.00
GOTERM_BP_ALL	GO:0009119~ribonucleoside metabolic process	3	1.62	6.54E-01	ENSMODG00000017363, ENSMODG00000019459, ENSMODG00000009825	1.34	1.00E+00	100.00
GOTERM_BP_ALL	GO:0009116~nucleoside metabolic process	3	1.62	7.00E-01	ENSMODG00000017363, ENSMODG00000019459, ENSMODG00000009825	1.23	1.00E+00	100.00
GOTERM_BP_ALL	GO:1901657~glycosyl compound metabolic process	3	1.62	7.29E-01	ENSMODG00000017363, ENSMODG00000019459, ENSMODG00000009825	1.16	1.00E+00	100.00
GOTERM_BP_ALL	GO:0009150~purine ribonucleotide metabolic process	3	1.62	8.05E-01	ENSMODG00000017363, ENSMODG00000019459, ENSMODG00000009825	0.99	1.00E+00	100.00
GOTERM_BP_ALL	GO:0009259~ribonucleotide metabolic process	3	1.62	8.24E-01	ENSMODG00000017363, ENSMODG00000019459, ENSMODG00000009825	0.95	1.00E+00	100.00

GOTERM_BP_ALL	GO:0006163~purine nucleotide metabolic process	3	1.62	8.29E-01	ENSMODG00000017363, ENSMODG00000019459, ENSMODG00000009825	0.94	1.00E+00	100.00
GOTERM_BP_ALL	GO:0019693~ribose phosphate metabolic process	3	1.62	8.35E-01	ENSMODG00000017363, ENSMODG00000019459, ENSMODG00000009825	0.93	1.00E+00	100.00
Annotation Cluster 30	Ion transport	Enrichment Score	0.18					
Category	Term	Count	%	PValue	Genes	Fold Enrichment	Benjamini	FDR
GOTERM_CC_ALL	GO:0034702~ion channel complex	3	1.62	6.22E-01	ENSMODG00000002333, ENSMODG00000018058, ENSMODG00000017184	1.42	9.87E-01	100.00
GOTERM_CC_ALL	GO:1902495~transmembrane transporter complex	3	1.62	6.69E-01	ENSMODG00000002333, ENSMODG00000018058, ENSMODG00000017184	1.31	9.91E-01	100.00
GOTERM_CC_ALL	GO:1990351~transporter complex	3	1.62	6.80E-01	ENSMODG00000002333, ENSMODG00000018058, ENSMODG00000017184	1.28	9.90E-01	100.00
Annotation Cluster 31	Catabolism	Enrichment Score	0.16					
Category	Term	Count	%	PValue	Genes	Fold Enrichment	Benjamini	FDR
GOTERM_BP_ALL	GO:0044270~cellular nitrogen compound catabolic process	3	1.62	6.76E-01	ENSMODG00000016406, ENSMODG00000006673, ENSMODG00000014990	1.29	1.00E+00	100.00
GOTERM_BP_ALL	GO:0046700~heterocycle catabolic process	3	1.62	6.76E-01	ENSMODG00000016406, ENSMODG00000006673, ENSMODG00000014990	1.29	1.00E+00	100.00
GOTERM_BP_ALL	GO:0019439~aromatic compound catabolic process	3	1.62	6.87E-01	ENSMODG00000016406, ENSMODG00000006673, ENSMODG00000014990	1.26	1.00E+00	100.00
GOTERM_BP_ALL	GO:1901361~organic cyclic compound catabolic process	3	1.62	7.15E-01	ENSMODG00000016406, ENSMODG00000006673, ENSMODG00000014990	1.20	1.00E+00	100.00
Annotation Cluster 32	Transcriptional regulation	Enrichment Score	0.14					
Category	Term	Count	%	PValue	Genes	Fold Enrichment	Benjamini	FDR
GOTERM_MF_ALL	GO:0003712~transcription cofactor activity	4	2.16	6.65E-01	ENSMODG00000007725, ENSMODG00000016888, ENSMODG00000012475, ENSMODG00000023300	1.17	9.97E-01	100.00
GOTERM_MF_ALL	GO:0000989~transcription factor activity, transcription factor binding	4	2.16	7.52E-01	ENSMODG00000007725, ENSMODG00000016888, ENSMODG00000012475, ENSMODG00000023300	1.02	9.99E-01	100.00
GOTERM_MF_ALL	GO:0000988~transcription factor activity, protein binding	4	2.16	7.59E-01	ENSMODG00000007725, ENSMODG00000016888, ENSMODG00000012475, ENSMODG00000023300	1.01	9.99E-01	100.00
Annotation Cluster 33	Protein transport	Enrichment Score	0.14					
Category	Term	Count	%	PValue	Genes	Fold Enrichment	Benjamini	FDR
GOTERM_BP_ALL	GO:0090316~positive regulation of intracellular protein transport	3	1.62	5.92E-01	ENSMODG00000020355, ENSMODG00000004556, ENSMODG00000015800	1.50	1.00E+00	100.00
GOTERM_BP_ALL	GO:0032388~positive regulation of intracellular transport	3	1.62	6.39E-01	ENSMODG00000020355, ENSMODG00000004556, ENSMODG00000015800	1.38	1.00E+00	100.00
GOTERM_BP_ALL	GO:1903829~positive regulation of cellular protein localization	3	1.62	7.52E-01	ENSMODG00000020355, ENSMODG00000004556, ENSMODG00000015800	1.11	1.00E+00	100.00
GOTERM_BP_ALL	GO:0033157~regulation of intracellular protein transport	3	1.62	7.89E-01	ENSMODG00000020355, ENSMODG00000004556, ENSMODG00000015800	1.03	1.00E+00	100.00
GOTERM_BP_ALL	GO:1903827~regulation of cellular protein localization	3	1.62	9.29E-01	ENSMODG00000020355, ENSMODG00000004556, ENSMODG00000015800	0.70	1.00E+00	100.00
Annotation Cluster 34	Biosynthesis	Enrichment Score	0.12					
Category	Term	Count	%	PValue	Genes	Fold Enrichment	Benjamini	FDR
GOTERM_BP_ALL	GO:0006412~translation	5	2.70	7.23E-01	ENSMODG00000006673, ENSMODG00000007498, ENSMODG00000002920, ENSMODG00000020405, ENSMODG00000021351	1.02	1.00E+00	100.00
GOTERM_BP_ALL	GO:0043043~peptide biosynthetic process	5	2.70	7.42E-01	ENSMODG00000006673, ENSMODG00000007498, ENSMODG00000002920, ENSMODG00000020405, ENSMODG00000021351	0.99	1.00E+00	100.00
GOTERM_BP_ALL	GO:0043604~amide biosynthetic process	5	2.70	8.18E-01	ENSMODG00000006673, ENSMODG00000007498, ENSMODG00000002920, ENSMODG00000020405, ENSMODG00000021351	0.88	1.00E+00	100.00
Annotation Cluster 35	Nucleotide binding	Enrichment Score	0.11					
Category	Term	Count	%	PValue	Genes	Fold Enrichment	Benjamini	FDR
GOTERM_MF_ALL	GO:0005524~ATP binding	11	5.95	7.53E-01	ENSMODG00000017363, ENSMODG00000007831, ENSMODG00000020949, ENSMODG00000019764, ENSMODG00000017776, ENSMODG00000013318, ENSMODG00000000983, ENSMODG00000010647, ENSMODG00000009825, ENSMODG00000020405, ENSMODG00000012957	0.93	9.99E-01	100.00
GOTERM_MF_ALL	GO:0032559~adenyl ribonucleotide binding	11	5.95	7.72E-01	ENSMODG00000017363, ENSMODG00000007831, ENSMODG00000020949, ENSMODG00000019764, ENSMODG00000017776, ENSMODG00000013318, ENSMODG00000000983, ENSMODG00000010647, ENSMODG00000009825, ENSMODG00000020405, ENSMODG00000012957	0.91	9.99E-01	100.00

GOTERM_MF_ALL	GO:0030554~adenyl nucleotide binding	11	5.95	7.77E-01	ENSMODG00000017363, ENSMODG00000007831, ENSMODG00000020949, ENSMODG00000019764, ENSMODG00000017776, ENSMODG00000013318, ENSMODG00000000983, ENSMODG00000010647, ENSMODG00000009825, ENSMODG00000020405, ENSMODG00000012957	0.91	9.99E-01	100.00
Annotation Cluster 36	Cell motility	Enrichment Score	0.09					
Category	Term	Count	%	PValue	Genes	Fold Enrichment	Benjamini	FDR
GOTERM_BP_ALL	GO:0030334~regulation of cell migration	5	2.70	7.75E-01	ENSMODG00000016955, ENSMODG00000002333, ENSMODG00000019764, ENSMODG00000010647, ENSMODG00000011701	0.95	1.00E+00	100.00
GOTERM_BP_ALL	GO:2000145~regulation of cell motility	5	2.70	8.03E-01	ENSMODG00000016955, ENSMODG00000002333, ENSMODG00000019764, ENSMODG00000010647, ENSMODG00000011701	0.91	1.00E+00	100.00
GOTERM_BP_ALL	GO:0040012~regulation of locomotion	5	2.70	8.28E-01	ENSMODG00000016955, ENSMODG00000002333, ENSMODG00000019764, ENSMODG00000010647, ENSMODG00000011701	0.87	1.00E+00	100.00
GOTERM_BP_ALL	GO:0051270~regulation of cellular component movement	5	2.70	8.50E-01	ENSMODG00000016955, ENSMODG00000002333, ENSMODG00000019764, ENSMODG00000010647, ENSMODG00000011701	0.84	1.00E+00	100.00
Annotation Cluster 37	Apoptosis	Enrichment Score	0.08					
Category	Term	Count	%	PValue	Genes	Fold Enrichment	Benjamini	FDR
GOTERM_BP_ALL	GO:0043066~negative regulation of apoptotic process	5	2.70	8.15E-01	ENSMODG00000010088, ENSMODG00000019764, ENSMODG00000017776, ENSMODG00000013318, ENSMODG00000004298	0.89	1.00E+00	100.00
GOTERM_BP_ALL	GO:0043069~negative regulation of programmed cell death	5	2.70	8.30E-01	ENSMODG00000010088, ENSMODG00000019764, ENSMODG00000017776, ENSMODG00000013318, ENSMODG00000004298	0.87	1.00E+00	100.00
GOTERM_BP_ALL	GO:0060548~negative regulation of cell death	5	2.70	8.70E-01	ENSMODG00000010088, ENSMODG00000019764, ENSMODG00000017776, ENSMODG00000013318, ENSMODG00000004298	0.81	1.00E+00	100.00
Annotation Cluster 38	Apoptosis	Enrichment Score	0.06					
Category	Term	Count	%	PValue	Genes	Fold Enrichment	Benjamini	FDR
GOTERM_BP_ALL	GO:0042981~regulation of apoptotic process	8	4.32	8.49E-01	ENSMODG00000010088, ENSMODG00000019764, ENSMODG00000017776, ENSMODG00000013318, ENSMODG00000010647, ENSMODG00000004298, ENSMODG00000007925, ENSMODG00000019181	0.83	1.00E+00	100.00
GOTERM_BP_ALL	GO:0043067~regulation of programmed cell death	8	4.32	8.60E-01	ENSMODG00000010088, ENSMODG00000019764, ENSMODG00000017776, ENSMODG00000013318, ENSMODG00000010647, ENSMODG00000004298, ENSMODG00000007925, ENSMODG00000019181	0.82	1.00E+00	100.00
GOTERM_BP_ALL	GO:0010941~regulation of cell death	8	4.32	8.97E-01	ENSMODG00000010088, ENSMODG00000019764, ENSMODG00000017776, ENSMODG00000013318, ENSMODG00000010647, ENSMODG00000004298, ENSMODG00000007925, ENSMODG00000019181	0.77	1.00E+00	100.00
Annotation Cluster 39	Development	Enrichment Score	0.04					
Category	Term	Count	%	PValue	Genes	Fold Enrichment	Benjamini	FDR
GOTERM_BP_ALL	GO:0007420~brain development	4	2.16	8.73E-01	ENSMODG00000019764, ENSMODG00000010647, ENSMODG00000004298, ENSMODG00000008033	0.81	1.00E+00	100.00
GOTERM_BP_ALL	GO:0060322~head development	4	2.16	9.00E-01	ENSMODG00000019764, ENSMODG00000010647, ENSMODG00000004298, ENSMODG00000008033	0.76	1.00E+00	100.00
GOTERM_BP_ALL	GO:0007417~central nervous system development	4	2.16	9.58E-01	ENSMODG00000019764, ENSMODG00000010647, ENSMODG00000004298, ENSMODG00000008033	0.62	1.00E+00	100.00
Annotation Cluster 40	Membrane function	Enrichment Score	0.03					
Category	Term	Count	%	PValue	Genes	Fold Enrichment	Benjamini	FDR
GOTERM_BP_ALL	GO:0072657~protein localization to membrane	3	1.62	8.21E-01	ENSMODG00000004576, ENSMODG00000016096, ENSMODG00000004631	0.96	1.00E+00	100.00
GOTERM_BP_ALL	GO:0044802~single-organism membrane organization	3	1.62	9.80E-01	ENSMODG00000004576, ENSMODG00000016096, ENSMODG00000004631	0.52	1.00E+00	100.00
GOTERM_BP_ALL	GO:0061024~membrane organization	3	1.62	9.89E-01	ENSMODG00000004576, ENSMODG00000016096, ENSMODG00000004631	0.47	1.00E+00	100.00
Annotation Cluster 41	DNA binding	Enrichment Score	0.03					
Category	Term	Count	%	PValue	Genes	Fold Enrichment	Benjamini	FDR

GOTERM_MF_ALL	GO:0001228~transcriptional activator activity, RNA polymerase II transcription regulatory region sequence-specific binding	3	1.62	7.71E-01	ENSMODG00000004298, ENSMODG00000009972, ENSMODG00000007389	1.07	9.99E-01	100.00
GOTERM_MF_ALL	GO:0000976~transcription regulatory region sequence-specific DNA binding	3	1.62	9.71E-01	ENSMODG00000004298, ENSMODG00000009972, ENSMODG00000007389	0.57	1.00E+00	100.00
GOTERM_MF_ALL	GO:1990837~sequence-specific double-stranded DNA binding	3	1.62	9.77E-01	ENSMODG00000004298, ENSMODG00000009972, ENSMODG00000007389	0.54	1.00E+00	100.00
GOTERM_MF_ALL	GO:0003690~double-stranded DNA binding	3	1.62	9.85E-01	ENSMODG00000004298, ENSMODG00000009972, ENSMODG00000007389	0.49	1.00E+00	100.00
GOTERM_MF_ALL	GO:0043565~sequence-specific DNA binding	3	1.62	9.98E-01	ENSMODG00000004298, ENSMODG00000009972, ENSMODG00000007389	0.35	1.00E+00	100.00
Annotation Cluster 42	Development	Enrichment Score	0.02					
Category	Term	Count	%	PValue	Genes	Fold Enrichment	Benjamini	FDR
GOTERM_BP_ALL	GO:0031175~neuron projection development	5	2.70	8.64E-01	ENSMODG00000010647, ENSMODG00000003907, ENSMODG00000004298, ENSMODG00000011909, ENSMODG00000015034	0.82	1.00E+00	100.00
GOTERM_BP_ALL	GO:0048666~neuron development	5	2.70	9.44E-01	ENSMODG00000010647, ENSMODG00000003907, ENSMODG00000004298, ENSMODG00000011909, ENSMODG00000015034	0.67	1.00E+00	100.00
GOTERM_BP_ALL	GO:0030182~neuron differentiation	5	2.70	9.88E-01	ENSMODG00000010647, ENSMODG00000003907, ENSMODG00000004298, ENSMODG00000011909, ENSMODG00000015034	0.52	1.00E+00	100.00
GOTERM_BP_ALL	GO:0048699~generation of neurons	5	2.70	9.94E-01	ENSMODG00000010647, ENSMODG00000003907, ENSMODG00000004298, ENSMODG00000011909, ENSMODG00000015034	0.47	1.00E+00	100.00
Annotation Cluster 43	Transcription	Enrichment Score	0.02					
Category	Term	Count	%	PValue	Genes	Fold Enrichment	Benjamini	FDR
GOTERM_BP_ALL	GO:0000122~negative regulation of transcription from RNA polymerase II promoter	4	2.16	7.93E-01	ENSMODG00000010647, ENSMODG00000006673, ENSMODG00000012475, ENSMODG00000004298	0.95	1.00E+00	100.00
GOTERM_BP_ALL	GO:0045892~negative regulation of transcription, DNA-templated	4	2.16	9.65E-01	ENSMODG00000010647, ENSMODG00000006673, ENSMODG00000012475, ENSMODG00000004298	0.60	1.00E+00	100.00
GOTERM_BP_ALL	GO:1903507~negative regulation of nucleic acid-templated transcription	4	2.16	9.66E-01	ENSMODG00000010647, ENSMODG00000006673, ENSMODG00000012475, ENSMODG00000004298	0.60	1.00E+00	100.00
GOTERM_BP_ALL	GO:1902679~negative regulation of RNA biosynthetic process	4	2.16	9.69E-01	ENSMODG00000010647, ENSMODG00000006673, ENSMODG00000012475, ENSMODG00000004298	0.59	1.00E+00	100.00
GOTERM_BP_ALL	GO:0051253~negative regulation of RNA metabolic process	4	2.16	9.76E-01	ENSMODG00000010647, ENSMODG00000006673, ENSMODG00000012475, ENSMODG00000004298	0.56	1.00E+00	100.00
GOTERM_BP_ALL	GO:2000113~negative regulation of cellular macromolecule biosynthetic process	4	2.16	9.89E-01	ENSMODG00000010647, ENSMODG00000006673, ENSMODG00000012475, ENSMODG00000004298	0.49	1.00E+00	100.00
GOTERM_BP_ALL	GO:0045934~negative regulation of nucleobase-containing compound metabolic process	4	2.16	9.90E-01	ENSMODG00000010647, ENSMODG00000006673, ENSMODG00000012475, ENSMODG00000004298	0.49	1.00E+00	100.00
GOTERM_BP_ALL	GO:0010558~negative regulation of macromolecule biosynthetic process	4	2.16	9.92E-01	ENSMODG00000010647, ENSMODG00000006673, ENSMODG00000012475, ENSMODG00000004298	0.47	1.00E+00	100.00
GOTERM_BP_ALL	GO:0031327~negative regulation of cellular biosynthetic process	4	2.16	9.94E-01	ENSMODG00000010647, ENSMODG00000006673, ENSMODG00000012475, ENSMODG00000004298	0.45	1.00E+00	100.00
GOTERM_BP_ALL	GO:0009890~negative regulation of biosynthetic process	4	2.16	9.95E-01	ENSMODG00000010647, ENSMODG00000006673, ENSMODG00000012475, ENSMODG00000004298	0.44	1.00E+00	100.00
GOTERM_BP_ALL	GO:0051172~negative regulation of nitrogen compound metabolic process	4	2.16	9.95E-01	ENSMODG00000010647, ENSMODG00000006673, ENSMODG00000012475, ENSMODG00000004298	0.44	1.00E+00	100.00
Annotation Cluster 44	Transcriptional regulation	Enrichment Score	0.02					
Category	Term	Count	%	PValue	Genes	Fold Enrichment	Benjamini	FDR
GOTERM_MF_ALL	GO:0000981~RNA polymerase II transcription factor activity, sequence-specific DNA binding	4	2.16	9.07E-01	ENSMODG00000016888, ENSMODG00000004298, ENSMODG00000009972, ENSMODG00000007389	0.74	1.00E+00	100.00
GOTERM_MF_ALL	GO:0044212~transcription regulatory region DNA binding	4	2.16	9.64E-01	ENSMODG00000016888, ENSMODG00000004298, ENSMODG00000009972, ENSMODG00000007389	0.60	1.00E+00	100.00
GOTERM_MF_ALL	GO:0001067~regulatory region nucleic acid binding	4	2.16	9.65E-01	ENSMODG00000016888, ENSMODG00000004298, ENSMODG00000009972, ENSMODG00000007389	0.60	1.00E+00	100.00
GOTERM_MF_ALL	GO:0000975~regulatory region DNA binding	4	2.16	9.65E-01	ENSMODG00000016888, ENSMODG00000004298, ENSMODG00000009972, ENSMODG00000007389	0.60	1.00E+00	100.00
GOTERM_MF_ALL	GO:0001071~nucleic acid binding transcription factor activity	4	2.16	9.95E-01	ENSMODG00000016888, ENSMODG00000004298, ENSMODG00000009972, ENSMODG00000007389	0.44	1.00E+00	100.00

GOTERM_MF_ALL	GO:0003700~transcription factor activity, sequence-specific DNA binding	4	2.16	9.95E-01	ENSMODG00000016888, ENSMODG00000004298, ENSMODG00000009972, ENSMODG00000007389	0.44	1.00E+00	100.00
Annotation Cluster 45	Transferase activity	Enrichment Score	0.01					
Category	Term	Count	%	PValue	Genes	Fold Enrichment	Benjamini	FDR
GOTERM_BP_ALL	GO:0051347~positive regulation of transferase activity	3	1.62	8.87E-01	ENSMODG00000012796, ENSMODG00000019764, ENSMODG00000010647	0.81	1.00E+00	100.00
GOTERM_BP_ALL	GO:0051338~regulation of transferase activity	3	1.62	9.79E-01	ENSMODG00000012796, ENSMODG00000019764, ENSMODG00000010647	0.53	1.00E+00	100.00
GOTERM_BP_ALL	GO:0031401~positive regulation of protein modification process	3	1.62	9.97E-01	ENSMODG00000012796, ENSMODG00000019764, ENSMODG00000010647	0.38	1.00E+00	100.00
GOTERM_BP_ALL	GO:0032270~positive regulation of cellular protein metabolic process	3	1.62	1.00E+00	ENSMODG00000012796, ENSMODG00000019764, ENSMODG00000010647	0.31	1.00E+00	100.00
GOTERM_BP_ALL	GO:0031399~regulation of protein modification process	3	1.62	1.00E+00	ENSMODG00000012796, ENSMODG00000019764, ENSMODG00000010647	0.25	1.00E+00	100.00
Annotation Cluster 46	Cell motility	Enrichment Score	0.01					
Category	Term	Count	%	PValue	Genes	Fold Enrichment	Benjamini	FDR
GOTERM_BP_ALL	GO:0016477~cell migration	6	3.24	9.51E-01	ENSMODG00000016955, ENSMODG00000002333, ENSMODG00000019764, ENSMODG00000010647, ENSMODG00000004298, ENSMODG00000011701	0.66	1.00E+00	100.00
GOTERM_BP_ALL	GO:0051674~localization of cell	6	3.24	9.77E-01	ENSMODG00000016955, ENSMODG00000002333, ENSMODG00000019764, ENSMODG00000010647, ENSMODG00000004298, ENSMODG00000011701	0.59	1.00E+00	100.00
GOTERM_BP_ALL	GO:0048870~cell motility	6	3.24	9.77E-01	ENSMODG00000016955, ENSMODG00000002333, ENSMODG00000019764, ENSMODG00000010647, ENSMODG00000004298, ENSMODG00000011701	0.59	1.00E+00	100.00
GOTERM_BP_ALL	GO:0040011~locomotion	6	3.24	9.92E-01	ENSMODG00000016955, ENSMODG00000002333, ENSMODG00000019764, ENSMODG00000010647, ENSMODG00000004298, ENSMODG00000011701	0.52	1.00E+00	100.00
Annotation Cluster 47	Cell adhesion	Enrichment Score	0.01					
Category	Term	Count	%	PValue	Genes	Fold Enrichment	Benjamini	FDR
GOTERM_BP_ALL	GO:0098609~cell-cell adhesion	4	2.16	9.48E-01	ENSMODG00000010471, ENSMODG00000000983, ENSMODG00000004631, ENSMODG00000015034	0.65	1.00E+00	100.00
GOTERM_BP_ALL	GO:0007155~cell adhesion	4	2.16	9.97E-01	ENSMODG00000010471, ENSMODG00000000983, ENSMODG00000004631, ENSMODG00000015034	0.42	1.00E+00	100.00
GOTERM_BP_ALL	GO:0022610~biological adhesion	4	2.16	9.97E-01	ENSMODG00000010471, ENSMODG00000000983, ENSMODG00000004631, ENSMODG00000015034	0.42	1.00E+00	100.00
Annotation Cluster 48	Transcription	Enrichment Score	0.01					
Category	Term	Count	%	PValue	Genes	Fold Enrichment	Benjamini	FDR
GOTERM_BP_ALL	GO:0006357~regulation of transcription from RNA polymerase II promoter	9	4.86	9.07E-01	ENSMODG00000015775, ENSMODG00000010647, ENSMODG00000006673, ENSMODG00000016888, ENSMODG00000012475, ENSMODG00000004298, ENSMODG00000023300, ENSMODG00000009972, ENSMODG00000016512	0.77	1.00E+00	100.00
GOTERM_BP_ALL	GO:0006355~regulation of transcription, DNA-templated	9	4.86	1.00E+00	ENSMODG00000015775, ENSMODG00000010647, ENSMODG00000006673, ENSMODG00000016888, ENSMODG00000012475, ENSMODG00000004298, ENSMODG00000023300, ENSMODG00000009972, ENSMODG00000016512	0.43	1.00E+00	100.00
GOTERM_BP_ALL	GO:1903506~regulation of nucleic acid-templated transcription	9	4.86	1.00E+00	ENSMODG00000015775, ENSMODG00000010647, ENSMODG00000006673, ENSMODG00000016888, ENSMODG00000012475, ENSMODG00000004298, ENSMODG00000023300, ENSMODG00000009972, ENSMODG00000016512	0.43	1.00E+00	100.00
GOTERM_BP_ALL	GO:2001141~regulation of RNA biosynthetic process	9	4.86	1.00E+00	ENSMODG00000015775, ENSMODG00000010647, ENSMODG00000006673, ENSMODG00000016888, ENSMODG00000012475, ENSMODG00000004298, ENSMODG00000023300, ENSMODG00000009972, ENSMODG00000016512	0.43	1.00E+00	100.00
GOTERM_BP_ALL	GO:0051252~regulation of RNA metabolic process	9	4.86	1.00E+00	ENSMODG00000015775, ENSMODG00000010647, ENSMODG00000006673, ENSMODG00000016888, ENSMODG00000012475, ENSMODG00000004298, ENSMODG00000023300, ENSMODG00000009972, ENSMODG00000016512	0.41	1.00E+00	100.00
GOTERM_BP_ALL	GO:2000112~regulation of cellular macromolecule biosynthetic process	9	4.86	1.00E+00	ENSMODG00000015775, ENSMODG00000010647, ENSMODG00000006673, ENSMODG00000016888, ENSMODG00000012475, ENSMODG00000004298, ENSMODG00000023300, ENSMODG00000009972, ENSMODG00000016512	0.38	1.00E+00	100.00
GOTERM_BP_ALL	GO:0010556~regulation of macromolecule biosynthetic process	9	4.86	1.00E+00	ENSMODG00000015775, ENSMODG00000010647, ENSMODG00000006673, ENSMODG00000016888, ENSMODG00000012475, ENSMODG00000004298, ENSMODG00000023300, ENSMODG00000009972, ENSMODG00000016512	0.37	1.00E+00	100.00

Annotation Cluster 49	Hemopoiesis	Enrichment Score	0.01					
Category	Term	Count	%	PValue	Genes	Fold Enrichment	Benjamini	FDR
GOTERM_BP_ALL	GO:0030097~hemopoiesis	3	1.62	9.84E-01	ENSMODG00000004631, ENSMODG00000010248, ENSMODG00000012130	0.50	1.00E+00	100.00
GOTERM_BP_ALL	GO:0048534~hematopoietic or lymphoid organ development	3	1.62	9.89E-01	ENSMODG00000004631, ENSMODG00000010248, ENSMODG00000012130	0.47	1.00E+00	100.00
GOTERM_BP_ALL	GO:0002520~immune system development	3	1.62	9.92E-01	ENSMODG00000004631, ENSMODG00000010248, ENSMODG00000012130	0.45	1.00E+00	100.00
Annotation Cluster 50	Protein organization	Enrichment Score	0.00					
Category	Term	Count	%	PValue	Genes	Fold Enrichment	Benjamini	FDR
GOTERM_BP_ALL	GO:0006461~protein complex assembly	4	2.16	9.92E-01	ENSMODG00000004392, ENSMODG00000025295, ENSMODG00000024056, ENSMODG00000020578	0.47	1.00E+00	100.00
GOTERM_BP_ALL	GO:0070271~protein complex biogenesis	4	2.16	9.92E-01	ENSMODG00000004392, ENSMODG00000025295, ENSMODG00000024056, ENSMODG00000020578	0.47	1.00E+00	100.00
GOTERM_BP_ALL	GO:0071822~protein complex subunit organization	4	2.16	9.96E-01	ENSMODG00000004392, ENSMODG00000025295, ENSMODG00000024056, ENSMODG00000020578	0.42	1.00E+00	100.00
GOTERM_BP_ALL	GO:0065003~macromolecular complex assembly	4	2.16	9.99E-01	ENSMODG00000004392, ENSMODG00000025295, ENSMODG00000024056, ENSMODG00000020578	0.37	1.00E+00	100.00
GOTERM_BP_ALL	GO:0043933~macromolecular complex subunit organization	4	2.16	1.00E+00	ENSMODG00000004392, ENSMODG00000025295, ENSMODG00000024056, ENSMODG00000020578	0.26	1.00E+00	100.00
Annotation Cluster 51	Transcription	Enrichment Score	0.00					
Category	Term	Count	%	PValue	Genes	Fold Enrichment	Benjamini	FDR
GOTERM_BP_ALL	GO:0006351~transcription, DNA-templated	10	5.41	9.97E-01	ENSMODG00000015775, ENSMODG00000010647, ENSMODG00000006673, ENSMODG00000016888, ENSMODG00000012475, ENSMODG00000020086, ENSMODG00000004298, ENSMODG00000023300, ENSMODG00000009972, ENSMODG00000016512	0.53	1.00E+00	100.00
GOTERM_BP_ALL	GO:0097659~nucleic acid-templated transcription	10	5.41	1.00E+00	ENSMODG00000015775, ENSMODG00000010647, ENSMODG00000006673, ENSMODG00000016888, ENSMODG00000012475, ENSMODG00000020086, ENSMODG00000004298, ENSMODG00000023300, ENSMODG00000009972, ENSMODG00000016512	0.45	1.00E+00	100.00
GOTERM_BP_ALL	GO:0032774~RNA biosynthetic process	10	5.41	1.00E+00	ENSMODG00000015775, ENSMODG00000010647, ENSMODG00000006673, ENSMODG00000016888, ENSMODG00000012475, ENSMODG00000020086, ENSMODG00000004298, ENSMODG00000023300, ENSMODG00000009972, ENSMODG00000016512	0.44	1.00E+00	100.00

**Supplementary Table 6. Cluster analysis of shared downregulated uterine genes in early pregnancy compared to non-reproductive state between *Monodelphis domestica* and *Sminthopsis crassicaudata*.**								
Annotation Cluster 1	Morphogenesis	Enrichment Score: 3.3016848448784697						
Category	Term	Count	%	PValue	Genes	Fold Enrichment	Benjamini	FDR
GOTERM_BP_ALL	GO:0072078~nephron tubule morphogenesis	7	3.431373	2.59E-04	ENSMODG00000000453, ENSMODG00000014680, ENSMODG00000029105, ENSMODG00000006420, ENSMODG00000020725, ENSMODG00000018877, ENSMODG00000015051	7.79	1.43E-02	0.47
GOTERM_BP_ALL	GO:0072088~nephron epithelium morphogenesis	7	3.431373	3.33E-04	ENSMODG00000000453, ENSMODG00000014680, ENSMODG00000029105, ENSMODG00000006420, ENSMODG00000020725, ENSMODG00000018877, ENSMODG00000015051	7.44	1.71E-02	0.60
GOTERM_BP_ALL	GO:0072028~nephron morphogenesis	7	3.431373	3.61E-04	ENSMODG00000000453, ENSMODG00000014680, ENSMODG00000029105, ENSMODG00000006420, ENSMODG00000020725, ENSMODG00000018877, ENSMODG00000015051	7.33	1.79E-02	0.65
GOTERM_BP_ALL	GO:0072080~nephron tubule development	7	3.431373	6.59E-04	ENSMODG00000000453, ENSMODG00000014680, ENSMODG00000029105, ENSMODG00000006420, ENSMODG00000020725, ENSMODG00000018877, ENSMODG00000015051	6.56	2.55E-02	1.18
GOTERM_BP_ALL	GO:0072009~nephron epithelium development	7	3.431373	0.001509	ENSMODG00000000453, ENSMODG00000014680, ENSMODG00000029105, ENSMODG00000006420, ENSMODG00000020725, ENSMODG00000018877, ENSMODG00000015051	5.60	4.42E-02	2.69
Annotation Cluster 2	Development	Enrichment Score: 2.993183831246702						
Category	Term	Count	%	PValue	Genes	Fold Enrichment	Benjamini	FDR
GOTERM_BP_ALL	GO:0001657~ureteric bud development	7	3.431373	9.23E-04	ENSMODG00000000453, ENSMODG00000014680, ENSMODG00000029105, ENSMODG00000014926, ENSMODG00000006420, ENSMODG00000020725, ENSMODG00000018877	6.15	3.23E-02	1.65
GOTERM_BP_ALL	GO:0072164~mesonephric tubule development	7	3.431373	9.85E-04	ENSMODG00000000453, ENSMODG00000014680, ENSMODG00000029105, ENSMODG00000014926, ENSMODG00000006420, ENSMODG00000020725, ENSMODG00000018877	6.08	3.36E-02	1.76
GOTERM_BP_ALL	GO:0072163~mesonephric epithelium development	7	3.431373	9.85E-04	ENSMODG00000000453, ENSMODG00000014680, ENSMODG00000029105, ENSMODG00000014926, ENSMODG00000006420, ENSMODG00000020725, ENSMODG00000018877	6.08	3.36E-02	1.76
GOTERM_BP_ALL	GO:0001823~mesonephros development	7	3.431373	0.001189	ENSMODG00000000453, ENSMODG00000014680, ENSMODG00000029105, ENSMODG00000014926, ENSMODG00000006420, ENSMODG00000020725, ENSMODG00000018877	5.86	3.75E-02	2.12
Annotation Cluster 3	Transport	Enrichment Score: 2.4439581755737048						
Category	Term	Count	%	PValue	Genes	Fold Enrichment	Benjamini	FDR
GOTERM_CC_ALL	GO:0034702~ion channel complex	9	4.411765	0.002439	ENSMODG00000003798, ENSMODG00000017022, ENSMODG00000004898, ENSMODG00000006537, ENSMODG00000000954, ENSMODG00000006708, ENSMODG00000006821, ENSMODG00000005162, ENSMODG00000014746	3.82	6.44E-02	3.25
GOTERM_CC_ALL	GO:1902495~transmembrane transporter complex	9	4.411765	0.00411	ENSMODG00000003798, ENSMODG00000017022, ENSMODG00000004898, ENSMODG00000006537, ENSMODG00000000954, ENSMODG00000006708, ENSMODG00000006821, ENSMODG00000005162, ENSMODG00000014746	3.51	9.17E-02	5.42
GOTERM_CC_ALL	GO:1990351~transporter complex	9	4.411765	0.004645	ENSMODG00000003798, ENSMODG00000017022, ENSMODG00000004898, ENSMODG00000006537, ENSMODG00000000954, ENSMODG00000006708, ENSMODG00000006821, ENSMODG00000005162, ENSMODG00000014746	3.44	9.08E-02	6.11
Annotation Cluster 4	Morphogenesis	Enrichment Score: 2.163018045722483						
Category	Term	Count	%	PValue	Genes	Fold Enrichment	Benjamini	FDR
GOTERM_BP_ALL	GO:0007368~determination of left/right symmetry	6	2.941176	0.005733	ENSMODG00000014680, ENSMODG00000012059, ENSMODG00000016160, ENSMODG00000003548, ENSMODG00000020725, ENSMODG00000013125	5.21	1.14E-01	9.85
GOTERM_BP_ALL	GO:0009855~determination of bilateral symmetry	6	2.941176	0.007345	ENSMODG00000014680, ENSMODG00000012059, ENSMODG00000016160, ENSMODG00000003548, ENSMODG00000020725, ENSMODG00000013125	4.91	1.40E-01	12.45
GOTERM_BP_ALL	GO:0009799~specification of symmetry	6	2.941176	0.007702	ENSMODG00000014680, ENSMODG00000012059, ENSMODG00000016160, ENSMODG00000003548, ENSMODG00000020725, ENSMODG00000013125	4.85	1.44E-01	13.02
Annotation Cluster 5	Ion binding	Enrichment Score: 2.124210338253309						
Category	Term	Count	%	PValue	Genes	Fold Enrichment	Benjamini	FDR

GOTERM_MF_ALL	GO:0046872~metal ion binding	42	20.58824	0.005325	ENSMODG00000003807, ENSMODG00000016954, ENSMODG00000016375, ENSMODG00000025001, ENSMODG00000010122, ENSMODG00000018141, ENSMODG00000003112, ENSMODG00000010173, ENSMODG00000016774, ENSMODG00000006996, ENSMODG00000011719, ENSMODG00000002061, ENSMODG00000020725, ENSMODG00000018775, ENSMODG00000005807, ENSMODG00000006821, ENSMODG00000016176, ENSMODG00000018947, ENSMODG00000013409, ENSMODG00000015435, ENSMODG00000016387, ENSMODG00000000911, ENSMODG00000006068, ENSMODG00000015171, ENSMODG00000003959, ENSMODG00000007847, ENSMODG00000020329, ENSMODG00000017473, ENSMODG00000005011, ENSMODG00000018877, ENSMODG00000012390, ENSMODG00000024585, ENSMODG00000020425, ENSMODG00000021408, ENSMODG00000011014, ENSMODG00000014926, ENSMODG00000011920, ENSMODG00000004834, ENSMODG00000003977, ENSMODG00000021299, ENSMODG00000014540, ENSMODG00000015403	1.48	3.56E-01	7.21
GOTERM_MF_ALL	GO:0043169~cation binding	42	20.58824	0.006195	ENSMODG00000003807, ENSMODG00000016954, ENSMODG00000016375, ENSMODG00000025001, ENSMODG00000010122, ENSMODG00000018141, ENSMODG00000003112, ENSMODG00000010173, ENSMODG00000016774, ENSMODG00000006996, ENSMODG00000011719, ENSMODG00000002061, ENSMODG00000020725, ENSMODG00000018775, ENSMODG00000005807, ENSMODG00000006821, ENSMODG00000016176, ENSMODG00000018947, ENSMODG00000013409, ENSMODG00000015435, ENSMODG00000016387, ENSMODG00000000911, ENSMODG00000006068, ENSMODG00000015171, ENSMODG00000003959, ENSMODG00000007847, ENSMODG00000020329, ENSMODG00000017473, ENSMODG00000005011, ENSMODG00000018877, ENSMODG00000012390, ENSMODG00000024585, ENSMODG00000020425, ENSMODG00000021408, ENSMODG00000011014, ENSMODG00000014926, ENSMODG00000011920, ENSMODG00000004834, ENSMODG00000003977, ENSMODG00000021299, ENSMODG00000014540, ENSMODG00000015403	1.47	3.47E-01	8.34
GOTERM_MF_ALL	GO:0043167~ion binding	42	20.58824	0.012851	ENSMODG00000003807, ENSMODG00000016954, ENSMODG00000016375, ENSMODG00000025001, ENSMODG00000010122, ENSMODG00000018141, ENSMODG00000003112, ENSMODG00000010173, ENSMODG00000016774, ENSMODG00000006996, ENSMODG00000011719, ENSMODG00000002061, ENSMODG00000020725, ENSMODG00000018775, ENSMODG00000005807, ENSMODG00000006821, ENSMODG00000016176, ENSMODG00000018947, ENSMODG00000013409, ENSMODG00000015435, ENSMODG00000016387, ENSMODG00000000911, ENSMODG00000006068, ENSMODG00000015171, ENSMODG00000003959, ENSMODG00000007847, ENSMODG00000020329, ENSMODG00000017473, ENSMODG00000005011, ENSMODG00000018877, ENSMODG00000012390, ENSMODG00000024585, ENSMODG00000020425, ENSMODG00000021408, ENSMODG00000011014, ENSMODG00000014926, ENSMODG00000011920, ENSMODG00000004834, ENSMODG00000003977, ENSMODG00000021299, ENSMODG00000014540, ENSMODG00000015403	1.41	4.47E-01	16.57
Annotation Cluster 6	Cellular motility	Enrichment Score: 2.1131397396965856						
Category	Term	Count	%	PValue	Genes	Fold Enrichment	Benjamini	FDR
GOTERM_BP_ALL	GO:2000145~regulation of cell motility	15	7.352941	0.005453	ENSMODG00000000646, ENSMODG00000029105, ENSMODG00000018991, ENSMODG00000010848, ENSMODG00000009971, ENSMODG00000010173, ENSMODG00000019403, ENSMODG00000001183, ENSMODG00000004834, ENSMODG00000019535, ENSMODG00000018325, ENSMODG00000013389, ENSMODG00000003977, ENSMODG00000016857, ENSMODG00000003513	2.30	1.11E-01	9.39
GOTERM_BP_ALL	GO:0040012~regulation of locomotion	15	7.352941	0.007812	ENSMODG00000000646, ENSMODG00000029105, ENSMODG00000018991, ENSMODG00000010848, ENSMODG00000009971, ENSMODG00000010173, ENSMODG00000019403, ENSMODG00000001183, ENSMODG00000004834, ENSMODG00000019535, ENSMODG00000018325, ENSMODG00000013389, ENSMODG00000003977, ENSMODG00000016857, ENSMODG00000003513	2.21	1.43E-01	13.19
GOTERM_BP_ALL	GO:0051270~regulation of cellular component movement	15	7.352941	0.010745	ENSMODG00000000646, ENSMODG00000029105, ENSMODG00000018991, ENSMODG00000010848, ENSMODG00000009971, ENSMODG00000010173, ENSMODG00000019403, ENSMODG00000001183, ENSMODG00000004834, ENSMODG00000019535, ENSMODG00000018325, ENSMODG00000013389, ENSMODG00000003977, ENSMODG00000016857, ENSMODG00000003513	2.12	1.74E-01	17.71
Annotation Cluster 7	Cellular motility	Enrichment Score: 1.9884024117721995						
Category	Term	Count	%	PValue	Genes	Fold Enrichment	Benjamini	FDR

GOTERM_BP_ALL	GO:0030335~positive regulation of cell migration	10	4.901961	0.008845	ENSMODG00000019403, ENSMODG00000000646, ENSMODG00000029105, ENSMODG00000004834, ENSMODG00000019535, ENSMODG00000018325, ENSMODG00000010848, ENSMODG00000003977, ENSMODG00000009971, ENSMODG00000010173	2.82	1.57E-01	14.81
GOTERM_BP_ALL	GO:2000147~positive regulation of cell motility	10	4.901961	0.009748	ENSMODG00000019403, ENSMODG00000000646, ENSMODG00000029105, ENSMODG00000004834, ENSMODG00000019535, ENSMODG00000018325, ENSMODG00000010848, ENSMODG00000003977, ENSMODG00000009971, ENSMODG00000010173	2.78	1.64E-01	16.20
GOTERM_BP_ALL	GO:0051272~positive regulation of cellular component movement	10	4.901961	0.010973	ENSMODG00000019403, ENSMODG00000000646, ENSMODG00000029105, ENSMODG00000004834, ENSMODG00000019535, ENSMODG00000018325, ENSMODG00000010848, ENSMODG00000003977, ENSMODG00000009971, ENSMODG00000010173	2.73	1.76E-01	18.05
GOTERM_BP_ALL	GO:0040017~positive regulation of locomotion	10	4.901961	0.011761	ENSMODG00000019403, ENSMODG00000000646, ENSMODG00000029105, ENSMODG00000004834, ENSMODG00000019535, ENSMODG00000018325, ENSMODG00000010848, ENSMODG00000003977, ENSMODG00000009971, ENSMODG00000010173	2.70	1.85E-01	19.22
Annotation Cluster 8	Ion transport	Enrichment Score: 1.722902063049012						
Category	Term	Count	%	PValue	Genes	Fold Enrichment	Benjamini	FDR
GOTERM_MF_ALL	GO:0005216~ion channel activity	10	4.901961	0.014833	ENSMODG00000003798, ENSMODG00000017022, ENSMODG00000006537, ENSMODG00000020725, ENSMODG00000000954, ENSMODG00000015701, ENSMODG00000006708, ENSMODG00000004966, ENSMODG00000005162, ENSMODG00000014746	2.59	4.60E-01	18.89
GOTERM_MF_ALL	GO:0022838~substrate-specific channel activity	10	4.901961	0.017126	ENSMODG00000003798, ENSMODG00000017022, ENSMODG00000006537, ENSMODG00000020725, ENSMODG00000000954, ENSMODG00000015701, ENSMODG00000006708, ENSMODG00000004966, ENSMODG00000005162, ENSMODG00000014746	2.52	4.76E-01	21.49
GOTERM_MF_ALL	GO:0022803~passive transmembrane transporter activity	10	4.901961	0.022478	ENSMODG00000003798, ENSMODG00000017022, ENSMODG00000006537, ENSMODG00000020725, ENSMODG00000000954, ENSMODG00000015701, ENSMODG00000006708, ENSMODG00000004966, ENSMODG00000005162, ENSMODG00000014746	2.41	4.88E-01	27.27
GOTERM_MF_ALL	GO:0015267~channel activity	10	4.901961	0.022478	ENSMODG00000003798, ENSMODG00000017022, ENSMODG00000006537, ENSMODG00000020725, ENSMODG00000000954, ENSMODG00000015701, ENSMODG00000006708, ENSMODG00000004966, ENSMODG00000005162, ENSMODG00000014746	2.41	4.88E-01	27.27
Annotation Cluster 9	Protein localization	Enrichment Score: 1.6776127351729047						
Category	Term	Count	%	PValue	Genes	Fold Enrichment	Benjamini	FDR
GOTERM_BP_ALL	GO:1904590~negative regulation of protein import	4	1.960784	0.016902	ENSMODG00000001074, ENSMODG00000004260, ENSMODG00000020725, ENSMODG00000010173	7.30	2.29E-01	26.48
GOTERM_BP_ALL	GO:0042308~negative regulation of protein import into nucleus	4	1.960784	0.016902	ENSMODG00000001074, ENSMODG00000004260, ENSMODG00000020725, ENSMODG00000010173	7.30	2.29E-01	26.48
GOTERM_BP_ALL	GO:1900181~negative regulation of protein localization to nucleus	4	1.960784	0.032455	ENSMODG00000001074, ENSMODG00000004260, ENSMODG00000020725, ENSMODG00000010173	5.69	3.29E-01	44.86
Annotation Cluster 10	Transport	Enrichment Score: 1.6717563505036188						
Category	Term	Count	%	PValue	Genes	Fold Enrichment	Benjamini	FDR
GOTERM_BP_ALL	GO:0046823~negative regulation of nucleocytoplasmic transport	5	2.45098	0.003786	ENSMODG00000011338, ENSMODG00000001074, ENSMODG00000004260, ENSMODG00000020725, ENSMODG00000010173	7.74	8.71E-02	6.61
GOTERM_BP_ALL	GO:0090317~negative regulation of intracellular protein transport	5	2.45098	0.007653	ENSMODG00000011338, ENSMODG00000001074, ENSMODG00000004260, ENSMODG00000020725, ENSMODG00000010173	6.36	1.44E-01	12.94
GOTERM_BP_ALL	GO:0032387~negative regulation of intracellular transport	5	2.45098	0.014926	ENSMODG00000011338, ENSMODG00000001074, ENSMODG00000004260, ENSMODG00000020725, ENSMODG00000010173	5.23	2.11E-01	23.76
GOTERM_BP_ALL	GO:0051224~negative regulation of protein transport	5	2.45098	0.091263	ENSMODG00000011338, ENSMODG00000001074, ENSMODG00000004260, ENSMODG00000020725, ENSMODG00000010173	2.92	5.50E-01	82.21
GOTERM_BP_ALL	GO:1904950~negative regulation of establishment of protein localization	5	2.45098	0.110934	ENSMODG00000011338, ENSMODG00000001074, ENSMODG00000004260, ENSMODG00000020725, ENSMODG00000010173	2.72	5.95E-01	88.01
Annotation Cluster 11	DNA binding	Enrichment Score: 1.647024057722411						
Category	Term	Count	%	PValue	Genes	Fold Enrichment	Benjamini	FDR

GOTERM_MF_ALL	GO:0044212~transcription regulatory region DNA binding	14	6.862745	0.021993	ENSMODG00000016387, ENSMODG00000006068, ENSMODG00000016375, ENSMODG00000012059, ENSMODG00000015190, ENSMODG00000007847, ENSMODG00000016774, ENSMODG00000002992, ENSMODG00000011338, ENSMODG00000019416, ENSMODG00000014926, ENSMODG00000003548, ENSMODG00000018597, ENSMODG00000016176	1.99	5.34E-01	26.77
GOTERM_MF_ALL	GO:0000975~regulatory region DNA binding	14	6.862745	0.02282	ENSMODG00000016387, ENSMODG00000006068, ENSMODG00000016375, ENSMODG00000012059, ENSMODG00000015190, ENSMODG00000007847, ENSMODG00000016774, ENSMODG00000002992, ENSMODG00000011338, ENSMODG00000019416, ENSMODG00000014926, ENSMODG00000003548, ENSMODG00000018597, ENSMODG00000016176	1.98	4.48E-01	27.63
GOTERM_MF_ALL	GO:0001067~regulatory region nucleic acid binding	14	6.862745	0.02282	ENSMODG00000016387, ENSMODG00000006068, ENSMODG00000016375, ENSMODG00000012059, ENSMODG00000015190, ENSMODG00000007847, ENSMODG00000016774, ENSMODG00000002992, ENSMODG00000011338, ENSMODG00000019416, ENSMODG00000014926, ENSMODG00000003548, ENSMODG00000018597, ENSMODG00000016176	1.98	4.48E-01	27.63
Annotation Cluster 12	Morphogenesis	Enrichment Score: 1.6212777930106994						
Category	Term	Count	%	PValue	Genes	Fold Enrichment	Benjamini	FDR
GOTERM_BP_ALL	GO:0001947~heart looping	4	1.960784	0.018089	ENSMODG00000014680, ENSMODG00000012059, ENSMODG00000003548, ENSMODG00000020725	7.12	2.38E-01	28.06
GOTERM_BP_ALL	GO:0061371~determination of heart left/right asymmetry	4	1.960784	0.021922	ENSMODG00000014680, ENSMODG00000012059, ENSMODG00000003548, ENSMODG00000020725	6.62	2.69E-01	32.96
GOTERM_BP_ALL	GO:0003143~embryonic heart tube morphogenesis	4	1.960784	0.021922	ENSMODG00000014680, ENSMODG00000012059, ENSMODG00000003548, ENSMODG00000020725	6.62	2.69E-01	32.96
GOTERM_BP_ALL	GO:0035050~embryonic heart tube development	4	1.960784	0.037644	ENSMODG00000014680, ENSMODG00000012059, ENSMODG00000003548, ENSMODG00000020725	5.37	3.64E-01	49.96
Annotation Cluster 13	Protein transport	Enrichment Score: 1.6123936489745756						
Category	Term	Count	%	PValue	Genes	Fold Enrichment	Benjamini	FDR
GOTERM_BP_ALL	GO:0042306~regulation of protein import into nucleus	7	3.431373	0.004782	ENSMODG00000001183, ENSMODG00000014680, ENSMODG00000001074, ENSMODG00000004834, ENSMODG00000004260, ENSMODG00000020725, ENSMODG00000010173	4.45	1.03E-01	8.29
GOTERM_BP_ALL	GO:1904589~regulation of protein import	7	3.431373	0.005211	ENSMODG00000001183, ENSMODG00000014680, ENSMODG00000001074, ENSMODG00000004834, ENSMODG00000004260, ENSMODG00000020725, ENSMODG00000010173	4.37	1.09E-01	9.00
GOTERM_BP_ALL	GO:1902593~single-organism nuclear import	7	3.431373	0.038659	ENSMODG00000001183, ENSMODG00000014680, ENSMODG00000001074, ENSMODG00000004834, ENSMODG00000004260, ENSMODG00000020725, ENSMODG00000010173	2.80	3.69E-01	50.90
GOTERM_BP_ALL	GO:0006606~protein import into nucleus	7	3.431373	0.038659	ENSMODG00000001183, ENSMODG00000014680, ENSMODG00000001074, ENSMODG00000004834, ENSMODG00000004260, ENSMODG00000020725, ENSMODG00000010173	2.80	3.69E-01	50.90
GOTERM_BP_ALL	GO:0044744~protein targeting to nucleus	7	3.431373	0.038659	ENSMODG00000001183, ENSMODG00000014680, ENSMODG00000001074, ENSMODG00000004834, ENSMODG00000004260, ENSMODG00000020725, ENSMODG00000010173	2.80	3.69E-01	50.90
GOTERM_BP_ALL	GO:0051170~nuclear import	7	3.431373	0.039505	ENSMODG00000001183, ENSMODG00000014680, ENSMODG00000001074, ENSMODG00000004834, ENSMODG00000004260, ENSMODG00000020725, ENSMODG00000010173	2.78	3.75E-01	51.67
GOTERM_BP_ALL	GO:0017038~protein import	7	3.431373	0.090835	ENSMODG00000001183, ENSMODG00000014680, ENSMODG00000001074, ENSMODG00000004834, ENSMODG00000004260, ENSMODG00000020725, ENSMODG00000010173	2.24	5.49E-01	82.06
Annotation Cluster 14	Nuclear transport	Enrichment Score: 1.5511008010914755						
Category	Term	Count	%	PValue	Genes	Fold Enrichment	Benjamini	FDR
GOTERM_BP_ALL	GO:0046822~regulation of nucleocytoplasmic transport	8	3.921569	0.003596	ENSMODG00000011338, ENSMODG00000001183, ENSMODG00000014680, ENSMODG00000001074, ENSMODG00000004834, ENSMODG00000004260, ENSMODG00000020725, ENSMODG00000010173	4.04	8.42E-02	6.29
GOTERM_BP_ALL	GO:0006913~nucleocytoplasmic transport	8	3.921569	0.076799	ENSMODG00000011338, ENSMODG00000001183, ENSMODG00000014680, ENSMODG00000001074, ENSMODG00000004834, ENSMODG00000004260, ENSMODG00000020725, ENSMODG00000010173	2.16	5.16E-01	76.35
GOTERM_BP_ALL	GO:0051169~nuclear transport	8	3.921569	0.080446	ENSMODG00000011338, ENSMODG00000001183, ENSMODG00000014680, ENSMODG00000001074, ENSMODG00000004834, ENSMODG00000004260, ENSMODG00000020725, ENSMODG00000010173	2.13	5.28E-01	77.98

Annotation Cluster 15	Cell motility	Enrichment Score: 1.533949607054988						
Category	Term	Count	%	PValue	Genes	Fold Enrichment	Benjamini	FDR
GOTERM_BP_ALL	GO:0010631~epithelial cell migration	7	3.431373	0.027164	ENSMODG00000029105, ENSMODG00000019535, ENSMODG00000010848, ENSMODG00000003977, ENSMODG00000016857, ENSMODG00000003112, ENSMODG00000004739	3.04	3.02E-01	39.16
GOTERM_BP_ALL	GO:0090132~epithelium migration	7	3.431373	0.028861	ENSMODG00000029105, ENSMODG00000019535, ENSMODG00000010848, ENSMODG00000003977, ENSMODG00000016857, ENSMODG00000003112, ENSMODG00000004739	3.00	3.13E-01	41.04
GOTERM_BP_ALL	GO:0090130~tissue migration	7	3.431373	0.031904	ENSMODG00000029105, ENSMODG00000019535, ENSMODG00000010848, ENSMODG00000003977, ENSMODG00000016857, ENSMODG00000003112, ENSMODG00000004739	2.93	3.26E-01	44.29
Annotation Cluster 16	Ion transport	Enrichment Score: 1.5265326357082847						
Category	Term	Count	%	PValue	Genes	Fold Enrichment	Benjamini	FDR
GOTERM_BP_ALL	GO:1901379~regulation of potassium ion transmembrane transport	4	1.960784	0.011641	ENSMODG00000003798, ENSMODG00000004898, ENSMODG00000015701, ENSMODG00000006821	8.37	1.84E-01	19.04
GOTERM_BP_ALL	GO:0043266~regulation of potassium ion transport	4	1.960784	0.020599	ENSMODG00000003798, ENSMODG00000004898, ENSMODG00000015701, ENSMODG00000006821	6.78	2.62E-01	31.31
GOTERM_BP_ALL	GO:0006813~potassium ion transport	4	1.960784	0.109792	ENSMODG00000003798, ENSMODG00000004898, ENSMODG00000015701, ENSMODG00000006821	3.43	5.92E-01	87.73
Annotation Cluster 17	Phosphorylation	Enrichment Score: 1.4789320824418497						
Category	Term	Count	%	PValue	Genes	Fold Enrichment	Benjamini	FDR
GOTERM_BP_ALL	GO:0001934~positive regulation of protein phosphorylation	15	7.352941	0.017317	ENSMODG00000002143, ENSMODG00000012068, ENSMODG00000007648, ENSMODG00000029105, ENSMODG00000018991, ENSMODG00000003112, ENSMODG00000019579, ENSMODG00000019403, ENSMODG00000001183, ENSMODG00000009785, ENSMODG00000020725, ENSMODG00000004260, ENSMODG00000003977, ENSMODG00000018947, ENSMODG00000019334	2.00	2.32E-01	27.03
GOTERM_BP_ALL	GO:0042327~positive regulation of phosphorylation	15	7.352941	0.024241	ENSMODG00000002143, ENSMODG00000012068, ENSMODG00000007648, ENSMODG00000029105, ENSMODG00000018991, ENSMODG00000003112, ENSMODG00000019579, ENSMODG00000019403, ENSMODG00000001183, ENSMODG00000009785, ENSMODG00000020725, ENSMODG00000004260, ENSMODG00000003977, ENSMODG00000018947, ENSMODG00000019334	1.91	2.85E-01	35.77
GOTERM_BP_ALL	GO:0045937~positive regulation of phosphate metabolic process	15	7.352941	0.05378	ENSMODG00000002143, ENSMODG00000012068, ENSMODG00000007648, ENSMODG00000029105, ENSMODG00000018991, ENSMODG00000003112, ENSMODG00000019579, ENSMODG00000019403, ENSMODG00000001183, ENSMODG00000009785, ENSMODG00000020725, ENSMODG00000004260, ENSMODG00000003977, ENSMODG00000018947, ENSMODG00000019334	1.71	4.41E-01	63.11
GOTERM_BP_ALL	GO:0010562~positive regulation of phosphorus metabolic process	15	7.352941	0.05378	ENSMODG00000002143, ENSMODG00000012068, ENSMODG00000007648, ENSMODG00000029105, ENSMODG00000018991, ENSMODG00000003112, ENSMODG00000019579, ENSMODG00000019403, ENSMODG00000001183, ENSMODG00000009785, ENSMODG00000020725, ENSMODG00000004260, ENSMODG00000003977, ENSMODG00000018947, ENSMODG00000019334	1.71	4.41E-01	63.11
Annotation Cluster 18	Gamete differentiation	Enrichment Score: 1.4707521462087296						
Category	Term	Count	%	PValue	Genes	Fold Enrichment	Benjamini	FDR
GOTERM_BP_ALL	GO:0009994~oocyte differentiation	4	1.960784	0.012604	ENSMODG00000007007, ENSMODG00000020406, ENSMODG00000018597, ENSMODG00000018947	8.14	1.93E-01	20.45
GOTERM_BP_ALL	GO:0048477~oogenesis	4	1.960784	0.041325	ENSMODG00000007007, ENSMODG00000020406, ENSMODG00000018597, ENSMODG00000018947	5.18	3.85E-01	53.30
GOTERM_BP_ALL	GO:0007292~female gamete generation	4	1.960784	0.074307	ENSMODG00000007007, ENSMODG00000020406, ENSMODG00000018597, ENSMODG00000018947	4.07	5.07E-01	75.17
Annotation Cluster 19	Development	Enrichment Score: 1.3084071399341777						
Category	Term	Count	%	PValue	Genes	Fold Enrichment	Benjamini	FDR
GOTERM_BP_ALL	GO:0042633~hair cycle	5	2.45098	0.013494	ENSMODG00000014680, ENSMODG00000002526, ENSMODG00000015361, ENSMODG00000021440, ENSMODG00000020406	5.39	1.99E-01	21.74
GOTERM_BP_ALL	GO:0042303~molting cycle	5	2.45098	0.013494	ENSMODG00000014680, ENSMODG00000002526, ENSMODG00000015361, ENSMODG00000021440, ENSMODG00000020406	5.39	1.99E-01	21.74

GOTERM_BP_ALL	GO:0043588~skin development	5	2.45098	0.14998	ENSMODG00000014680, ENSMODG00000002526, ENSMODG00000015361, ENSMODG00000021440, ENSMODG00000020406	2.42	6.69E-01	94.67
GOTERM_BP_ALL	GO:0008544~epidermis development	5	2.45098	0.213838	ENSMODG00000014680, ENSMODG00000002526, ENSMODG00000015361, ENSMODG00000021440, ENSMODG00000020406	2.09	7.69E-01	98.70
Annotation Cluster 20	Morphogenesis	Enrichment Score: 1.3046669436398965						
Category	Term	Count	%	PValue	Genes	Fold Enrichment	Benjamini	FDR
GOTERM_BP_ALL	GO:0072273~metanephric nephron morphogenesis	3	1.470588	0.031218	ENSMODG00000014680, ENSMODG00000006420, ENSMODG00000020725	10.68	3.24E-01	43.57
GOTERM_BP_ALL	GO:0003338~metanephros morphogenesis	3	1.470588	0.050586	ENSMODG00000014680, ENSMODG00000006420, ENSMODG00000020725	8.21	4.28E-01	60.80
GOTERM_BP_ALL	GO:0072210~metanephric nephron development	3	1.470588	0.07719	ENSMODG00000014680, ENSMODG00000006420, ENSMODG00000020725	6.47	5.16E-01	76.53
Annotation Cluster 21	Development	Enrichment Score: 1.2990804436603658						
Category	Term	Count	%	PValue	Genes	Fold Enrichment	Benjamini	FDR
GOTERM_BP_ALL	GO:0001942~hair follicle development	4	1.960784	0.049208	ENSMODG00000014680, ENSMODG00000015361, ENSMODG00000021440, ENSMODG00000020406	4.83	4.23E-01	59.76
GOTERM_BP_ALL	GO:0022404~molting cycle process	4	1.960784	0.049208	ENSMODG00000014680, ENSMODG00000015361, ENSMODG00000021440, ENSMODG00000020406	4.83	4.23E-01	59.76
GOTERM_BP_ALL	GO:0022405~hair cycle process	4	1.960784	0.049208	ENSMODG00000014680, ENSMODG00000015361, ENSMODG00000021440, ENSMODG00000020406	4.83	4.23E-01	59.76
GOTERM_BP_ALL	GO:0098773~skin epidermis development	4	1.960784	0.053405	ENSMODG00000014680, ENSMODG00000015361, ENSMODG00000021440, ENSMODG00000020406	4.67	4.40E-01	62.85
Annotation Cluster 22	Cadherin function	Enrichment Score: 1.2811412294692217						
Category	Term	Count	%	PValue	Genes	Fold Enrichment	Benjamini	FDR
INTERPRO	IPR020894:Cadherin conserved site	4	1.960784	0.034809	ENSMODG00000021408, ENSMODG00000006996, ENSMODG00000017473, ENSMODG00000018877	5.56	6.18E-01	39.35
INTERPRO	IPR002126:Cadherin	4	1.960784	0.04611	ENSMODG00000021408, ENSMODG00000006996, ENSMODG00000017473, ENSMODG00000018877	4.97	7.00E-01	48.64
INTERPRO	IPR015919:Cadherin-like	4	1.960784	0.049634	ENSMODG00000021408, ENSMODG00000006996, ENSMODG00000017473, ENSMODG00000018877	4.82	7.07E-01	51.25
SMART	SM00112:CA	4	1.960784	0.094224	ENSMODG00000021408, ENSMODG00000006996, ENSMODG00000017473, ENSMODG00000018877	3.66	8.41E-01	68.41
Annotation Cluster 23	Morphogenesis	Enrichment Score: 1.2589275960726924						
Category	Term	Count	%	PValue	Genes	Fold Enrichment	Benjamini	FDR
GOTERM_BP_ALL	GO:0001841~neural tube formation	5	2.45098	0.02872	ENSMODG00000011338, ENSMODG00000016387, ENSMODG00000001183, ENSMODG00000003112, ENSMODG00000018983	4.29	3.14E-01	40.89
GOTERM_BP_ALL	GO:0072175~epithelial tube formation	5	2.45098	0.059279	ENSMODG00000011338, ENSMODG00000016387, ENSMODG00000001183, ENSMODG00000003112, ENSMODG00000018983	3.39	4.61E-01	66.80
GOTERM_BP_ALL	GO:0001838~embryonic epithelial tube formation	5	2.45098	0.059279	ENSMODG00000011338, ENSMODG00000016387, ENSMODG00000001183, ENSMODG00000003112, ENSMODG00000018983	3.39	4.61E-01	66.80
GOTERM_BP_ALL	GO:0016331~morphogenesis of embryonic epithelium	5	2.45098	0.091263	ENSMODG00000011338, ENSMODG00000016387, ENSMODG00000001183, ENSMODG00000003112, ENSMODG00000018983	2.92	5.50E-01	82.21
Annotation Cluster 24	Kinase activity	Enrichment Score: 1.116731951471132						
Category	Term	Count	%	PValue	Genes	Fold Enrichment	Benjamini	FDR
GOTERM_BP_ALL	GO:0071902~positive regulation of protein serine/threonine kinase activity	7	3.431373	0.025331	ENSMODG00000019579, ENSMODG00000019403, ENSMODG00000029105, ENSMODG00000018991, ENSMODG00000020725, ENSMODG00000003112, ENSMODG00000018947	3.10	2.91E-01	37.05
GOTERM_BP_ALL	GO:0045860~positive regulation of protein kinase activity	7	3.431373	0.111263	ENSMODG00000019579, ENSMODG00000019403, ENSMODG00000029105, ENSMODG00000018991, ENSMODG00000020725, ENSMODG00000003112, ENSMODG00000018947	2.12	5.94E-01	88.09
GOTERM_BP_ALL	GO:0033674~positive regulation of kinase activity	7	3.431373	0.158418	ENSMODG00000019579, ENSMODG00000019403, ENSMODG00000029105, ENSMODG00000018991, ENSMODG00000020725, ENSMODG00000003112, ENSMODG00000018947	1.91	6.84E-01	95.55
Annotation Cluster 25	Hypoxic response	Enrichment Score: 1.1124439569297477						
Category	Term	Count	%	PValue	Genes	Fold Enrichment	Benjamini	FDR

GOTERM_BP_ALL	GO:0001666~response to hypoxia	5	2.45098	0.073453	ENSMODG00000019416, ENSMODG00000011920, ENSMODG00000019535, ENSMODG00000000658, ENSMODG00000000954	3.15	5.04E-01	74.75
GOTERM_BP_ALL	GO:0036293~response to decreased oxygen levels	5	2.45098	0.075336	ENSMODG00000019416, ENSMODG00000011920, ENSMODG00000019535, ENSMODG00000000658, ENSMODG00000000954	3.12	5.11E-01	75.66
GOTERM_BP_ALL	GO:0070482~response to oxygen levels	5	2.45098	0.08311	ENSMODG00000019416, ENSMODG00000011920, ENSMODG00000019535, ENSMODG00000000658, ENSMODG00000000954	3.02	5.33E-01	79.10
Annotation Cluster 26	Morphogenesis	Enrichment Score: 1.0913249698003271						
Category	Term	Count	%	PValue	Genes	Fold Enrichment	Benjamini	FDR
GOTERM_BP_ALL	GO:0001843~neural tube closure	4	1.960784	0.076824	ENSMODG00000016387, ENSMODG00000001183, ENSMODG00000003112, ENSMODG00000018983	4.01	5.15E-01	76.36
GOTERM_BP_ALL	GO:0060606~tube closure	4	1.960784	0.079379	ENSMODG00000016387, ENSMODG00000001183, ENSMODG00000003112, ENSMODG00000018983	3.95	5.24E-01	77.51
GOTERM_BP_ALL	GO:0014020~primary neural tube formation	4	1.960784	0.087261	ENSMODG00000016387, ENSMODG00000001183, ENSMODG00000003112, ENSMODG00000018983	3.80	5.40E-01	80.74
Annotation Cluster 27	Transport	Enrichment Score: 1.05304709537729						
Category	Term	Count	%	PValue	Genes	Fold Enrichment	Benjamini	FDR
GOTERM_BP_ALL	GO:0035411~catenin import into nucleus	3	1.470588	0.022962	ENSMODG00000001183, ENSMODG00000004260, ENSMODG00000010173	12.56	2.78E-01	34.24
GOTERM_BP_ALL	GO:0035412~regulation of catenin import into nucleus	3	1.470588	0.037275	ENSMODG00000001183, ENSMODG00000004260, ENSMODG00000010173	9.71	3.62E-01	49.61
GOTERM_BP_ALL	GO:0001837~epithelial to mesenchymal transition	3	1.470588	0.24541	ENSMODG00000001183, ENSMODG00000004260, ENSMODG00000010173	3.14	8.00E-01	99.38
GOTERM_BP_ALL	GO:0090090~negative regulation of canonical Wnt signaling pathway	3	1.470588	0.292073	ENSMODG00000001183, ENSMODG00000004260, ENSMODG00000010173	2.77	8.42E-01	99.80
Annotation Cluster 28	Transcription	Enrichment Score: 0.9672023664623242						
Category	Term	Count	%	PValue	Genes	Fold Enrichment	Benjamini	FDR
GOTERM_BP_ALL	GO:0045892~negative regulation of transcription, DNA-templated	13	6.372549	0.095203	ENSMODG00000016387, ENSMODG00000001183, ENSMODG00000006068, ENSMODG00000014680, ENSMODG00000016375, ENSMODG00000014926, ENSMODG00000018991, ENSMODG00000002137, ENSMODG00000018597, ENSMODG00000014540, ENSMODG00000003112, ENSMODG00000010173, ENSMODG00000002992	1.64	5.62E-01	83.55
GOTERM_BP_ALL	GO:1903507~negative regulation of nucleic acid-templated transcription	13	6.372549	0.097085	ENSMODG00000016387, ENSMODG00000001183, ENSMODG00000006068, ENSMODG00000014680, ENSMODG00000016375, ENSMODG00000014926, ENSMODG00000018991, ENSMODG00000002137, ENSMODG00000018597, ENSMODG00000014540, ENSMODG00000003112, ENSMODG00000010173, ENSMODG00000002992	1.64	5.64E-01	84.16
GOTERM_BP_ALL	GO:1902679~negative regulation of RNA biosynthetic process	13	6.372549	0.105835	ENSMODG00000016387, ENSMODG00000001183, ENSMODG00000006068, ENSMODG00000014680, ENSMODG00000016375, ENSMODG00000014926, ENSMODG00000018991, ENSMODG00000002137, ENSMODG00000018597, ENSMODG00000014540, ENSMODG00000003112, ENSMODG00000010173, ENSMODG00000002992	1.61	5.80E-01	86.71
GOTERM_BP_ALL	GO:0051253~negative regulation of RNA metabolic process	13	6.372549	0.13828	ENSMODG00000016387, ENSMODG00000001183, ENSMODG00000006068, ENSMODG00000014680, ENSMODG00000016375, ENSMODG00000014926, ENSMODG00000018991, ENSMODG00000002137, ENSMODG00000018597, ENSMODG00000014540, ENSMODG00000003112, ENSMODG00000010173, ENSMODG00000002992	1.53	6.52E-01	93.18
Annotation Cluster 29	Cell cycle	Enrichment Score: 0.959268922263687						
Category	Term	Count	%	PValue	Genes	Fold Enrichment	Benjamini	FDR
GOTERM_BP_ALL	GO:0000082~G1/S transition of mitotic cell cycle	5	2.45098	0.030984	ENSMODG00000011338, ENSMODG00000002290, ENSMODG00000015171, ENSMODG00000020725, ENSMODG00000019560	4.19	3.23E-01	43.33
GOTERM_BP_ALL	GO:0044843~cell cycle G1/S phase transition	5	2.45098	0.046724	ENSMODG00000011338, ENSMODG00000002290, ENSMODG00000015171, ENSMODG00000020725, ENSMODG00000019560	3.67	4.12E-01	57.82
GOTERM_BP_ALL	GO:0044772~mitotic cell cycle phase transition	5	2.45098	0.289896	ENSMODG00000011338, ENSMODG00000002290, ENSMODG00000015171, ENSMODG00000020725, ENSMODG00000019560	1.83	8.41E-01	99.79
GOTERM_BP_ALL	GO:0044770~cell cycle phase transition	5	2.45098	0.346745	ENSMODG00000011338, ENSMODG00000002290, ENSMODG00000015171, ENSMODG00000020725, ENSMODG00000019560	1.67	8.83E-01	99.95
Annotation Cluster 30	Kinase	Enrichment Score: 0.9204037087680441						
Category	Term	Count	%	PValue	Genes	Fold Enrichment	Benjamini	FDR

GOTERM_MF_ALL	GO:0004672~protein kinase activity	11	5.392157	0.061362	ENSMODG00000012068, ENSMODG00000018418, ENSMODG00000015165, ENSMODG00000001074, ENSMODG00000003798, ENSMODG00000007007, ENSMODG00000019560, ENSMODG00000015051, ENSMODG00000004739, ENSMODG00000005255, ENSMODG00000018983	1.90	5.57E-01	58.81
GOTERM_MF_ALL	GO:0016773~phosphotransferase activity, alcohol group as acceptor	11	5.392157	0.139569	ENSMODG00000012068, ENSMODG00000018418, ENSMODG00000015165, ENSMODG00000001074, ENSMODG00000003798, ENSMODG00000007007, ENSMODG00000019560, ENSMODG00000015051, ENSMODG00000004739, ENSMODG00000005255, ENSMODG00000018983	1.61	7.71E-01	87.82
GOTERM_MF_ALL	GO:0016301~kinase activity	11	5.392157	0.202348	ENSMODG00000012068, ENSMODG00000018418, ENSMODG00000015165, ENSMODG00000001074, ENSMODG00000003798, ENSMODG00000007007, ENSMODG00000019560, ENSMODG00000015051, ENSMODG00000004739, ENSMODG00000005255, ENSMODG00000018983	1.48	8.28E-01	95.79
Annotation Cluster 31	Biosynthesis	Enrichment Score: 0.8670007740607152						
Category	Term	Count	%	PValue	Genes	Fold Enrichment	Benjamini	FDR
GOTERM_BP_ALL	GO:0010557~positive regulation of macromolecule biosynthetic process	18	8.823529	0.101608	ENSMODG00000016387, ENSMODG00000015125, ENSMODG00000012059, ENSMODG00000029105, ENSMODG00000018991, ENSMODG00000007847, ENSMODG00000002992, ENSMODG00000011338, ENSMODG00000000453, ENSMODG00000001183, ENSMODG00000003856, ENSMODG00000014680, ENSMODG00000001074, ENSMODG00000014926, ENSMODG00000003548, ENSMODG00000020725, ENSMODG00000016890, ENSMODG00000014540	1.47	5.71E-01	85.53
GOTERM_BP_ALL	GO:0031328~positive regulation of cellular biosynthetic process	18	8.823529	0.149596	ENSMODG00000016387, ENSMODG00000015125, ENSMODG00000012059, ENSMODG00000029105, ENSMODG00000018991, ENSMODG00000007847, ENSMODG00000002992, ENSMODG00000011338, ENSMODG00000000453, ENSMODG00000001183, ENSMODG00000003856, ENSMODG00000014680, ENSMODG00000001074, ENSMODG00000014926, ENSMODG00000003548, ENSMODG00000020725, ENSMODG00000016890, ENSMODG00000014540	1.38	6.69E-01	94.63
GOTERM_BP_ALL	GO:0009891~positive regulation of biosynthetic process	18	8.823529	0.164872	ENSMODG00000016387, ENSMODG00000015125, ENSMODG00000012059, ENSMODG00000029105, ENSMODG00000018991, ENSMODG00000007847, ENSMODG00000002992, ENSMODG00000011338, ENSMODG00000000453, ENSMODG00000001183, ENSMODG00000003856, ENSMODG00000014680, ENSMODG00000001074, ENSMODG00000014926, ENSMODG00000003548, ENSMODG00000020725, ENSMODG00000016890, ENSMODG00000014540	1.36	6.96E-01	96.12
Annotation Cluster 32	Cell motility	Enrichment Score: 0.7561472904902939						
Category	Term	Count	%	PValue	Genes	Fold Enrichment	Benjamini	FDR
INTERPRO	IPR001752:Kinesin, motor domain	3	1.470588	0.088305	ENSMODG00000019548, ENSMODG00000006420, ENSMODG00000002620	6.00	8.39E-01	72.88
GOTERM_CC_ALL	GO:0005871~kinesin complex	3	1.470588	0.12127	ENSMODG00000019548, ENSMODG00000006420, ENSMODG00000002620	4.96	5.63E-01	82.63
SMART	SM00129:KISc	3	1.470588	0.149128	ENSMODG00000019548, ENSMODG00000006420, ENSMODG00000002620	4.35	8.77E-01	84.75
GOTERM_MF_ALL	GO:0003777~microtubule motor activity	3	1.470588	0.16706	ENSMODG00000019548, ENSMODG00000006420, ENSMODG00000002620	4.05	8.05E-01	92.27
GOTERM_MF_ALL	GO:0003774~motor activity	3	1.470588	0.288833	ENSMODG00000019548, ENSMODG00000006420, ENSMODG00000002620	2.79	8.96E-01	99.16
GOTERM_CC_ALL	GO:0005875~microtubule associated complex	3	1.470588	0.376962	ENSMODG00000019548, ENSMODG00000006420, ENSMODG00000002620	2.28	8.59E-01	99.83
Annotation Cluster 33	Fibroblast function	Enrichment Score: 0.7500817227230353						
Category	Term	Count	%	PValue	Genes	Fold Enrichment	Benjamini	FDR
GOTERM_BP_ALL	GO:0008543~fibroblast growth factor receptor signaling pathway	3	1.470588	0.149274	ENSMODG00000029105, ENSMODG00000018877, ENSMODG00000003513	4.36	6.69E-01	94.59
GOTERM_BP_ALL	GO:0071774~response to fibroblast growth factor	3	1.470588	0.194037	ENSMODG00000029105, ENSMODG00000018877, ENSMODG00000003513	3.68	7.41E-01	97.96
GOTERM_BP_ALL	GO:0044344~cellular response to fibroblast growth factor stimulus	3	1.470588	0.194037	ENSMODG00000029105, ENSMODG00000018877, ENSMODG00000003513	3.68	7.41E-01	97.96
Annotation Cluster 34	Laminins	Enrichment Score: 0.6919804522293099						
Category	Term	Count	%	PValue	Genes	Fold Enrichment	Benjamini	FDR
INTERPRO	IPR001791:Laminin G domain	3	1.470588	0.115103	ENSMODG00000029358, ENSMODG00000020329, ENSMODG00000018877	5.12	8.80E-01	82.20
SMART	SM00282:LamG	3	1.470588	0.115235	ENSMODG00000029358, ENSMODG00000020329, ENSMODG00000018877	5.10	8.63E-01	75.97
INTERPRO	IPR013320:Concanavalin A-like lectin/glucanase, subgroup	3	1.470588	0.632977	ENSMODG00000029358, ENSMODG00000020329, ENSMODG00000018877	1.40	1.00E+00	100.00
Annotation Cluster 35	Contraction	Enrichment Score: 0.6757532131069676						
Category	Term	Count	%	PValue	Genes	Fold Enrichment	Benjamini	FDR

GOTERM_BP_ALL	GO:0008016~regulation of heart contraction	4	1.960784	0.160498	ENSMODG00000003798, ENSMODG00000017022, ENSMODG00000009157, ENSMODG00000018947	2.88	6.88E-01	95.74
GOTERM_BP_ALL	GO:0060047~heart contraction	4	1.960784	0.20593	ENSMODG00000003798, ENSMODG00000017022, ENSMODG00000009157, ENSMODG00000018947	2.54	7.59E-01	98.44
GOTERM_BP_ALL	GO:0003015~heart process	4	1.960784	0.216811	ENSMODG00000003798, ENSMODG00000017022, ENSMODG00000009157, ENSMODG00000018947	2.48	7.72E-01	98.78
GOTERM_BP_ALL	GO:1903522~regulation of blood circulation	4	1.960784	0.276514	ENSMODG00000003798, ENSMODG00000017022, ENSMODG00000009157, ENSMODG00000018947	2.17	8.30E-01	99.71
Annotation Cluster 36	Protein localisation	Enrichment Score: 0.6618150685887341						
Category	Term	Count	%	PValue	Genes	Fold Enrichment	Benjamini	FDR
GOTERM_BP_ALL	GO:0032507~maintenance of protein location in cell	3	1.470588	0.194037	ENSMODG00000020725, ENSMODG00000025001, ENSMODG00000004633	3.68	7.41E-01	97.96
GOTERM_BP_ALL	GO:0045185~maintenance of protein location	3	1.470588	0.204217	ENSMODG00000020725, ENSMODG00000025001, ENSMODG00000004633	3.56	7.57E-01	98.38
GOTERM_BP_ALL	GO:0051651~maintenance of location in cell	3	1.470588	0.260963	ENSMODG00000020725, ENSMODG00000025001, ENSMODG00000004633	3.01	8.14E-01	99.57
Annotation Cluster 37	Morphogenesis	Enrichment Score: 0.6595343239992638						
Category	Term	Count	%	PValue	Genes	Fold Enrichment	Benjamini	FDR
GOTERM_BP_ALL	GO:0090102~cochlea development	3	1.470588	0.07719	ENSMODG00000018014, ENSMODG00000007847, ENSMODG00000018983	6.47	5.16E-01	76.53
GOTERM_BP_ALL	GO:0042472~inner ear morphogenesis	3	1.470588	0.333264	ENSMODG00000018014, ENSMODG00000007847, ENSMODG00000018983	2.51	8.75E-01	99.93
GOTERM_BP_ALL	GO:0042471~ear morphogenesis	3	1.470588	0.408366	ENSMODG00000018014, ENSMODG00000007847, ENSMODG00000018983	2.14	9.20E-01	99.99
Annotation Cluster 38	Biosynthesis	Enrichment Score: 0.6540162240905547						
Category	Term	Count	%	PValue	Genes	Fold Enrichment	Benjamini	FDR
GOTERM_BP_ALL	GO:0034654~nucleobase-containing compound biosynthetic process	36	17.64706	0.182502	ENSMODG00000016375, ENSMODG00000012059, ENSMODG00000018991, ENSMODG00000007007, ENSMODG00000000658, ENSMODG00000003112, ENSMODG00000011437, ENSMODG00000010173, ENSMODG00000001183, ENSMODG00000000453, ENSMODG00000020725, ENSMODG00000002137, ENSMODG00000013409, ENSMODG00000016387, ENSMODG00000006068, ENSMODG00000029105, ENSMODG00000015171, ENSMODG00000018927, ENSMODG00000008171, ENSMODG00000007847, ENSMODG00000012390, ENSMODG00000024585, ENSMODG00000002992, ENSMODG00000003856, ENSMODG00000014680, ENSMODG00000001074, ENSMODG00000014926, ENSMODG00000011920, ENSMODG00000003548, ENSMODG00000004260, ENSMODG00000016890, ENSMODG00000018597, ENSMODG00000002910, ENSMODG00000021299, ENSMODG00000014540, ENSMODG00000015403	1.19	7.22E-01	97.36
GOTERM_BP_ALL	GO:0018130~heterocycle biosynthetic process	36	17.64706	0.218881	ENSMODG00000016375, ENSMODG00000012059, ENSMODG00000018991, ENSMODG00000007007, ENSMODG00000000658, ENSMODG00000003112, ENSMODG00000011437, ENSMODG00000010173, ENSMODG00000001183, ENSMODG00000000453, ENSMODG00000020725, ENSMODG00000002137, ENSMODG00000013409, ENSMODG00000016387, ENSMODG00000006068, ENSMODG00000029105, ENSMODG00000015171, ENSMODG00000018927, ENSMODG00000008171, ENSMODG00000007847, ENSMODG00000012390, ENSMODG00000024585, ENSMODG00000002992, ENSMODG00000003856, ENSMODG00000014680, ENSMODG00000001074, ENSMODG00000014926, ENSMODG00000011920, ENSMODG00000003548, ENSMODG00000004260, ENSMODG00000016890, ENSMODG00000018597, ENSMODG00000002910, ENSMODG00000021299, ENSMODG00000014540, ENSMODG00000015403	1.17	7.75E-01	98.84
GOTERM_BP_ALL	GO:0019438~aromatic compound biosynthetic process	36	17.64706	0.222285	ENSMODG00000016375, ENSMODG00000012059, ENSMODG00000018991, ENSMODG00000007007, ENSMODG00000000658, ENSMODG00000003112, ENSMODG00000011437, ENSMODG00000010173, ENSMODG00000001183, ENSMODG00000000453, ENSMODG00000020725, ENSMODG00000002137, ENSMODG00000013409, ENSMODG00000016387, ENSMODG00000006068, ENSMODG00000029105, ENSMODG00000015171, ENSMODG00000018927, ENSMODG00000008171, ENSMODG00000007847, ENSMODG00000012390, ENSMODG00000024585, ENSMODG00000002992, ENSMODG00000003856, ENSMODG00000014680, ENSMODG00000001074, ENSMODG00000014926, ENSMODG00000011920, ENSMODG00000003548, ENSMODG00000004260, ENSMODG00000016890, ENSMODG00000018597, ENSMODG00000002910, ENSMODG00000021299, ENSMODG00000014540, ENSMODG00000015403	1.16	7.77E-01	98.93

GOTERM_BP_ALL	GO:1901362~organic cyclic compound biosynthetic process	36	17.64706	0.272615	ENSMODG00000016375, ENSMODG00000012059, ENSMODG00000018991, ENSMODG00000007007, ENSMODG00000000658, ENSMODG00000003112, ENSMODG00000011437, ENSMODG00000010173, ENSMODG00000001183, ENSMODG00000000453, ENSMODG00000020725, ENSMODG00000002137, ENSMODG00000013409, ENSMODG00000016387, ENSMODG00000006068, ENSMODG00000029105, ENSMODG00000015171, ENSMODG00000018927, ENSMODG00000008171, ENSMODG00000007847, ENSMODG00000012390, ENSMODG00000024585, ENSMODG00000002992, ENSMODG00000003856, ENSMODG00000014680, ENSMODG00000001074, ENSMODG00000014926, ENSMODG00000011920, ENSMODG00000003548, ENSMODG00000004260, ENSMODG00000016890, ENSMODG00000018597, ENSMODG00000002910, ENSMODG00000021299, ENSMODG00000014540, ENSMODG00000015403	1.13	8.26E-01	99.68
Annotation Cluster 39	Cellular organization	Enrichment Score: 0.6357919450720831						
Category	Term	Count	%	PValue	Genes	Fold Enrichment	Benjamini	FDR
GOTERM_BP_ALL	GO:0042384~cilium assembly	5	2.45098	0.169475	ENSMODG00000016160, ENSMODG00000018927, ENSMODG00000025001, ENSMODG00000013389, ENSMODG00000016420	2.31	7.02E-01	96.49
GOTERM_BP_ALL	GO:0044782~cilium organization	5	2.45098	0.210624	ENSMODG00000016160, ENSMODG00000018927, ENSMODG00000025001, ENSMODG00000013389, ENSMODG00000016420	2.11	7.65E-01	98.60
GOTERM_BP_ALL	GO:0010927~cellular component assembly involved in morphogenesis	5	2.45098	0.346745	ENSMODG00000016160, ENSMODG00000018927, ENSMODG00000025001, ENSMODG00000013389, ENSMODG00000016420	1.67	8.83E-01	99.95
Annotation Cluster 40	Peptidase	Enrichment Score: 0.6299661861255804						
Category	Term	Count	%	PValue	Genes	Fold Enrichment	Benjamini	FDR
GOTERM_MF_ALL	GO:0008235~metalloexopeptidase activity	3	1.470588	0.106743	ENSMODG00000020425, ENSMODG00000006871, ENSMODG00000016176	5.35	6.87E-01	79.43
GOTERM_MF_ALL	GO:0008238~exopeptidase activity	3	1.470588	0.236067	ENSMODG00000020425, ENSMODG00000006871, ENSMODG00000016176	3.22	8.52E-01	97.70
GOTERM_MF_ALL	GO:0008237~metallopeptidase activity	3	1.470588	0.511359	ENSMODG00000020425, ENSMODG00000006871, ENSMODG00000016176	1.75	9.52E-01	100.00
Annotation Cluster 41	Ankyrin	Enrichment Score: 0.6225320381497007						
Category	Term	Count	%	PValue	Genes	Fold Enrichment	Benjamini	FDR
INTERPRO	IPR002110:Ankyrin repeat	5	2.45098	0.187206	ENSMODG00000012068, ENSMODG00000021156, ENSMODG00000003766, ENSMODG00000001539, ENSMODG00000014540	2.21	9.60E-01	94.64
INTERPRO	IPR020683:Ankyrin repeat-containing domain	5	2.45098	0.20267	ENSMODG00000012068, ENSMODG00000021156, ENSMODG00000003766, ENSMODG00000001539, ENSMODG00000014540	2.15	9.58E-01	95.91
SMART	SM00248:ANK	5	2.45098	0.357515	ENSMODG00000012068, ENSMODG00000021156, ENSMODG00000003766, ENSMODG00000001539, ENSMODG00000014540	1.64	9.84E-01	99.42
Annotation Cluster 42	Protein transport	Enrichment Score: 0.6197547704233994						
Category	Term	Count	%	PValue	Genes	Fold Enrichment	Benjamini	FDR
GOTERM_BP_ALL	GO:0042307~positive regulation of protein import into nucleus	3	1.470588	0.214454	ENSMODG00000001183, ENSMODG00000014680, ENSMODG00000004834	3.44	7.69E-01	98.72
GOTERM_BP_ALL	GO:1904591~positive regulation of protein import	3	1.470588	0.224739	ENSMODG00000001183, ENSMODG00000014680, ENSMODG00000004834	3.34	7.80E-01	98.99
GOTERM_BP_ALL	GO:0046824~positive regulation of nucleocytoplasmic transport	3	1.470588	0.286895	ENSMODG00000001183, ENSMODG00000014680, ENSMODG00000004834	2.81	8.38E-01	99.78
Annotation Cluster 43	Endopeptidase	Enrichment Score: 0.6142915261700133						
Category	Term	Count	%	PValue	Genes	Fold Enrichment	Benjamini	FDR
GOTERM_BP_ALL	GO:0043280~positive regulation of cysteine-type endopeptidase activity involved in apoptotic process	3	1.470588	0.209329	ENSMODG00000019579, ENSMODG00000018597, ENSMODG00000016857	3.50	7.64E-01	98.56
GOTERM_BP_ALL	GO:2001056~positive regulation of cysteine-type endopeptidase activity	3	1.470588	0.240233	ENSMODG00000019579, ENSMODG00000018597, ENSMODG00000016857	3.19	7.95E-01	99.30
GOTERM_BP_ALL	GO:0010950~positive regulation of endopeptidase activity	3	1.470588	0.255776	ENSMODG00000019579, ENSMODG00000018597, ENSMODG00000016857	3.05	8.08E-01	99.52
GOTERM_BP_ALL	GO:0010952~positive regulation of peptidase activity	3	1.470588	0.271339	ENSMODG00000019579, ENSMODG00000018597, ENSMODG00000016857	2.93	8.25E-01	99.67
Annotation Cluster 44	Transcription	Enrichment Score: 0.6141747941431892						
Category	Term	Count	%	PValue	Genes	Fold Enrichment	Benjamini	FDR
GOTERM_BP_ALL	GO:1903508~positive regulation of nucleic acid-templated transcription	14	6.862745	0.229599	ENSMODG00000016387, ENSMODG00000001183, ENSMODG00000000453, ENSMODG00000014680, ENSMODG00000012059, ENSMODG00000001074, ENSMODG00000029105, ENSMODG00000014926, ENSMODG00000003548, ENSMODG00000018991, ENSMODG00000020725, ENSMODG00000007847, ENSMODG00000014540, ENSMODG00000002992	1.35	7.85E-01	99.10

GOTERM_BP_ALL	GO:0045893~positive regulation of transcription, DNA-templated	14	6.862745	0.229599	ENSMODG00000016387, ENSMODG00000001183, ENSMODG00000000453, ENSMODG00000014680, ENSMODG00000012059, ENSMODG00000001074, ENSMODG00000029105, ENSMODG00000014926, ENSMODG00000003548, ENSMODG00000018991, ENSMODG00000020725, ENSMODG00000007847, ENSMODG00000014540, ENSMODG00000002992	1.35	7.85E-01	99.10
GOTERM_BP_ALL	GO:1902680~positive regulation of RNA biosynthetic process	14	6.862745	0.235335	ENSMODG00000016387, ENSMODG00000001183, ENSMODG00000000453, ENSMODG00000014680, ENSMODG00000012059, ENSMODG00000001074, ENSMODG00000029105, ENSMODG00000014926, ENSMODG00000003548, ENSMODG00000018991, ENSMODG00000020725, ENSMODG00000007847, ENSMODG00000014540, ENSMODG00000002992	1.34	7.90E-01	99.21
GOTERM_BP_ALL	GO:0051254~positive regulation of RNA metabolic process	14	6.862745	0.281627	ENSMODG00000016387, ENSMODG00000001183, ENSMODG00000000453, ENSMODG00000014680, ENSMODG00000012059, ENSMODG00000001074, ENSMODG00000029105, ENSMODG00000014926, ENSMODG00000003548, ENSMODG00000018991, ENSMODG00000020725, ENSMODG00000007847, ENSMODG00000014540, ENSMODG00000002992	1.29	8.34E-01	99.74
Annotation Cluster 45	Endopeptidase	Enrichment Score: 0.5992024452935635						
Category	Term	Count	%	PValue	Genes	Fold Enrichment	Benjamini	FDR
GOTERM_BP_ALL	GO:0043281~regulation of cysteine-type endopeptidase activity involved in apoptotic process	4	1.960784	0.19163	ENSMODG00000019579, ENSMODG00000001183, ENSMODG00000018597, ENSMODG00000016857	2.64	7.37E-01	97.85
GOTERM_BP_ALL	GO:2000116~regulation of cysteine-type endopeptidase activity	4	1.960784	0.22781	ENSMODG00000019579, ENSMODG00000001183, ENSMODG00000018597, ENSMODG00000016857	2.41	7.83E-01	99.06
GOTERM_BP_ALL	GO:0052548~regulation of endopeptidase activity	4	1.960784	0.295546	ENSMODG00000019579, ENSMODG00000001183, ENSMODG00000018597, ENSMODG00000016857	2.09	8.45E-01	99.82
GOTERM_BP_ALL	GO:0052547~regulation of peptidase activity	4	1.960784	0.310833	ENSMODG00000019579, ENSMODG00000001183, ENSMODG00000018597, ENSMODG00000016857	2.03	8.58E-01	99.88
Annotation Cluster 46	Signaling	Enrichment Score: 0.5866021513906475						
Category	Term	Count	%	PValue	Genes	Fold Enrichment	Benjamini	FDR
GOTERM_BP_ALL	GO:0009968~negative regulation of signal transduction	13	6.372549	0.218762	ENSMODG00000016375, ENSMODG00000010848, ENSMODG00000010173, ENSMODG00000009971, ENSMODG00000002992, ENSMODG00000001183, ENSMODG00000001074, ENSMODG00000011920, ENSMODG00000020725, ENSMODG00000004260, ENSMODG00000021440, ENSMODG00000016857, ENSMODG00000003513	1.39	7.75E-01	98.84
GOTERM_BP_ALL	GO:0010648~negative regulation of cell communication	13	6.372549	0.280305	ENSMODG00000016375, ENSMODG00000010848, ENSMODG00000010173, ENSMODG00000009971, ENSMODG00000002992, ENSMODG00000001183, ENSMODG00000001074, ENSMODG00000011920, ENSMODG00000020725, ENSMODG00000004260, ENSMODG00000021440, ENSMODG00000016857, ENSMODG00000003513	1.31	8.33E-01	99.74
GOTERM_BP_ALL	GO:0023057~negative regulation of signaling	13	6.372549	0.283525	ENSMODG00000016375, ENSMODG00000010848, ENSMODG00000010173, ENSMODG00000009971, ENSMODG00000002992, ENSMODG00000001183, ENSMODG00000001074, ENSMODG00000011920, ENSMODG00000020725, ENSMODG00000004260, ENSMODG00000021440, ENSMODG00000016857, ENSMODG00000003513	1.31	8.35E-01	99.76
Annotation Cluster 47	Nucleotide binding	Enrichment Score: 0.5549107667129044						
Category	Term	Count	%	PValue	Genes	Fold Enrichment	Benjamini	FDR
GOTERM_MF_ALL	GO:0005524~ATP binding	16	7.843137	0.261608	ENSMODG00000012068, ENSMODG00000015165, ENSMODG00000007007, ENSMODG00000019560, ENSMODG00000015051, ENSMODG00000018983, ENSMODG00000011014, ENSMODG00000018418, ENSMODG00000001074, ENSMODG00000019548, ENSMODG00000001152, ENSMODG00000006420, ENSMODG00000002620, ENSMODG00000016890, ENSMODG00000004739, ENSMODG00000005255	1.28	8.80E-01	98.57
GOTERM_MF_ALL	GO:0032559~adenyl ribonucleotide binding	16	7.843137	0.284554	ENSMODG00000012068, ENSMODG00000015165, ENSMODG00000007007, ENSMODG00000019560, ENSMODG00000015051, ENSMODG00000018983, ENSMODG00000011014, ENSMODG00000018418, ENSMODG00000001074, ENSMODG00000019548, ENSMODG00000001152, ENSMODG00000006420, ENSMODG00000002620, ENSMODG00000016890, ENSMODG00000004739, ENSMODG00000005255	1.25	8.96E-01	99.08

GOTERM_MF_ALL	GO:0030554~adenyl nucleotide binding	16	7.843137	0.290705	ENSMODG00000012068, ENSMODG00000015165, ENSMODG00000007007, ENSMODG00000019560, ENSMODG00000015051, ENSMODG00000018983, ENSMODG00000011014, ENSMODG00000018418, ENSMODG00000001074, ENSMODG00000019548, ENSMODG00000001152, ENSMODG00000006420, ENSMODG00000002620, ENSMODG00000016890, ENSMODG00000004739, ENSMODG00000005255	1.25	8.90E-01	99.19
Annotation Cluster 48	Phosphorylation	Enrichment Score: 0.5172701837390364						
Category	Term	Count	%	PValue	Genes	Fold Enrichment	Benjamini	FDR
GOTERM_BP_ALL	GO:0050730~regulation of peptidyl-tyrosine phosphorylation	4	1.960784	0.246369	ENSMODG00000012068, ENSMODG00000001183, ENSMODG00000003977, ENSMODG00000019334	2.31	8.00E-01	99.39
GOTERM_BP_ALL	GO:0018108~peptidyl-tyrosine phosphorylation	4	1.960784	0.333804	ENSMODG00000012068, ENSMODG00000001183, ENSMODG00000003977, ENSMODG00000019334	1.95	8.75E-01	99.93
GOTERM_BP_ALL	GO:0018212~peptidyl-tyrosine modification	4	1.960784	0.34128	ENSMODG00000012068, ENSMODG00000001183, ENSMODG00000003977, ENSMODG00000019334	1.92	8.80E-01	99.95
Annotation Cluster 49	Immunity	Enrichment Score: 0.4617191430275958						
Category	Term	Count	%	PValue	Genes	Fold Enrichment	Benjamini	FDR
GOTERM_BP_ALL	GO:0070489~T cell aggregation	6	2.941176	0.25502	ENSMODG00000011338, ENSMODG00000005170, ENSMODG00000001161, ENSMODG00000010122, ENSMODG00000007847, ENSMODG00000018947	1.75	8.08E-01	99.51
GOTERM_BP_ALL	GO:0071593~lymphocyte aggregation	6	2.941176	0.25502	ENSMODG00000011338, ENSMODG00000005170, ENSMODG00000001161, ENSMODG00000010122, ENSMODG00000007847, ENSMODG00000018947	1.75	8.08E-01	99.51
GOTERM_BP_ALL	GO:0042110~T cell activation	6	2.941176	0.25502	ENSMODG00000011338, ENSMODG00000005170, ENSMODG00000001161, ENSMODG00000010122, ENSMODG00000007847, ENSMODG00000018947	1.75	8.08E-01	99.51
GOTERM_BP_ALL	GO:0070486~leukocyte aggregation	6	2.941176	0.273614	ENSMODG00000011338, ENSMODG00000005170, ENSMODG00000001161, ENSMODG00000010122, ENSMODG00000007847, ENSMODG00000018947	1.70	8.27E-01	99.69
GOTERM_BP_ALL	GO:0046649~lymphocyte activation	6	2.941176	0.558131	ENSMODG00000011338, ENSMODG00000005170, ENSMODG00000001161, ENSMODG00000010122, ENSMODG00000007847, ENSMODG00000018947	1.20	9.72E-01	100.00
GOTERM_BP_ALL	GO:0045321~leukocyte activation	6	2.941176	0.670024	ENSMODG00000011338, ENSMODG00000005170, ENSMODG00000001161, ENSMODG00000010122, ENSMODG00000007847, ENSMODG00000018947	1.06	9.89E-01	100.00
Annotation Cluster 50	DNA binding	Enrichment Score: 0.44390038060124415						
Category	Term	Count	%	PValue	Genes	Fold Enrichment	Benjamini	FDR
GOTERM_MF_ALL	GO:0000978~RNA polymerase II core promoter proximal region sequence-specific DNA binding	5	2.45098	0.33595	ENSMODG00000019416, ENSMODG00000012059, ENSMODG00000015190, ENSMODG00000018597, ENSMODG00000002992	1.70	9.16E-01	99.68
GOTERM_MF_ALL	GO:0000987~core promoter proximal region sequence-specific DNA binding	5	2.45098	0.371055	ENSMODG00000019416, ENSMODG00000012059, ENSMODG00000015190, ENSMODG00000018597, ENSMODG00000002992	1.61	9.24E-01	99.85
GOTERM_MF_ALL	GO:0001159~core promoter proximal region DNA binding	5	2.45098	0.373754	ENSMODG00000019416, ENSMODG00000012059, ENSMODG00000015190, ENSMODG00000018597, ENSMODG00000002992	1.60	9.24E-01	99.86
Annotation Cluster 51	Hydrolysis	Enrichment Score: 0.43839407267050023						
Category	Term	Count	%	PValue	Genes	Fold Enrichment	Benjamini	FDR
GOTERM_MF_ALL	GO:0016462~pyrophosphatase activity	9	4.411765	0.35784	ENSMODG00000011014, ENSMODG00000003856, ENSMODG00000019548, ENSMODG00000001152, ENSMODG00000006420, ENSMODG00000016890, ENSMODG00000002620, ENSMODG00000003977, ENSMODG00000017938	1.33	9.23E-01	99.80
GOTERM_MF_ALL	GO:0016818~hydrolase activity, acting on acid anhydrides, in phosphorus-containing anhydrides	9	4.411765	0.361462	ENSMODG00000011014, ENSMODG00000003856, ENSMODG00000019548, ENSMODG00000001152, ENSMODG00000006420, ENSMODG00000016890, ENSMODG00000002620, ENSMODG00000003977, ENSMODG00000017938	1.32	9.23E-01	99.81
GOTERM_MF_ALL	GO:0016817~hydrolase activity, acting on acid anhydrides	9	4.411765	0.374168	ENSMODG00000011014, ENSMODG00000003856, ENSMODG00000019548, ENSMODG00000001152, ENSMODG00000006420, ENSMODG00000016890, ENSMODG00000002620, ENSMODG00000003977, ENSMODG00000017938	1.31	9.21E-01	99.86
Annotation Cluster 52	Metabolism	Enrichment Score: 0.42969545995350944						
Category	Term	Count	%	PValue	Genes	Fold Enrichment	Benjamini	FDR
GOTERM_BP_ALL	GO:0030799~regulation of cyclic nucleotide metabolic process	3	1.470588	0.266151	ENSMODG00000020725, ENSMODG00000000658, ENSMODG00000018947	2.97	8.19E-01	99.62
GOTERM_BP_ALL	GO:1900542~regulation of purine nucleotide metabolic process	3	1.470588	0.427728	ENSMODG00000020725, ENSMODG00000000658, ENSMODG00000018947	2.05	9.30E-01	100.00
GOTERM_BP_ALL	GO:0006140~regulation of nucleotide metabolic process	3	1.470588	0.451458	ENSMODG00000020725, ENSMODG00000000658, ENSMODG00000018947	1.96	9.41E-01	100.00
Annotation Cluster 53	Transcription	Enrichment Score: 0.4188889185757528						

Category	Term	Count	%	PValue	Genes	Fold Enrichment	Benjamini	FDR
GOTERM_BP_ALL	GO:0006355~regulation of transcription, DNA-templated	28	13.72549	0.32115	ENSMODG00000016375, ENSMODG00000012059, ENSMODG00000018991, ENSMODG00000010173, ENSMODG00000003112, ENSMODG00000000453, ENSMODG00000001183, ENSMODG00000020725, ENSMODG00000002137, ENSMODG00000013409, ENSMODG00000016387, ENSMODG00000006068, ENSMODG00000029105, ENSMODG00000018927, ENSMODG00000007847, ENSMODG00000012390, ENSMODG00000024585, ENSMODG00000002992, ENSMODG00000014680, ENSMODG00000001074, ENSMODG00000011920, ENSMODG00000014926, ENSMODG00000003548, ENSMODG00000004260, ENSMODG00000018597, ENSMODG00000021299, ENSMODG00000014540, ENSMODG00000015403	1.14	8.67E-01	99.91
GOTERM_BP_ALL	GO:1903506~regulation of nucleic acid-templated transcription	28	13.72549	0.325642	ENSMODG00000016375, ENSMODG00000012059, ENSMODG00000018991, ENSMODG00000010173, ENSMODG00000003112, ENSMODG00000000453, ENSMODG00000001183, ENSMODG00000020725, ENSMODG00000002137, ENSMODG00000013409, ENSMODG00000016387, ENSMODG00000006068, ENSMODG00000029105, ENSMODG00000018927, ENSMODG00000007847, ENSMODG00000012390, ENSMODG00000024585, ENSMODG00000002992, ENSMODG00000014680, ENSMODG00000001074, ENSMODG00000011920, ENSMODG00000014926, ENSMODG00000003548, ENSMODG00000004260, ENSMODG00000018597, ENSMODG00000021299, ENSMODG00000014540, ENSMODG00000015403	1.13	8.70E-01	99.92
GOTERM_BP_ALL	GO:2001141~regulation of RNA biosynthetic process	28	13.72549	0.330154	ENSMODG00000016375, ENSMODG00000012059, ENSMODG00000018991, ENSMODG00000010173, ENSMODG00000003112, ENSMODG00000000453, ENSMODG00000001183, ENSMODG00000020725, ENSMODG00000002137, ENSMODG00000013409, ENSMODG00000016387, ENSMODG00000006068, ENSMODG00000029105, ENSMODG00000018927, ENSMODG00000007847, ENSMODG00000012390, ENSMODG00000024585, ENSMODG00000002992, ENSMODG00000014680, ENSMODG00000001074, ENSMODG00000011920, ENSMODG00000014926, ENSMODG00000003548, ENSMODG00000004260, ENSMODG00000018597, ENSMODG00000021299, ENSMODG00000014540, ENSMODG00000015403	1.13	8.73E-01	99.93
GOTERM_BP_ALL	GO:0097659~nucleic acid-templated transcription	28	13.72549	0.477367	ENSMODG00000016375, ENSMODG00000012059, ENSMODG00000018991, ENSMODG00000010173, ENSMODG00000003112, ENSMODG00000000453, ENSMODG00000001183, ENSMODG00000020725, ENSMODG00000002137, ENSMODG00000013409, ENSMODG00000016387, ENSMODG00000006068, ENSMODG00000029105, ENSMODG00000018927, ENSMODG00000007847, ENSMODG00000012390, ENSMODG00000024585, ENSMODG00000002992, ENSMODG00000014680, ENSMODG00000001074, ENSMODG00000011920, ENSMODG00000014926, ENSMODG00000003548, ENSMODG00000004260, ENSMODG00000018597, ENSMODG00000021299, ENSMODG00000014540, ENSMODG00000015403	1.06	9.51E-01	100.00
GOTERM_BP_ALL	GO:0032774~RNA biosynthetic process	28	13.72549	0.488134	ENSMODG00000016375, ENSMODG00000012059, ENSMODG00000018991, ENSMODG00000010173, ENSMODG00000003112, ENSMODG00000000453, ENSMODG00000001183, ENSMODG00000020725, ENSMODG00000002137, ENSMODG00000013409, ENSMODG00000016387, ENSMODG00000006068, ENSMODG00000029105, ENSMODG00000018927, ENSMODG00000007847, ENSMODG00000012390, ENSMODG00000024585, ENSMODG00000002992, ENSMODG00000014680, ENSMODG00000001074, ENSMODG00000011920, ENSMODG00000014926, ENSMODG00000003548, ENSMODG00000004260, ENSMODG00000018597, ENSMODG00000021299, ENSMODG00000014540, ENSMODG00000015403	1.05	9.54E-01	100.00
Annotation Cluster 54	Cell cycle	Enrichment Score: 0.4170289452140332						
Category	Term	Count	%	PValue	Genes	Fold Enrichment	Benjamini	FDR
GOTERM_BP_ALL	GO:2000045~regulation of G1/S transition of mitotic cell cycle	3	1.470588	0.178903	ENSMODG00000011338, ENSMODG00000002290, ENSMODG00000020725	3.88	7.17E-01	97.15
GOTERM_BP_ALL	GO:1902806~regulation of cell cycle G1/S phase transition	3	1.470588	0.199119	ENSMODG00000011338, ENSMODG00000002290, ENSMODG00000020725	3.62	7.49E-01	98.18
GOTERM_BP_ALL	GO:0010948~negative regulation of cell cycle process	3	1.470588	0.483729	ENSMODG00000011338, ENSMODG00000002290, ENSMODG00000020725	1.84	9.53E-01	100.00
GOTERM_BP_ALL	GO:0090068~positive regulation of cell cycle process	3	1.470588	0.532013	ENSMODG00000011338, ENSMODG00000002290, ENSMODG00000020725	1.68	9.66E-01	100.00
GOTERM_BP_ALL	GO:1901990~regulation of mitotic cell cycle phase transition	3	1.470588	0.561111	ENSMODG00000011338, ENSMODG00000002290, ENSMODG00000020725	1.59	9.72E-01	100.00
GOTERM_BP_ALL	GO:1901987~regulation of cell cycle phase transition	3	1.470588	0.61168	ENSMODG00000011338, ENSMODG00000002290, ENSMODG00000020725	1.45	9.81E-01	100.00
Annotation Cluster 55	Apoptosis	Enrichment Score: 0.36783054697373657						

Category	Term	Count	%	PValue	Genes	Fold Enrichment	Benjamini	FDR
GOTERM_BP_ALL	GO:2001237~negative regulation of extrinsic apoptotic signaling pathway	3	1.470588	0.250591	ENSMODG00000001183, ENSMODG00000001074, ENSMODG00000010173	3.10	8.04E-01	99.45
GOTERM_BP_ALL	GO:2001236~regulation of extrinsic apoptotic signaling pathway	3	1.470588	0.488243	ENSMODG00000001183, ENSMODG00000001074, ENSMODG00000010173	1.83	9.54E-01	100.00
GOTERM_BP_ALL	GO:0097191~extrinsic apoptotic signaling pathway	3	1.470588	0.64403	ENSMODG00000001183, ENSMODG00000001074, ENSMODG00000010173	1.37	9.86E-01	100.00
Annotation Cluster 56	Nucleotide binding	Enrichment Score: 0.36080922725768233						
Category	Term	Count	%	PValue	Genes	Fold Enrichment	Benjamini	FDR
GOTERM_MF_ALL	GO:0032555~purine ribonucleotide binding	18	8.823529	0.428176	ENSMODG00000012068, ENSMODG00000015165, ENSMODG00000007007, ENSMODG00000019560, ENSMODG00000015051, ENSMODG00000018983, ENSMODG00000011014, ENSMODG00000018418, ENSMODG00000001074, ENSMODG00000019548, ENSMODG00000001152, ENSMODG00000006420, ENSMODG00000002620, ENSMODG00000016890, ENSMODG00000017938, ENSMODG00000018947, ENSMODG00000004739, ENSMODG00000005255	1.12	9.38E-01	99.96
GOTERM_MF_ALL	GO:0017076~purine nucleotide binding	18	8.823529	0.435746	ENSMODG00000012068, ENSMODG00000015165, ENSMODG00000007007, ENSMODG00000019560, ENSMODG00000015051, ENSMODG00000018983, ENSMODG00000011014, ENSMODG00000018418, ENSMODG00000001074, ENSMODG00000019548, ENSMODG00000001152, ENSMODG00000006420, ENSMODG00000002620, ENSMODG00000016890, ENSMODG00000017938, ENSMODG00000018947, ENSMODG00000004739, ENSMODG00000005255	1.12	9.38E-01	99.97
GOTERM_MF_ALL	GO:0032553~ribonucleotide binding	18	8.823529	0.44332	ENSMODG00000012068, ENSMODG00000015165, ENSMODG00000007007, ENSMODG00000019560, ENSMODG00000015051, ENSMODG00000018983, ENSMODG00000011014, ENSMODG00000018418, ENSMODG00000001074, ENSMODG00000019548, ENSMODG00000001152, ENSMODG00000006420, ENSMODG00000002620, ENSMODG00000016890, ENSMODG00000017938, ENSMODG00000018947, ENSMODG00000004739, ENSMODG00000005255	1.11	9.38E-01	99.97
Annotation Cluster 57	Cell cycle	Enrichment Score: 0.3361598266044361						
Category	Term	Count	%	PValue	Genes	Fold Enrichment	Benjamini	FDR
GOTERM_CC_ALL	GO:0000775~chromosome, centromeric region	4	1.960784	0.145984	ENSMODG00000002290, ENSMODG00000003856, ENSMODG00000019261, ENSMODG00000002620	3.01	6.22E-01	88.19
GOTERM_BP_ALL	GO:0007067~mitotic nuclear division	4	1.960784	0.485689	ENSMODG00000002290, ENSMODG00000003856, ENSMODG00000019261, ENSMODG00000002620	1.53	9.54E-01	100.00
GOTERM_BP_ALL	GO:0000280~nuclear division	4	1.960784	0.772701	ENSMODG00000002290, ENSMODG00000003856, ENSMODG00000019261, ENSMODG00000002620	0.99	9.97E-01	100.00
GOTERM_BP_ALL	GO:0048285~organelle fission	4	1.960784	0.82544	ENSMODG00000002290, ENSMODG00000003856, ENSMODG00000019261, ENSMODG00000002620	0.90	9.99E-01	100.00
Annotation Cluster 58	Reproduction	Enrichment Score: 0.3077432439118753						
Category	Term	Count	%	PValue	Genes	Fold Enrichment	Benjamini	FDR
GOTERM_BP_ALL	GO:0007276~gamete generation	6	2.941176	0.378043	ENSMODG00000000453, ENSMODG00000007007, ENSMODG00000020406, ENSMODG00000013389, ENSMODG00000018597, ENSMODG00000018947	1.48	9.02E-01	99.98
GOTERM_BP_ALL	GO:0048609~multicellular organismal reproductive process	6	2.941176	0.55561	ENSMODG00000000453, ENSMODG00000007007, ENSMODG00000020406, ENSMODG00000013389, ENSMODG00000018597, ENSMODG00000018947	1.20	9.71E-01	100.00
GOTERM_BP_ALL	GO:0032504~multicellular organism reproduction	6	2.941176	0.568145	ENSMODG00000000453, ENSMODG00000007007, ENSMODG00000020406, ENSMODG00000013389, ENSMODG00000018597, ENSMODG00000018947	1.19	9.74E-01	100.00
Annotation Cluster 59	Cell morphogenesis	Enrichment Score: 0.30666247966249255						
Category	Term	Count	%	PValue	Genes	Fold Enrichment	Benjamini	FDR
GOTERM_BP_ALL	GO:0030833~regulation of actin filament polymerization	3	1.470588	0.413236	ENSMODG00000021426, ENSMODG00000008346, ENSMODG00000005255	2.11	9.23E-01	99.99
GOTERM_BP_ALL	GO:0008064~regulation of actin polymerization or depolymerization	3	1.470588	0.460795	ENSMODG00000021426, ENSMODG00000008346, ENSMODG00000005255	1.92	9.45E-01	100.00
GOTERM_BP_ALL	GO:0030832~regulation of actin filament length	3	1.470588	0.460795	ENSMODG00000021426, ENSMODG00000008346, ENSMODG00000005255	1.92	9.45E-01	100.00
GOTERM_BP_ALL	GO:0032271~regulation of protein polymerization	3	1.470588	0.492734	ENSMODG00000021426, ENSMODG00000008346, ENSMODG00000005255	1.81	9.56E-01	100.00
GOTERM_BP_ALL	GO:0008154~actin polymerization or depolymerization	3	1.470588	0.51915	ENSMODG00000021426, ENSMODG00000008346, ENSMODG00000005255	1.72	9.63E-01	100.00
GOTERM_BP_ALL	GO:0051258~protein polymerization	3	1.470588	0.64403	ENSMODG00000021426, ENSMODG00000008346, ENSMODG00000005255	1.37	9.86E-01	100.00
Annotation Cluster 60	Homeostasis	Enrichment Score: 0.30446959118520095						

Category	Term	Count	%	PValue	Genes	Fold Enrichment	Benjamini	FDR
GOTERM_BP_ALL	GO:0055065~metal ion homeostasis	6	2.941176	0.403057	ENSMODG00000019403, ENSMODG00000003798, ENSMODG00000009157, ENSMODG00000018766, ENSMODG00000020725, ENSMODG00000000954	1.43	9.18E-01	99.99
GOTERM_BP_ALL	GO:0055080~cation homeostasis	6	2.941176	0.537773	ENSMODG00000019403, ENSMODG00000003798, ENSMODG00000009157, ENSMODG00000018766, ENSMODG00000020725, ENSMODG00000000954	1.23	9.67E-01	100.00
GOTERM_BP_ALL	GO:0098771~inorganic ion homeostasis	6	2.941176	0.563152	ENSMODG00000019403, ENSMODG00000003798, ENSMODG00000009157, ENSMODG00000018766, ENSMODG00000020725, ENSMODG00000000954	1.19	9.73E-01	100.00
Annotation Cluster 61	Nucleoside binding	Enrichment Score: 0.29335730882582367						
Category	Term	Count	%	PValue	Genes	Fold Enrichment	Benjamini	FDR
GOTERM_MF_ALL	GO:0035639~purine ribonucleoside triphosphate binding	17	8.333333	0.49894	ENSMODG00000012068, ENSMODG00000015165, ENSMODG00000007007, ENSMODG00000019560, ENSMODG00000015051, ENSMODG00000018983, ENSMODG00000011014, ENSMODG00000018418, ENSMODG00000001074, ENSMODG00000019548, ENSMODG00000001152, ENSMODG00000006420, ENSMODG00000002620, ENSMODG00000016890, ENSMODG00000017938, ENSMODG00000004739, ENSMODG00000005255	1.08	9.52E-01	99.99
GOTERM_MF_ALL	GO:0032550~purine ribonucleoside binding	17	8.333333	0.507927	ENSMODG00000012068, ENSMODG00000015165, ENSMODG00000007007, ENSMODG00000019560, ENSMODG00000015051, ENSMODG00000018983, ENSMODG00000011014, ENSMODG00000018418, ENSMODG00000001074, ENSMODG00000019548, ENSMODG00000001152, ENSMODG00000006420, ENSMODG00000002620, ENSMODG00000016890, ENSMODG00000017938, ENSMODG00000004739, ENSMODG00000005255	1.07	9.54E-01	100.00
GOTERM_MF_ALL	GO:0001883~purine nucleoside binding	17	8.333333	0.509209	ENSMODG00000012068, ENSMODG00000015165, ENSMODG00000007007, ENSMODG00000019560, ENSMODG00000015051, ENSMODG00000018983, ENSMODG00000011014, ENSMODG00000018418, ENSMODG00000001074, ENSMODG00000019548, ENSMODG00000001152, ENSMODG00000006420, ENSMODG00000002620, ENSMODG00000016890, ENSMODG00000017938, ENSMODG00000004739, ENSMODG00000005255	1.07	9.53E-01	100.00
GOTERM_MF_ALL	GO:0032549~ribonucleoside binding	17	8.333333	0.51177	ENSMODG00000012068, ENSMODG00000015165, ENSMODG00000007007, ENSMODG00000019560, ENSMODG00000015051, ENSMODG00000018983, ENSMODG00000011014, ENSMODG00000018418, ENSMODG00000001074, ENSMODG00000019548, ENSMODG00000001152, ENSMODG00000006420, ENSMODG00000002620, ENSMODG00000016890, ENSMODG00000017938, ENSMODG00000004739, ENSMODG00000005255	1.07	9.51E-01	100.00
GOTERM_MF_ALL	GO:0001882~nucleoside binding	17	8.333333	0.516884	ENSMODG00000012068, ENSMODG00000015165, ENSMODG00000007007, ENSMODG00000019560, ENSMODG00000015051, ENSMODG00000018983, ENSMODG00000011014, ENSMODG00000018418, ENSMODG00000001074, ENSMODG00000019548, ENSMODG00000001152, ENSMODG00000006420, ENSMODG00000002620, ENSMODG00000016890, ENSMODG00000017938, ENSMODG00000004739, ENSMODG00000005255	1.07	9.52E-01	100.00
Annotation Cluster 62	MAPK function	Enrichment Score: 0.2622931505067298						
Category	Term	Count	%	PValue	Genes	Fold Enrichment	Benjamini	FDR
GOTERM_BP_ALL	GO:0043408~regulation of MAPK cascade	7	3.431373	0.538137	ENSMODG00000007648, ENSMODG00000001183, ENSMODG00000001074, ENSMODG00000029105, ENSMODG00000018991, ENSMODG00000003112, ENSMODG00000018947	1.18	9.67E-01	100.00
GOTERM_BP_ALL	GO:0000165~MAPK cascade	7	3.431373	0.549795	ENSMODG00000007648, ENSMODG00000001183, ENSMODG00000001074, ENSMODG00000029105, ENSMODG00000018991, ENSMODG00000003112, ENSMODG00000018947	1.17	9.70E-01	100.00
GOTERM_BP_ALL	GO:0023014~signal transduction by protein phosphorylation	7	3.431373	0.552111	ENSMODG00000007648, ENSMODG00000001183, ENSMODG00000001074, ENSMODG00000029105, ENSMODG00000018991, ENSMODG00000003112, ENSMODG00000018947	1.17	9.71E-01	100.00
Annotation Cluster 63	Membrane function	Enrichment Score: 0.2564667662477175						
Category	Term	Count	%	PValue	Genes	Fold Enrichment	Benjamini	FDR
GOTERM_CC_ALL	GO:0098857~membrane microdomain	3	1.470588	0.517358	ENSMODG00000014680, ENSMODG00000014607, ENSMODG00000000954	1.73	9.31E-01	99.99
GOTERM_CC_ALL	GO:0045121~membrane raft	3	1.470588	0.517358	ENSMODG00000014680, ENSMODG00000014607, ENSMODG00000000954	1.73	9.31E-01	99.99
GOTERM_CC_ALL	GO:0098589~membrane region	3	1.470588	0.635356	ENSMODG00000014680, ENSMODG00000014607, ENSMODG00000000954	1.39	9.68E-01	100.00
Annotation Cluster 64	Homeostasis	Enrichment Score: 0.24658033316573888						

Category	Term	Count	%	PValue	Genes	Fold Enrichment	Benjamini	FDR
GOTERM_BP_ALL	GO:0006875~cellular metal ion homeostasis	5	2.45098	0.490542	ENSMODG00000019403, ENSMODG00000009157, ENSMODG00000018766, ENSMODG00000020725, ENSMODG00000000954	1.37	9.55E-01	100.00
GOTERM_BP_ALL	GO:0030003~cellular cation homeostasis	5	2.45098	0.596248	ENSMODG00000019403, ENSMODG00000009157, ENSMODG00000018766, ENSMODG00000020725, ENSMODG00000000954	1.20	9.79E-01	100.00
GOTERM_BP_ALL	GO:0006873~cellular ion homeostasis	5	2.45098	0.622524	ENSMODG00000019403, ENSMODG00000009157, ENSMODG00000018766, ENSMODG00000020725, ENSMODG00000000954	1.16	9.83E-01	100.00
Annotation Cluster 65	Kinase	Enrichment Score: 0.23427982342518625						
Category	Term	Count	%	PValue	Genes	Fold Enrichment	Benjamini	FDR
GOTERM_BP_ALL	GO:0006469~negative regulation of protein kinase activity	3	1.470588	0.544642	ENSMODG00000001183, ENSMODG00000001074, ENSMODG00000003513	1.64	9.69E-01	100.00
GOTERM_BP_ALL	GO:0033673~negative regulation of kinase activity	3	1.470588	0.581098	ENSMODG00000001183, ENSMODG00000001074, ENSMODG00000003513	1.54	9.76E-01	100.00
GOTERM_BP_ALL	GO:0051348~negative regulation of transferase activity	3	1.470588	0.626325	ENSMODG00000001183, ENSMODG00000001074, ENSMODG00000003513	1.41	9.84E-01	100.00
Annotation Cluster 66	Ion transport	Enrichment Score: 0.21664149334244834						
Category	Term	Count	%	PValue	Genes	Fold Enrichment	Benjamini	FDR
GOTERM_BP_ALL	GO:0070509~calcium ion import	3	1.470588	0.333264	ENSMODG00000019403, ENSMODG00000009157, ENSMODG00000020725	2.51	8.75E-01	99.93
GOTERM_BP_ALL	GO:0006816~calcium ion transport	3	1.470588	0.683828	ENSMODG00000019403, ENSMODG00000009157, ENSMODG00000020725	1.27	9.91E-01	100.00
GOTERM_BP_ALL	GO:0072511~divalent inorganic cation transport	3	1.470588	0.772413	ENSMODG00000019403, ENSMODG00000009157, ENSMODG00000020725	1.07	9.97E-01	100.00
GOTERM_BP_ALL	GO:0070838~divalent metal ion transport	3	1.470588	0.772413	ENSMODG00000019403, ENSMODG00000009157, ENSMODG00000020725	1.07	9.97E-01	100.00
Annotation Cluster 67	Protein function	Enrichment Score: 0.19897517100619058						
Category	Term	Count	%	PValue	Genes	Fold Enrichment	Benjamini	FDR
GOTERM_BP_ALL	GO:0036211~protein modification process	29	14.21569	0.597306	ENSMODG00000002143, ENSMODG00000003766, ENSMODG00000018991, ENSMODG00000003112, ENSMODG00000010173, ENSMODG00000011338, ENSMODG00000001183, ENSMODG00000019403, ENSMODG00000004657, ENSMODG00000020725, ENSMODG00000018947, ENSMODG00000003513, ENSMODG00000019334, ENSMODG00000012068, ENSMODG00000007648, ENSMODG00000029105, ENSMODG00000020329, ENSMODG00000019560, ENSMODG00000010808, ENSMODG00000015051, ENSMODG00000019579, ENSMODG00000003856, ENSMODG00000009785, ENSMODG00000001074, ENSMODG00000004260, ENSMODG00000003977, ENSMODG00000002910, ENSMODG00000021299, ENSMODG00000004739	1.00	9.79E-01	100.00
GOTERM_BP_ALL	GO:0006464~cellular protein modification process	29	14.21569	0.597306	ENSMODG00000002143, ENSMODG00000003766, ENSMODG00000018991, ENSMODG00000003112, ENSMODG00000010173, ENSMODG00000011338, ENSMODG00000001183, ENSMODG00000019403, ENSMODG00000004657, ENSMODG00000020725, ENSMODG00000018947, ENSMODG00000003513, ENSMODG00000019334, ENSMODG00000012068, ENSMODG00000007648, ENSMODG00000029105, ENSMODG00000020329, ENSMODG00000019560, ENSMODG00000010808, ENSMODG00000015051, ENSMODG00000019579, ENSMODG00000003856, ENSMODG00000009785, ENSMODG00000001074, ENSMODG00000004260, ENSMODG00000003977, ENSMODG00000002910, ENSMODG00000021299, ENSMODG00000004739	1.00	9.79E-01	100.00
GOTERM_BP_ALL	GO:0043412~macromolecule modification	29	14.21569	0.709056	ENSMODG00000002143, ENSMODG00000003766, ENSMODG00000018991, ENSMODG00000003112, ENSMODG00000010173, ENSMODG00000011338, ENSMODG00000001183, ENSMODG00000019403, ENSMODG00000004657, ENSMODG00000020725, ENSMODG00000018947, ENSMODG00000003513, ENSMODG00000019334, ENSMODG00000012068, ENSMODG00000007648, ENSMODG00000029105, ENSMODG00000020329, ENSMODG00000019560, ENSMODG00000010808, ENSMODG00000015051, ENSMODG00000019579, ENSMODG00000003856, ENSMODG00000009785, ENSMODG00000001074, ENSMODG00000004260, ENSMODG00000003977, ENSMODG00000002910, ENSMODG00000021299, ENSMODG00000004739	0.95	9.93E-01	100.00
Annotation Cluster 68	Biosynthesis	Enrichment Score: 0.18634876085056826						
Category	Term	Count	%	PValue	Genes	Fold Enrichment	Benjamini	FDR
GOTERM_BP_ALL	GO:0009152~purine ribonucleotide biosynthetic process	3	1.470588	0.557034	ENSMODG00000007007, ENSMODG00000000658, ENSMODG00000008171	1.61	9.72E-01	100.00
GOTERM_BP_ALL	GO:0006164~purine nucleotide biosynthetic process	3	1.470588	0.577154	ENSMODG00000007007, ENSMODG00000000658, ENSMODG00000008171	1.55	9.75E-01	100.00
GOTERM_BP_ALL	GO:0009260~ribonucleotide biosynthetic process	3	1.470588	0.592768	ENSMODG00000007007, ENSMODG00000000658, ENSMODG00000008171	1.50	9.78E-01	100.00
GOTERM_BP_ALL	GO:0046390~ribose phosphate biosynthetic process	3	1.470588	0.596604	ENSMODG00000007007, ENSMODG00000000658, ENSMODG00000008171	1.49	9.79E-01	100.00

GOTERM_BP_ALL	GO:0072522~purine-containing compound biosynthetic process	3	1.470588	0.615381	ENSMODG00000007007, ENSMODG00000000658, ENSMODG00000008171	1.44	9.82E-01	100.00
GOTERM_BP_ALL	GO:0009165~nucleotide biosynthetic process	3	1.470588	0.711245	ENSMODG00000007007, ENSMODG00000000658, ENSMODG00000008171	1.21	9.93E-01	100.00
GOTERM_BP_ALL	GO:1901293~nucleoside phosphate biosynthetic process	3	1.470588	0.722782	ENSMODG00000007007, ENSMODG00000000658, ENSMODG00000008171	1.18	9.94E-01	100.00
GOTERM_BP_ALL	GO:0090407~organophosphate biosynthetic process	3	1.470588	0.898037	ENSMODG00000007007, ENSMODG00000000658, ENSMODG00000008171	0.78	1.00E+00	100.00
Annotation Cluster 69	Metabolism	Enrichment Score: 0.17991537483644496						
Category	Term	Count	%	PValue	Genes	Fold Enrichment	Benjamini	FDR
GOTERM_BP_ALL	GO:0006163~purine nucleotide metabolic process	5	2.45098	0.517357	ENSMODG00000007007, ENSMODG00000020725, ENSMODG00000000658, ENSMODG00000008171, ENSMODG00000018947	1.33	9.63E-01	100.00
GOTERM_BP_ALL	GO:0072521~purine-containing compound metabolic process	5	2.45098	0.590866	ENSMODG00000007007, ENSMODG00000020725, ENSMODG00000000658, ENSMODG00000008171, ENSMODG00000018947	1.21	9.78E-01	100.00
GOTERM_BP_ALL	GO:0009117~nucleotide metabolic process	5	2.45098	0.707905	ENSMODG00000007007, ENSMODG00000020725, ENSMODG00000000658, ENSMODG00000008171, ENSMODG00000018947	1.04	9.93E-01	100.00
GOTERM_BP_ALL	GO:0006753~nucleoside phosphate metabolic process	5	2.45098	0.728988	ENSMODG00000007007, ENSMODG00000020725, ENSMODG00000000658, ENSMODG00000008171, ENSMODG00000018947	1.01	9.94E-01	100.00
GOTERM_BP_ALL	GO:0055086~nucleobase-containing small molecule metabolic process	5	2.45098	0.798819	ENSMODG00000007007, ENSMODG00000020725, ENSMODG00000000658, ENSMODG00000008171, ENSMODG00000018947	0.91	9.98E-01	100.00
Annotation Cluster 70	Translation	Enrichment Score: 0.17826134374742528						
Category	Term	Count	%	PValue	Genes	Fold Enrichment	Benjamini	FDR
GOTERM_BP_ALL	GO:0006417~regulation of translation	4	1.960784	0.43603	ENSMODG00000011338, ENSMODG00000015125, ENSMODG00000011920, ENSMODG00000010122	1.65	9.34E-01	100.00
GOTERM_BP_ALL	GO:0034248~regulation of cellular amide metabolic process	4	1.960784	0.507634	ENSMODG00000011338, ENSMODG00000015125, ENSMODG00000011920, ENSMODG00000010122	1.50	9.60E-01	100.00
GOTERM_BP_ALL	GO:0006412~translation	4	1.960784	0.931592	ENSMODG00000011338, ENSMODG00000015125, ENSMODG00000011920, ENSMODG00000010122	0.69	1.00E+00	100.00
GOTERM_BP_ALL	GO:0043043~peptide biosynthetic process	4	1.960784	0.938991	ENSMODG00000011338, ENSMODG00000015125, ENSMODG00000011920, ENSMODG00000010122	0.67	1.00E+00	100.00
Annotation Cluster 71	Focal adhesion	Enrichment Score: 0.16305189764222416						
Category	Term	Count	%	PValue	Genes	Fold Enrichment	Benjamini	FDR
GOTERM_CC_ALL	GO:0005925~focal adhesion	4	1.960784	0.679715	ENSMODG00000010369, ENSMODG00000004834, ENSMODG00000003513, ENSMODG00000018983	1.14	9.78E-01	100.00
GOTERM_CC_ALL	GO:0005924~cell-substrate adherens junction	4	1.960784	0.687054	ENSMODG00000010369, ENSMODG00000004834, ENSMODG00000003513, ENSMODG00000018983	1.13	9.78E-01	100.00
GOTERM_CC_ALL	GO:0030055~cell-substrate junction	4	1.960784	0.694267	ENSMODG00000010369, ENSMODG00000004834, ENSMODG00000003513, ENSMODG00000018983	1.12	9.79E-01	100.00
Annotation Cluster 72	Metabolism	Enrichment Score: 0.14507616109456714						
Category	Term	Count	%	PValue	Genes	Fold Enrichment	Benjamini	FDR
GOTERM_BP_ALL	GO:0009150~purine ribonucleotide metabolic process	4	1.960784	0.69268	ENSMODG00000007007, ENSMODG00000000658, ENSMODG00000008171, ENSMODG00000018947	1.12	9.91E-01	100.00
GOTERM_BP_ALL	GO:0009259~ribonucleotide metabolic process	4	1.960784	0.719868	ENSMODG00000007007, ENSMODG00000000658, ENSMODG00000008171, ENSMODG00000018947	1.07	9.94E-01	100.00
GOTERM_BP_ALL	GO:0019693~ribose phosphate metabolic process	4	1.960784	0.736183	ENSMODG00000007007, ENSMODG00000000658, ENSMODG00000008171, ENSMODG00000018947	1.05	9.95E-01	100.00
Annotation Cluster 73	Biosynthesis	Enrichment Score: 0.13875349382596516						
Category	Term	Count	%	PValue	Genes	Fold Enrichment	Benjamini	FDR

GOTERM_BP_ALL	GO:0044249~cellular biosynthetic process	45	22.05882	0.681161	ENSMODG00000002143, ENSMODG00000016375, ENSMODG00000012059, ENSMODG00000018991, ENSMODG00000007007, ENSMODG00000000658, ENSMODG00000010122, ENSMODG00000003112, ENSMODG00000010173, ENSMODG00000011437, ENSMODG00000011338, ENSMODG00000001183, ENSMODG00000000453, ENSMODG00000004657, ENSMODG00000001161, ENSMODG00000020725, ENSMODG00000002137, ENSMODG00000013409, ENSMODG00000021156, ENSMODG00000016387, ENSMODG00000006068, ENSMODG00000015125, ENSMODG00000029105, ENSMODG00000015171, ENSMODG00000018927, ENSMODG00000008171, ENSMODG00000007847, ENSMODG00000012390, ENSMODG00000024585, ENSMODG00000002992, ENSMODG00000011014, ENSMODG00000003856, ENSMODG00000014680, ENSMODG00000001074, ENSMODG00000014926, ENSMODG00000011920, ENSMODG00000001152, ENSMODG00000003548, ENSMODG00000004260, ENSMODG00000016890, ENSMODG00000018597, ENSMODG00000002910, ENSMODG00000021299, ENSMODG00000014540, ENSMODG00000015403	0.97	9.90E-01	100.00
GOTERM_BP_ALL	GO:1901576~organic substance biosynthetic process	45	22.05882	0.724051	ENSMODG00000002143, ENSMODG00000016375, ENSMODG00000012059, ENSMODG00000018991, ENSMODG00000007007, ENSMODG00000000658, ENSMODG00000010122, ENSMODG00000003112, ENSMODG00000010173, ENSMODG00000011437, ENSMODG00000011338, ENSMODG00000001183, ENSMODG00000000453, ENSMODG00000004657, ENSMODG00000001161, ENSMODG00000020725, ENSMODG00000002137, ENSMODG00000013409, ENSMODG00000021156, ENSMODG00000016387, ENSMODG00000006068, ENSMODG00000015125, ENSMODG00000029105, ENSMODG00000015171, ENSMODG00000018927, ENSMODG00000008171, ENSMODG00000007847, ENSMODG00000012390, ENSMODG00000024585, ENSMODG00000002992, ENSMODG00000011014, ENSMODG00000003856, ENSMODG00000014680, ENSMODG00000001074, ENSMODG00000014926, ENSMODG00000011920, ENSMODG00000001152, ENSMODG00000003548, ENSMODG00000004260, ENSMODG00000016890, ENSMODG00000018597, ENSMODG00000002910, ENSMODG00000021299, ENSMODG00000014540, ENSMODG00000015403	0.95	9.94E-01	100.00
GOTERM_BP_ALL	GO:0009058~biosynthetic process	45	22.05882	0.777536	ENSMODG00000002143, ENSMODG00000016375, ENSMODG00000012059, ENSMODG00000018991, ENSMODG00000007007, ENSMODG00000000658, ENSMODG00000010122, ENSMODG00000003112, ENSMODG00000010173, ENSMODG00000011437, ENSMODG00000011338, ENSMODG00000001183, ENSMODG00000000453, ENSMODG00000004657, ENSMODG00000001161, ENSMODG00000020725, ENSMODG00000002137, ENSMODG00000013409, ENSMODG00000021156, ENSMODG00000016387, ENSMODG00000006068, ENSMODG00000015125, ENSMODG00000029105, ENSMODG00000015171, ENSMODG00000018927, ENSMODG00000008171, ENSMODG00000007847, ENSMODG00000012390, ENSMODG00000024585, ENSMODG00000002992, ENSMODG00000011014, ENSMODG00000003856, ENSMODG00000014680, ENSMODG00000001074, ENSMODG00000014926, ENSMODG00000011920, ENSMODG00000001152, ENSMODG00000003548, ENSMODG00000004260, ENSMODG00000016890, ENSMODG00000018597, ENSMODG00000002910, ENSMODG00000021299, ENSMODG00000014540, ENSMODG00000015403	0.93	9.97E-01	100.00
Annotation Cluster 74	Transcription	Enrichment Score: 0.12063409055644805						
Category	Term	Count	%	PValue	Genes	Fold Enrichment	Benjamini	FDR
GOTERM_MF_ALL	GO:0003712~transcription cofactor activity	4	1.960784	0.701051	ENSMODG00000011338, ENSMODG00000011920, ENSMODG00000018991, ENSMODG00000016176	1.11	9.90E-01	100.00
GOTERM_MF_ALL	GO:0000989~transcription factor activity, transcription factor binding	4	1.960784	0.784107	ENSMODG00000011338, ENSMODG00000011920, ENSMODG00000018991, ENSMODG00000016176	0.97	9.97E-01	100.00
GOTERM_MF_ALL	GO:0000988~transcription factor activity, protein binding	4	1.960784	0.790629	ENSMODG00000011338, ENSMODG00000011920, ENSMODG00000018991, ENSMODG00000016176	0.95	9.97E-01	100.00
Annotation Cluster 75	Immunity	Enrichment Score: 0.12035649031194827						
Category	Term	Count	%	PValue	Genes	Fold Enrichment	Benjamini	FDR
GOTERM_BP_ALL	GO:0050863~regulation of T cell activation	3	1.470588	0.585015	ENSMODG00000005170, ENSMODG00000007847, ENSMODG00000018947	1.53	9.77E-01	100.00
GOTERM_BP_ALL	GO:0051249~regulation of lymphocyte activation	3	1.470588	0.777162	ENSMODG00000005170, ENSMODG00000007847, ENSMODG00000018947	1.06	9.97E-01	100.00
GOTERM_BP_ALL	GO:0002694~regulation of leukocyte activation	3	1.470588	0.835098	ENSMODG00000005170, ENSMODG00000007847, ENSMODG00000018947	0.93	9.99E-01	100.00
GOTERM_BP_ALL	GO:0050865~regulation of cell activation	3	1.470588	0.869276	ENSMODG00000005170, ENSMODG00000007847, ENSMODG00000018947	0.85	9.99E-01	100.00
Annotation Cluster 76	Catabolism	Enrichment Score: 0.12022142787197503						

Category	Term	Count	%	PValue	Genes	Fold Enrichment	Benjamini	FDR
GOTERM_BP_ALL	GO:0034655~nucleobase-containing compound catabolic process	3	1.470588	0.699312	ENSMODG00000010122, ENSMODG00000018597, ENSMODG00000018947	1.23	9.92E-01	100.00
GOTERM_BP_ALL	GO:0044270~cellular nitrogen compound catabolic process	3	1.470588	0.76265	ENSMODG00000010122, ENSMODG00000018597, ENSMODG00000018947	1.09	9.96E-01	100.00
GOTERM_BP_ALL	GO:0046700~heterocycle catabolic process	3	1.470588	0.76265	ENSMODG00000010122, ENSMODG00000018597, ENSMODG00000018947	1.09	9.96E-01	100.00
GOTERM_BP_ALL	GO:0019439~aromatic compound catabolic process	3	1.470588	0.772413	ENSMODG00000010122, ENSMODG00000018597, ENSMODG00000018947	1.07	9.97E-01	100.00
GOTERM_BP_ALL	GO:1901361~organic cyclic compound catabolic process	3	1.470588	0.797483	ENSMODG00000010122, ENSMODG00000018597, ENSMODG00000018947	1.01	9.98E-01	100.00
Annotation Cluster 77	Homeostasis	Enrichment Score: 0.0979783146588978						
Category	Term	Count	%	PValue	Genes	Fold Enrichment	Benjamini	FDR
GOTERM_BP_ALL	GO:0006874~cellular calcium ion homeostasis	3	1.470588	0.767576	ENSMODG00000019403, ENSMODG00000018766, ENSMODG00000020725	1.08	9.97E-01	100.00
GOTERM_BP_ALL	GO:0055074~calcium ion homeostasis	3	1.470588	0.790898	ENSMODG00000019403, ENSMODG00000018766, ENSMODG00000020725	1.03	9.98E-01	100.00
GOTERM_BP_ALL	GO:0072503~cellular divalent inorganic cation homeostasis	3	1.470588	0.801772	ENSMODG00000019403, ENSMODG00000018766, ENSMODG00000020725	1.00	9.98E-01	100.00
GOTERM_BP_ALL	GO:0072507~divalent inorganic cation homeostasis	3	1.470588	0.833286	ENSMODG00000019403, ENSMODG00000018766, ENSMODG00000020725	0.93	9.99E-01	100.00
Annotation Cluster 78	Cellular organization	Enrichment Score: 0.07984805702560724						
Category	Term	Count	%	PValue	Genes	Fold Enrichment	Benjamini	FDR
GOTERM_CC_ALL	GO:0043233~organelle lumen	27	13.23529	0.82509	ENSMODG00000021156, ENSMODG00000016387, ENSMODG00000006068, ENSMODG00000016375, ENSMODG00000029105, ENSMODG00000015171, ENSMODG00000025001, ENSMODG00000007847, ENSMODG00000001830, ENSMODG00000010173, ENSMODG00000011437, ENSMODG00000010536, ENSMODG00000012390, ENSMODG00000011338, ENSMODG00000019899, ENSMODG00000003856, ENSMODG00000001074, ENSMODG00000014926, ENSMODG00000001152, ENSMODG00000003548, ENSMODG00000004260, ENSMODG00000016890, ENSMODG00000002137, ENSMODG00000002910, ENSMODG00000016420, ENSMODG00000014540, ENSMODG00000015403	0.89	9.95E-01	100.00
GOTERM_CC_ALL	GO:0070013~intracellular organelle lumen	27	13.23529	0.82509	ENSMODG00000021156, ENSMODG00000016387, ENSMODG00000006068, ENSMODG00000016375, ENSMODG00000029105, ENSMODG00000015171, ENSMODG00000025001, ENSMODG00000007847, ENSMODG00000001830, ENSMODG00000010173, ENSMODG00000011437, ENSMODG00000010536, ENSMODG00000012390, ENSMODG00000011338, ENSMODG00000019899, ENSMODG00000003856, ENSMODG00000001074, ENSMODG00000014926, ENSMODG00000001152, ENSMODG00000003548, ENSMODG00000004260, ENSMODG00000016890, ENSMODG00000002137, ENSMODG00000002910, ENSMODG00000016420, ENSMODG00000014540, ENSMODG00000015403	0.89	9.95E-01	100.00
GOTERM_CC_ALL	GO:0031974~membrane-enclosed lumen	27	13.23529	0.846161	ENSMODG00000021156, ENSMODG00000016387, ENSMODG00000006068, ENSMODG00000016375, ENSMODG00000029105, ENSMODG00000015171, ENSMODG00000025001, ENSMODG00000007847, ENSMODG00000001830, ENSMODG00000010173, ENSMODG00000011437, ENSMODG00000010536, ENSMODG00000012390, ENSMODG00000011338, ENSMODG00000019899, ENSMODG00000003856, ENSMODG00000001074, ENSMODG00000014926, ENSMODG00000001152, ENSMODG00000003548, ENSMODG00000004260, ENSMODG00000016890, ENSMODG00000002137, ENSMODG00000002910, ENSMODG00000016420, ENSMODG00000014540, ENSMODG00000015403	0.88	9.96E-01	100.00
Annotation Cluster 79	Signaling	Enrichment Score: 0.025179988282000455						
Category	Term	Count	%	PValue	Genes	Fold Enrichment	Benjamini	FDR
GOTERM_BP_ALL	GO:0098916~anterograde trans-synaptic signaling	3	1.470588	0.94367	ENSMODG00000015598, ENSMODG00000009510, ENSMODG00000019560	0.66	1.00E+00	100.00
GOTERM_BP_ALL	GO:0099536~synaptic signaling	3	1.470588	0.94367	ENSMODG00000015598, ENSMODG00000009510, ENSMODG00000019560	0.66	1.00E+00	100.00
GOTERM_BP_ALL	GO:0099537~trans-synaptic signaling	3	1.470588	0.94367	ENSMODG00000015598, ENSMODG00000009510, ENSMODG00000019560	0.66	1.00E+00	100.00
GOTERM_BP_ALL	GO:0007268~chemical synaptic transmission	3	1.470588	0.94367	ENSMODG00000015598, ENSMODG00000009510, ENSMODG00000019560	0.66	1.00E+00	100.00
Annotation Cluster 80	Protein assembly	Enrichment Score: 0.020299020661705816						
Category	Term	Count	%	PValue	Genes	Fold Enrichment	Benjamini	FDR
GOTERM_BP_ALL	GO:0006461~protein complex assembly	7	3.431373	0.937356	ENSMODG00000015598, ENSMODG00000021426, ENSMODG00000018991, ENSMODG00000010536, ENSMODG00000008346, ENSMODG00000005162, ENSMODG00000005255	0.70	1.00E+00	100.00
GOTERM_BP_ALL	GO:0070271~protein complex biogenesis	7	3.431373	0.937356	ENSMODG00000015598, ENSMODG00000021426, ENSMODG00000018991, ENSMODG00000010536, ENSMODG00000008346, ENSMODG00000005162, ENSMODG00000005255	0.70	1.00E+00	100.00

GOTERM_BP_ALL	GO:0065003~macromolecular complex assembly	7	3.431373	0.989221	ENSMODG00000015598, ENSMODG00000021426, ENSMODG00000018991, ENSMODG00000010536, ENSMODG00000008346, ENSMODG00000005162, ENSMODG00000005255	0.55	1.00E+00	100.00
Annotation Cluster 81	Catabolism	Enrichment Score: 0.007736663409725543						
Category	Term	Count	%	PValue	Genes	Fold Enrichment	Benjamini	FDR
GOTERM_BP_ALL	GO:0006511~ubiquitin-dependent protein catabolic process	3	1.470588	0.974306	ENSMODG00000018927, ENSMODG00000002910, ENSMODG00000021299	0.55	1.00E+00	100.00
GOTERM_BP_ALL	GO:0019941~modification-dependent protein catabolic process	3	1.470588	0.976427	ENSMODG00000018927, ENSMODG00000002910, ENSMODG00000021299	0.54	1.00E+00	100.00
GOTERM_BP_ALL	GO:0043632~modification-dependent macromolecule catabolic process	3	1.470588	0.97838	ENSMODG00000018927, ENSMODG00000002910, ENSMODG00000021299	0.53	1.00E+00	100.00
GOTERM_BP_ALL	GO:0051603~proteolysis involved in cellular protein catabolic process	3	1.470588	0.990063	ENSMODG00000018927, ENSMODG00000002910, ENSMODG00000021299	0.46	1.00E+00	100.00
GOTERM_BP_ALL	GO:0044257~cellular protein catabolic process	3	1.470588	0.992683	ENSMODG00000018927, ENSMODG00000002910, ENSMODG00000021299	0.44	1.00E+00	100.00

Supplementary Table 7. KEGG pathway analysis of shared upregulated uterine genes in early pregnancy compared to non-reproductive state between *Monodelphis domestica* and *Sminthopsis crassicaudata* .								
**Category**	**Term**	**Count**	**%**	**PValue**	**Genes**	**Fold Enrichment**	**Benjamini**	**FDR**
KEGG_PATHWAY	mdo01100:Metabolic pathways	33	17.83783784	1.03E-06	ENSMODG00000007831, ENSMODG00000004392, ENSMODG00000005737, ENSMODG00000012667, ENSMODG00000010248, ENSMODG00000019574, ENSMODG00000015800, ENSMODG00000003065, ENSMODG00000009941, ENSMODG00000009610, ENSMODG00000014033, ENSMODG00000017776, ENSMODG00000029505, ENSMODG00000003558, ENSMODG00000001738, ENSMODG00000016512, ENSMODG00000012906, ENSMODG00000015775, ENSMODG00000015439, ENSMODG00000014843, ENSMODG00000005175, ENSMODG00000013285, ENSMODG00000014803, ENSMODG00000003073, ENSMODG00000017363, ENSMODG00000005464, ENSMODG00000023342, ENSMODG00000014990, ENSMODG00000019459, ENSMODG00000009825, ENSMODG00000016356, ENSMODG00000009431, ENSMODG00000017742	2.36	1.19E-04	0.00
KEGG_PATHWAY	mdo03060:Protein export	5	2.702702703	1.55E-04	ENSMODG00000019764, ENSMODG00000004576, ENSMODG00000012664, ENSMODG00000020427, ENSMODG00000016096	17.55	8.96E-03	0.18
KEGG_PATHWAY	mdo04141:Protein processing in endoplasmic reticulum	9	4.864864865	6.19E-04	ENSMODG00000010088, ENSMODG00000019764, ENSMODG00000020424, ENSMODG00000012664, ENSMODG00000013318, ENSMODG00000013307, ENSMODG00000016096, ENSMODG00000004556, ENSMODG00000014811	4.62	2.37E-02	0.70
KEGG_PATHWAY	mdo00510:N-Glycan biosynthesis	5	2.702702703	0.002671998	ENSMODG00000023342, ENSMODG00000012667, ENSMODG00000016356, ENSMODG00000005175, ENSMODG00000013285	8.42	7.47E-02	3.01
KEGG_PATHWAY	mdo04966:Collecting duct acid secretion	4	2.162162162	0.002958559	ENSMODG00000015439, ENSMODG00000009825, ENSMODG00000019574, ENSMODG00000014803	13.47	6.64E-02	3.32
KEGG_PATHWAY	mdo04721:Synaptic vesicle cycle	5	2.702702703	0.005180414	ENSMODG00000015439, ENSMODG00000009825, ENSMODG00000019574, ENSMODG00000009431, ENSMODG00000014803	7.02	9.55E-02	5.75
KEGG_PATHWAY	mdo05323:Rheumatoid arthritis	5	2.702702703	0.010809861	ENSMODG00000015439, ENSMODG00000009825, ENSMODG00000019574, ENSMODG00000009431, ENSMODG00000014803	5.69	1.65E-01	11.66
KEGG_PATHWAY	mdo04964:Proximal tubule bicarbonate reclamation	3	1.621621622	0.018377913	ENSMODG00000002920, ENSMODG00000016512, ENSMODG00000003073	14.04	2.36E-01	19.07
KEGG_PATHWAY	mdo00190:Oxidative phosphorylation	6	3.243243243	0.01837868	ENSMODG00000015439, ENSMODG00000018503, ENSMODG00000009825, ENSMODG00000019574, ENSMODG00000009431, ENSMODG00000014803	3.86	2.13E-01	19.07
KEGG_PATHWAY	mdo04145:Phagosome	6	3.243243243	0.023110914	ENSMODG00000015439, ENSMODG00000012664, ENSMODG00000009825, ENSMODG00000019574, ENSMODG00000009431, ENSMODG00000014803	3.64	2.38E-01	23.41
KEGG_PATHWAY	mdo03320:PPAR signaling pathway	4	2.162162162	0.040281742	ENSMODG00000007494, ENSMODG00000029505, ENSMODG00000016512, ENSMODG00000000393	5.18	3.52E-01	37.44
KEGG_PATHWAY	mdo00020:Citrate cycle (TCA cycle)	3	1.621621622	0.042120555	ENSMODG00000007831, ENSMODG00000016512, ENSMODG00000003073	9.02	3.40E-01	38.80
KEGG_PATHWAY	mdo04142:Lysosome	5	2.702702703	0.049174769	ENSMODG00000015439, ENSMODG00000004631, ENSMODG00000017579, ENSMODG00000009431, ENSMODG00000005871	3.57	3.62E-01	43.75
KEGG_PATHWAY	mdo00565:Ether lipid metabolism	3	1.621621622	0.086469548	ENSMODG00000009610, ENSMODG00000014033, ENSMODG00000015800	6.02	5.27E-01	64.36
KEGG_PATHWAY	mdo00620:Pyruvate metabolism	3	1.621621622	0.086469548	ENSMODG00000007831, ENSMODG00000016512, ENSMODG00000003073	6.02	5.27E-01	64.36
KEGG_PATHWAY	mdo01130:Biosynthesis of antibiotics	6	3.243243243	0.090395435	ENSMODG00000009941, ENSMODG00000017363, ENSMODG00000014033, ENSMODG00000003558, ENSMODG00000016512, ENSMODG00000003073	2.48	5.19E-01	66.07

Supplementary Table 8. KEGG pathway analysis of shared downregulated uterine genes in early pregnancy compared to non-reproductive state between *Monodelphis domestica* and *Sminthopsis crassicaudata* .

Category	Term	Count	%	PValue	Genes	Fold Enrichment	Benjamini	FDR
KEGG_PATHWAY	mdo04360:Axon guidance	8	3.921569	2.43E-04	ENSMODG00000016811, ENSMODG00000018325, ENSMODG00000012511, ENSMODG00000016857, ENSMODG00000004739, ENSMODG00000005255, ENSMODG00000002992, ENSMODG00000019334	6.24	2.85E-02	0.28
KEGG_PATHWAY	mdo05200:Pathways in cancer	11	5.392157	0.004946	ENSMODG00000019416, ENSMODG00000014680, ENSMODG00000001735, ENSMODG00000002526, ENSMODG00000029105, ENSMODG00000014926, ENSMODG00000015361, ENSMODG00000004260, ENSMODG00000000658, ENSMODG00000012412, ENSMODG00000009971	2.79	2.56E-01	5.53
KEGG_PATHWAY	mdo04310:Wnt signaling pathway	6	2.941176	0.009846	ENSMODG00000001183, ENSMODG00000001735, ENSMODG00000015361, ENSMODG00000004260, ENSMODG00000013125, ENSMODG00000002992	4.50	3.25E-01	10.72
KEGG_PATHWAY	mdo05217:Basal cell carcinoma	4	1.960784	0.013921	ENSMODG00000014680, ENSMODG00000002526, ENSMODG00000015361, ENSMODG00000004260	7.77	3.41E-01	14.84
KEGG_PATHWAY	mdo04022:cGMP-PKG signaling pathway	6	2.941176	0.018138	ENSMODG00000009157, ENSMODG00000007007, ENSMODG00000000658, ENSMODG00000000954, ENSMODG00000018947, ENSMODG00000002992	3.86	3.53E-01	18.93
KEGG_PATHWAY	mdo04390:Hippo signaling pathway	6	2.941176	0.019112	ENSMODG00000001735, ENSMODG00000011920, ENSMODG00000029105, ENSMODG00000015361, ENSMODG00000004260, ENSMODG00000010173	3.81	3.18E-01	19.84
KEGG_PATHWAY	mdo00230:Purine metabolism	6	2.941176	0.035006	ENSMODG00000015171, ENSMODG00000007007, ENSMODG00000005727, ENSMODG00000008171, ENSMODG00000011437, ENSMODG00000018947	3.25	4.54E-01	33.53
KEGG_PATHWAY	mdo03030:DNA replication	3	1.470588	0.045372	ENSMODG00000001152, ENSMODG00000015171, ENSMODG00000011437	8.65	4.99E-01	41.27
KEGG_PATHWAY	mdo04080:Neuroactive ligand-receptor interaction	7	3.431373	0.072527	ENSMODG00000015831, ENSMODG00000009510, ENSMODG00000001161, ENSMODG00000000658, ENSMODG00000018290, ENSMODG00000014746, ENSMODG00000015872	2.35	6.30E-01	57.81
KEGG_PATHWAY	mdo03420:Nucleotide excision repair	3	1.470588	0.072683	ENSMODG00000015171, ENSMODG00000019904, ENSMODG00000011437	6.64	5.93E-01	57.89
KEGG_PATHWAY	mdo05206:MicroRNAs in cancer	6	2.941176	0.077875	ENSMODG00000021156, ENSMODG00000001735, ENSMODG00000001074, ENSMODG00000007847, ENSMODG00000014540, ENSMODG00000003112	2.58	5.84E-01	60.52
